# Mechanochemical Activation of NaHCO_3_: A Solid CO_2_ Surrogate in Carboxylation Reactions

**DOI:** 10.1002/cssc.202500461

**Published:** 2025-06-02

**Authors:** Francesco Mele, Andrea Aquilini, Ana Maria Constantin, Francesco Pancrazzi, Lara Righi, Andrea Porcheddu, Raimondo Maggi, Daniele Alessandro Cauzzi, Giovanni Maestri, Elena Motti, Luca Capaldo, Nicola Della Ca'

**Affiliations:** ^1^ SynCat Lab Department of Chemistry Life Sciences and Environmental Sustainability (SCVSA) University of Parma Parco Area delle Scienze, 17/A 43124 Parma Italy; ^2^ Department of Chemistry Life Sciences and Environmental Sustainability (SCVSA) University of Parma Parco Area delle Scienze, 17/A 43124 Parma Italy; ^3^ Department of Chemical and Geological Science University of Cagliari Monserrato 09042 Cagliari Italy; ^4^ CIRCC (Interuniversity Consortium Chemical Reactivity and Catalysis) Via Celso Ulpiani 27 Bari 70126 Italy

**Keywords:** carbamates, carbonates, carboxylation, mechanochemical activation, sodium bicarbonate

## Abstract

Carbon dioxide, a primary driver of global warming, offers a promising feedstock for valuable chemical synthesis. Nonetheless, the reliance on highly pressurized canisters and specialized equipment limits its practical application in fine chemical synthesis. This study explores the innovative use of sodium bicarbonate (NaHCO_3_) as a safe, solid, on‐demand source of CO_2_ under mechanochemical conditions to perform carboxylation reactions. Two applications of the practical mechanochemical syntheses using sodium bicarbonate are developed: first, the synthesis of cyclic carbamates from propargylic amines is investigated, and second, cyclic organic carbonates are derived from epoxides. The potential of this approach in the pharmaceutical industry is further showcased by demonstrating the solvent‐minimized synthesis of pharmaceutically relevant molecules and introducing a ^13^C‐labeling strategy utilizing NaH^13^CO_3_.

## Graphical abstract

This study presents sodium bicarbonate (NaHCO_3_) as a safe, solid source of CO_2_ for mechanochemical carboxylation reactions. Mechanochemical methods for synthesizing cyclic carbamates and organic carbonates from NaHCO_3_ are developed. This approach holds significant potential for pharmaceutical applications, highlighting solvent‐minimized synthesis and an innovative ^13^C‐labeling strategy.

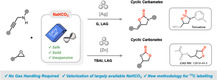

## 1 Introduction

**Figure 1:**
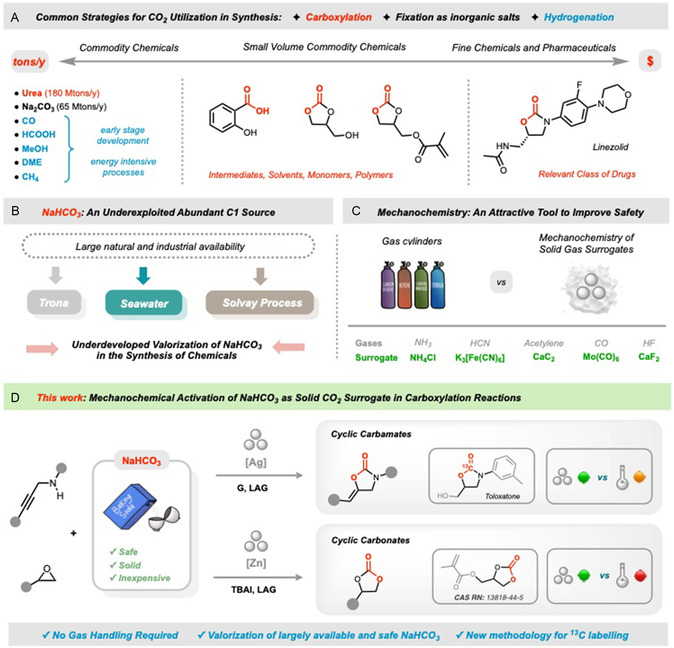
A) CO_2_ as valuable source of carbon for chemicals and fuels. B) NaHCO_3_ as a natural and safe CO_2_ surrogate. C) Mechanochemical strategies to replace gaseous reactants. D) *This work* (*G* = guanidine; TBAI = tetrabutylammonium iodide; LAG = Liquid Assisted Grinding).

CO_2_ is the principal greenhouse gas and the main responsible of the global warming. From the beginning of the industrial revolution, the increasing impact of human activities has been causing irreversible effects on the planet and, currently, the reduction of CO_2_ emissions has become one of the main priorities for society.^[^ ^1^ ^]^ CO_2_, once regarded as a waste product, is now recognized as a renewable source of carbon that holds potential for synthesizing a diverse array of industrially significant products, including chemicals, fuels, and materials (**Figure** **1**A).^[^ ^2^, ^3^ ^]^ While reactions involving gaseous CO_2_ can result in improved atom economy, CO_2_‐to‐Chemicals (C2C) processes are hindered by the need for pressurized canisters and specialized equipment.^[^ ^4^, ^5^ ^]^ A breakthrough in the field would involve the introduction of a cost‐effective and readily available CO_2_ surrogate for chemical reactions.

Nature stores CO_2_ primarily as inorganic carbonates and bicarbonates. While the former represents a permanent storage solution, bicarbonate offers a more dynamic option for use in chemical synthesis as an abundant CO_2_ surrogate (Figure 1B).^[^ ^6^, ^7^, ^8^ ^]^ Our vision is to use NaHCO_3_ for the manufacturing of organic compounds under solvent‐free conditions, contributing to the indirect valorization of CO_2_. As bicarbonate is not soluble in the vast majority of organic solvents, we resorted to mechanochemistry.^[^ ^9^ ^]^ In fact, this approach allows for the design of synthetic transformations that require minimal amounts of solvents, and often none at all, greatly increasing the sustainability of chemical reactions.^[^ ^10^, ^11^, ^12^, ^13^, ^14^, ^15^, ^16^ ^]^ It has been also demonstrated that mechanochemistry can promote alternative reactivity and may improve safety of chemical reactions.^[^ ^17^, ^18^, ^19^ ^]^ For example, although solid–gas reactions are reported to be a viable synthetic strategy under ball milling conditions,^[^ ^20^, ^21^ ^]^ researchers have turned to mechanochemical techniques for the in situ generation of gases, starting from solid surrogates (Figure 1C).^[^ ^22^, ^23^, ^24^, ^25^, ^26^ ^]^ This approach avoids the storage of potentially hazardous gases and the use of specialized equipment, allowing one to further improve the safety of a process while reducing, at the same time, its complexity and cost.

The implementation of mechanochemical carboxylative reactions that employ solid CO_2_ surrogates remains restricted to a few investigations. For instance, in 2011, Pinhas et al. utilized dry ice as a solid source of CO_2_ for the synthesis of oxazolidinones from aziridines via high‐speed ball milling (HSBM) technique.^[^ ^27^ ^]^ In the same year, the same method was used to obtain dialkyl carbonates from organic halides and potassium/caesium carbonate, in the presence of cation complexing reagents and dry ice.^[^ ^28^ ^]^ The generation of diethyl carbonate from inorganic carbonates and ethyl trifluoromethanesulfonate under mechanochemical conditions has been recently elucidated by Borchardt and coworkers through implementation of both ex situ and in situ monitoring techniques.^[^ ^29^ ^]^ Sodium methyl carbonate has been employed in conjunction with Grignard reagent by Bolm's group for the synthesis of carboxylic acids under ball milling conditions.^[^ ^30^ ^]^ An intriguing study by Lewiński and coworkers explores the use of biguanidine for CO_2_ capture through solution‐based and liquid‐assisted mechanochemical methods, resulting in various biguanidinium carbonate and bicarbonate networks.^[^ ^31^ ^]^

We present here the mechanochemical activation of NaHCO_3_ for carboxylation reactions under nearly solventless conditions (Figure 1D). This method leads to industrially relevant chemicals, such as cyclic carbamates and carbonates, from NaHCO_3_ and the corresponding propargylamines or epoxides. As a widely accessible source of CO_2_, NaHCO_3_ is inexpensive, safe, easy to handle, and eliminates the need for high‐pressure equipment.

## 2 Results and Discussion

### 2.1 The Synthesis of Oxazolidinones from NaHCO_3_ and Propargylic Amine

**Figure 2:**
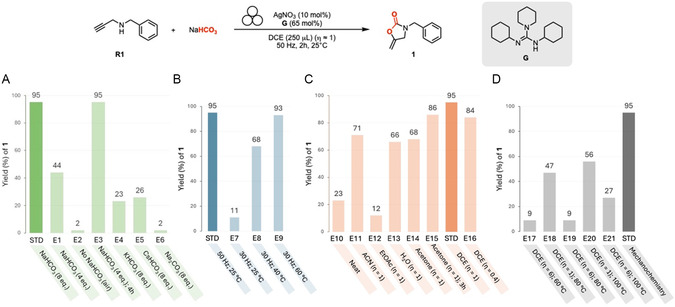
Effect of the most relevant parameters in the mechanochemical carboxylation of propargyl amine **R1** to oxazolidinone **1**. *Standard conditions*: Stainless‐steel jar of 15 mL, stainless‐steel ball (∅: 15 mm, 13.5 g), 2 h, 50 Hz, **S1** (0.3 mmol, 1 equiv.), NaHCO_3_ (2.4 mmol, 8 equiv.), **G** (0.195 mmol, 65 mol%), AgNO_3_ (0.03 mmol, 10 mol%) dry DCE as LAG agent (250 μL, η ≈ 1). Yield determined by ^1^H NMR spectroscopy with dimethyl maleate as internal standard. A) Variation of CO_2_ Source. B) Variation of frequency and heating. C) Effect of liquid‐assisted grinding. D) Comparison with solution‐based approach.

We started our investigation working on the synthesis of the oxazolidinones from propargylamines and CO_2_.^[^ ^32^ ^]^ The oxazolidinone fragment is found in pharmaceutical compounds such as *Linezolid*,^[^ ^33^ ^]^ agrochemicals,^[^ ^34^ ^]^ and Evans auxiliaries.^[^ ^35^ ^]^ Straightforward methodologies to access this valuable scaffold from propargylamines may involve the utilization of metallic catalysts (i.e., Ag, Cu, Pd, Zn, and Au) often in combination with strong bases (amidines and guanidines).^[^ ^36^, ^37^, ^38^, ^39^, ^40^, ^41^, ^42^, ^43^, ^44^ ^]^ In a limited number of examples, oxazolidinones can be obtained via carboxylation using caesium or potassium hydrogencarbonate as CO_2_ surrogates with the use of large amount of organic solvent.^[^ ^6^, ^7^, ^45^, ^46^ ^]^ We initially set out to develop a versatile method to mechanochemically activate NaHCO_3_ for the incorporation of the —CO_2_— moiety into a propargylamine with a minimal amount of solvent and short reaction times. An extensive optimization study (Table S1–S6, Supporting Information) allowed to identify the optimal reaction conditions and the most relevant deviations (**Figure** **2**). The selected catalytic system (AgNO_3_ and guanidine **G**) enabled the almost complete consumption of propargylamine **R1** and NaHCO_3_, delivering the corresponding oxazolidinone **1** in 95% yield at 50 Hz, without heating, in 2 h (Figure 2A, STD, *standard conditions*). The yield of oxazolidinone **1** dropped to 44% using 4 equiv. of NaHCO_3_ while the reaction was completely hindered in the absence of NaHCO_3_ (Figure 2A, E1 and E2).^[^ ^47^ ^]^ On the other hand, keeping 4 equiv. of NaHCO_3_, an excellent 95% yield of the desired cyclic carbamate was again obtained by increasing the reaction time to 4 h (Figure 2A, E3). Alternative bicarbonate sources, such as KHCO_3_ and CsHCO_3_, were less efficient likely because their much higher decomposition temperature if compared with NaHCO_3_ (Figure 2A, E4 and E5).^[^ ^48^, ^49^ ^]^ The same reason can explain why Na_2_CO_3_ in place of NaHCO_3_ was completely ineffective (Figure 2A, E6). The combined effect of frequency and temperature was then elucidated. The milling frequency was found to be crucial for a productive transformation as only 11% of **1** was observed at 30 Hz (Figure 2B, E7). However, recent observations indicate that increasing the temperature during the grinding process, known as “Heat & Beat,” can significantly enhance outcomes.^[^ ^50^ ^]^ In fact, when the temperature was increased to 40 °C, 30 Hz were good enough to obtain 68% yield of **1** (Figure 2B, E8). Pleasantly, we achieved an excellent 93% with external heating at 60 °C (Figure 2B, E9). Regarding the use of liquid‐assisted grinding (LAG) agent, it is evident the beneficial presence of a small amount of solvent, since under neat conditions only 23% was observed (Figure 2C, E10). If 1,2‐dichloroethane (DCE) allowed to achieve the best result (Figure 2C, STD), several greener alternatives can lead to satisfactory yields for carbamate **1** (71%, 68%, and 66%, respectively, for acetonitrile, H_2_O, and acetone, Figure 2C, E11, E13, and E14). Notably, we have shown that acetone can be comparable to DCE by simply extending the reaction time from 2 to 3 h (Figure 2C, E15). Furthermore, by reducing the amount of LAG agent from 250 to 100 μL, the yield of product **1** remains quite acceptable (84%, Figure 2C, E16). Finally, a comparison with a solution‐based approach was carried out. Under our reaction conditions, the mechanochemical approach is essential (Figure 2D, E17–21) as poor results can be observed in the absence of ball milling even at higher temperature (from 60 to 100 °C) and concentrations (up to η ≈ 1). The entire optimization study can be found in Table S1–S6 of the supporting information file, meanwhile, an indication of the environmental impact of the process is provided through the calculation of the corresponding E factor (Section 5, Supporting Information).

### 2.2 Substrate Scope for the Synthesis of Oxazolidinones

**Figure 3:**
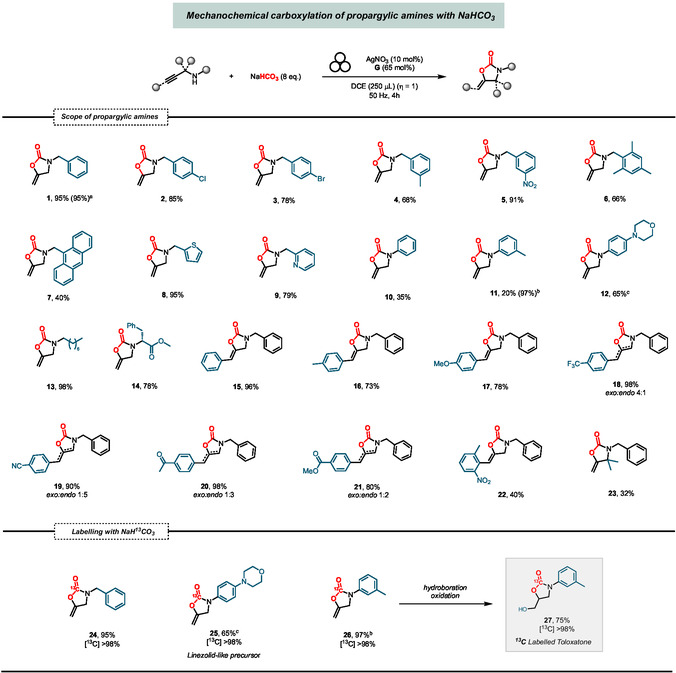
Scope of the mechanochemical carboxylation of propargylic amines with NaHCO_3_: *Reaction conditions*: Stainless‐steel jar of 15 mL, stainless‐steel ball (∅: 15 mm, 13.5 g), 4 h, 50 Hz, propargylic amine (0.3 mmol), **G** (0.195 mmol), NaHCO_3_ (2.4 mmol), dry DCE as LAG agent (250 μL, η ≈ 1). All reported yields are intended after isolation. a) NaHCO_3_ purchased from supermarket (1.5 Eur Kg^−1^). b) Modified conditions: 4 h, 50 Hz, 60 °C, propargylic amine (0.3 mmol), **G** (0.6 mmol), NaHCO_3_ (2.4 mmol), dry DCE as LAG agent (250 μL, η ≈ 1). c) Modified conditions: 4 h, 50 Hz, 60 °C, propargylic amine (0.3 mmol), **G** (0.3 mmol), NaHCO_3_ (2.4 mmol), dry DCE as LAG agent (250 μL, η ≈ 1).

After obtaining suitable reaction conditions, we explored the substrate scope for the Ag/**G**‐catalyzed cyclocarboxylation of propargylic amines using NaHCO_3_ as substitute of gaseous CO_2_ (**Figure** **3**). Electron‐rich and electron‐poor benzyl moieties on the N nucleus gave good to excellent yields of the products, including those with sterically hindered *ortho* positions (**1**‐**6**). Notably, even inexpensive and readily available NaHCO_3_, as directly purchased from a supermarket (1.5 € kg^−1^), proved to be equally effective in the reactions, demonstrating its practicality and accessibility for use in these synthetic transformations. The anthracenyl unit is less compatible (**7**), whereas heteroaromatic rings such as pyridine and thiophene can be incorporated into the final product with high yields (**8**‐**9**). As expected, a less nucleophilic phenyl ring directly attached to the N affords only 35% yield of the corresponding oxazolidinone (**10**).^[^ ^32^ ^]^ Increasing the temperature of the system to 60 °C, along with an improved electron density on the aromatic ring, results in fully satisfactory outcomes (**11**‐**12**). Remarkably, compound **12** is present in the core structure of *Linezolid*. Propargyl amine bearing an alkyl chain gave quantitatively compound **13**. Importantly, product **14** containing the D‐phenylalanine fragment was obtained in 78% yield. Propargyl amines with an internal triple bond were compatible with this transformation, as arenes substituted with both electron‐donating (Me, OMe) and electron‐withdrawing groups (CF_3_, CN, COMe, CO_2_Me, NO_2_) on the phenylacetylene unit were nicely tolerated (**15**‐**22**). In these cases, an *endo* /*exo* mixture of isomers was observed with electron‐withdrawing groups (CF_3_, CN, COMe, CO_2_Me), whereas the *Z*‐isomers of the *exo* product were selectively formed using more electron‐enriched arenes (**15**–**17**). *Endo* isomers, that were successfully separated from their analogues, may derive from the tautomerization of *exo* isomers under basic conditions.^[^ ^51^ ^]^ Steric congestion generated by double substitution at the *ortho* position of the aromatic ring (**22**) or at the propargylic position (**23**) led to lower yields. However, the unreacted starting material can be recovered in these cases. Stable labeled pharmaceutical molecules are essential for accurately tracking and studying drug distribution, metabolism, and elimination in biological systems.^[^ ^52^, ^53^ ^]^ Indeed, in line with the latest strategy to reduce solvent use in the synthesis of bioactive compounds,^[^ ^54^, ^55^, ^56^ ^]^ we attempted the incorporation of ^13^C isotope into bioactive compounds using NaH^13^CO_3_. Our method, allowing to consume a controlled amount of expensive ^13^C isotope‐containing CO_2_ surrogate, provided labeled oxazolidinones **24** and **25** in good to excellent yields. Moreover, labeled compound **26** was obtained with 97% yield and subsequently converted into the corresponding antidepressant *Toloxatone* **27** by hydroboration/oxidation sequence with complete carbon isotope incorporation (see Supporting Information).

### 2.3 The Synthesis of Cyclic Carbonates from NaHCO_3_ and Epoxides

**Figure 4:**
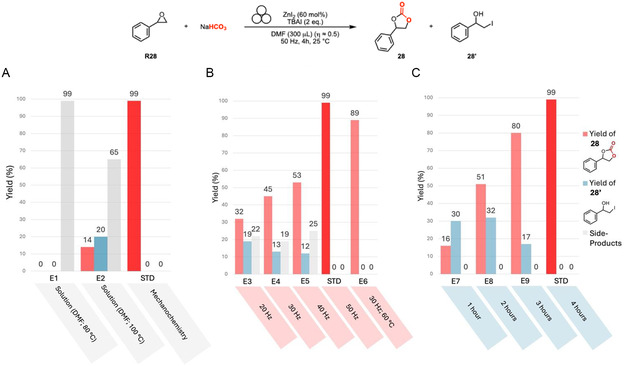
Effect of the most relevant parameters in the mechanochemical carboxylation of styrene oxide **R28**. *Standard conditions*: Stainless‐steel jar of 15 mL, stainless‐steel ball (∅: 15 mm, 13.5 g), 4 h, 50 Hz, **R28** (0.3 mmol, 1 equiv.), ZnI_2_ (0.18 mmol, 60 mol %), TBAI (0.6 mmol, 2 equiv.), NaHCO_3_ (6.0 mmol, 20 equiv.), dry DMF as LAG agent (300 μL, η ≈ 0.5). Yield determined by ^1^H NMR spectroscopy with dimethyl maleate as internal standard. A) Comparison with solution‐based approach. B) Effect of milling frequency. C) Effect of milling time.

From an industrial point of view, the synthesis of cyclic carbonates by carboxylation of epoxides is one of the most relevant CO_2_‐based processes, since cyclic carbonates are used as electrolytes in lithium‐ion batteries, in pharmaceuticals and agrochemicals, and as monomers for polycarbonates.^[^ ^57^ ^]^ In contrast to systems that utilize CO_2_ for synthesizing these products, our method circumvents the direct use of gaseous CO_2_, eliminates the necessity for complex catalytic systems needed for CO_2_ incorporation at room temperature, and facilitates precise dosing to prevent the re‐emission of excess CO_2_.^[^ ^58^, ^59^, ^60^, ^61^ ^]^ We started our research by investigating the most used metal catalysts with the potential to effectively carboxylate styrene oxide. An extensive experimental investigation (Tables S7–14, Supporting Information) led us to conclude that the most efficient activation system relied on utilizing commercially accessible ZnI_2_ and tetrabutylammonium iodide (TBAI) as a source of iodide (**Figure** **4**). In particular, the standard conditions enabled the formation of styrene carbonate (**28**) in almost quantitative yield (Figure 4A, STD). Importantly, the reaction conducted in solution at 80–100 °C without ball milling did not yield the expected product in significant amount (Figure 4, E1 and E2), highlighting the crucial role of mechanochemistry for the activation of NaHCO_3_. It is important to remark that this second type of reactivity, which leads to cyclic carbonates, is an example of a reaction that scarcely proceed under conventional solvent‐based conditions but is effectively enabled by mechanochemistry. The milling frequency is also crucial to achieve a high reaction rate and full selectivity (Figure 4B, E3–5). As observed previously with carbamate **1**, cyclic carbonate **28** can be selectively obtained at lower frequency applying external heating (Figure 4B, E6). A further investigation into the reaction's kinetic profile showed that compound **28’** is an intermediate in this transformation, as it is formed during the first two hours and then disappears by the end of the reaction (Figure 4C, E7–9, see Supporting Information, for independent conversion of **28’** to **28**).^[^ ^62^ ^]^ Byproducts formed at lower frequency could be kinetically favored polymeric materials.^[^ ^63^ ^]^

### 2.4 Substrate Scope for the Synthesis of Cyclic Carbonates

**Figure 5:**
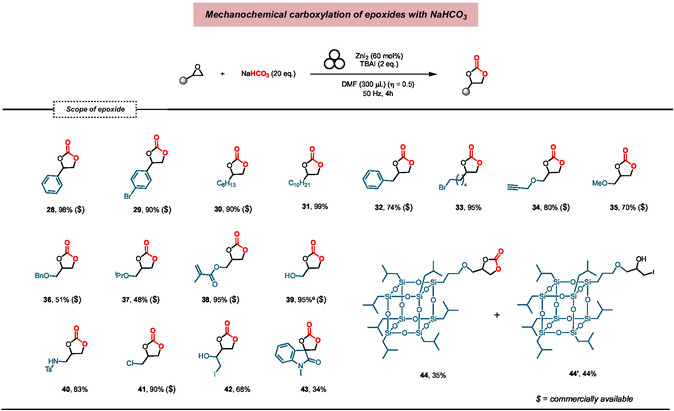
Scope of the mechanochemical carboxylation of epoxides with NaHCO_3_: *Reaction conditions*: Stainless‐steel jar of 15 mL, stainless‐steel ball (∅: 15 mm, 13.5 g), 4 h, 50 Hz, starting epoxide (0.3 mmol), TBAI (0.6 mmol), NaHCO_3_ (6.0 mmol), dry DMF as LAG agent (300 μL, η ≈ 0.5). All reported yields are intended after isolation. ^a^ Yield determined by ^1^H NMR spectroscopy.

With the optimized conditions in hands, a series of terminal epoxides was studied, and the results are summarized in **Figure** **5**. Monosubstituted epoxides were converted into cyclic carbonates under standard conditions in good to excellent yields. Similarly to styrene oxide, 4‐bromostyrene oxide, bearing a useful substituent for versatile derivatization, afforded the corresponding cyclic carbonate **29** in 90% yield. Reactions of alkyl‐substituted epoxides proceeded smoothly and generated cyclic carbonates **30**–**33** in high yields. Apparently, the presence of the benzyl substituent hinders the cycloaddition to a certain extent, while a bromide on the alkyl chain is fully tolerated. Different ether‐substituted epoxides reacted nicely and gave cyclic carbonates **34**–**37** in 48–80% yields. Remarkably, the methacrylate unit, which is commonly used in the production of copolymers, is highly compatible with this transformation (**38**). An epoxide derivative containing a free OH group led to the corresponding product **39** in near quantitative yield. Synthetically useful handles, such as *p*‐toluenesulfonamide (NHTs) and Cl substituents, were also tolerated as compounds **40** and **41** were isolated in 83% and 90% yield, respectively. Symmetric substrate **R42**, displaying two epoxide units, underwent mono cyclocarboxylation on one ring and iodohydrin formation on the other. Although with limited conversion, biologically relevant spirooxindole carbonate **43** was accessed from the corresponding epoxide with high selectivity.^[^ ^64^ ^]^ Finally, a polyhedral oligomeric silsesquioxane (POSS) bearing the epoxide function was successfully converted into the desired cyclic carbonate **44** together with its functionalized precursor **44’**. POSSs are regarded as ideal building blocks for fabricating hybrid materials in biomedical applications.^[^ ^65^ ^]^

### 2.5 Proposed Reaction Pathway

**Figure 6:**
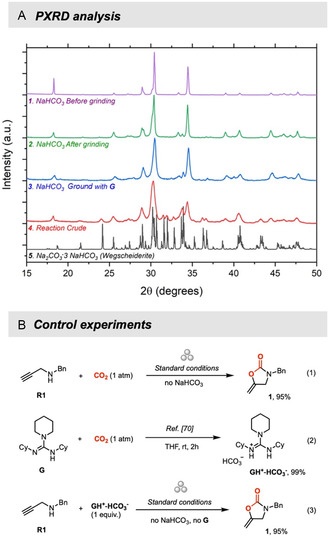
A) PXRD: 1) NaHCO_3_ prior to grinding, 2) NaHCO_3_ after ball milling (50 Hz, 2 h), 3) NaHCO_3_ (2.5 mmol) and guanidine (0.195 mmol) after ball milling (50 Hz, 2 h) 4) residual powders from the reaction crude after standard conditions, and 5) the calculated pattern of Na_2_CO_3_·3NaHCO_3_ (Wegscheiderite). B) Control experiments.

It is well known that NaHCO_3_ starts to slowly release CO_2_ at 80 °C with the concomitant formation of Na_2_CO_3_.^[^ ^66^ ^]^ Nevertheless, substantial decomposition is observed at temperatures exceeding 100 °C (Figure S6, Supporting Information, TGA of NaHCO_3_). To gain a deeper understanding of the presumed mechanochemical activation of NaHCO_3_, we first utilized powder X‐ray diffraction (PXRD) to investigate the characteristics of the solid material produced when NaHCO_3_ is subjected to ball milling. In particular, **Figure** **6**A illustrates the comparison of XRD patterns for 1) NaHCO_3_ prior to grinding (purple line), 2) NaHCO_3_ after ball milling (50 Hz, 2 h, green line), 3) NaHCO_3_ (2.5 mmol) and guanidine (0.195 mmol) after ball milling (50 Hz, 2 h, blue line), and 4) residual powders from the reaction crude after standard conditions (red line). Indeed, when NaHCO_3_ alone is subjected to ball milling (Figure 6A‐2, green line), the majority of the NaHCO_3_ remains unaffected, except for the expected decrease of the crystal size. Importantly, this reduction in crystal size is accompanied by the partial decomposition of NaHCO_3_, resulting in the formation of a small but significant amount of Na_2_CO_3_ in the form of a double salt of Na_2_CO_3_ and NaHCO_3_ called *Wegscheiderite* (see below for comparison with the calculated diffractogram). These results indicate that ball milling action can cause, albeit partially, the release of CO_2_ and H_2_O and consequent formation of Na_2_CO_3_. Moreover, the presence of **G** is found to promote this process (Figure 6A‐3, blue line).^[^ ^67^ ^]^ This is consistent with experimental data that demonstrate the promotion effect of the guanidine (Table S1, entry 1, Supporting Information). This phenomenon is more evident studying the PXRD of the reaction crude after removal of organic compounds (Figure 6A‐4, red line),^[^ ^68^ ^]^ where the new phase Na_2_CO_3_·3NaHCO_3_ (*Wegscheiderite*) can be clearly identified by comparison with its calculated diffractogram (Figure 6A‐5, black line) and indicates the improved decomposition of NaHCO_3_ to Na_2_CO_3_ under standard conditions.^[^ ^69^ ^]^

Secondly, grinding the bicarbonate alone (2.5 mmol) resulted in a weight loss consistent with the release of CO_2_, corresponding to the formation of ≈0.19 mmol of CO_2_ due to partial decomposition of NaHCO_3_ (see Table S17, Section 7, Supporting Information). In contrast, when the bicarbonate (2.5 mmol) was ground together with guanidine (0.195 mmol), the final weight remained unchanged. This is likely because the released CO_2_ was quickly captured by the guanidine to form GH^+^HCO_3_^−^. This final observation aligns with the accelerated CO_2_ capture exhibited by an amine derivative under mechanochemical conditions.^[^ ^21^ ^]^

Third, considering the impact of heating (Figure 2B and 4B), the best results have been achieved either at 50 Hz, when the macroscopic temperature spontaneously increased from 25 °C to ≈65 °C, or at 30 Hz, with the external heating set to 60 °C (Figure S1, Supporting Information).

These preliminary findings support a scenario in which ball‐milling induces the in situ generation of CO_2_ at temperature that are well below the common decomposition temperature of NaHCO_3_ (Figure S6, Supporting Information, TGA of NaHCO_3_). Additionally, these observations highlight the role of mechanical grinding in facilitating the decomposition of NaHCO_3_. The reduction in particle size achieved through grinding significantly enhances the reaction, allowing the required decomposition temperature to drop from above 100 °C to ≈60 °C.^[^ ^66^, ^70^ ^]^

We then demonstrated that gaseous CO_2_ can be successfully incorporated in the oxazolidinone core under standard milling conditions in the absence of NaHCO_3_ (Figure 6B‐1). In addition, a synthesized guanidine‐hydrogencarbonate adduct **GH** ^**+**^ **HCO**_**3**_^**−**^ (Figure 6B‐2),^[^ ^71^ ^]^ which is supposed to be the most favored form of guanidinium‐CO_2_ adduct in the presence of CO_2_ and moisture, was subjected to standard conditions affording the corresponding carboxylated compound in 95% yield (Figure 6B‐3). These results suggest that the guanidinium salt may act as an intermediate in the process.

Collectively, these data provide support for a plausible mechanism that starts with the mild generation of CO_2_ and proceeds with its further catalytic incorporation in organic substrates (propargyl amines) possibly through **GH** ^**+**^ **HCO**_**3**_^**−**^. As further evidence, temperature can promote the reaction likely by boosting NaHCO_3_ decomposition to CO_2_ (Figure 2B, E7–9). In this context, the complete inefficiency of Na_2_CO_3_ (Figure 2A, E6) can be explained by its high decomposition temperature (up to 780 °C for pure Na_2_CO_3_). However, an alternative reaction pathway that involves a solid‐state process without the generation of gaseous CO_2_ cannot be completely ruled out.

## 3 Conclusion

In summary, we have described the use of NaHCO_3_ as cheap and safe solid CO_2_ surrogate for carboxylation reactions under mechanochemical conditions. High value‐added chemicals such as cyclic carbamates and carbonates can be efficiently obtained by ball‐milling NaHCO_3_ with propargylamines and epoxides, respectively. Labeled NaH^13^CO_3_ is equally effective to provide pharmaceutically active labeled molecules such as *Toloxatone* and a *Linezolid‐like* precursor without handling gases and high‐pressured cylinders. Remarkably, in comparison to solvent conditions, reactions that produce carbamates occur at lower temperatures and require shorter reaction times, whereas carbonates can be synthesized from NaHCO_3_ only under mechanochemical onditions. The assessment of the impact of various parameters, such as frequency, temperature, and time, along with control experiments and PXRD analysis, supports the activation of NaHCO_3_ under mechanochemical conditions resulting in the in situ generation of CO_2_ gas under very mild conditions (< 60 °C).

Beyond the synthesis of oxazolidinones and cyclic carbonates from CO_2_, this work lays the foundation for future advancements. We believe that NaHCO_3_ could serve as a viable alternative to CO_2_ in pharmaceutical synthesis and polymer chemistry, thereby enhancing the value of this abundant and renewable C1 carbon source.

## Conflict of Interest

The authors declare no conflict of interest.

## Supporting information

Supplementary Material

## Data Availability

The data that support the findings of this study are available in the supplementary material of this article.
