# Supplementary material for: Mechanochemical Activation of NaHCO3: A Solid CO2 Surrogate in Carboxylation Reactions
Source: ChemSusChem. 2025 Jun 2;18(14):e202500461. doi: 10.1002/cssc.202500461 (PMC12270364; doi:10.1002/cssc.202500461)
Supplement: Supplementary file 1 — Supplementary Material [file CSSC-18-e202500461-s001.pdf]

Supporting Information

Mechanochemical Activation of $NaHCO_3$: A Solid CO_2 Surrogate in Carboxylation Reactions

*Francesco Mele, Andrea Aquilini, Ana Maria Constantin, Francesco Pancrazzi, Lara Righi, Andrea Porcheddu, Raimondo Maggi, Daniele Alessandro Cauzzi, Giovanni Maestri, Elena Motti, Luca Capaldo and Nicola Della Ca'**

Table of Contents

1. GENERAL INFORMATION S2

2. OPTIMIZATION OF REACTION CONDITIONS S3

2.1 Optimization of conditions for reaction of propargylic amines S3
2.2 Optimization of conditions for reaction of epoxides S8

3. CHARTS OF STARTING MATERIALS S13

3.1 Amines S13
3.2 Epoxides S14
3.3 Substrate Limitations S14

4. EXPERIMENTAL PROCEDURES S15

4.1 General procedures for the synthesis of propargylic amines S15
4.2 General procedures for the synthesis of epoxides S16
4.3 General procedure for mechanochemical synthesis of oxazolidinones S18
4.4 General procedure for mechanochemical synthesis of cyclic carbonates S19
4.5 General procedure for mechanochemical labelling of oxazolidinones S19
4.6 Synthesis of ^{13}C labelled Toloxatone (**27**) S20
4.7 Synthesis of (Z)-N,N'-dicyclohexylpiperidine-1-carboximidamide (**G**) S20
4.8 Synthesis of guanidinium bicarbonate **[GH][HCO$_3$]** S20
4.9 Synthesis of intermediate **28’** S21
4.10 Mechanosynthesis of 4-phenyl-1,3-dioxolan-2-one from intermediate **28’** S21

5. E FACTOR CALCULATION S22

6. THERMOGRAVIMETRIC ANALYSIS S23

7. CALCULATIONS ON WEIGHT LOSS DURING MILLING S24

8. CHARACTERIZATION DATA S25

8.1 Propargylic Amines S25
8.2 Epoxides S32
8.3 Oxazolidinones S34
8.4 Cyclic Carbonates S45
8.5 Iodohydrin **28’** and **44’** S51
8.6 Guanidine **G** and **[GH][HCO$_3$]** S52

9. REFERENCES S53

10. AUTHOR CONTRIBUTIONS S56

11. COPY OF NMR SPECTRA S57

1. General Information

All reagents were used as received from commercial sources without further purification. All solvents were dried over activated 4 Å molecular sieves for 24 h. All reactions were conducted as single replicate and were analysed by TLC and by GC using a 30 m SE-30 capillary column. Flash column chromatography was performed on silica gel 60 (70–230 mesh).

NMR spectroscopy. Unless otherwise indicated NMR spectra were recorded on Bruker AVANCE 400 and JEOL 600MHz ECZ600R spectrometers in deuterated chloroform, using the solvent residual signals as internal reference (7.26 and 77.00 ppm, respectively for ^{1}H and ^{13}C), or deuterated dimethyl sulfoxide (2.50 and 39.52 ppm, respectively for ^{1}H and ^{13}C). Chemical shifts (δ) and coupling constants (J) are given in ppm and in Hz, respectively. The acquired NMR spectra were analyzed using MestReNova software version 14.3.3-33362.

Mass spectrometry. HRMS spectra were obtained with LTQ Orbitrap XL Thermo.

Melting point. Melting points were measured with an Electrothermal apparatus and are uncorrected.

X-ray diffraction. X-ray diffraction data were acquired using a Rigaku Smartlab Multipurpose diffractometer equipped with a HyPix3000 two-dimensional detector (Rigaku, Tokyo, Japan) on powder samples. The diffraction patterns were collected under ambient conditions in Bragg–Brentano geometry with CuKα radiation (40 kV and 30 mA) in the 2θ angular range of 10°–60° at a scan rate of 5°/min with a step size of 0.01°. The identification of crystalline phases was performed using QUALX software (Institute of Crystallography – CNR – Bari) and the ICDD (International Centre of Diffraction Data) data base.

Mechanochemical Apparatus. The ball mill grinding experiments were all performed using a Fritsch P23 vertical movement Shaker Mill. This mill has a fixed amplitude of 9 mm and adjustable frequency from 15 Hz to 50 Hz with an adjustable timer.

Temperature control over mechanochemical reaction. Temperature measurements were carried out both inside and outside the vessel using an infrared thermometer, immediately before and after the milling process. For the experiments conducted at high temperature, a heat gun was used to heat the vessel. The external temperature of the vessel was monitored, and milling was initiated once the temperature stabilized at the desired value.

2. Optimization of reaction conditions

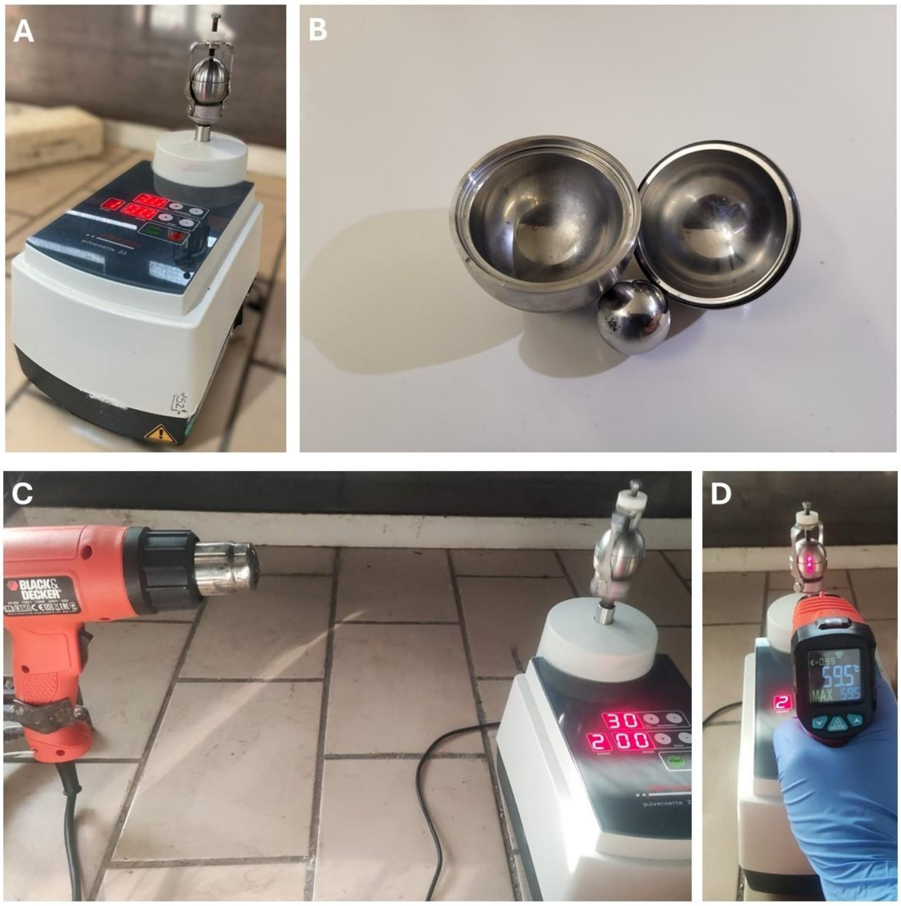

Figure S1. Setup for the mechanochemical reaction: A) Fritsch P23 vibratory ball mill. B) Stainless-steel jar of 15 mL and stainless-steel milling ball (Ø: 15 mm, 13.5 g). C) Experimental setup for high-temperature conditions. D) Monitoring of the external temperature of the vessel before, during, and after heating.

2.1 Optimization of conditions for reaction of propargylic amines

The optimization of the reaction conditions was carried out by studying the reaction of N-benzylprop-2-yn-1-amine (**R1**) and sodium bicarbonate to give 3-benzyl-5-methyleneoxazolidin-2-one (**1**) on a 0.3 mmol scale (see **Table S1-6**).

Propargylic amine (0.2 mmol), superbase (*x* mol%), metal catalyst (*x* mol%), and $NaHCO_3$ (*x* mmol), were placed in a ball milling vessel (stainless steel, 15 mL) loaded with one grinding ball (stainless steel, diameter: 15 mm, 13.5 g). Then the chosen LAG agent (η = 0 or 1) was added via a syringe, the vessel was closed in air, and finally it was placed in the vibratory ball mill (Fritsch Pulverisette P23, 50 Hz). For high-temperature reactions, heating was carried out using a heat gun with adjustable settings. The temperature was externally monitored using an infrared thermometer, and milling was started once the temperature stabilized at the desired value. After 2h, the jar was opened, the mixture was recovered with 10 mL of EtOAc and filtered through a short silica plug to remove solids. The solvent was removed, and the sample was analyzed via ^{1}H NMR spectroscopy ($CDCl_3$, dimethyl maleate as internal standard).

2.1.1 Screening of superbases

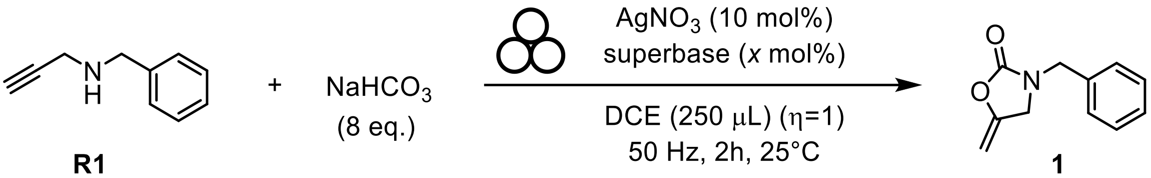

R1 **1**

Entry	Superbase	Yield [a]
**1**	-	15%
**2**	G (10 mol%)	20%
**3**	G (25 mol%)	25%
**4**	G (50 mol%)	59%
**5**	**G (65 mol%)**	**95% (95%)[b]**
**6**	TBD (65 mol%)	74%
**7**	MTBD (65 mol%)	81%
**8**	DBU (65 mol%)	76%

Table S1. Screening of superbases. [a] Yields determined by ^{1}H NMR spectroscopy, dimethyl maleate as internal standard. [b] Yields after purification.

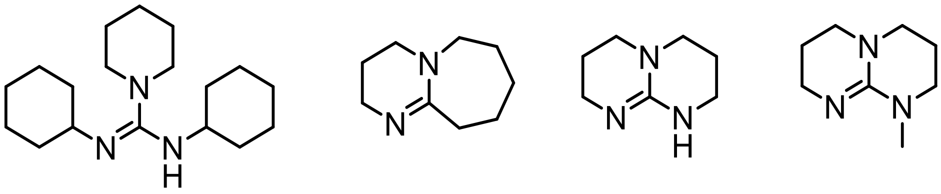

G **DBU** **TBD** **MTBD**

Figure S2. Structure of superbases tested during the optimization of reaction conditions.

2.1.2 Screening of metal catalysts

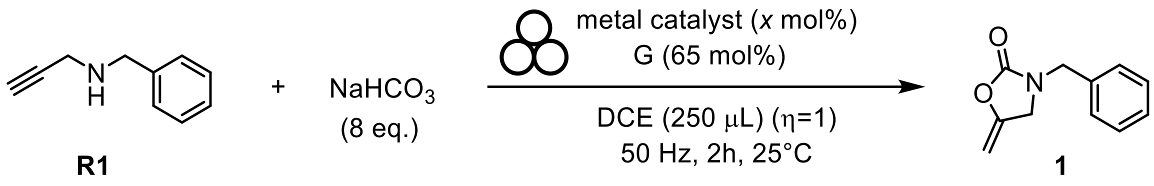

Entry	Catalyst	Yield [a]
**1**	-	10%
**2** [b]	-	-
**3**	$AgNO_3$ (5 mol%)	70%
**4**	Ag_2O (5 mol%)	73%
**5**	**$AgNO_3$ (10 mol%)**	**95% (95%)[c]**
**6**	Ag_2O (10 mol%)	77%
**7**	Cu_2O (10 mol%)	-
**8**	CuBr (10 mol%)	36%
**9**	$AgSbF_6$ (10 mol%)	73%

Table S2. Screening of metal catalysts. [a] Yields determined by ^{1}H NMR spectroscopy, dimethyl maleate as internal standard. [b] Without silver and superbase **G**. [c] Yields after purification.

2.1.3 Screening of LAG

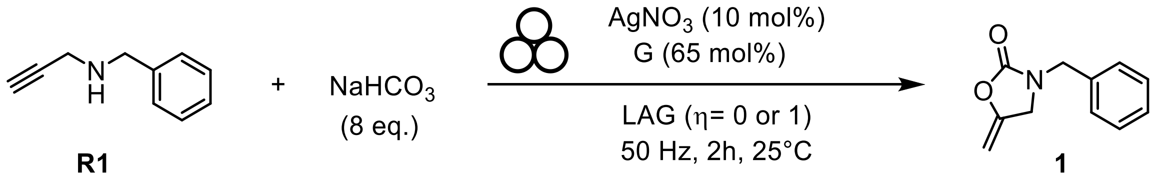

Entry	Ref MainText	LAG	Yield [a]
**1**	(Fig. 2, E10)	-	23%
**2**	(Fig. 2, E11)	ACN dry (250 µL)	71%
**3**	(Fig. 2, E12)	EtOAc (250 µL)	12%
**4**	(Fig. 2, E13)	H_2O (250 µL)	66%
**5**	(Fig. 2, E14)	Acetone (250 µL)	68%
**6**	**(Fig. 2, STD)**	**DCE dry (250 µL)**	**95% (95%)[b]**
**7**	(Fig. 2, E15)	Acetone (250 µL)	86% [c]
**8**	(Fig. 2, E16)	DCE dry (100 µL)	84%

Table S3. Screening of LAG. [a] Yields determined by ^{1}H NMR spectroscopy, dimethyl maleate as internal standard. [b] Yields after purification. [c] Yield calculated by ^{1}H NMR spectroscopy after 3 hours.

2.1.4 Screening of C1 source

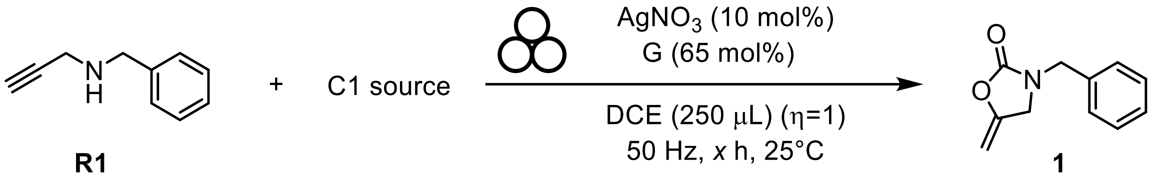

R1 **1**

Entry	Ref MainText	C1 Source	Milling frequency	Milling time	Yield [a]
**1**	**(Fig. 2, STD)**	**$NaHCO_3$ (8.0 eq.)**	**50 Hz**	**2h**	**95% (95%)[b]**
**2**	(Fig. 2, E6)	Na_2CO_3 (8.0 eq.)	50 Hz	2h	2%
**3**	(Fig. 2, E2)	CO_2 (air)	50 Hz	2h	2%
**4**	-	CO_2 (1 atm)	50 Hz	2h	94%
**5**	(Fig. 2, E1)	$NaHCO_3$ (4.0 eq.)	50 Hz	2h	44%
**6**	(Fig. 2, E3)	$NaHCO_3$ (4.0 eq.)	50 Hz	4h	95%
**7**	(Fig. 2, E4)	$KHCO_3$ (8.0 eq.)	50 Hz	2h	23%
**8**	(Fig. 2, E5)	$CsHCO_3$ (8.0 eq.)	50 Hz	2h	26%

Table S4. Screening of C1 source. [a] Yields determined by ^1H NMR spectroscopy, dimethyl maleate as internal standard. [b] Yields after purification.

2.1.5 Screening of milling parameters

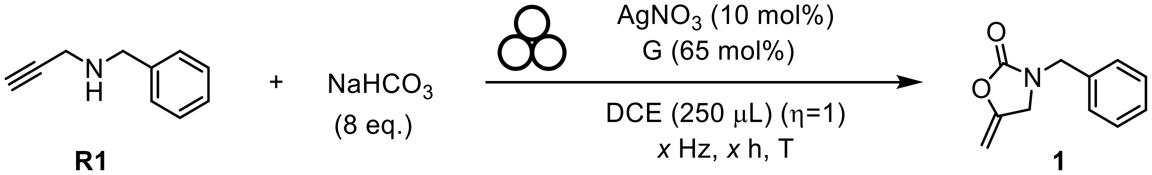

R1 **1**

Entry	Ref MainText	Milling frequency	Milling time	Temperature	Yield [a]
**1**	**(Fig. 2, STD)**	**50 Hz**	**2h**	**25 °C**	**95% (95%)[b]**
**2**	-	20 Hz	2h	40 °C	58%
**3**	-	30 Hz	2h	15 °C	-
**4**	(Fig. 2, E7)	30 Hz	2h	25 °C	11%
**5**	(Fig. 2, E8)	30 Hz	2h	40 °C	68%
**6**	(Fig. 2, E9)	30 Hz	2h	60 °C	93%

Table S5. Screening of milling parameters. [a] Yields determined by ^1H NMR spectroscopy, dimethyl maleate as internal standard. [b] Yields after purification.

2.1.6 Comparison with solution-based approach

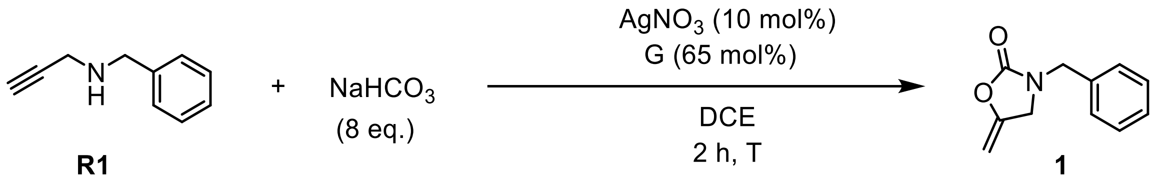

Entry	Ref MainText	Solvent	Temperature	Yield [a]
**1**	(Fig. 2, E17)	DCE (1.5 mL)	60 °C	9%
**2**	(Fig. 2, E18)	DCE (250 µL)	80 °C	47%
**3**	(Fig. 2, E19)	DCE (1.5 mL)	80 °C	9%
**4**	(Fig. 2, E20)	DCE (250 µL)	100 °C	56%
**5**	(Fig. 2, E21)	DCE (1.5 mL)	100 °C	27%
**6 [b]**	**(Fig. 2, STD)**	**DCE (250 µL)**	**25 °C**	**95% (95%)[c]**

Table S6. Model reaction tested in solution. [a] Yields determined by ^{1}H NMR spectroscopy, dimethyl maleate as internal standard. [b] Reaction under standard condition through ball milling. [c] Yields after purification.

2.2 Optimization of conditions for reaction of epoxides

The optimization of the reaction conditions was carried out by studying the reaction of styrene oxide (**R28**) and sodium bicarbonate to give 4-phenyl-1,3-dioxolan-2-one (**28**) on a 0.3 mmol scale (see **Table S7-14**).

Epoxide (0.30 mmol), iodide source (*x* eq.), metal catalyst (*x* mol%), and $NaHCO_3$ (*x* eq.) were placed in a ball milling vessel (stainless steel, 15 mL) loaded with one grinding ball (stainless steel, diameter: 15 mm, 13.5 g). Then the LAG agent (η = 0 or 0.5) was added via a syringe, the vessel was closed in air, and finally it was placed in the vibratory ball mill (Fritsch Pulverisette P23, *x* Hz). After *x* h, the jar was opened, and the mixture was recovered with 10 mL of EtOAc and filtered through a short silica plug to remove solids. The solvent was removed, and the sample was analyzed via ^1H NMR spectroscopy ($CDCl_3$, dimethyl maleate as internal standard).

2.2.1 Screening of metal catalysts

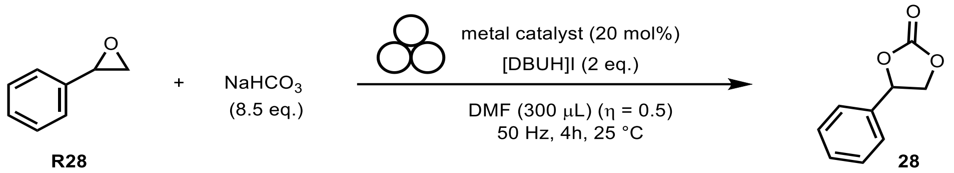

Entry	Metal catalyst (20 mol%)	Yield [a]
1	$AgNO_3$	3%
**2**	**ZnI_2**	**30%**
3	-	3%
4	$Mg(ClO_4)_2$	20%
5	$[TBA]_2ZnI_4$	16%
6	FeI_2	9%

Table S7. Screening of metal catalyst. [a] Yields determined by ^1H NMR spectroscopy, dimethyl maleate as internal standard.

2.2.2 Screening of LAG

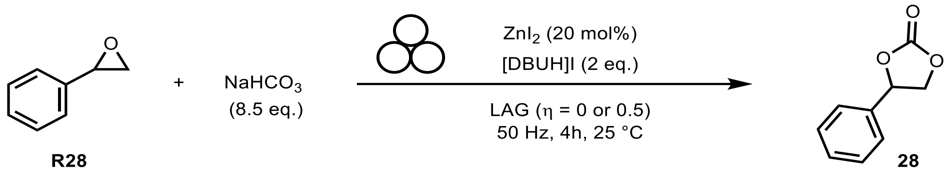

Entry	LAG	Yield [a]
**1**	DCE (300 µL)	6%
**2**	Neat	-
**3**	**DMF (300 µL)**	**30%**

Table S8. Screening of LAG. [a] Yields determined by ^1H NMR spectroscopy, dimethyl maleate as internal standard.

2.2.3 Screening of sodium bicarbonate loading

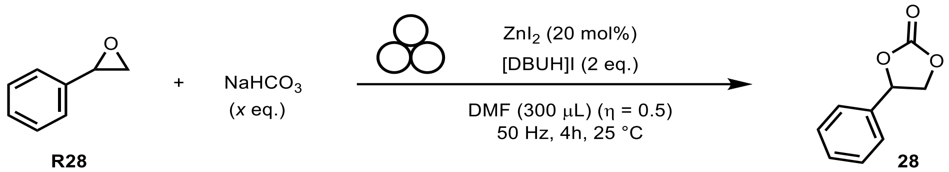

Entry	$NaHCO_3$	Yield [a]
**1**	8.5 eq.	30%
**2**	**20 eq.**	**49%**

Table S9. Screening of sodium bicarbonate loading. [a] Yields determined by ^1H NMR spectroscopy, dimethyl maleate as internal standard.

2.2.4 Screening of zinc iodide loading

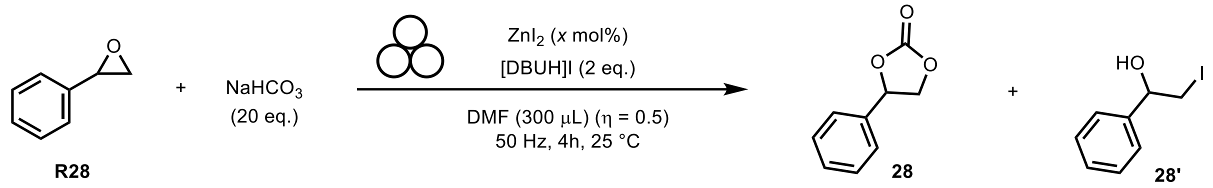

Entry	ZnI_2 (*x* mol%)	Conversion [a]	Yield of 28 [b]	Yield of 28' [b]
1	35%	87%	54%	29%
2	50%	98%	60%	11%
**3**	**60%**	**99%**	**79%**	**21%**

Table S10. Screening of zinc iodide loading. [a] Conversion of **R28** determined by ^{1}H NMR spectroscopy. [b] Yields determined by ^{1}H NMR spectroscopy, dimethyl maleate as internal standard.

2.2.5 Screening of iodide source

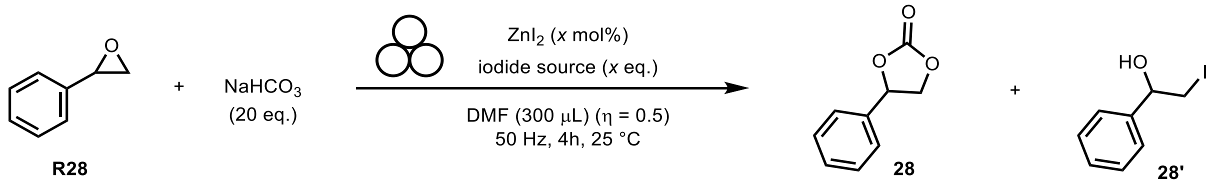

Entry	Iodide source	ZnI_2 (*x* mol%)	Conversion [a]	Yield of 28 [b]	Yield of 28' [b]
1	[DBUH]I (2 eq.)	60%	99%	79%	21%
2	[DBUH]I (50 mol%)	60%	98%	60%	11%
3	-	-	-	-	-
**4**	**TBAI (2 eq.)**	**60%**	**99%**	**99%**	**-**
5	-	100%	99%	75%	25%
6	TBAI (2 eq.)	30%	95%	70%	22%
7	TBAI (50 mol%)	60%	95%	73%	22%

Table S11. Screening of iodide source. [a] Conversion of **R28** determined by ^{1}H NMR spectroscopy. [b] Yields determined by ^{1}H NMR spectroscopy, dimethyl maleate as internal standard.

2.2.6 Screening of milling time

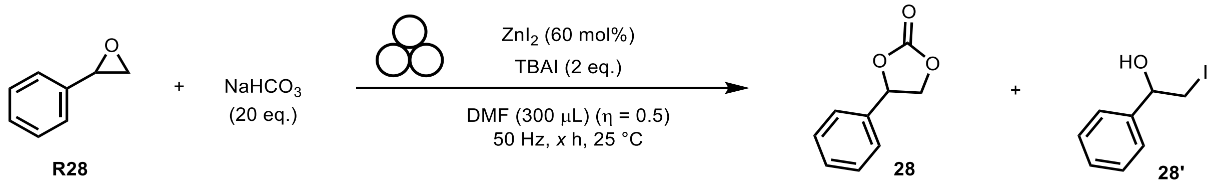

Entry	Ref MainText	Time (h)	Conversion [a]	Yield of 28 [b]	Yield of 28' [b]
**1**	(Fig. 4, E7)	1	50%	16%	30%
**2**	(Fig. 4, E8)	2	85%	51%	32%
**3**	(Fig. 4, E9)	3	97%	80%	17%
**4**	**(Fig. 4, STD)**	**4**	**99%**	**99%**	**-**

Table S12. Screening of milling time. [a] Conversion of **R28** determined by ^1H NMR spectroscopy. [b] Yields determined by ^1H NMR spectroscopy, dimethyl maleate as internal standard.

2.2.7 Screening of milling frequency

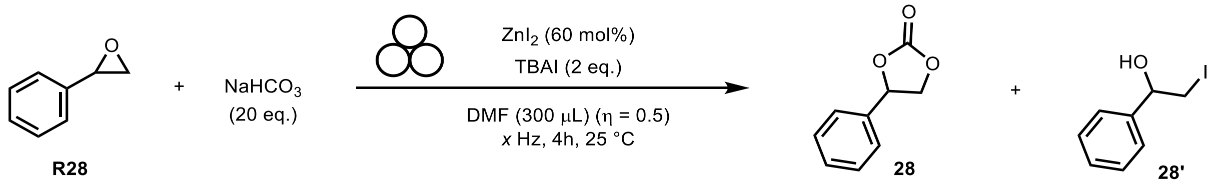

Entry	Ref MainText	Frequency	Conversion [a]	Yield of 28 [b]	Yield of 28' [b]
**1**	(Fig. 4, E3)	20	73%	32%	19%
**2**	(Fig. 4, E4)	30	77%	45%	13%
**3**	(Fig. 4, E5)	40	90%	53%	12%
**4**	**(Fig. 4, STD)**	**50**	**99%**	**99%**	**-**
**5** [c]	(Fig. 4, E6)	30	99%	89%	-

Table S13. Screening of milling frequency. [a] Conversion of **R28** determined by ^1H NMR spectroscopy. [b] Yields determined by ^1H NMR spectroscopy, dimethyl maleate as internal standard. [c] Milling at 30 Hz with external heating at 60 °C.

2.2.8 Comparison with solution-based approach

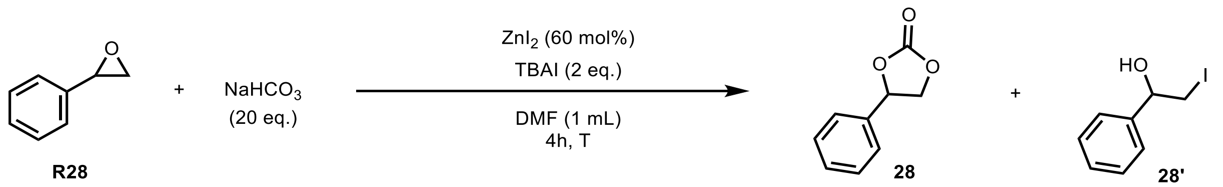

Entry	Ref MainText	Solvent	Temperature	Conversion [a]	Yield of 28 [b]	Yield of 28' [b]
**1**	(Fig. 4, E1)	DMF (1mL)	80 °C	99%	-	-
**2**	(Fig. 4, E2)	DMF (1mL)	100 °C	99%	14%	20%
**3 [c]**	**(Fig. 4, STD)**	**DMF (300 µL)**	**25 °C**	**99%**	**99%**	**-**

Table S14. Model reaction tested in solution. [a] Conversion of **R28** determined by ^1H NMR spectroscopy. [b] Yields determined by ^1H NMR spectroscopy, dimethyl maleate as internal standard. [c] Reaction under standard condition through ball milling.

3. Charts of starting materials

3.1 Amines

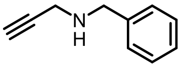

R1

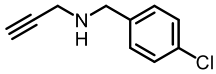

R2

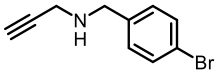

R3

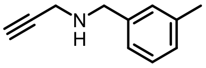

R4

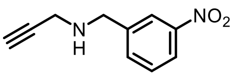

R5

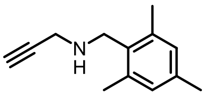

R6

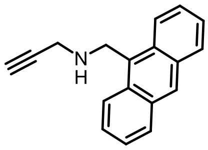

R7

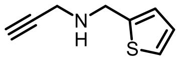

R8

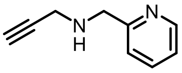

R9

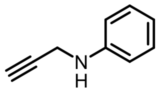

R10

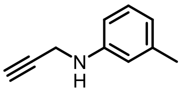

R11

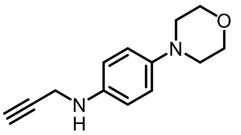

R12

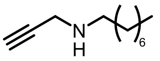

R13

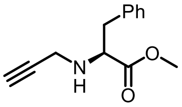

R14

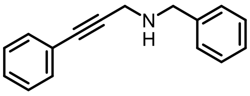

R15

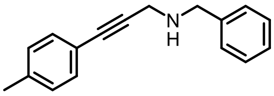

R16

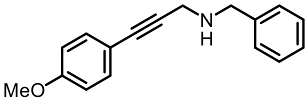

R17

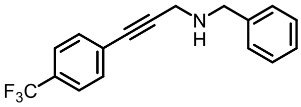

R18

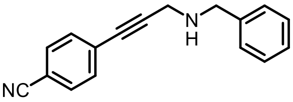

R19

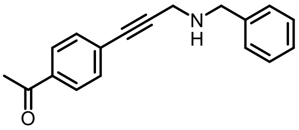

R20

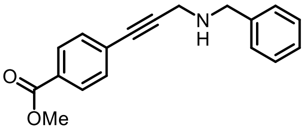

R21

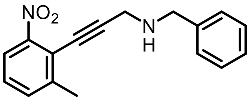

R22

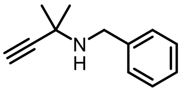

R23

Figure S3. Propargylic amines used as starting materials for mechanochemical carboxylation.

3.2 Epoxides

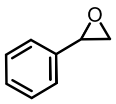
R28

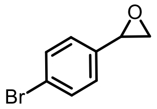
R29

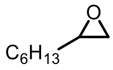
R30

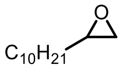
R31

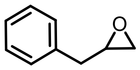
R32

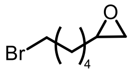
R33

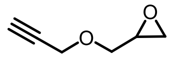
R34

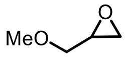
R35

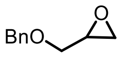
R36

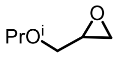
R37

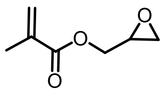
R38

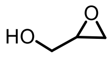
R39

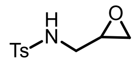
R40

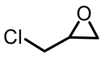
R41

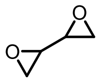
R42

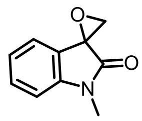
R43

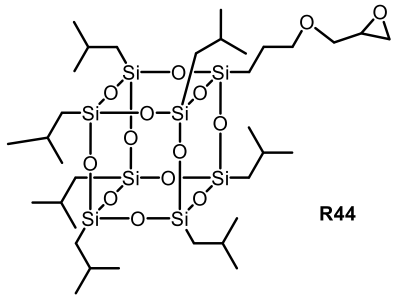
R44

Figure S4. Epoxides used as starting materials for mechanochemical carboxylation.

3.3 Substrate Limitations

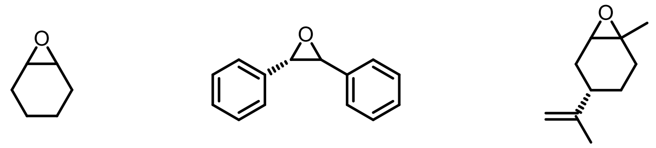
Unreactive substrates

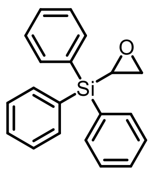
Converted to iodohydrine

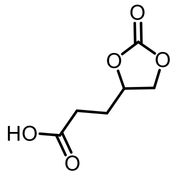
NMR Yield, 10%
Not Isolable

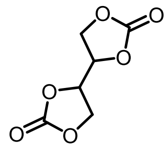
Not Detected

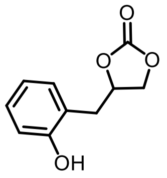
NMR Yield, 62%
Not Isolable

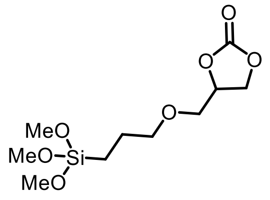
NMR Yield, 65%
Not Isolable

Figure S5. Substrates identified as a limitation for mechanochemical carboxylation.

4. Experimental Procedures

4.1 General procedures for the synthesis of propargylic amines

The propargyl amines were synthesized using three different methods according to literature procedures.

Procedure A: Synthesis from propargyl bromide

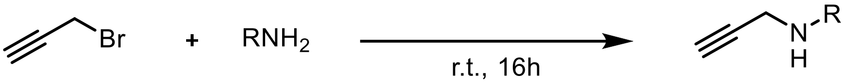

Following the literature procedure [1], the propargylic bromide (1 equiv.) was added dropwise to the corresponding neat amine (6 equiv.) at 0 °C, under nitrogen atmosphere. The mixture was allowed to warm to room temperature and stirred overnight. Then, a mixture of a solution 1 M of NaOH (4 mL/mmol) and Et_2O (4 mL/mmol) was added and stirred for 15 min. The mixture was transferred to a separating funnel, and it was extracted with Et_2O (3x). The combined organic phases were dried over anhydrous Na_2SO_4, filtered, and concentrated under reduced pressure. The crude was then purified by flash column chromatography (eluent Hexane:EtoAc).

Procedure B: Reductive amination

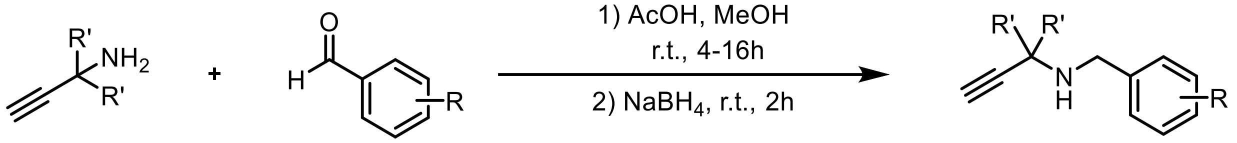

According to the literature procedure [2], four drops of AcOH was added to a solution of propargyl amine (1 equiv.) and aryl aldehyde (1.1 eq.) in MeOH (0.6 M). The resulting mixture was then stirred at room temperature and was monitored with TLC until propargyl imine was formed. $NaBH_4$ (1.5 equiv.) was added in three different portions at 0 °C and the solution was then stirred for 1 h prior to the evaporation of the solvent. The mixture was diluted with water, extracted with DCM (2 times), and the combined organic layers were then washed with a 1 M HCl solution. Aqueous layers were neutralized, extracted with DCM (2 times), and the resulting organic phase was washed with brine, dried over Na_2SO_4, concentrated under reduced pressure, and finally purified by flash column chromatography (eluent Hexane/EtOAc gradient).

Procedure C: Sonogashira's coupling

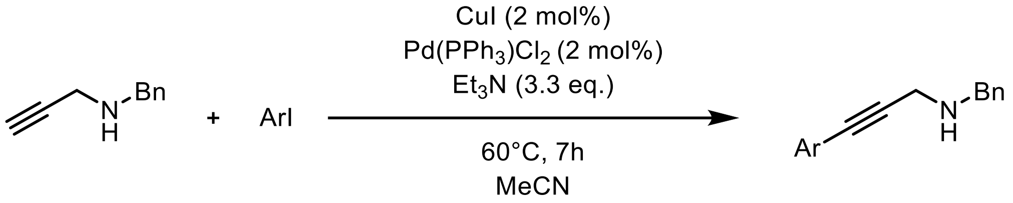

According to the literature procedure[3], in a Schlenk tube under nitrogen atmosphere, $Pd(PPh_3)_2Cl_2$ (2 mol%), CuI (2 mol%), Et_3N (3.3 equiv.) and dry MeCN (0.1 M) were added. Then, the iodoarene (1.1 equiv.) was added and the mixture was heated to 60 °C and stirred for 5 minutes. Benzyl propargyl amine (**R1**) (1.0 equiv.) was added and the reaction mixture was stirred for 7 hours at 60 °C. Then, the reaction mixture was cooled down to ambient temperature and concentrated in vacuo. The resulting crude was dissolved in EtOAc (10 mL), then washed with water (10 mL) and brine (10 mL). The organic layer was dried over Na_2SO_4, filtered, concentrated under reduced pressure and finally purified by flash column chromatography (eluent Hexane/EtOAc gradient).

4.2 General procedures for the synthesis of epoxides

The epoxides were synthesized using four different methods according to literature procedures.

Procedure E: Epoxidation of alkenes

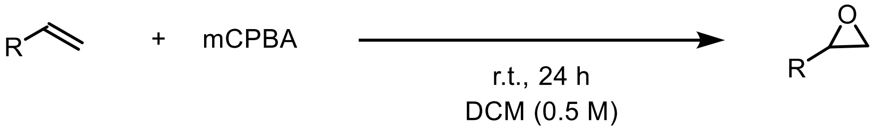

Following the literature procedure[4], to an ice cooled stirred solution of the alkene (1 eq.) in dichloromethane, 3-chloroperbenzoic acid (mCPBA) (1.8 eq.) was added portion-wise in 5 minutes. Then, the solution was stirred in the dark at room temperature for 24h. After the competition of the reaction, the crude mixture was washed with 10% aq. $Na_2S_2O_3$ (10 mL) and extracted with DCM (3x10 mL). The combined organic layers were dried over Na_2SO_4, filtered, concentrated under reduced pressure, and finally purified by flash column chromatography (eluent Hexane/EtOAc gradient).

Procedure F: Johnson−Corey−Chaykovsky epoxidation of benzaldehydes.

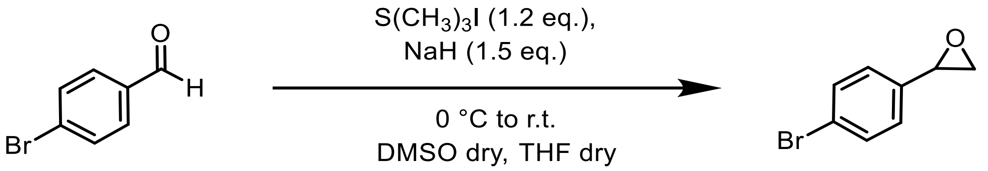

According to the literature procedure [5], sodium hydride (1.5 eq.) was washed with petroleum ether. The residual petroleum ether was removed under vacuum. In a Schlenk tube under nitrogen atmosphere, dry NaH was suspended in dry THF (0.5 M) and the reaction mixture was cooled in an ice bath. A solution of trimethylsulfonium iodide (1.2 eq.) in DMSO (1 M) was added and the mixture was stirred for 5 minutes. After that, 4-bromobenzaldehyde (1 eq.) was added in one portion. The reaction mixture was stirred at 0 °C for 30 min and at room temperature for an additional 16 h. Then, the reaction mixture was slowly quenched with a mixture of water and ice (15 mL) and extracted with EtOAc (3 × 10 mL). The combined organic layers were washed with brine (2 × 30 mL), dried over Na_2SO_4, and filtered. The reaction mixture was purified by flash column chromatography (eluent Hexane/EtOAc gradient) to yield 2-(4-bromophenyl)oxirane (79% yield).

Procedure G: Synthesis of spiro-epoxyoxindole.

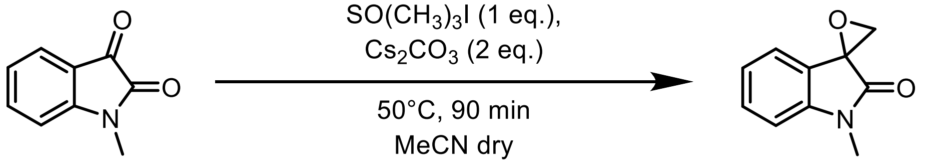

According to the literature procedure [6], trimethylsulfoxonium iodide (1 eq.) and caesium carbonate (2 eq.) was taken in round bottom flask with dry MeCN and stirred at 50 °C under inert atmosphere for 1h. Next, a solution of N-methyl isatin (1eq.) in 10 mL of dry CH_3CN was added dropwise over a period of 10 minutes. The progress of the reaction was monitored by thin-layer chromatography (TLC). Once the reaction was complete, the reaction mixture was filtered through a Celite bed, and the filtrate was then evaporated to concentrate the product. The reaction mixture was purified by flash column chromatography (eluent Hexane/EtOAc gradient) to yield 1-methylspiro[indoline-3,2'-oxiran]-2-one (85% yield).

Procedure H: Synthesis from epichlorohydrin.

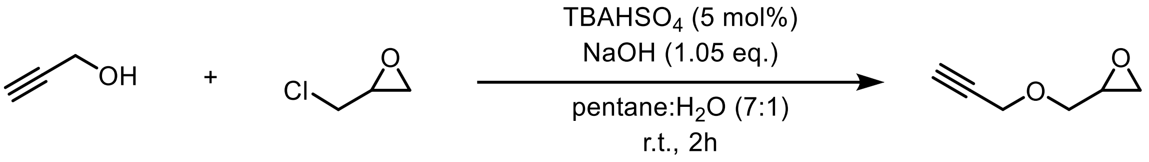

According to the literature procedure [7], propargyl alcohol (1 eq.) was added to the stirring NaOH solution (1.05 eq., 40% in water) at 0° C. The reaction mixture was allowed to stir for 30 min before a solution containing tetrabutylammonium hydrogensulfate ($TBAHSO_4$, 5 mol%), pentanes, H_2O and epichlorohydrin (2 eq.) was added. The reaction was allowed to proceed for 2 hr, then was slowly quenched with brine (10 mL) and extracted with Et_2O (3 × 8 mL). The organic layers were combined, dried over Na_2SO_4, and concentrated under vacuum. The reaction mixture was finally purified by flash column chromatography (eluent Hexane/EtOAc gradient) to yield 2-((prop-2-yn-1-yloxy)methyl)oxirane (45% yield).

4.3 General procedure for mechanochemical synthesis of oxazolidinones

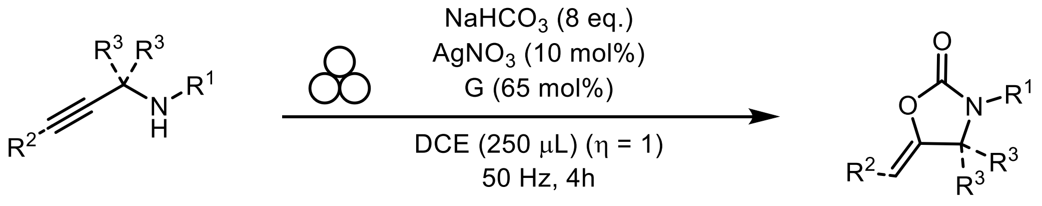

Propargylic amine (0.30 mmol), (Z)-N,N'-dicyclohexylpiperidine-1-carboximidamide (**G**, 0.195 mmol, 0.65 eq., $AgNO_3$ (0.03 mmol, 10 mol%), and $NaHCO_3$ (2.4 mmol, 8 eq.) were placed in a ball milling vessel (stainless steel, 15 mL) loaded with one grinding ball (stainless steel, diameter: 15 mm, 13.5 g). Then DCE (250 µL, η = 1) was added via a syringe. After the vessel was closed in air, it was placed in the vibratory ball mill (Fritsch Pulverisette P23, 50 Hz). For reactions conducted at high temperature, a heat gun with a temperature control function is used. After 4h, the jar was opened, and the mixture was recovered with 10 mL of EtOAc or DCM. The crude mixture was directly purified by flash column chromatography (eluent Hexane/EtOAc gradient) to give the corresponding oxazolidinone.

4.4 General procedure for mechanochemical synthesis of cyclic carbonates

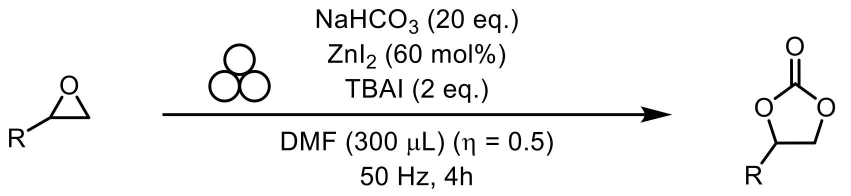

Epoxide (0.30 mmol), TBAI (0.60 mmol, 2.0 eq.), ZnI_2 (0.18 mmol, 60 mol%), and $NaHCO_3$ (6.0 mmol, 20 eq.) were placed in a ball milling vessel (stainless steel, 15 mL) loaded with one grinding ball (stainless steel , diameter: 15 mm, 13.5 g). Then DMF (300 μL, η = 0.5) was added via a syringe. After the vessel was closed in air, it was placed in the vibratory ball mill (Fritsch Pulverisette P23, 50 Hz). After 4h, the jar was opened, and the mixture was recovered with 10 mL of EtOAc. The crude mixture was washed with water and extracted with EtOAc (3 × 10 mL). The organic layers were combined, dried over Na_2SO_4, and concentrated under vacuum. The reaction mixture was finally purified by flash column chromatography (eluent Hexane/EtOAc gradient) to give the corresponding carbonate.

4.5 General procedure for mechanochemical labelling of oxazolidinones

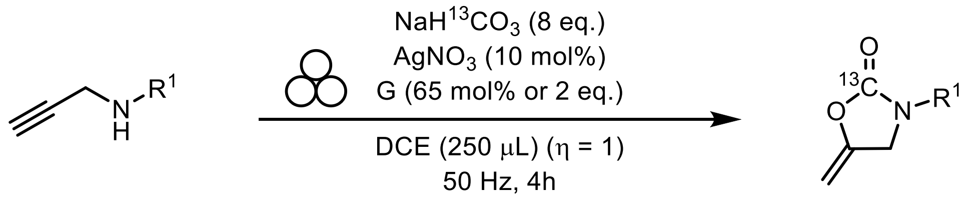

Propargylic amine (0.30 mmol), (Z)-N,N'-dicyclohexylpiperidine-1-carboximidamide (**G,** 0.195 mmol, 0.65, $AgNO_3$ (0.03 mmol, 10 mol%), and $NaH^{13}CO_3$ (98 atom % 13C, 2.4 mmol, 8 eq.) were placed in a ball milling vessel (stainless steel, 15 mL) loaded with one grinding ball (stainless steel, diameter: 15 mm, 13.5 g). Then DCE (250 μL, η = 1) was added via a syringe. After the vessel was closed in air, it was placed in the vibratory ball mill (Fritsch Pulverisette P23, 50 Hz). For reactions conducted at high temperature, a heat gun with a temperature control function is used. After 4h, the jar was opened, and the mixture was recovered with 10 mL of EtOAc or DCM. The crude mixture was directly purified by flash column chromatography (eluent Hexane/EtOAc gradient) to give the corresponding labelled oxazolidinone.

4.6 Synthesis of ^{13}C labelled Toloxatone (**27**)

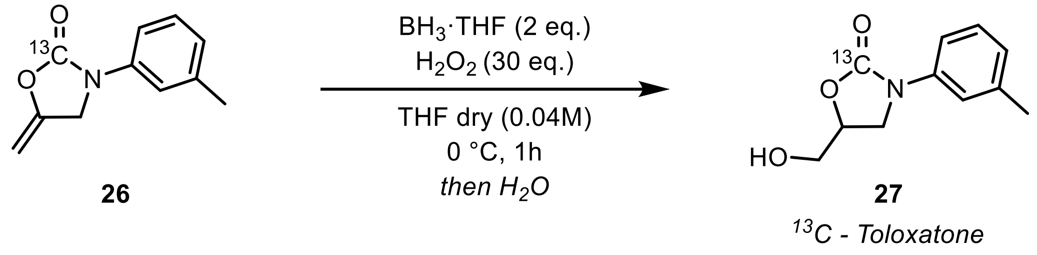

Synthesis of Toloxatone follows an unoptimized procedure. In a Schlenk tube under nitrogen atmosphere, 3-benzyl-5-methyleneoxazolidin-2-one-2-^{13}C (**24**, 0.15 mmol) was dissolved in THF dry (0.04 M) and cooled to 0°C. Then, $BH_3 \cdot THF$ (2 eq.) was slowly added into the mixture in 5 minutes. The reaction mixture was stirred at 0 °C for 30 minutes. After that, H_2O_2 (15% (w/w) in H_2O, 30 eq.) was added dropwise in 15 minutes and the mixture was keep stirred 1h at 0 °C. Upon completion as indicated by TLC, the reaction mixture was slowly quenched with water and extracted with EtOAc (3 × 10 mL). The combined organic layers were dried over Na_2SO_4, and filtered. The reaction mixture was purified by flash column chromatography (eluent Hexane/EtOAc gradient) to yield 5-(hydroxymethyl)-3-(m-tolyl)oxazolidin-2-one-2-13C (75% yield).

4.7 Synthesis of (Z)-N,N'-dicyclohexylpiperidine-1-carboximidamide (**G**)

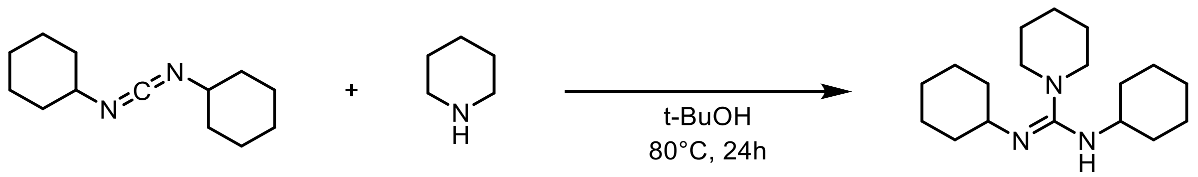

(Z)-N,N'-dicyclohexylpiperidine-1-carboximidamide (**G**) was prepared according to a literature procedure [8] In a Schlenk tube under nitrogen atmosphere, 1,3-dicyclohexylcarbodiimide (1 eq.) and piperidine (2 eq.) were dissolved in dry tert-butyl alcohol (0.5 M). The resulting mixture was stirred 80 °C for 24 h. After that, the solvent and the unconverted piperidine were removed under reduced pressure, thus obtaining pure guanidine **G** (74% yield) as a pale-yellow solid.

4.8 Synthesis of guanidinium bicarbonate **[GH][HCO₃]**

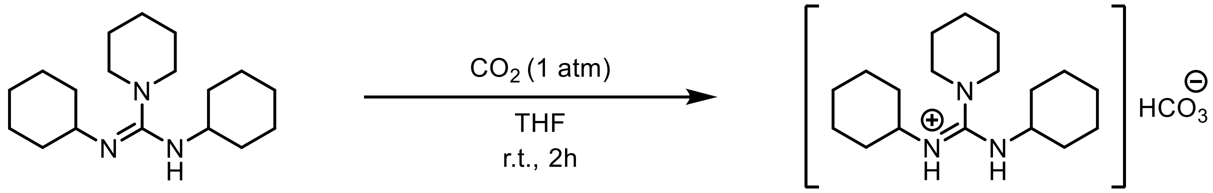

Following a literature procedure for an analogue guanidine [10], a flask containing **G** wet THF was filled with CO_2 (1 atm) and kept under stirring for 2h at room temperature. After that, the solvent was discarded by decantation, and the white powder of the product was dried under vacuum, giving **[GH][HCO₃]** in quantitative yield.

4.9 Synthesis of intermediate **28’**

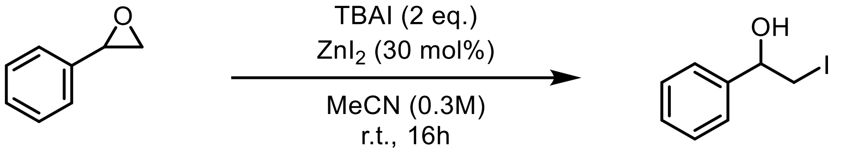

28'

Styrene oxide (1 eq.), TBAI (2 eq.) and ZnI_2 (30 mol%) was dissolved in MeCN (0.3 M). The reaction mixture was stirred at room temperature overnight. Upon completion as indicated by TLC, the mixture was concentrated in vacuo, and directly purified by flash column chromatography (eluent Hexane/EtOAc 8:1) to yield 2-iodo-1-phenylethan-1-ol (49% yield) as an orange oil.

4.10 Mechanosynthesis of 4-phenyl-1,3-dioxolan-2-one from intermediate **28’**

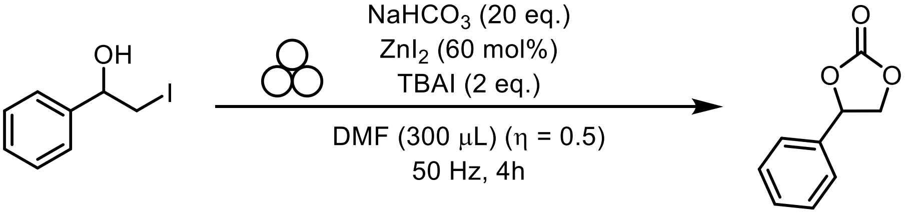

28' **28**

The intermediate 2-iodo-1-phenylethan-1-ol (**28’**) (0.30 mmol), TBAI (2 eq.), ZnI_2 (60 mol%), and $NaHCO_3$ (20 eq.) were placed in a ball milling vessel (stainless steel, 15 mL) loaded with one grinding ball (stainless steel, diameter: 15 mm, 13.5 g). Then DMF (η = 0.5) was added via a syringe. After the vessel was closed in air, it was placed in the vibratory ball mill (Fritsch Pulverisette P23, 50 Hz). After 4 h, the jar was opened, and the mixture was recovered with 10 mL of EtOAc and filtered through a short silica plug to remove solids. The solvent was removed, and the sample was analyzed via ^{1}H NMR spectroscopy ($CDCl_3$, dimethyl maleate as internal standard). Product **28** was obtained in 60% yield.

5. E factor calculation

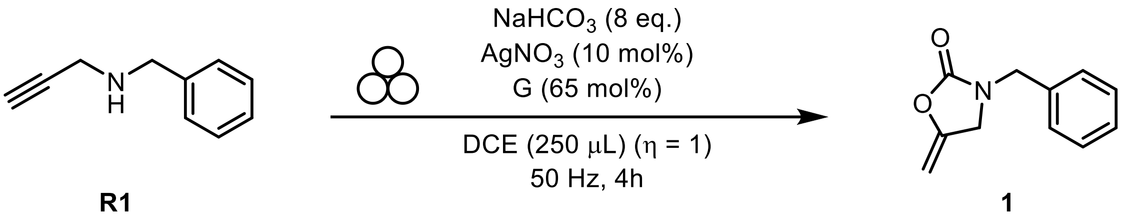

R1 1

Substance	PM	n (mmol)	weight (mg)
Substrate **R1**	145.1	0.3	43.5
$NaHCO_3$	84.0	2.4	201.6
$AgNO_3$	169.9	0.03	5.1
**G**	291.3	0.195	56.8
DCE	98.9	3.2	313.2
Product **1**	189.1	0.3	56.7
**E factor** = *mass of total waste/mass of product*			**9.9**

Table S15. E factor calculation for the oxazolidinone synthesis based on the model reaction.

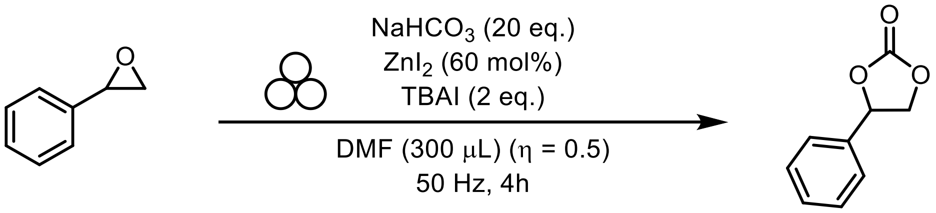

R28 28

Substance	PM	n (mmol)	weight (mg)
Substrate **R28**	120.1	0.3	36.0
$NaHCO_3$	84.0	6	504.0
ZnI_2	319.2	0.18	57.5
TBAI	369.4	0.6	221.6
DMF	73.1	3.9	284.4
Product **28**	164.1	0.3	49.2
**E factor** = *mass of total waste/mass of product*			**21.4**

Table S16. E factor calculation for the cyclic carbonate synthesis based on the model reaction.

6. Thermogravimetric Analysis

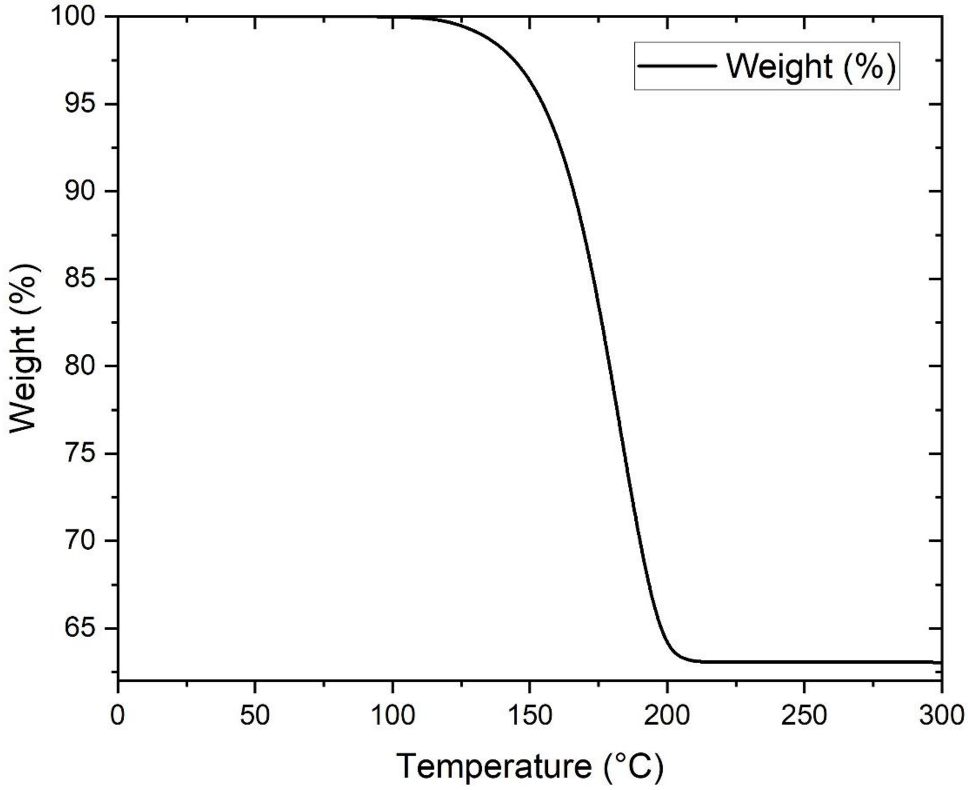

Figure S6. Thermogravimetric analysis (TGA) of $NaHCO_3$.

7. Calculations on Weight Loss during Milling

Control experiments on weight variation were performed by accurately measuring the sample weight before and after milling at 30 Hz, 60 °C for 2 hours using a Fritsch P23 mill, equipped with a 15 mL stainless steel jar and a single stainless-steel ball (Ø: 15 mm, 13.5 g).

Entry 1: 210.0 mg of $NaHCO_3$ (2.5 mmol) were weighed directly into the milling vessel. The milling ball was then added, and the jar was sealed. The total weight of the closed jar with its contents was recorded. The jar was immediately subjected to milling for 2 hours at 30 Hz, 60 °C. After milling, once the jar had returned to room temperature, it was carefully opened to release any potential overpressure, promptly resealed, and weighed again as a whole.

Entry 2: 210.0 mg of $NaHCO_3$ (2.5 mmol) and 56.8 mg of **G** (0.195 mmol) were weighed directly into the milling vessel. The milling ball was then added, and the jar was sealed. The total weight of the closed jar with its contents was recorded. The jar was immediately subjected to milling for 2 hours at 30 Hz, 60 °C. After milling, once the jar had returned to room temperature, it was carefully opened to release any potential overpressure, promptly resealed, and weighed again as a whole.

Entry	Initial Weight ($NaHCO_3$) (mg)	Initial Weight (G) (mg)	Initial Total Weight (jar) (g)	Final Total Weight (jar) (g)	Weight Difference (mg)	Theoretical CO_2 (mmol)
1	210.0	0	102.3329	102.3247	8.2	0.19
2	210.0	56.8	102.3817	102.3811	0.6	0.01

Table S17. Calculations on weight loss during milling of $NaHCO_3$ (2.5 mmol) and a mixture of $NaHCO_3$ (2.5 mmol) and **G** (0.195 mmol).

8. Characterization Data

8.1 Propargylic Amines

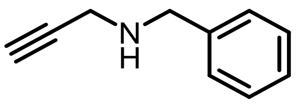

N-benzylprop-2-yn-1-amine ***(R1).*** Prepared according to GP1A. Pale yellow oil, (191.7 mg, yield 88%). ^{1}**H NMR** (400 MHz, $CDCl_3$) δ 7.42 – 7.34 (m, 4H), 7.29 (ddd, *J* = 6.1, 4.7, 2.4 Hz, 1H), 3.91 (s, 2H), 3.46 (d, *J* = 2.4 Hz, 2H), 2.29 (t, *J* = 2.4 Hz, 1H), 1.62 (s, 1H). ^{13}**C{^{1}H} NMR** (101 MHz, $CDCl_3$) δ 139.4, 128.5, 128.4, 127.2, 82.1, 71.6, 52.3, 37.3.

The NMR data closely match the ones previously reported in the literature [11].

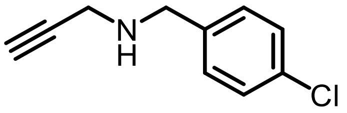

N-(4-chlorobenzyl)prop-2-yn-1-amine ***(R2).*** Prepared according to GP1B. Pale yellow oil, (247.9 mg, yield 92%). ^{1}**H NMR** (400 MHz, $CDCl_3$) δ 7.28 (s, 4H), 3.83 (s, 2H), 3.39 (d, *J* = 2.4 Hz, 2H), 2.28 (t, *J* = 2.4 Hz, 1H), 1.55 (s, 1H). ^{13}**C{^{1}H} NMR** (101 MHz, $CDCl_3$) δ 137.9, 132.8, 129.8, 128.5, 81.9, 71.8, 51.4, 37.2.

The NMR data closely match the ones previously reported in the literature [11]

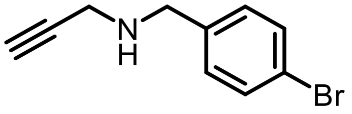

N-(4-bromobenzyl)prop-2-yn-1-amine ***(R3).*** Prepared according to GP1B. Yellow oil, (312.6 mg, yield 93%). ^{1}**H NMR** (400 MHz, $CDCl_3$) δ 7.52 – 7.42 (m, 2H), 7.29 – 7.20 (m, 2H), 3.85 (d, J = 1.4 Hz, 2H), 3.42 (dt, *J* = 2.2, 0.9 Hz, 2H), 2.28 (t, *J* = 2.4 Hz, 1H), 1.61 (s, 1H). ^{13}**C{^{1}H} NMR** (101 MHz, $CDCl_3$) δ 138.4, 131.5, 130.1, 121.0, 81.8, 71.8, 51.5, 37.2.

The NMR data closely match the ones previously reported in the literature [11]

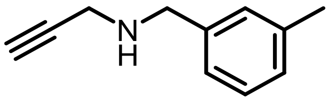

N-(3-methylbenzyl)prop-2-yn-1-amine ***(R4).*** Prepared according to GP1B. Yellow oil, (138.5 mg, yield 58%). ^{1}**H NMR** (400 MHz, $CDCl_3$) δ 7.25 (t, *J* = 7.5 Hz, 1H), 7.20 (s, 1H), 7.17 (d, *J* = 7.6 Hz, 1H), 7.11 (d, *J* = 7.4 Hz, 1H), 3.88 (s, 2H), 3.46 (d, *J* = 2.4 Hz, 2H), 2.38 (s, 3H), 2.29 (t, *J* = 2.4 Hz, 1H), 1.80 (s, 1H). ^{13}**C{^{1}H} NMR** (101 MHz, $CDCl_3$) δ 139.2, 138.1, 129.2, 128.4, 128.0, 125.5, 82.0, 71.7, 52.3, 37.4, 21.4.

The NMR data closely match the ones previously reported in the literature [11]

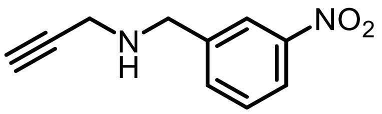

N-(3-nitrobenzyl)prop-2-yn-1-amine *(R5)*. Prepared according to GP1B. Yellow oil, (156.8 mg, yield 55%). **^{1}H NMR** (400 MHz, $CDCl_3$) δ 8.24 (s, 1H), 8.12 (d, *J* = 8.2 Hz, 1H), 7.71 (d, *J* = 7.6 Hz, 1H), 7.50 (t, *J* = 7.9 Hz, 1H), 4.00 (s, 2H), 3.45 (d, *J* = 2.4 Hz, 2H), 2.30 (t, *J* = 2.4 Hz, 1H), 2.17 (s, 1H). **^{13}C{^{1}H} NMR** (101 MHz, $CDCl_3$) δ 148.4, 141.6, 134.5, 129.3, 123.2, 122.3, 81.4, 72.2, 51.2, 37.3.

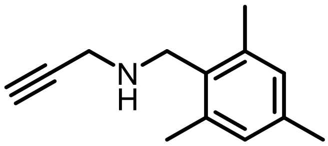

N-(2,4,6-trimethylbenzyl)prop-2-yn-1-amine *(R6)*. Prepared according to GP1B. Yellow oil, (132.0 mg, yield 47%). **^{1}H NMR** (400 MHz, $CDCl_3$) δ 6.88 (s, 2H), 3.87 (s, 2H), 3.52 (d, J = 2.4 Hz, 2H), 2.41 (s, 6H), 2.31 (t, J = 2.4 Hz, 1H), 2.29 (s, 3H), 1.44 (s, 1H). **^{13}C{^{1}H} NMR** (101 MHz, $CDCl_3$) δ 137.2, 136.7, 132.9, 129.0, 82.5, 71.4, 46.2, 38.2, 20.9, 19.4.

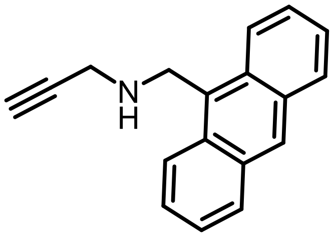

N-(anthracen-9-ylmethyl)prop-2-yn-1-amine *(R7).* Prepared according to GP1B. Yellow solid, (345.9 mg, yield 94%). **^{1}H NMR** (400 MHz, $CDCl_3$) δ 8.48 – 8.42 (m, 3H), 8.04 (dd, *J* = 8.3, 1.3 Hz, 2H), 7.58 (ddd, *J* = 9.0, 6.5, 1.4 Hz, 2H), 7.50 (ddd, *J* = 7.9, 6.6, 1.1 Hz, 2H), 4.88 (s, 2H), 3.68 (d, *J* = 2.4 Hz, 2H), 2.48 (t, *J* = 2.4 Hz, 1H), 1.83 (s, 1H). **^{13}C{^{1}H} NMR** (101 MHz, $CDCl_3$) δ 131.5, 130.6, 130.5, 129.2, 127.6, 126.3, 125.0, 124.1, 82.4, 72.0, 44.3, 38.4.

The NMR data closely match the ones previously reported in the literature [12]

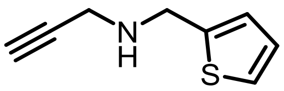

N-(thiophen-2-ylmethyl)prop-2-yn-1-amine *(R8).* Prepared according to GP1B. Yellow oil, (215.6 mg, yield 95%). **^{1}H NMR** (400 MHz, $CDCl_3$) δ 7.24 (dd, J = 4.8, 1.5 Hz, 1H), 7.03 – 6.94 (m, 2H), 4.11 (s, 2H), 3.47 (d, J = 2.4 Hz, 2H), 2.29 (t, J = 2.4 Hz, 1H), 1.75 (s, 1H). **^{13}C{^{1}H} NMR** (101 MHz, $CDCl_3$) δ 142.9, 126.7, 125.5, 124.8, 81.7, 71.9, 46.7, 37.0.

The NMR data closely match the ones previously reported in the literature [13]

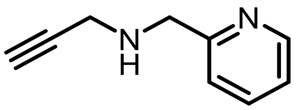

N-(pyridin-2-ylmethyl)prop-2-yn-1-amine ***(R9).*** Prepared according to GP1B. Yellow oil, (206.1 mg, yield 94%). **^{1}H NMR** (400 MHz, $CDCl_3$) δ 8.59 – 8.53 (m, 1H), 7.64 (td, J = 7.5, 1.6 Hz, 1H), 7.32 (d, J = 7.8 Hz, 1H), 7.17 (dd, J = 7.5, 4.9 Hz, 1H), 4.00 (s, 2H), 3.49 (dd, J = 2.4, 0.9 Hz, 2H), 2.26 – 2.24 (m, 2H). **^{13}C{^{1}H} NMR** (101 MHz, $CDCl_3$) δ 159.0, 149.4, 136.5, 122.4, 122.1, 81.8, 71.7, 53.6, 37.7.

The NMR data closely match the ones previously reported in the literature [14]

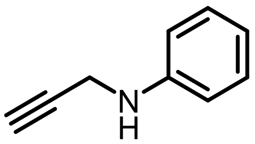

N-(prop-2-yn-1-yl)aniline ***(R10).*** Prepared according to GP1A. Yellow oil, (177.1 mg, yield 90%). **^{1}H NMR** (400 MHz, $CDCl_3$) δ 7.30 (td, *J* = 7.3, 2.1 Hz, 2H), 6.87 (t, *J* = 7.4 Hz, 1H), 6.76 (dd, *J* = 8.7, 1.1 Hz, 2H), 3.98 (d, *J* = 2.5 Hz, 2H), 2.29 (t, *J* = 2.4 Hz, 1H). **^{13}C{^{1}H} NMR** (101 MHz, $CDCl_3$) δ 146.9, 129.3, 118.7, 113.6, 81.2, 71.4, 33.7.

The NMR data closely match the ones previously reported in the literature [15]

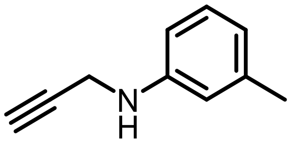

3-methyl-N-(prop-2-yn-1-yl)aniline ***(R11).*** Prepared according to GP1A. Yellow oil, (139.4 mg, yield 64%). **^{1}H NMR** (400 MHz, $CDCl_3$) δ 7.14 (t, *J* = 8.1 Hz, 1H), 6.65 (d, *J* = 7.5 Hz, 1H), 6.54 (d, *J* = 5.9 Hz, 2H), 3.96 (s, 2H), 3.84 (bs, 1H), 2.33 (s, 3H), 2.24 (t, *J* = 2.4 Hz, 1H). **^{13}C{^{1}H} NMR** (101 MHz, $CDCl_3$) δ 146.9, 139.1, 129.1, 119.6, 114.3, 110.7, 81.1, 71.2, 33.7, 21.6.

The NMR data closely match the ones previously reported in the literature [16]

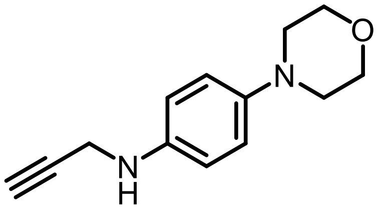

4-morpholino-N-(prop-2-yn-1-yl)aniline ***(R12).*** Prepared according to GP1A. Yellow oil, (126.6 mg, yield 40%). **^{1}H NMR** (400 MHz, $CDCl_3$) δ 6.93 – 6.84 (m, 2H), 6.75 – 6.67 (m, 2H), 3.93 (d, *J* = 2.5 Hz, 2H), 3.91 – 3.84 (m, 4H), 3.68 (bs, 1H), 3.10 – 3.01 (m, 4H), 2.23 (t, *J* = 2.4 Hz, 1H). **^{13}C{^{1}H} NMR** (101 MHz, $CDCl_3$) δ 144.7, 141.3, 118.2, 115.0, 81.5, 71.4, 67.2, 51.1, 34.5.

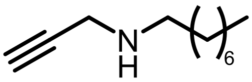

N-(prop-2-yn-1-yl)octan-1-amine ***(R13).*** Prepared according to GP1A. Yellow oil, (205.7 mg, yield 82%). ^{1}**H NMR** (400 MHz, $CDCl_3$) δ 3.44 (s, 2H), 2.69 (t, *J* = 7.2 Hz, 2H), 2.22 – 2.21 (t, 1H), 1.50 (p, J = 7.1 Hz, 2H), 1.43 (bs, 1H), 1.30 (d, *J* = 11.2 Hz, 10H), 0.89 (s, 3H). ^{13}**C{^{1}H} NMR** (101 MHz, $CDCl_3$) δ 82.3, 71.2, 48.7, 38.2, 31.8, 29.8, 29.5, 29.3, 27.3, 22.7, 14.1.

The NMR data closely match the ones previously reported in the literature [17]

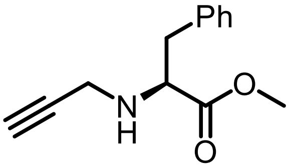

methyl prop-2-yn-1-yl-D-phenylalaninate ***(R14).*** Prepared according to GP1A. Colourless oil, (46.3 mg, yield 71%). ^{1}**H NMR** (400 MHz, $CDCl_3$) δ 7.35 – 7.29 (m, 2H), 7.28 – 7.19 (m, 3H), 3.78 (dd, *J* = 7.2, 6.3 Hz, 1H), 3.70 (s, 3H), 3.46 (dd, *J* = 17.0, 2.5 Hz, 1H), 3.39 (dd, *J* = 17.0, 2.5 Hz, 1H), 3.05 (dd, *J* = 13.6, 6.2 Hz, 1H), 2.98 (dd, *J* = 13.7, 7.3 Hz, 1H), 2.21 (t, *J* = 2.5 Hz, 1H), 1.80 (s, 1H). ^{13}**C{^{1}H} NMR** (101 MHz, $CDCl_3$) δ 174.3, 136.8, 129.2, 128.5, 126.9, 81.1, 71.8, 61.1, 51.8, 39.4, 36.8.

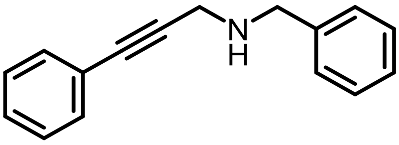

N-benzyl-3-phenylprop-2-yn-1-amine ***(R15).*** Prepared according to GP1C. Dark orange oil, (182.6 mg, yield 55%). ^{1}**H NMR** (400 MHz, $CDCl_3$) δ 7.54 – 7.45 (m, 2H), 7.45 – 7.37 (m, 4H), 7.37 – 7.29 (m, 4H), 3.99 (s, 2H), 3.69 (s, 2H), 1.75 (s, 1H). ^{13}**C{^{1}H} NMR** (101 MHz, $CDCl_3$) δ 139.5, 131.7, 128.5 (2C), 128.3, 128.1, 127.2, 123.3, 87.6, 83.8, 52.6, 38.3.

The NMR data closely match the ones previously reported in the literature [11]

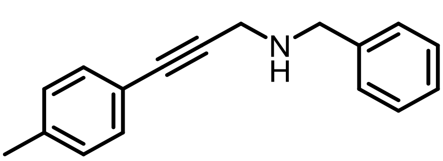

N-benzyl-3-(p-tolyl)prop-2-yn-1-amine ***(R16).*** Prepared according to GP1C. Dark orange oil, (338.8 mg, yield 96%). ^{1}**H NMR** (400 MHz, $CDCl_3$) δ 7.46 – 7.34 (m, 6H), 7.34 – 7.28 (m, 1H), 7.16 (d, *J* = 8.0 Hz, 2H), 3.99 (s, 2H), 3.69 (s, 2H), 2.39 (s, 3H), 1.71 (s, 1H). ^{13}**C{^{1}H} NMR** (101 MHz, $CDCl_3$) δ 139.6, 138.2, 131.6, 129.1, 128.5 (2C), 127.2, 120.2, 86.8, 83.9, 52.5, 38.3, 21.5.

The NMR data closely match the ones previously reported in the literature [3]

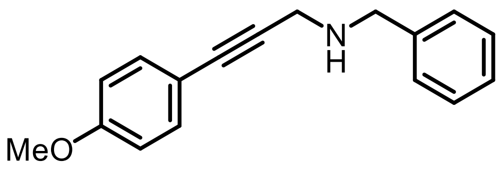

N-benzyl-3-(4-methoxyphenyl)prop-2-yn-1-amine ***(R17).*** Prepared according to GP1C. Orange solid, (211.1 mg, yield 56%). **^{1}H NMR** (400 MHz, $CDCl_3$) δ 7.44 – 7.34 (m, 6H), 7.32 – 7.27 (m, 1H), 6.91 – 6.83 (m, 2H), 3.97 (s, 2H), 3.83 (s, 3H), 3.67 (s, 2H), 1.77 (bs, 1H). **^{13}C{^{1}H} NMR** (101 MHz, $CDCl_3$) δ 159.4, 139.6, 133.1, 128.5 (2C), 127.2, 115.4, 113.9, 86.0, 83.6, 55.3, 52.5, 38.3.

The NMR data closely match the ones previously reported in the literature [3]

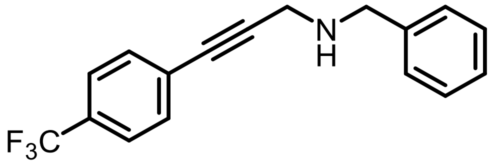

N-benzyl-3-(4-(trifluoromethyl)phenyl)prop-2-yn-1-amine ***(R18).*** Prepared according to GP1C. Pale yellow solid, (230.0 mg, yield 53%). **^{1}H NMR** (400 MHz, $CDCl_3$) δ 7.58 (q, J = 8.4 Hz, 4H), 7.44 – 7.36 (m, 4H), 7.34 – 7.29 (m, 1H), 3.99 (s, 2H), 3.71 (s, 2H), 1.85 (s, 1H). **^{13}C{^{1}H} NMR** (101 MHz, $CDCl_3$) δ 139.3, 131.9, 129.9 (q, J = 32.5 Hz), 128.5, 128.4, 127.3, 127.1, 125.24 (q, J = 4.0 Hz), 121.2 (d, J = 272.1 Hz), 90.2, 82.6, 52.6, 38.2. **^{19}F NMR** (565 MHz, $CDCl_3$) δ -62.7.

The NMR data closely match the ones previously reported in the literature [3]

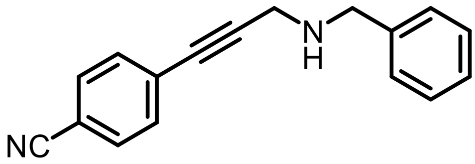

4-(3-(benzylamino)prop-1-yn-1-yl)benzonitrile ***(R19).*** Prepared according to GP1C. Dark orange oil, (206.9 mg, yield 56%). **^{1}H NMR** (400 MHz, $CDCl_3$) δ 7.72 – 7.55 (m, 2H), 7.55 – 7.51 (m, 2H), 7.42 – 7.30 (m, 5H), 3.97 (s, 2H), 3.71 (s, 2H), 1.92 (bs, 1H). **^{13}C{^{1}H} NMR** (101 MHz, $CDCl_3$) δ 139.0, 132.2, 132.0, 128.6, 128.4, 128.1, 127.4, 126.6, 118.5, 111.5, 82.4, 52.5, 38.1.

The NMR data closely match the ones previously reported in the literature [18]

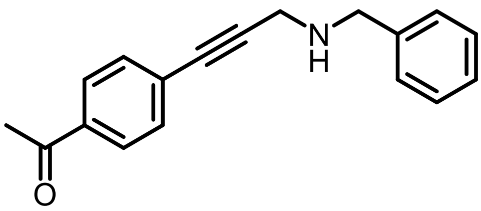

1-(4-(3-(benzylamino)prop-1-yn-1-yl)phenyl)ethan-1-one ***(R20).*** Prepared according to GP1C. Orange oil, (276.5 mg, yield 70%). **^{1}H NMR** (400 MHz, $CDCl_3$) δ 7.96 – 7.87 (m, 2H), 7.57 – 7.49 (m, 2H), 7.43 – 7.32 (m, 4H), 7.29 (tt, *J* = 6.1, 1.7 Hz, 1H), 3.98 (bs, 2H), 3.70 (bs, 2H), 2.61 (s, 3H), 1.85 (bs, 1H). **^{13}C{^{1}H} NMR** (101 MHz, $CDCl_3$) δ 197.3, 139.4, 136.1, 131.8, 128.5, 128.4, 128.3, 128.2, 127.3, 91.2, 83.2, 52.6, 38.2, 26.6.

The NMR data closely match the ones previously reported in the literature [18]

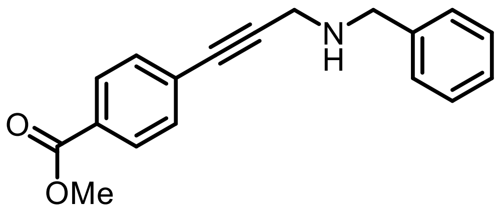

methyl 4-(3-(benzylamino)prop-1-yn-1-yl)benzoate ***(R21).*** Prepared according to GP1C. Orange oil, (238.8 mg, yield 57%). **^{1}H NMR** (400 MHz, $CDCl_3$) δ 8.06 – 7.96 (m, 2H), 7.55 – 7.48 (m, 2H), 7.44 – 7.35 (m, 4H), 7.33 – 7.29 (m, 1H), 3.99 (s, 2H), 3.94 (s, 3H), 3.71 (s, 2H), 2.05 (s, 1H). **^{13}C{^{1}H} NMR** (101 MHz, $CDCl_3$) δ 166.6, 139.0, 131.6, 129.5, 129.4, 128.6, 128.5, 127.9, 127.3, 90.4, 83.3, 52.4, 52.2, 38.1.

The NMR data closely match the ones previously reported in the literature [18]

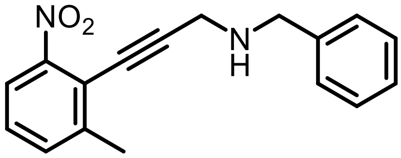

N-benzyl-3-(2-methyl-6-nitrophenyl)prop-2-yn-1-amine ***(R22).*** Prepared according to GP1C. Dark orange oil, (126.0 mg, yield 30%). **^{1}H NMR** (400 MHz, $CDCl_3$) δ 7.81 (dd, *J* = 8.3, 1.2 Hz, 1H), 7.50 (d, *J* = 7.6 Hz, 1H), 7.45 – 7.41 (m, 2H), 7.39 – 7.34 (m, 3H), 7.29 (q, *J* = 3.1 Hz, 1H), 4.03 (s, 2H), 3.78 (s, 2H), 2.56 (s, 3H), 1.94 (s, 1H). **^{13}C{^{1}H} NMR** (101 MHz, $CDCl_3$) δ 151.1, 143.2, 139.3, 133.7, 128.6, 128.5 (2C), 127.7, 127.3, 121.8, 117.8, 100.7, 52.3, 38.4, 21.4.

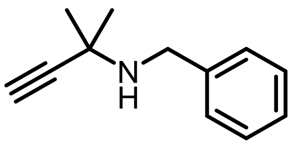

N-benzyl-2-methylbut-3-yn-2-amine ***(R23).*** Prepared according to GP1B. White solid, (252.1 mg, yield 97%). ^{1}**H NMR** (400 MHz, $CDCl_3$) δ 7.45 – 7.25 (m, 5H), 3.92 (s, 2H), 2.41 (d, *J* = 1.0 Hz, 1H), 1.47 (s, 6H), 1.39 (bs, 1H). ^{13}**C{^{1}H} NMR** (101 MHz, $CDCl_3$) δ 140.6, 128.5 (2C), 127.0, 89.1, 69.9, 50.1, 49.0, 29.6.

The NMR data closely match the ones previously reported in the literature [11]

8.2 Epoxides

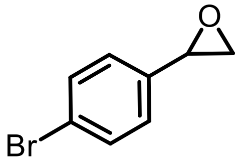

2-(4-bromophenyl)oxirane *(R29).* Prepared according to GP2F. Pale yellow solid, (269.0 mg, yield 90%). **^{1}H NMR** (400 MHz, $CDCl_3$) δ 7.53 – 7.45 (m, 2H), 7.22 – 7.12 (m, 2H), 3.85 (dd, *J* = 4.1, 2.6 Hz, 1H), 3.17 (dd, *J* = 5.5, 4.0 Hz, 1H), 2.77 (dd, *J* = 5.5, 2.5 Hz, 1H). **^{13}C{^{1}H} NMR** (101 MHz, $CDCl_3$) δ 136.7, 131.7, 127.2, 122.0, 51.9, 51.2.

The NMR data closely match the ones previously reported in the literature [5]

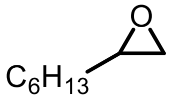

2-hexyloxirane *(R30).* Prepared according to GP2E. Colourless oil, (58.0 mg, yield 30%). **^{1}H NMR** (400 MHz, $CDCl_3$) δ 2.91 (tt, *J* = 5.9, 3.3 Hz, 1H), 2.75 (dd, *J* = 5.0, 4.0 Hz, 1H), 2.46 (dd, *J* = 5.1, 2.7 Hz, 1H), 1.57 – 1.40 (m, 4H), 1.39 – 1.25 (m, 6H), 0.89 (t, *J* = 6.7 Hz, 3H). **^{13}C{^{1}H} NMR** (101 MHz, $CDCl_3$) δ 52.4, 47.1, 32.5, 31.8, 29.1, 25.9, 22.5, 14.0.

The NMR data closely match the ones previously reported in the literature [19]

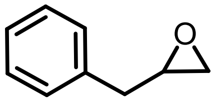

2-benzyloxirane *(R32).* Prepared according to GP2E. Colourless oil, (151.0 mg, yield 75%). **^{1}H NMR** (400 MHz, $CDCl_3$) δ 7.40 – 7.31 (m, 2H), 7.29 (d, *J* = 7.5 Hz, 3H), 3.19 (qd, *J* = 5.7, 3.2 Hz, 1H), 2.96 (dd, *J* = 14.5, 5.6 Hz, 1H), 2.90 – 2.80 (m, 2H), 2.59 (dd, *J* = 5.0, 2.7 Hz, 1H). **^{13}C{^{1}H} NMR** (101 MHz, $CDCl_3$) δ 137.2, 129.0, 128.5, 126.7, 52.5, 46.9, 38.8.

The NMR data closely match the ones previously reported in the literature [20]

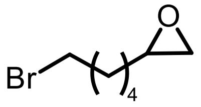

2-(5-bromopentyl)oxirane *(R33).* Prepared according to GP2E. Pale yellow oil, (255.0 mg, yield 88%). **^{1}H NMR** (400 MHz, $CDCl_3$) δ 3.41 (td, *J* = 6.8, 1.5 Hz, 2H), 2.95 – 2.86 (m, 1H), 2.75 (ddd, *J* = 6.0, 4.3, 1.7 Hz, 1H), 2.47 (dt, *J* = 4.7, 2.2 Hz, 1H), 1.87 (tt, *J* = 6.4, 3.4 Hz, 2H), 1.66 – 1.41 (m, 6H). **^{13}C{^{1}H} NMR** (101 MHz, $CDCl_3$) δ 52.1, 47.0, 33.7, 32.6, 32.3, 27.9, 25.2.

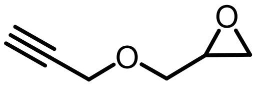

2-((prop-2-yn-1-yloxy)methyl)oxirane ***(R34).*** Prepared according to GP2H. Colourless oil, (70.6 mg, yield 42%). **^{1}H NMR** (400 MHz, $CDCl_3$) δ 4.31 – 4.17 (m, 2H), 3.85 (dd, J = 11.3, 3.0 Hz, 1H), 3.51 (dd, J = 11.3, 5.9 Hz, 1H), 3.20 (ddd, J = 5.9, 4.1, 2.9 Hz, 1H), 2.83 (dd, J = 5.0, 4.1 Hz, 1H), 2.66 (dd, J = 5.0, 2.7 Hz, 1H), 2.47 (t, J = 2.4 Hz, 1H). **^{13}C{^{1}H} NMR** (101 MHz, $CDCl_3$) δ 79.3, 74.8, 70.3, 58.5, 50.5, 44.3.

The NMR data closely match the ones previously reported in the literature [7]

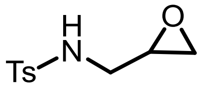

4-methyl-N-(oxiran-2-ylmethyl)benzenesulfonamide ***(R40)***. Prepared according to GP1E. White solid, (327.0 mg, yield 96%); **m.p.** 68.8-69.7 °C. **^{1}H NMR** (400 MHz, $CDCl_3$) δ 7.81 – 7.73 (m, 2H), 7.37 – 7.30 (m, 2H), 4.82 (t, J = 6.1 Hz, 1H), 3.36 (ddd, J = 13.4, 6.7, 2.3 Hz, 1H), 3.13 – 3.00 (m, 2H), 2.81 – 2.74 (m, 1H), 2.66 (dd, J = 4.7, 2.3 Hz, 1H), 2.45 (s, 3H). **^{13}C{^{1}H} NMR** (101 MHz, $CDCl_3$) δ 143.7, 136.8, 129.8, 127.1, 50.3, 45.1, 44.3, 21.5.

The NMR data closely match the ones previously reported in the literature [21]

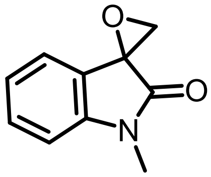

1-methylspiro[indoline-3,2'-oxiran]-2-one ***(R43).*** Prepared according to GP1G. Orange solid, (210.2 mg, yield 80%). **^{1}H NMR** (400 MHz, $CDCl_3$) δ 7.41 (td, J = 7.5, 1.9 Hz, 1H), 7.18 – 7.05 (m, 2H), 6.95 (d, J = 7.8 Hz, 1H), 3.61 (d, J = 6.7 Hz, 1H), 3.46 (d, J = 6.7 Hz, 1H), 3.31 (s, 3H). **^{13}C{^{1}H} NMR** (101 MHz, $CDCl_3$) δ 171.8, 145.1, 130.4, 122.9, 122.7, 122.1, 108.9, 56.4, 54.1, 26.7.

The NMR data closely match the ones previously reported in the literature [6]

8.3 Oxazolidinones

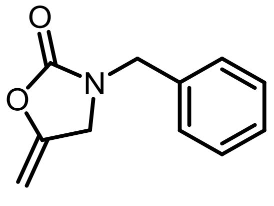

3-benzyl-5-methyleneoxazolidin-2-one *(1).* White solid, (53.9 mg, yield 95%). **^{1}H NMR** (400 MHz, $CDCl_3$) δ 7.42 – 7.32 (m, 3H), 7.32 – 7.27 (m, 2H), 4.75 (q, J = 2.8 Hz, 1H), 4.48 (s, 2H), 4.26 (q, J = 2.5 Hz, 1H), 4.04 (t, J = 2.4 Hz, 2H). **^{13}C{^{1}H} NMR** (101 MHz, $CDCl_3$) δ 155.7, 149.0, 135.0, 129.0, 128.3, 128.2, 86.8, 47.9, 47.2.

The NMR data closely match the ones previously reported in the literature [13]

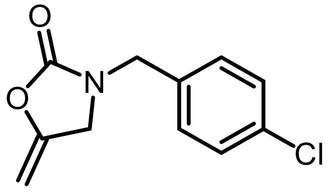

3-(4-chlorobenzyl)-5-methyleneoxazolidin-2-one *(2).* White solid, (56.9 mg, yield 85%). **^{1}H NMR** (400 MHz, $CDCl_3$) δ 7.36 – 7.32 (m, 2H), 7.25 – 7.21 (m, 2H), 4.75 (ddt, J = 4.6, 3.5, 2.2 Hz, 1H), 4.44 (s, 2H), 4.27 (dtd, J = 3.2, 2.2, 0.7 Hz, 1H), 4.03 (t, J = 2.4 Hz, 2H). **^{13}C{^{1}H} NMR** (101 MHz, $CDCl_3$) δ 155.6, 148.7, 134.2, 133.6, 129.5, 129.2, 87.0, 47.2, 47.2.

The NMR data closely match the ones previously reported in the literature [13]

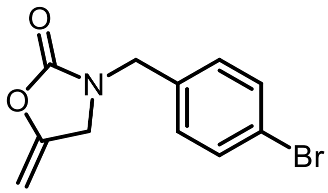

3-(4-bromobenzyl)-5-methyleneoxazolidin-2-one *(3).* White solid, (62.5 mg, yield 78%). **^{1}H NMR** (400 MHz, $CDCl_3$) δ 7.54 – 7.46 (m, 2H), 7.21 – 7.13 (m, 2H), 4.76 (dq, J = 3.2, 2.0 Hz, 1H), 4.43 (d, J = 1.8 Hz, 2H), 4.27 (dq, J = 3.6, 2.0 Hz, 1H), 4.03 (t, J = 2.5 Hz, 2H). **^{13}C{^{1}H} NMR** (101 MHz, $CDCl_3$) δ 155.6, 148.7, 134.1, 132.1, 129.9, 122.3, 87.1, 47.2 (2C).

The NMR data closely match the ones previously reported in the literature [13]

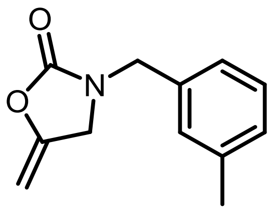

3-(3-methylbenzyl)-5-methyleneoxazolidin-2-one ***(4).*** Yellow oil, (41.4 mg, yield 68%). ^{1}**H NMR** (400 MHz, $CDCl_3$) δ 7.33 – 7.23 (m, 1H), 7.15 (d, J = 7.6 Hz, 1H), 7.12 – 7.06 (m, 2H), 4.75 (q, J = 2.7 Hz, 1H), 4.44 (s, 2H), 4.26 (dt, J = 3.2, 2.2 Hz, 1H), 4.04 (t, J = 2.4 Hz, 2H), 2.37 (s, 3H). ^{13}**C{^{1}H} NMR** (101 MHz, $CDCl_3$) δ 155.7, 149.0, 138.8, 134.9, 129.0, 128.9, 128.8, 125.2, 86.7, 47.8, 47.2, 21.4.

The NMR data closely match the ones previously reported in the literature [22]

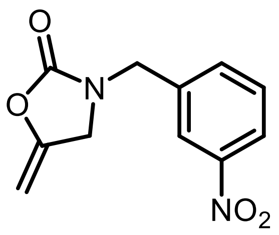

5-methylene-3-(3-nitrobenzyl)oxazolidin-2-one ***(5).*** Yellow solid, (63.9 mg, yield 91%); **m.p.** 108.7 – 110.0 °C. ^{1}**H NMR** (400 MHz, $CDCl_3$) δ 8.22 (ddd, J = 8.1, 2.3, 1.2 Hz, 1H), 8.15 (t, J = 2.0 Hz, 1H), 7.69 (dt, J = 7.7, 1.5 Hz, 1H), 7.60 (t, J = 7.9 Hz, 1H), 4.82 (q, J = 2.7 Hz, 1H), 4.60 (s, 2H), 4.33 (dt, J = 3.3, 2.2 Hz, 1H), 4.12 (t, J = 2.4 Hz, 2H). ^{13}**C{^{1}H} NMR** (101 MHz, $CDCl_3$) δ 155.7, 148.6, 148.3, 137.3, 134.1, 130.2, 123.4, 122.9, 87.6, 47.5, 47.3. **HRMS (ESI)** m/z calculated for $C_{11}H_{10}N_2NaO_4^+$ $[M+Na]^+$: 257.0533, found: 257.0534.

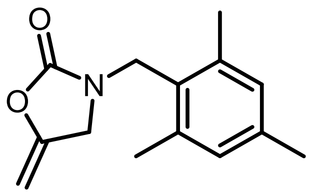

5-methylene-3-(2,4,6-trimethylbenzyl)oxazolidin-2-one ***(6).*** Colourless oil, (45.8 mg, yield 66%). ^{1}**H NMR** (400 MHz, $CDCl_3$) δ 6.90 (s, 2H), 4.72 (q, J = 2.8 Hz, 1H), 4.57 (s, 2H), 4.19 (q, J = 2.4 Hz, 1H), 3.87 (t, J = 2.4 Hz, 2H), 2.34 (s, 6H), 2.30 (s, 3H). ^{13}**C{^{1}H} NMR** (101 MHz, $CDCl_3$) δ 155.1, 149.2, 138.1, 137.7, 129.5, 127.6, 86.5, 46.6, 41.4, 20.9, 19.9.

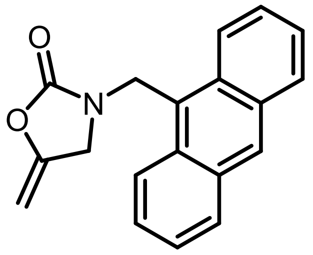

3-(anthracen-9-ylmethyl)-5-methyleneoxazolidin-2-one ***(7).*** Yellow solid, (34.7 mg, yield 40%); **m.p.** 153.0 – 154.2 °C. **^{1}H NMR** (400 MHz, $CDCl_3$) δ 8.54 (s, 1H), 8.39 (dt, J = 8.9, 1.0 Hz, 2H), 8.12 – 8.05 (m, 2H), 7.63 (ddd, J = 8.9, 6.5, 1.4 Hz, 2H), 7.55 (ddd, J = 7.7, 6.6, 1.1 Hz, 2H), 5.55 (s, 2H), 4.64 (q, J = 2.8 Hz, 1H), 4.05 (dt, J = 3.2, 2.2 Hz, 1H), 3.80 (t, J = 2.4 Hz, 2H). **^{13}C{^{1}H} NMR** (101 MHz, $CDCl_3$) δ 155.3, 149.1, 131.3, 130.9, 129.5, 129.1, 127.2, 125.3, 125.0, 123.3, 86.6, 47.1, 39.4. **HRMS (ESI)** m/z calculated for $C_{19}H_{15}NNaO_2^+$ $[M+Na]^+$: 312.0995, found: 312.0997.

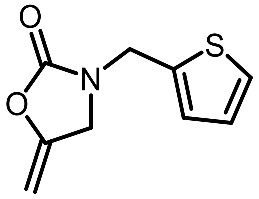

5-methylene-3-(thiophen-2-ylmethyl)oxazolidin-2-one ***(8).*** Pale yellow oil, (55.6 mg, yield 95%). **^{1}H NMR** (400 MHz, $CDCl_3$) δ 7.31 (dt, J = 5.0, 1.3 Hz, 1H), 7.05 – 7.02 (m, 1H), 7.00 (ddd, J = 4.7, 3.5, 1.1 Hz, 1H), 4.76 (dq, J = 4.5, 2.6 Hz, 1H), 4.67 (s, 2H), 4.28 (q, J = 2.5 Hz, 1H), 4.12 (t, J = 2.4 Hz, 2H). **^{13}C{^{1}H} NMR** (101 MHz, $CDCl_3$) δ 155.2, 148.8, 137.1, 127.5, 127.2, 126.3, 87.0, 47.1, 42.2.

The NMR data closely match the ones previously reported in the literature [22]

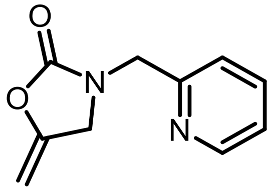

5-methylene-3-(pyridin-2-ylmethyl)oxazolidin-2-one ***(9).*** Dark orange oil, (45.1 mg, yield 79%). **^{1}H NMR** (400 MHz, $CDCl_3$) δ 8.56 (dq, J = 4.4, 1.4 Hz, 1H), 7.70 (tt, J = 7.7, 1.8 Hz, 1H), 7.33 – 7.29 (m, 1H), 7.24 (ddt, J = 7.7, 4.8, 1.4 Hz, 1H), 4.74 (p, J = 2.6 Hz, 1H), 4.58 (d, J = 1.7 Hz, 2H), 4.29 – 4.25 (m, 1H), 4.23 (q, J = 2.1 Hz, 2H). **^{13}C{^{1}H} NMR** (101 MHz, $CDCl_3$) δ 155.9, 155.1, 149.6, 149.1, 137.2, 123.0, 122.3, 86.7, 49.2, 48.1. **HRMS (ESI)** m/z calculated for $C_{10}H_{10}N_2NaO_2^+$ $[M+Na]^+$: 213.0634, found: 213.0633.

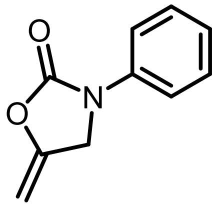

5-methylene-3-phenyloxazolidin-2-one *(10).* White solid, (18.4 mg, yield 35%). **^{1}H NMR** (400 MHz, $CDCl_3$) δ 7.62 – 7.53 (m, 2H), 7.47 – 7.37 (m, 2H), 7.24 – 7.15 (m, 1H), 4.91 – 4.85 (m, 1H), 4.67 (t, *J* = 2.4 Hz, 2H), 4.46 (dt, *J* = 3.4, 2.2 Hz, 1H). **^{13}C{^{1}H} NMR** (101 MHz, $CDCl_3$) δ 152.4, 147.7, 137.2, 129.3, 124.6, 118.1, 87.1, 48.4.

The NMR data closely match the ones previously reported in the literature [23]

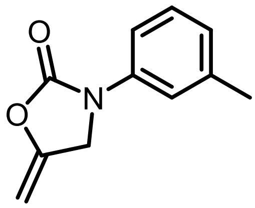

5-methylene-3-(m-tolyl)oxazolidin-2-one *(11).* White solid, (55.0 mg, yield 97%). **^{1}H NMR** (400 MHz, $CDCl_3$) δ 7.41 (s, 1H), 7.37 – 7.25 (m, 2H), 7.01 (d, J = 7.1 Hz, 1H), 4.87 (q, J = 2.8 Hz, 1H), 4.65 (t, J = 2.4 Hz, 2H), 4.44 (dt, J = 3.3, 2.2 Hz, 1H), 2.40 (s, 3H). **^{13}C{^{1}H} NMR** (101 MHz, $CDCl_3$) δ 152.4, 147.8, 139.3, 137.1, 129.1, 125.4, 118.9, 115.3, 87.0, 48.6, 21.6.

The NMR data closely match the ones previously reported in the literature [24]

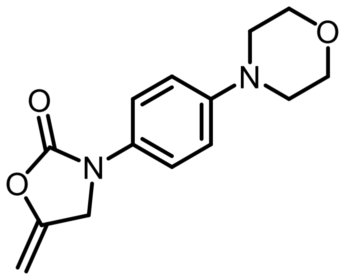

5-methylene-3-(4-morpholinophenyl)oxazolidin-2-one *(12).* Orange solid, (50.7 mg, yield 65%); **m.p.** 134.7 – 136.8 °C. **^{1}H NMR** (400 MHz, $CDCl_3$) δ 7.49 – 7.40 (m, 2H), 6.99 – 6.90 (m, 2H), 4.85 (q, J = 2.8 Hz, 1H), 4.62 (t, J = 2.4 Hz, 2H), 4.42 (dt, J = 3.3, 2.2 Hz, 1H), 3.95 – 3.84 (m, 4H), 3.19 – 3.12 (m, 4H). **^{13}C{^{1}H} NMR** (101 MHz, $CDCl_3$) δ 152.6, 148.5, 148.0, 129.7, 119.9, 116.3, 86.9, 66.8, 49.5, 48.9. **HRMS (ESI)** m/z calculated for $C_{14}H_{16}N_2NaO_3^+$ [M+Na]$^+$: 283.1053, found: 283.1058.

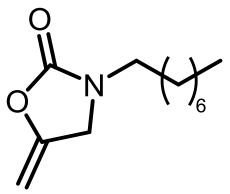

5-methylene-3-octyloxazolidin-2-one (*13).* Pale yellow oil, (62.1 mg, yield 98%). **^{1}H NMR** (400 MHz, $CDCl_3$) δ 4.75 (q, *J* = 2.7 Hz, 1H), 4.29 (q, *J* = 2.5 Hz, 1H), 4.17 (t, *J* = 2.4 Hz, 2H), 3.34 – 3.26 (m, 2H), 1.56 (t, *J* = 7.2 Hz, 2H), 1.37 – 1.20 (m, 10H), 0.93 – 0.85 (m, 3H). **^{13}C{^{1}H} NMR** (101 MHz, $CDCl_3$) δ 155.6, 149.2, 86.4, 47.8, 43.8, 31.7, 29.1 (2C), 27.3, 26.6, 22.6, 14.1.

The NMR data closely match the ones previously reported in the literature [25]

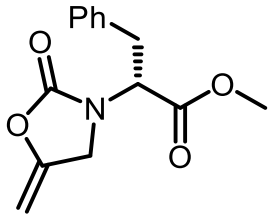

Methyl (R)-2-(5-methylene-2-oxooxazolidin-3-yl)-3-phenylpropanoate *(14).* Colourless oil, (61.1 mg, yield 78%). **^{1}H NMR** (400 MHz, $CDCl_3$) δ 7.38 – 7.19 (m, 5H), 4.89 (dd, J = 10.8, 5.5 Hz, 1H), 4.72 (q, J = 2.7 Hz, 1H), 4.34 (dt, J = 13.7, 2.4 Hz, 1H), 4.27 (dt, J = 3.2, 2.2 Hz, 1H), 4.11 (dt, J = 13.7, 2.4 Hz, 1H), 3.78 (s, 3H), 3.40 (dd, J = 14.7, 5.4 Hz, 1H), 3.01 (dd, J = 14.8, 10.8 Hz, 1H). **^{13}C{^{1}H} NMR** (101 MHz, $CDCl_3$) δ 170.4, 155.6, 149.0, 135.6, 128.9, 128.4, 127.3, 86.9, 56.3, 52.7, 45.4, 35.1. **HRMS (ESI)** m/z calculated for $C_{14}H_{15}NNaO_4^+$ $[M+Na]^+$: 284.0893, found: 284.0896.

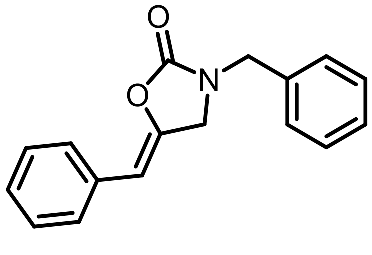

(Z)-3-benzyl-5-benzylideneoxazolidin-2-one *(15).* Dark orange solid, (76.4 mg, yield 96%). **^{1}H NMR** (400 MHz, $CDCl_3$) δ 7.62 – 7.55 (m, 2H), 7.45 – 7.30 (m, 7H), 7.27 – 7.19 (m, 1H), 5.47 (t, J = 2.1 Hz, 1H), 4.53 (s, 2H), 4.16 (d, J = 2.1 Hz, 2H). **^{13}C{^{1}H} NMR** (101 MHz, $CDCl_3$) δ 155.6, 141.8, 134.9, 133.5, 129.1, 128.5, 128.4, 128.3, 128.2, 126.9, 103.1, 48.2, 47.9.

The NMR data closely match the ones previously reported in the literature [22]

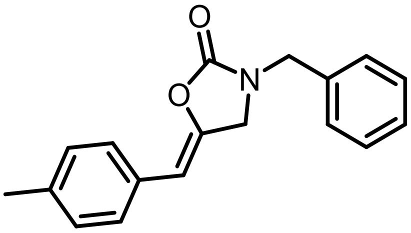

(Z)-3-benzyl-5-(4-methylbenzylidene)oxazolidin-2-one ***(16).*** White solid, (61.2 mg, yield 73%); **m.p.** 184.1 – 186.3 °C. **^{1}H NMR** (400 MHz, $CDCl_3$) δ 7.51 – 7.44 (m, 2H), 7.44 – 7.35 (m, 3H), 7.35 – 7.30 (m, 2H), 7.16 (d, J = 8.0 Hz, 2H), 5.44 (t, J = 2.1 Hz, 1H), 4.54 (s, 2H), 4.17 (d, J = 2.1 Hz, 2H), 2.36 (s, 3H). **^{13}C{^{1}H} NMR** (101 MHz, $CDCl_3$) δ 155.8, 140.9, 136.7, 134.9, 130.5, 129.2, 129.0, 128.3, 128.2, 128.1, 103.1, 48.2, 47.9, 21.2. **HRMS (ESI)** m/z calculated for $C_{18}H_{17}NNaO_2^+$ [M+Na]$^+$: 302.1151, found: 302.1148.

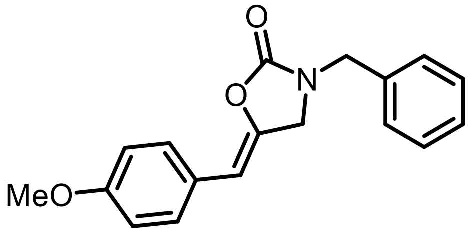

(Z)-3-benzyl-5-(4-methoxybenzylidene)oxazolidin-2-one ***(17).*** Yellow solid, (69.1 mg, yield 78%); **m.p.** 144.6 – 146.7 °C. **^{1}H NMR** (400 MHz, $CDCl_3$) δ 7.53 – 7.50 (m, 2H), 7.42 – 7.28 (m, 5H), 6.92 – 6.84 (m, 2H), 5.41 (t, J = 2.1 Hz, 1H), 4.53 (s, 2H), 4.16 (d, J = 2.1 Hz, 2H), 3.82 (s, 3H). **^{13}C{^{1}H} NMR** (101 MHz, $CDCl_3$) δ 158.4, 155.8, 140.0, 135.0, 129.5, 129.0, 128.3, 128.2, 126.2, 113.9, 102.7, 55.3, 48.1, 47.9. **HRMS (ESI)** m/z calculated for $C_{18}H_{17}NNaO_3^+$ [M+Na]$^+$: 318.1100, found: 318.1098.

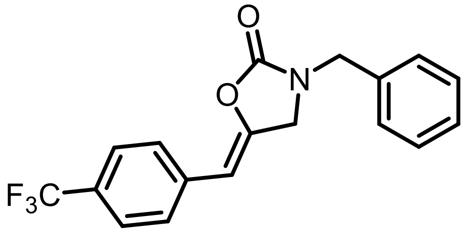

(Z)-3-benzyl-5-(4-(trifluoromethyl)benzylidene)oxazolidin-2-one ***(18 exo).*** Yellow solid, (77.0 mg, yield 77%); **m.p.** 191.3 – 193.7 °C. **^{1}H NMR** (400 MHz, $CDCl_3$) δ 7.66 (d, J = 8.2 Hz, 2H), 7.57 (d, J = 8.3 Hz, 2H), 7.46 – 7.29 (m, 5H), 5.51 (t, J = 2.1 Hz, 1H), 4.55 (s, 2H), 4.21 (d, J = 2.0 Hz, 2H). **^{13}C{^{1}H} NMR** (101 MHz, $CDCl_3$) δ 155.2, 144.0, 137.0, 134.7, 129.1 (d), 128.5 (q, J = 15.6 Hz), 128.3, 128.2 (2C), 125.4 (q, J = 3.9 Hz), 122.8, 101.9, 48.2, 47.9. **^{19}F NMR** (565 MHz, $CDCl_3$) δ -62.34. **HRMS (ESI)** m/z calculated for $C_{18}H_{14}F_3NNaO_2^+$ [M+Na]$^+$: 356.0869, found: 356.0873.

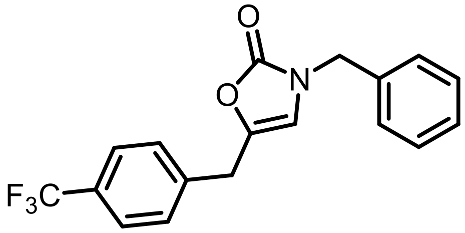

3-benzyl-5-(4-(trifluoromethyl)benzyl)oxazol-2(3H)-one *(18 endo).* Yellow solid, (21 mg, yield 21%); **m.p.** 157.2 – 158.1 °C. **^{1}H NMR** (400 MHz, $CDCl_3$) δ 7.59 (d, J = 8.0 Hz, 2H), 7.46 – 7.33 (m, 5H), 7.31 – 7.25 (m, 2H), 6.06 (t, J = 1.3 Hz, 1H), 4.70 (s, 2H), 3.77 (s, 2H). **^{13}C{^{1}H} NMR** (101 MHz, $CDCl_3$) δ 155.5, 139.6, 138.6, 135.4, 129.9 – 128.7 (m), 129.1, 129.0, 128.4, 128.0 (2C), 125.9 – 125.4 (m), 110.8, 47.7, 32.4. **^{19}F NMR** (565 MHz, $CDCl_3$) δ -62.45. **HRMS (ESI)** m/z calculated for $C_{18}H_{14}F_3NNaO_2^+$ $[M+Na]^+$: 356.0869, found: 356.0868.

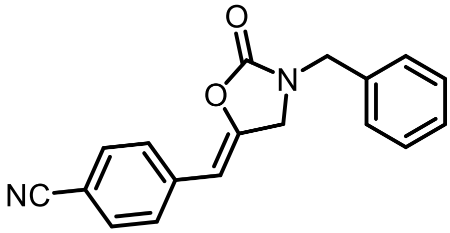

(Z)-4-((3-benzyl-2-oxooxazolidin-5-ylidene)methyl)benzonitrile *(19 exo).* Dark orange solid (13.0 mg, yield 15%); **m.p.** 169.3 – 171.5 °C. **^{1}H NMR** (400 MHz, $CDCl_3$) δ 7.63 (q, J = 8.6 Hz, 4H), 7.45 – 7.35 (m, 3H), 7.33 (dd, J = 7.8, 1.7 Hz, 2H), 5.50 (t, J = 2.1 Hz, 1H), 4.56 (s, 2H), 4.23 (d, J = 2.0 Hz, 2H). **^{13}C{^{1}H} NMR** (101 MHz, $CDCl_3$) δ 154.9, 145.1, 138.0, 134.5, 132.3, 129.1, 128.6, 128.5, 128.2, 119.0, 109.9, 101.7, 48.3, 48.0. **HRMS (ESI)** m/z calculated for $C_{18}H_{14}N_2NaO_2^+$ $[M+Na]^+$: 313.0947, found: 313.0950.

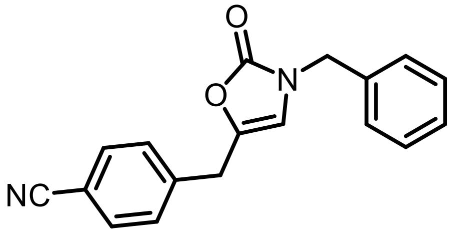

4-((3-benzyl-2-oxo-2,3-dihydrooxazol-5-yl)methyl)benzonitrile *(19 endo).* White solid, (65.3 mg, yield 75%); **m.p.** 107.8 – 108.9 °C. **^{1}H NMR** (400 MHz, $CDCl_3$) δ 7.61 – 7.54 (m, 2H), 7.41 – 7.28 (m, 5H), 7.30 – 7.24 (m, 2H), 6.15 (s, 1H), 4.67 (s, 2H), 3.75 (s, 2H). **^{13}C{^{1}H} NMR** (101 MHz, $CDCl_3$) δ 155.5, 141.2, 137.9, 135.4, 132.5, 129.6, 129.0, 128.4, 128.0, 118.7, 111.3, 111.1, 47.6, 32.5. **HRMS (ESI)** m/z calculated for $C_{18}H_{14}N_2NaO_2^+$ $[M+Na]^+$: 313.0947, found: 313.0948.

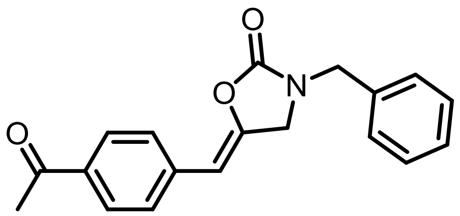

(Z)-5-(4-acetylbenzylidene)-3-benzyloxazolidin-2-one ***(20 exo).*** Pale yellow solid, (24.9 mg, yield 27%); **m.p.** 146.5 – 148.9 °C. **^{1}H NMR** (400 MHz, $CDCl_3$) δ 7.96 – 7.88 (m, 2H), 7.67 – 7.61 (m, 2H), 7.40 (dddd, J = 10.5, 6.7, 4.4, 2.5 Hz, 3H), 7.36 – 7.31 (m, 2H), 5.55 – 5.49 (m, 1H), 4.56 (s, 2H), 4.22 (d, J = 2.0 Hz, 2H), 2.61 (s, 3H). **^{13}C{^{1}H} NMR** (101 MHz, $CDCl_3$) δ 197.6, 155.2, 144.2, 138.2, 135.1, 134.7, 129.1, 128.6, 128.5, 128.2, 128.2, 102.3, 48.4, 48.0, 26.6. **HRMS (ESI)** m/z calculated for $C_{19}H_{17}NNaO_3^+$ [M+Na]$^+$: 330.1101, found: 330.1103.

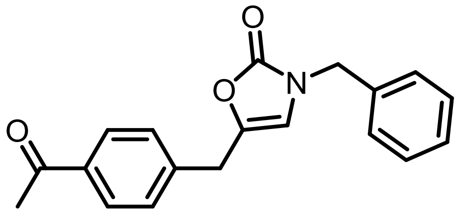

5-(4-acetylbenzyl)-3-benzyloxazol-2(3H)-one ***(20 endo).*** Dark orange solid, (65.5 mg, yield 71%); **m.p.** 123.0 – 125.1 °C.**^{1}H NMR** (400 MHz, $CDCl_3$) δ 7.96 – 7.87 (m, 2H), 7.43 – 7.30 (m, 5H), 7.27 (dd, *J* = 8.0, 1.9 Hz, 2H), 6.06 (t, *J* = 1.3 Hz, 1H), 4.68 (s, 2H), 3.76 (d, *J* = 1.3 Hz, 2H), 2.60 (s, 3H). **^{13}C{^{1}H} NMR** (101 MHz, $CDCl_3$) δ 197.7, 155.6, 141.0, 138.7, 136.1, 135.4, 129.0, 128.8, 128.4, 128.2, 128.0, 110.8, 47.6, 32.5, 26.6. **HRMS (ESI)** m/z calculated for $C_{19}H_{17}NNaO_3^+$ [M+Na]$^+$: 330.1101, found: 330.1106.

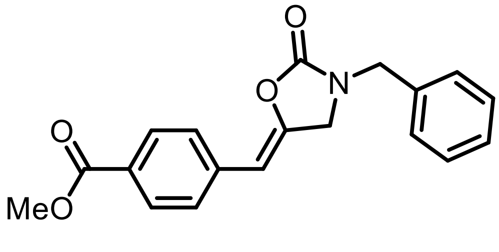

methyl (Z)-4-((3-benzyl-2-oxooxazolidin-5-ylidene)methyl)benzoate (21 exo). Yellow solid, (24.2 mg, yield 25%); **m.p.** 128.1 – 128.9 °C. **^{1}H NMR** (400 MHz, $CDCl_3$) δ 8.03 – 7.95 (m, 2H), 7.65 – 7.58 (m, 2H), 7.40 (dddd, J = 10.5, 6.7, 4.4, 2.5 Hz, 3H), 7.35 – 7.29 (m, 2H), 5.51 (t, J = 2.1 Hz, 1H), 4.55 (s, 2H), 4.21 (d, J = 2.0 Hz, 2H), 3.92 (s, 3H). **^{13}C{^{1}H} NMR** (101 MHz, $CDCl_3$) δ 166.9, 155.2, 144.0, 138.0, 134.7, 129.8, 129.1, 128.4, 128.2, 128.1, 128.0, 102.4, 52.1, 48.3, 47.9. **HRMS (ESI)** m/z calculated for $C_{19}H_{17}NNaO_4^+$ [M+Na]$^+$: 346.1050, found: 346.1054.

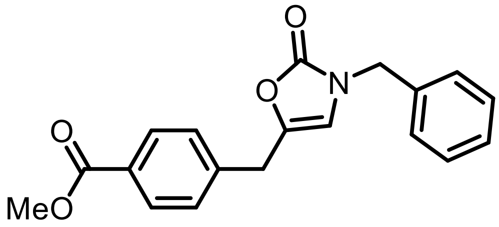

methyl 4-((3-benzyl-2-oxo-2,3-dihydrooxazol-5-yl)methyl)benzoate (21 endo). Yellow solid, (53.3 mg, yield 55%); **m.p.** 132.2 – 134.5 °C. ^{1}**H NMR** (400 MHz, $CDCl_3$) δ 8.03 – 7.96 (m, 2H), 7.41 – 7.34 (m, 3H), 7.33 – 7.26 (m, 4H), 6.03 (t, *J* = 1.4 Hz, 1H), 4.69 (s, 2H), 3.93 (s, 3H), 3.76 (d, *J* = 1.3 Hz, 2H). ^{13}**C{^{1}H} NMR** (101 MHz, $CDCl_3$) δ 166.8, 155.6, 140.7, 138.8, 135.4, 130.0, 129.1, 129.0, 128.8, 128.4, 128.0, 110.7, 52.2, 47.6, 32.5. **HRMS (ESI)** m/z calculated for $C_{19}H_{17}NNaO_4^+$ $[M+Na]^+$: 346.1050, found: 346.1047.

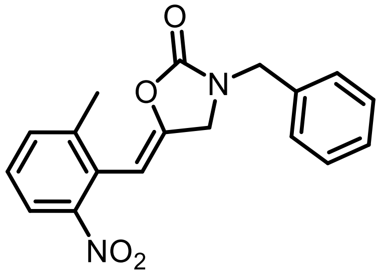

(Z)-3-benzyl-5-(2-methyl-6-nitrobenzylidene)oxazolidin-2-one *(22).* Yellow solid, (38.9 mg, yield 40%); **m.p.** 129.5 – 129.9 °C. ^{1}**H NMR** (400 MHz, $CDCl_3$) δ 7.77 (dd, *J* = 8.2, 1.3 Hz, 1H), 7.48 (d, *J* = 7.6 Hz, 1H), 7.41 (dddd, *J* = 10.5, 6.7, 4.4, 2.4 Hz, 3H), 7.35 – 7.30 (m, 3H), 5.83 (t, *J* = 2.2 Hz, 1H), 4.51 (s, 2H), 4.22 (d, *J* = 2.2 Hz, 2H), 2.38 (s, 3H). ^{13}**C{^{1}H} NMR** (101 MHz, $CDCl_3$) δ 154.9, 149.4, 143.6, 139.7, 134.7, 134.6, 129.1, 128.4, 128.3, 127.8, 126.8, 122.0, 96.9, 48.1, 47.6, 20.5. **HRMS (ESI)** m/z calculated for $C_{18}H_{16}N_2NaO_4^+$ $[M+Na]^+$: 347.1002, found: 347.1004.

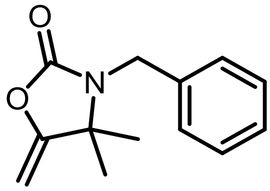

3-benzyl-4,4-dimethyl-5-methyleneoxazolidin-2-one *(23).* Yellow solid, (20.8 mg, yield 32%). ^{1}**H NMR** (400 MHz, CDCl3) δ 7.39 – 7.26 (m, 5H), 4.69 (d, J = 3.4 Hz, 1H), 4.47 (s, 2H), 4.24 (d, J = 3.3 Hz, 1H), 1.32 (s, 6H). ^{13}**C{^{1}H} NMR** (101 MHz, CDCl3) δ 160.8, 154.9, 137.6, 128.7, 127.8, 127.8, 84.2, 61.6, 44.1, 27.7.

The NMR data closely match the ones previously reported in the literature [26]

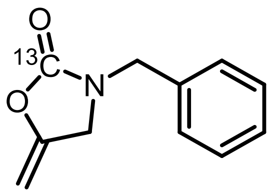

3-benzyl-5-methyleneoxazolidin-2-one-2-13C (*24*). White solid, (53.9 mg, yield 95%). **^{1}H NMR** (400 MHz, $CDCl_3$) δ 7.42 – 7.32 (m, 3H), 7.32 – 7.27 (m, 2H), 4.75 (q, *J* = 2.8 Hz, 1H), 4.48 (s, 2H), 4.26 (q, *J* = 2.5 Hz, 1H), 4.04 (t, *J* = 2.4 Hz, 2H). **$^{13}C\{^1H\}$ NMR** (101 MHz, $CDCl_3$) δ 155.7 (^{13}C enriched), 149.0, 135.0, 129.0, 128.3, 128.2, 86.8, 47.9, 47.2. **HRMS (ESI)** m/z calculated for $C_{10}{}^{13}CH_{11}NNaO_2^+$ $[M+Na]^+$: 213.0715, found: 213.0716.

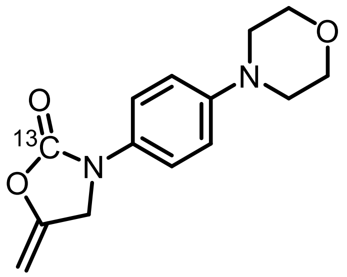

5-methylene-3-(4-morpholinophenyl)oxazolidin-2-one-2-13C *(25).* Orange solid, (50.7 mg, yield 65%). **^{1}H NMR** (400 MHz, $CDCl_3$) δ 7.48 – 7.40 (m, 2H), 6.99 – 6.90 (m, 2H), 4.85 (q, *J* = 2.8 Hz, 1H), 4.62 (q, *J* = 2.4 Hz, 2H), 4.42 (q, *J* = 2.5 Hz, 1H), 3.92 – 3.83 (m, 4H), 3.19 – 3.12 (m, 4H). **$^{13}C\{^1H\}$ NMR** (101 MHz, $CDCl_3$) δ 152.6 (^{13}C enriched), 148.5, 148.0, 129.7 (d, *J* = 2.7 Hz), 119.8, 116.3, 86.9 (d, *J* = 3.9 Hz), 66.8, 49.5, 48.8 (d, *J* = 4.7 Hz). **HRMS (ESI)** m/z calculated for $C_{13}{}^{13}CH_{16}N_2NaO_3^+$ $[M+Na]^+$: 284.1086, found: 284.1085.

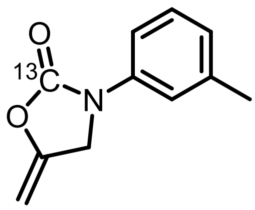

5-methylene-3-(m-tolyl)oxazolidin-2-one-2-13C *(26).* White solid, (55.0 mg, yield 97%). **^{1}H NMR** (400 MHz, $CDCl_3$) δ 7.41 (d, *J* = 2.1 Hz, 1H), 7.35 – 7.28 (m, 2H), 7.04 – 6.98 (m, 1H), 4.87 (q, *J* = 2.8 Hz, 1H), 4.65 (q, *J* = 2.4 Hz, 2H), 4.47 – 4.41 (m, 1H), 2.40 (s, 3H). **$^{13}C\{^1H\}$ NMR** (101 MHz, $CDCl_3$) δ 152.4 (^{13}C enriched), 147.8, 139.3, 137.1 (d, *J* = 2.7 Hz), 129.1, 125.4, 118.9, 115.3, 87.0 (d, *J* = 3.7 Hz), 48.5 (d, *J* = 4.6 Hz), 21.6. **HRMS (ESI)** m/z calculated for $C_{10}{}^{13}CH_{11}NNaO_2^+$ $[M+Na]^+$: 213.0715, found: 213.0718.

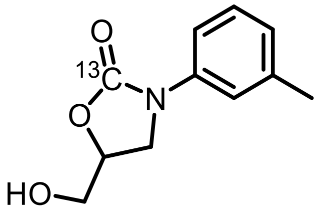

5-(hydroxymethyl)-3-(m-tolyl)oxazolidin-2-one-2-13C (Toloxatone) ***(27).*** White solid (23.4 mg, yield 75%). **^{1}H NMR** (400 MHz, $CDCl_3$) δ 7.40 (d, J = 2.1 Hz, 1H), 7.37 – 7.31 (m, 1H), 7.30 – 7.26 (m, 1H), 6.98 (d, J = 7.3 Hz, 1H), 4.74 (tdd, J = 6.7, 5.4, 3.4 Hz, 1H), 4.11 – 3.94 (m, 3H), 3.77 (dd, J = 12.6, 4.2 Hz, 1H), 2.59 (bs, 1H), 2.38 (s, 3H). **^{13}C{^{1}H} NMR** (101 MHz, $CDCl_3$) δ 154.8 (^{13}C enriched), 139.0, 138.0, 128.9, 125.1, 119.1, 115.5, 72.8, 62.9, 46.5 (d, J = 5.7 Hz), 21.6. **HRMS (ESI)** m/z calculated for $C_{10}{}^{13}CH_{13}NNaO_3^+$ $[M+Na]^+$: 231.0821, found: 231.0824.

8.4 Cyclic Carbonates

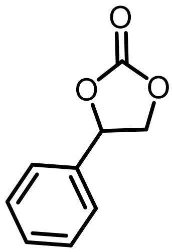

4-phenyl-1,3-dioxolan-2-one *(28).* White solid, (48.5 mg, yield 98%). **^{1}H NMR** (400 MHz, $CDCl_3$) δ 7.49 – 7.41 (m, 3H), 7.38 (dd, J = 7.4, 2.2 Hz, 2H), 5.70 (t, J = 8.0 Hz, 1H), 4.82 (t, J = 8.4 Hz, 1H), 4.36 (t, J = 8.2 Hz, 1H). **^{13}C{^{1}H} NMR** (101 MHz, $CDCl_3$) δ 154.9, 135.8, 129.7, 129.3, 125.9, 78.0, 71.2.

The NMR data closely match the ones previously reported in the literature [27]

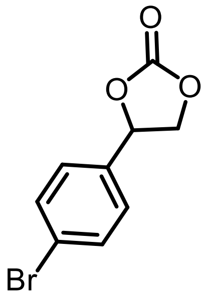

4-(4-bromophenyl)-1,3-dioxolan-2-one *(29).* Pale yellow solid (65.6 mg, yield 90%). **^{1}H NMR** (400 MHz, $CDCl_3$) δ 7.62 – 7.55 (m, 2H), 7.26 (d, J = 8.5 Hz, 2H), 5.66 (t, J = 8.0 Hz, 1H), 4.82 (t, J = 8.4 Hz, 1H), 4.35 – 4.27 (m, 1H). **^{13}C{^{1}H} NMR** (101 MHz, $CDCl_3$) δ 154.6, 134.9, 132.5, 127.6, 123.9, 77.3, 71.0.

The NMR data closely match the ones previously reported in the literature [28]

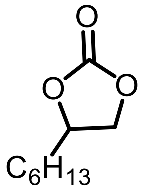

4-hexyl-1,3-dioxolan-2-one *(30).* White solid, (46.4 mg, yield 90%). **^{1}H NMR** (400 MHz, $CDCl_3$) δ 4.72 (qd, J = 7.5, 5.3 Hz, 1H), 4.54 (t, J = 8.1 Hz, 1H), 4.14 – 4.04 (m, 1H), 1.81 (ddd, J = 17.1, 9.1, 5.4 Hz, 1H), 1.69 (ddt, J = 14.6, 10.7, 5.1 Hz, 1H), 1.54 – 1.22 (m, 8H), 0.90 (t, J = 6.6 Hz, 3H). **^{13}C{^{1}H} NMR** (101 MHz, $CDCl_3$) δ 155.1, 77.1, 69.4, 33.9, 31.5, 28.8, 24.3, 22.5, 14.0.

The NMR data closely match the ones previously reported in the literature [29]

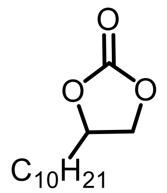

4-decyl-1,3-dioxolan-2-one *(31).* White solid, (67.7 mg, yield 99%). ^{1}**H NMR** (400 MHz, $CDCl_3$) δ 4.71 (qd, J = 7.5, 5.4 Hz, 1H), 4.54 (t, J = 8.1 Hz, 1H), 4.08 (dd, J = 8.4, 7.2 Hz, 1H), 1.92 – 1.77 (m, 1H), 1.69 (td, J = 9.4, 5.1 Hz, 1H), 1.53 – 1.44 (m, 1H), 1.40 – 1.20 (m, 15H), 0.89 (t, J = 6.7 Hz, 3H). ^{13}**C{^{1}H} NMR** (101 MHz, $CDCl_3$) δ 155.1, 77.1, 69.4, 33.9, 31.9, 29.5, 29.4, 29.3, 29.3, 29.1, 24.4, 22.7, 14.1.

The NMR data closely match the ones previously reported in the literature [28]

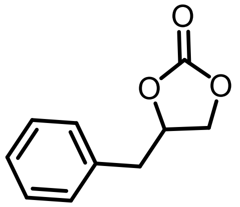

4-benzyl-1,3-dioxolan-2-one *(32).* White solid, (39.6 mg, yield 74%). ^{1}**H NMR** (400 MHz, $CDCl_3$) δ 7.40 – 7.28 (m, 3H), 7.28 – 7.21 (m, 2H), 4.96 (dq, J = 7.9, 6.6 Hz, 1H), 4.46 (dd, J = 8.6, 7.8 Hz, 1H), 4.19 (dd, J = 8.6, 6.9 Hz, 1H), 3.18 (dd, J = 14.2, 6.2 Hz, 1H), 3.01 (dd, J = 14.2, 6.6 Hz, 1H). ^{13}**C{^{1}H} NMR** (101 MHz, $CDCl_3$) δ 154.8, 133.9, 129.4, 129.0, 127.6, 76.9, 68.5, 39.6.

The NMR data closely match the ones previously reported in the literature [30]

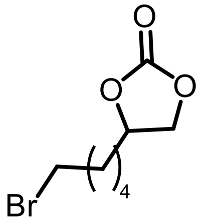

4-(5-bromopentyl)-1,3-dioxolan-2-one (*33*). Pale yellow oil (67.5 mg, yield 95%). ^{1}**H NMR** (400 MHz, $CDCl_3$) δ 4.73 (qd, J = 7.7, 5.0 Hz, 1H), 4.55 (t, J = 8.1 Hz, 1H), 4.08 (dd, J = 8.4, 7.1 Hz, 1H), 3.21 (t, J = 6.9 Hz, 2H), 1.94 – 1.79 (m, 3H), 1.72 (td, J = 9.5, 4.9 Hz, 1H), 1.61 – 1.36 (m, 4H). ^{13}**C{^{1}H} NMR** (101 MHz, $CDCl_3$) δ 155.0, 76.8, 69.3, 33.7, 33.0, 30.0, 23.5, 6.6. **HRMS (ESI)** m/z calculated for $C_8H_{13}BrNaO_3^+$ $[M+Na]^+$: 258.9940, found: 258.9941.

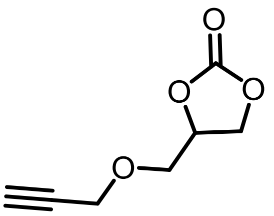

4-((prop-2-yn-1-yloxy)methyl)-1,3-dioxolan-2-one ***(34).*** Colourless oil, (37,4 mg, yield 80%). **^{1}H NMR** (400 MHz, $CDCl_3$) δ 4.87 (ddt, J = 8.1, 6.1, 3.9 Hz, 1H), 4.53 (t, J = 8.4 Hz, 1H), 4.42 (dd, J = 8.4, 6.1 Hz, 1H), 4.34 – 4.18 (m, 2H), 3.78 (qd, J = 10.8, 3.9 Hz, 2H), 2.51 (t, J = 2.4 Hz, 1H). **^{13}C{^{1}H} NMR** (101 MHz, $CDCl_3$) δ 154.8, 78.5, 75.7, 74.7, 68.4, 66.2, 58.9.

The NMR data closely match the ones previously reported in the literature [31]

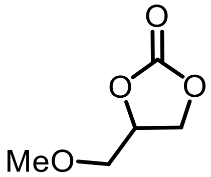

4-(methoxymethyl)-1,3-dioxolan-2-one ***(35).*** Colourless oil, (27,8 mg, yield 70%). **^{1}H NMR** (400 MHz, $CDCl_3$) δ 4.82 (ddt, J = 8.3, 6.1, 3.8 Hz, 1H), 4.51 (t, J = 8.4 Hz, 1H), 4.39 (ddd, J = 8.4, 6.1, 0.8 Hz, 1H), 3.70 – 3.54 (m, 2H), 3.44 (d, J = 0.9 Hz, 3H). **^{13}C{^{1}H} NMR** (101 MHz, $CDCl_3$) δ 154.9, 75.0, 71.5, 66.2, 59.7.

The NMR data closely match the ones previously reported in the literature [30]

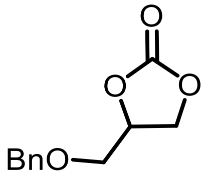

4-((benzyloxy)methyl)-1,3-dioxolan-2-one ***(36).*** Yellow solid, (31.8 mg, yield 51%). **^{1}H NMR** (400 MHz, $CDCl_3$) δ 7.44 – 7.32 (m, 5H), 4.84 (ddt, J = 8.0, 6.0, 3.9 Hz, 1H), 4.62 (q, J = 12.0 Hz, 2H), 4.51 (t, J = 8.4 Hz, 1H), 4.41 (dd, J = 8.4, 6.0 Hz, 1H), 3.76 – 3.61 (m, 2H). **^{13}C{^{1}H} NMR** (101 MHz, $CDCl_3$) δ 154.9, 137.1, 128.6, 128.1, 127.8, 75.0, 73.7, 68.8, 66.3.

The NMR data closely match the ones previously reported in the literature [30]

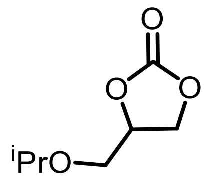

4-(isopropoxymethyl)-1,3-dioxolan-2-one ***(37).*** Colourless oil, (23.1 mg, yield 48%). ^{1}**H NMR** (400 MHz, $CDCl_3$) δ 4.80 (ddt, J = 8.0, 5.9, 3.9 Hz, 1H), 4.50 (t, J = 8.3 Hz, 1H), 4.39 (dd, J = 8.3, 6.0 Hz, 1H), 3.72 – 3.57 (m, 3H), 1.17 (dd, J = 6.2, 1.6 Hz, 6H). ^{13}**C{^{1}H} NMR** (101 MHz, $CDCl_3$) δ 155.1, 75.2, 73.0, 67.1, 66.5, 21.9, 21.8.

The NMR data closely match the ones previously reported in the literature [29]

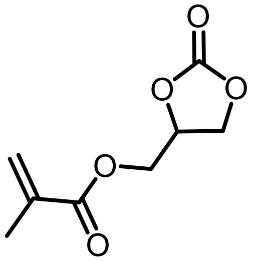

(2-oxo-1,3-dioxolan-4-yl)methyl methacrylate ***(38).*** Colourless oil, (53 mg, yield 95%). ^{1}**H NMR** (400 MHz, $CDCl_3$) δ 6.17 (s, 1H), 5.68 (p, J = 1.5 Hz, 1H), 5.00 (ddt, J = 8.7, 5.6, 3.5 Hz, 1H), 4.60 (t, J = 8.6 Hz, 1H), 4.45 (dd, J = 12.6, 3.2 Hz, 1H), 4.41 – 4.31 (m, 2H), 1.97 (s, 3H). ^{13}**C{^{1}H} NMR** (101 MHz, $CDCl_3$) δ 166.7, 154.5, 135.1, 127.3, 73.8, 66.1, 63.4, 18.2.

The NMR data closely match the ones previously reported in the literature [32]

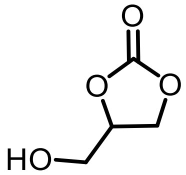

4-(hydroxymethyl)-1,3-dioxolan-2-one ***(39).*** Yellow oil (33.6 mg, yield 95%). ^{1}**H NMR** (400 MHz, $CDCl_3$) δ 4.91 (ddt, J = 8.1, 6.7, 3.5 Hz, 1H), 4.62 – 4.49 (m, 2H), 3.98 (dd, J = 12.7, 3.4 Hz, 1H), 3.85 (dd, J = 12.7, 3.7 Hz, 1H). ^{13}**C{^{1}H} NMR** (101 MHz, $CDCl_3$) δ 155.5, 76.8 ,66.3, 61.6.

The NMR data closely match the ones previously reported in the literature [31]

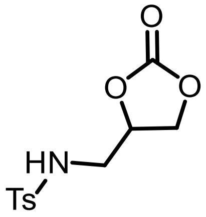

4-methyl-N-((2-oxo-1,3-dioxolan-4-yl)methyl)benzenesulfonamide ***(40).*** Light red solid, (64.0 mg, yield 83%); **m.p.** 127.2 – 127.8 °C. **^{1}H NMR** (400 MHz, $CDCl_3$) δ 7.76 (d, J = 7.9 Hz, 2H), 7.36 (d, J = 7.9 Hz, 2H), 5.26 (bs, 1H), 4.90 – 4.79 (m, 1H), 4.56 (t, J = 8.5 Hz, 1H), 4.43 (dd, J = 8.8, 6.5 Hz, 1H), 3.34 (d, J = 14.5 Hz, 1H), 3.28 – 3.17 (m, 1H), 2.46 (s, 3H). **^{13}C{^{1}H} NMR** (101 MHz, $CDCl_3$) δ 154.5, 144.3, 136.3, 130.1, 129.9, 127.1, 127.0, 74.9, 66.5, 44.5, 21.6.

The NMR data closely match the ones previously reported in the literature [21]

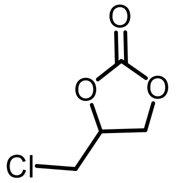

4-(chloromethyl)-1,3-dioxolan-2-one ***(41).*** Colourless oil (36.9 mg, yield 90%). **^{1}H NMR** (400 MHz, $CDCl_3$) δ 4.98 (dtd, J = 8.2, 5.7, 3.9 Hz, 1H), 4.61 (dd, J = 8.9, 8.2 Hz, 1H), 4.44 (dd, J = 8.9, 5.7 Hz, 1H), 3.84 – 3.71 (m, 2H). **^{13}C{^{1}H} NMR** (101 MHz, $CDCl_3$) δ 154.1, 74.2, 67.0, 43.6.

The NMR data closely match the ones previously reported in the literature [31]

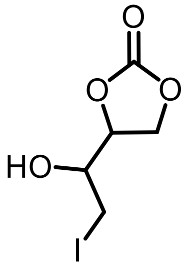

4-(1-hydroxy-2-iodoethyl)-1,3-dioxolan-2-one ***(42).*** Dark orange oil, (52,6 mg, yield 68%). **1H NMR** (400 MHz, $CDCl_3$) δ 4.76 (dt, J = 7.6, 4.5 Hz, 1H), 4.51 (dt, J = 5.5, 2.9 Hz, 1H), 4.07 (dd, J = 12.9, 2.9 Hz, 1H), 3.79 (dd, J = 12.9, 3.1 Hz, 1H), 3.42 (qd, J = 10.7, 5.9 Hz, 2H), 2.91 (bs, 1H). **^{13}C{^{1}H} NMR** (101 MHz, $CDCl_3$) δ 154.1, 82.0, 75.9, 62.0, 4.2. **HRMS (ESI)** m/z calculated for $C_5H_7INaO_4^+$ $[M+Na]^+$: 280.9281, found: 280.9285.

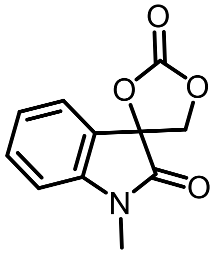

1-methylspiro[indoline-3,4'-[1,3]dioxolane]-2,2'-dione *(43).* White solid, (22.4 mg, yield 34%). **^{1}H NMR** (400 MHz, $CDCl_3$) δ 7.49 (ddd, J = 8.5, 7.5, 1.1 Hz, 2H), 7.22 (td, J = 7.6, 0.9 Hz, 1H), 6.96 – 6.90 (m, 1H), 4.75 (d, J = 8.9 Hz, 1H), 4.56 (d, J = 8.9 Hz, 1H), 3.26 (s, 3H). **^{13}C{^{1}H} NMR** (101 MHz, $CDCl_3$) δ 171.3, 153.7, 144.4, 132.5, 124.9, 124.1, 123.5, 109.4, 79.1, 70.8, 26.7

The NMR data closely match the ones previously reported in the literature [6]

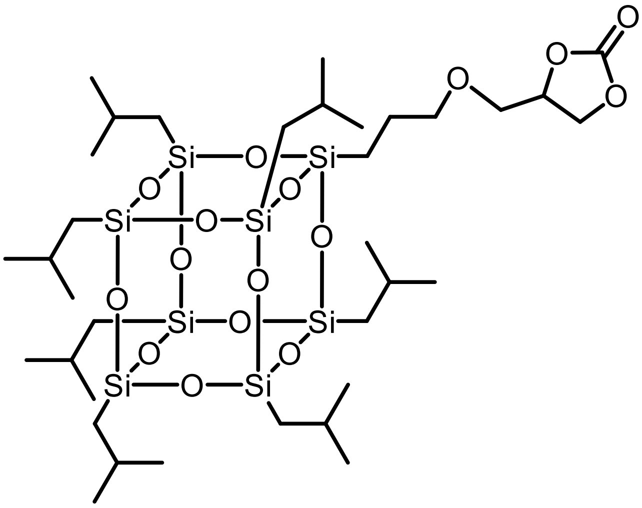

4-((3-(3,5,7,9,11,13,15-heptaisobutyl-2,4,6,8,10,12,14,16,17,18,19,20-dodecaoxa-1,3,5,7,9,11,13,15-octasilapentacyclo[9.5.1.13,9.15,15.17,13]icosan-1-yl)propoxy)methyl)-1,3-dioxolan-2-one *(44).* White solid, (102.4 mg, yield 35%); **m.p.** 142.9 – 144.1 °C. **^{1}H NMR** (400 MHz, $CDCl_3$) δ 4.81 (ddd, J = 8.3, 6.2, 4.2 Hz, 1H), 4.50 (d, J = 8.3 Hz, 1H), 4.42 (d, J = 6.1 Hz, 1H), 3.72 – 3.59 (m, 2H), 3.55 – 3.45 (m, 2H), 1.88 (dpd, J = 13.4, 6.7, 1.7 Hz, 7H), 1.75 – 1.63 (m, 2H), 0.98 (d, J = 6.6 Hz, 42H), 0.63 (dd, J = 7.0, 2.4 Hz, 16H). **^{13}C{^{1}H} NMR** (101 MHz, $CDCl_3$) δ 154.9, 74.9, 74.1, 69.5, 66.4, 25.7, 23.9, 22.8, 22.5, 8.2. **HRMS (ESI)** m/z calculated for $C_{35}H_{75}O_{16}Si_8^+$ $[M+H]^+$: 975.3204, found: 975.3206.

8.5 Iodohydrin **28'** and **44'**

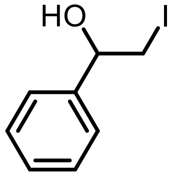

2-iodo-1-phenylethan-1-ol (*28'*). Orange oil, (36.5 mg, yield 49%). **^{1}H NMR** (400 MHz, $CDCl_3$) δ 7.49 – 7.30 (m, 5H), 4.87 (dd, J = 9.1, 3.4 Hz, 1H), 3.52 (ddd, J = 10.4, 3.7, 1.6 Hz, 1H), 3.43 (ddd, J = 10.2, 8.8, 1.2 Hz, 1H), 2.55 (bs, 1H). **^{13}C{^{1}H} NMR** (101 MHz, $CDCl_3$) δ 141.1, 128.7, 128.4, 125.8, 74.1, 15.4.

The NMR data closely match the ones previously reported in the literature [33]

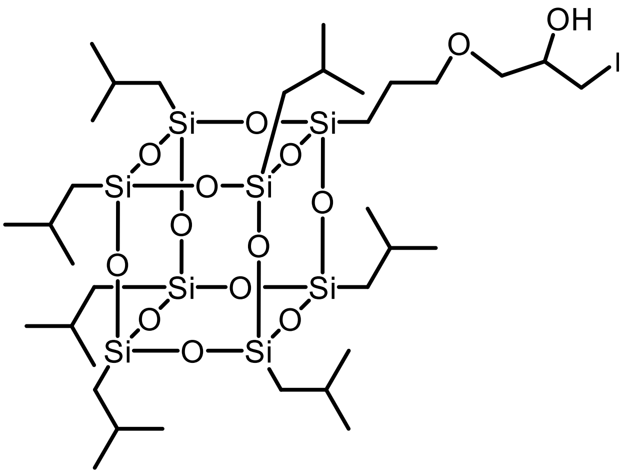

1-(3-(3,5,7,9,11,13,15-heptaisobutyl-2,4,6,8,10,12,14,16,17,18,19,20-dodecaoxa-1,3,5,7,9,11,13,15-octasilapentacyclo[9.5.1.13,9.15,15.17,13]icosan-1-yl)propoxy)-3-iodopropan-2-ol *(44')*. White solid, (139.7 mg, yield 44%); **m.p.** 118.5 – 120.4 °C. **^{1}H NMR** (400 MHz, $CDCl_3$) δ 3.78 (h, J = 5.5 Hz, 1H), 3.56 – 3.51 (m, 2H), 3.51 – 3.44 (m, 2H), 3.36 (dd, J = 10.2, 5.6 Hz, 1H), 3.29 (dd, J = 10.2, 5.8 Hz, 1H), 2.53 (d, J = 5.4 Hz, 1H), 1.88 (dp, J = 13.4, 6.7 Hz, 7H), 1.76 – 1.64 (m, 2H), 0.98 (dd, J = 6.6, 1.4 Hz, 42H), 0.63 (dd, J = 7.1, 3.3 Hz, 16H). **^{13}C{^{1}H} NMR** (101 MHz, $CDCl_3$) δ 73.6, 72.9, 69.9, 25.7, 23.9, 22.9, 22.5, 9.2, 8.3. **HRMS (ESI)** m/z calculated for $C_{34}H_{76}IO_{14}Si_8^+$ $[M+H]^+$: 1059.2429, found: 1059.2433.

8.6 Guanidine **G** and **[GH][HCO$_3$]**

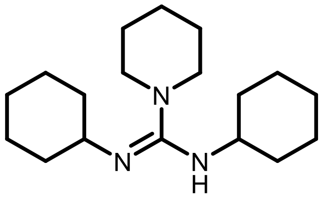

(Z)-N,N'-dicyclohexylpiperidine-1-carboximidamide *(G).* Pale yellow solid (847.4 mg, 97%). **^{1}H NMR** (400 MHz, CD$_3$OD_SPE) δ 3.26 (dt, J = 14.1, 4.7 Hz, 6H), 1.94 – 1.75 (m, 8H), 1.68 (pt, J = 10.2, 3.9 Hz, 8H), 1.37 (td, J = 9.1, 2.3 Hz, 8H), 1.23 (tt, J = 12.2, 3.3 Hz, 2H). **^{13}C{^{1}H} NMR** (101 MHz, CD$_3$OD_SPE) δ 158.0, 54.6, 49.1, 33.5, 25.5, 25.1, 25.0, 24.0.

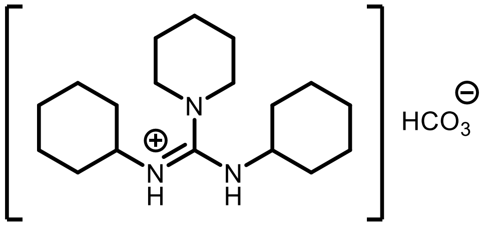

(Z)-N-((cyclohexylamino)(piperidin-1-yl)methylene)cyclohexanaminium bicarbonate *([GH]HCO$_3$).* White solid (529.9 mg, 99%). **^{1}H NMR** (400 MHz, CD$_3$OD_SPE) δ 3.33 – 3.22 (m, 7H), 1.91 – 1.78 (m, 8H), 1.72 – 1.67 (m, 8H), 1.38 (tt, J = 13.4, 6.2 Hz, 8H), 1.29 – 1.13 (m, 2H).**^{13}C{^{1}H} NMR** (101 MHz, CD$_3$OD_SPE) δ 159.9, 158.0, 54.6, 49.1, 33.6, 25.6, 25.1 (2C), 24.2.

9. References

1. García-Domínguez, P., Fehr, L., Rusconi, G. & Nevado, C. Palladium-catalyzed incorporation of atmospheric CO_2: efficient synthesis of functionalized oxazolidinones. *Chem Sci* **7**, 3914–3918 (2016).

2. Chiminelli, M. *et al.* Visible-Light Promoted Intramolecular *para* -Cycloadditions on Simple Aromatics. *Angew Chem Int Ed* **62**, (2023).

3. Puriņš, M. & Waser, J. Asymmetric Cyclopropanation and Epoxidation via a Catalytically Formed Chiral Auxiliary. *Angew Chem Int Ed* **61**, (2022).

4. Villo, P., Toom, L., Eriste, E. & Vares, L. Synthesis of Linear Aza and Thio Analogues of Acetogenins and Evaluation of Their Cytotoxicity. *Eur J Org Chem* **2013**, 6886–6899 (2013).

5. Li, S., Shi, Y., Li, P. & Xu, J. Nucleophilic Organic Base DABCO-Mediated Chemospecific Meinwald Rearrangement of Terminal Epoxides into Methyl Ketones. *J Org Chem* **84**, 4443–4450 (2019).

6. Dabas, S. *et al.* Modular synthesis of spirocyclic carbonates: unravelling the synergistic interplay of electronic and electrostatic sites on phenolic catalyst. *Green Chem* **26**, 2198–2206 (2024).

7. Beaver, M. G. & Jamison, T. F. Ni(II) Salts and 2-Propanol Effect Catalytic Reductive Coupling of Epoxides and Alkynes. *Org Lett* **13**, 4140–4143 (2011).

8. Costa, M., Chiusoli, G. P., Taffurelli, D. & Dalmonego, G. Superbase catalysis of oxazolidin-2-one ring formation from carbon dioxide and prop-2-yn-1-amines under homogeneous or heterogenous conditions. *J Chem Soc Perkin 1* 1541–1546 (1998) doi:10.1039/a800453f.

9. Aoyagi, N., Furusho, Y. & Endo, T. Cyclic amidine hydroiodide for the synthesis of cyclic carbonates and cyclic dithiocarbonates from carbon dioxide or carbon disulfide under mild conditions. *Tetrahedron* **75**, 130781 (2019).

10. Villiers, C., Dognon, J., Pollet, R., Thuéry, P. & Ephritikhine, M. An Isolated CO_2 Adduct of a Nitrogen Base: Crystal and Electronic Structures. *Angew Chem Int Ed* **49**, 3465–3468 (2010).

11. Cai, S.-F., Qiu, L.-Q., Huang, W.-B., Li, H.-R. & He, L.-N. Palladium-catalyzed carboxylative cyclization of propargylic amines with aryl iodides, CO_2 and CO under ambient pressure. *Chem Commun* **58**, 6332–6335 (2022).

12. Man, J. Y. H. & Au-Yeung, H. Y. Synthesis of a [6]rotaxane with singly threaded γ-cyclodextrins as a single stereoisomer. *Beilstein J Org Chem* **15**, 1829–1837 (2019).

13. Zhang, C.-H., Hu, T.-D., Zhai, Y.-T., Zhang, Y.-X. & Wu, Z.-L. Stepwise engineering of the pore environment within metal–organic frameworks for green conversion of CO_2 and propargylic amines. *Green Chem* **25**, 1938–1947 (2023).

14. Atilgan, A. *et al.* Near-IR-Triggered, Remote-Controlled Release of Metal Ions: A Novel Strategy for Caged Ions. *Angew Chem Int Ed* **53**, 10678–10681 (2014).

15. Zhang, Y. *et al.* Controllable encapsulation of silver nanoparticles by porous pyridine-based covalent organic frameworks for efficient CO_2 conversion using propargylic amines. *Green Chem* **24**, 930–940 (2022).

16. Chou, C. *et al.* Synthesis and Photophysical Characterization of 2,3-Dihydroquinolin-4-imines: New Fluorophores with Color-Tailored Emission. *Chem Eur J* **24**, 1112–1120 (2018).

17. Andreev, V. P., Sobolev, P. S., Zaitsev, D. O., Remizova, L. A. & Tafeenko, V. A. Coordination of secondary and tertiary amines to zinc tetraphenylporphyrin. *Russ J Gen Chem* **84**, 1979–1988 (2014).

18. Das, A., Buzzetti, L., Puriņš, M. & Waser, J. Palladium-Catalyzed *trans* -Hydroalkoxylation: Counterintuitive Use of an Aryl Iodide Additive to Promote C–H Bond Formation. *ACS Catal* **12**, 7565–7570 (2022).

19. Murphy, A., Pace, A. & Stack, T. D. P. Ligand and pH Influence on Manganese-Mediated Peracetic Acid Epoxidation of Terminal Olefins. *Org Lett* **6**, 3119–3122 (2004).

20. Li, S., Chen, X. & Xu, J. Microwave-assisted copper-catalyzed stereoselective ring expansion of three-membered heterocycles with α-diazo-β-dicarbonyl compounds. *Tetrahedron* **74**, 1613–1620 (2018).

21. Rintjema, J. *et al.* Substrate-Controlled Product Divergence: Conversion of CO_2 into Heterocyclic Products. *Angew Chem Int Ed* **55**, 3972–3976 (2016).

22. Pan, Y.-Z. *et al.* Electrochemically Mediated Carboxylative Cyclization of Allylic/Homoallylic Amines with CO_2 at Ambient Pressure. *Org Lett* **24**, 8239–8243 (2022).

23. Cao, C. *et al.* Highly Efficient Conversion of Propargylic Amines and CO_2 Catalyzed by Noble-Metal-Free [Zn_{116}] Nanocages. *Angew Chem Int Ed* **59**, 8586–8593 (2020).

24. Paisuwan, W., Chantra, T., Rashatasakhon, P., Sukwattanasinitt, M. & Ajavakom, A. Direct synthesis of oxazolidin-2-ones from tert -butyl allylcarbamate via halo-induced cyclisation. *Tetrahedron* **73**, 3363–3367 (2017).

25. Jin, H. *et al.* Copper-catalyzed cascade reactions of N-(2-bromoallyl)amines with KHCO3 as the C1 source: an efficient process for the synthesis of oxazolidin-2-ones. *RSC Adv* **4**, 26990 (2014).

26. Song, Q. & He, L. Robust Silver(I) Catalyst for the Carboxylative Cyclization of Propargylic Alcohols with Carbon Dioxide under Ambient Conditions. *Adv Synth Catal* **358**, 1251–1258 (2016).

27. Pearson, D. M., Conley, N. R. & Waymouth, R. M. Palladium-Catalyzed Carbonylation of Diols to Cyclic Carbonates. *Adv Synth Catal* **353**, 3007–3013 (2011).

28. Castro-Osma, J. A., North, M. & Wu, X. Synthesis of Cyclic Carbonates Catalysed by Chromium and Aluminium Salphen Complexes. *Chem Eur J* **22**, 2100–2107 (2016).

29. Liu, X. *et al.* Cooperative calcium-based catalysis with 1,8-diazabicyclo[5.4.0]-undec-7-ene for the cycloaddition of epoxides with CO_2 at atmospheric pressure. *Green Chem* **18**, 2871–2876 (2016).

30. Whiteoak, C. J., Martin, E., Belmonte, M. M., Benet-Buchholz, J. & Kleij, A. W. An Efficient Iron Catalyst for the Synthesis of Five- and Six-Membered Organic Carbonates under Mild Conditions. *Adv Synth Catal* **354**, 469–476 (2012).

31. Whiteoak, C. J. *et al.* A Powerful Aluminum Catalyst for the Synthesis of Highly Functional Organic Carbonates. *J Am Chem Soc* **135**, 1228–1231 (2013).

32. Aoyagi, N., Furusho, Y. & Endo, T. Effective synthesis of cyclic carbonates from carbon dioxide and epoxides by phosphonium iodides as catalysts in alcoholic solvents. *Tetrahedron Lett* **54**, 7031–7034 (2013).

33. Baussière, F. & Haugland, M. M. Radical Group Transfer of Vinyl and Alkynyl Silanes Driven by Photoredox Catalysis. *J Org Chem* **88**, 12451–12463 (2023).

10. Author Contributions

Conceptualization: N.D. and R.M. Methodology: F.M., A.A., A.M.C. Investigation: L.R., L.C., F.P. Validation: A.P., D.A.C., G.M., E.M., L.C., R.M. Writing – original draft: F.M. Writing – review & editing: N.D., L.C., R.M., G.M. Funding acquisition: N.D. Supervision: N.D.

11. Copy of NMR Spectra

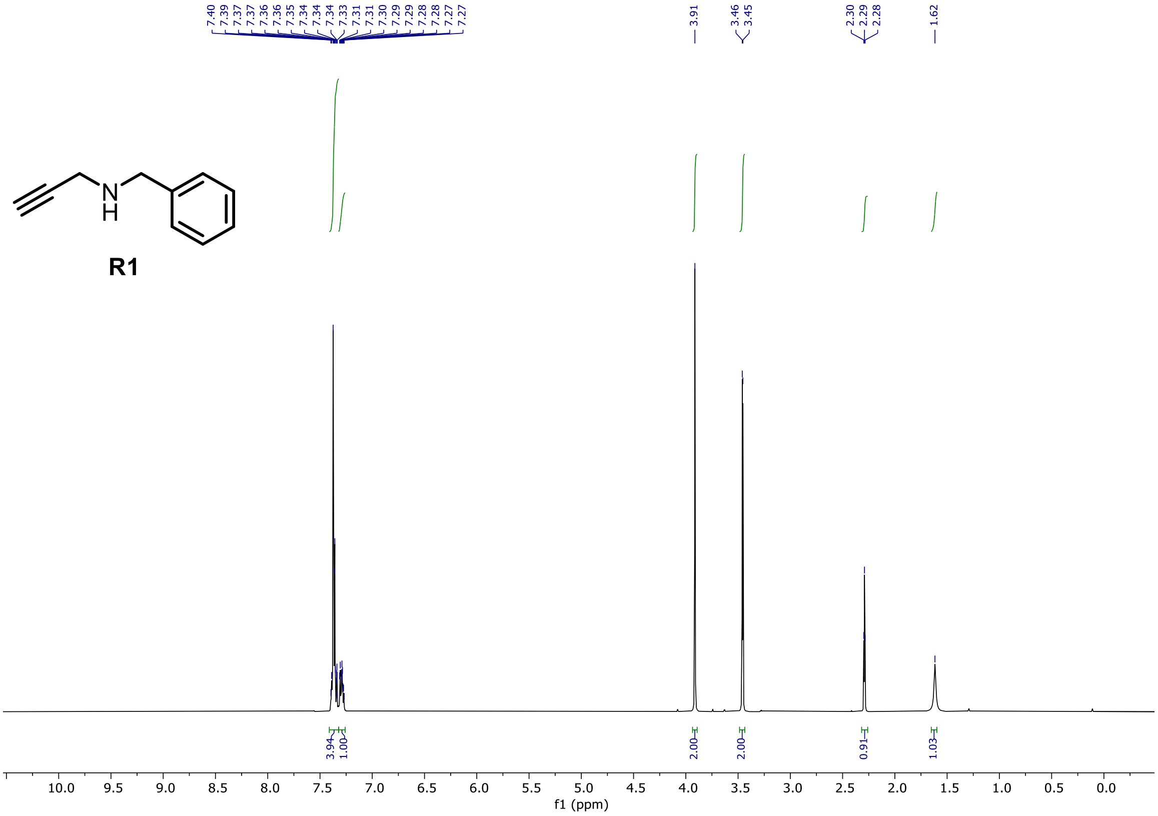

^{1}H NMR spectrum of compound **R1** (400 MHz, $CDCl_3$)

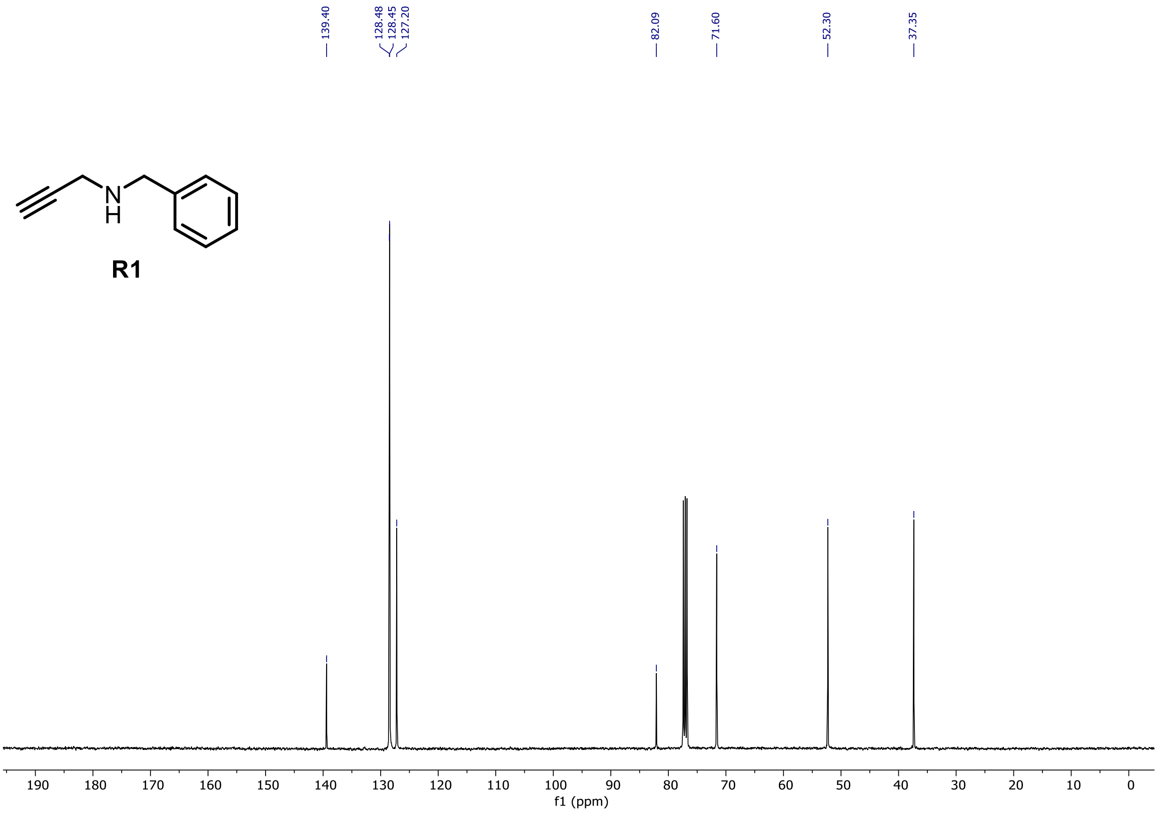

$^{13}C\{^1H\}$ NMR spectrum of compound **R1** (101 MHz, $CDCl_3$)

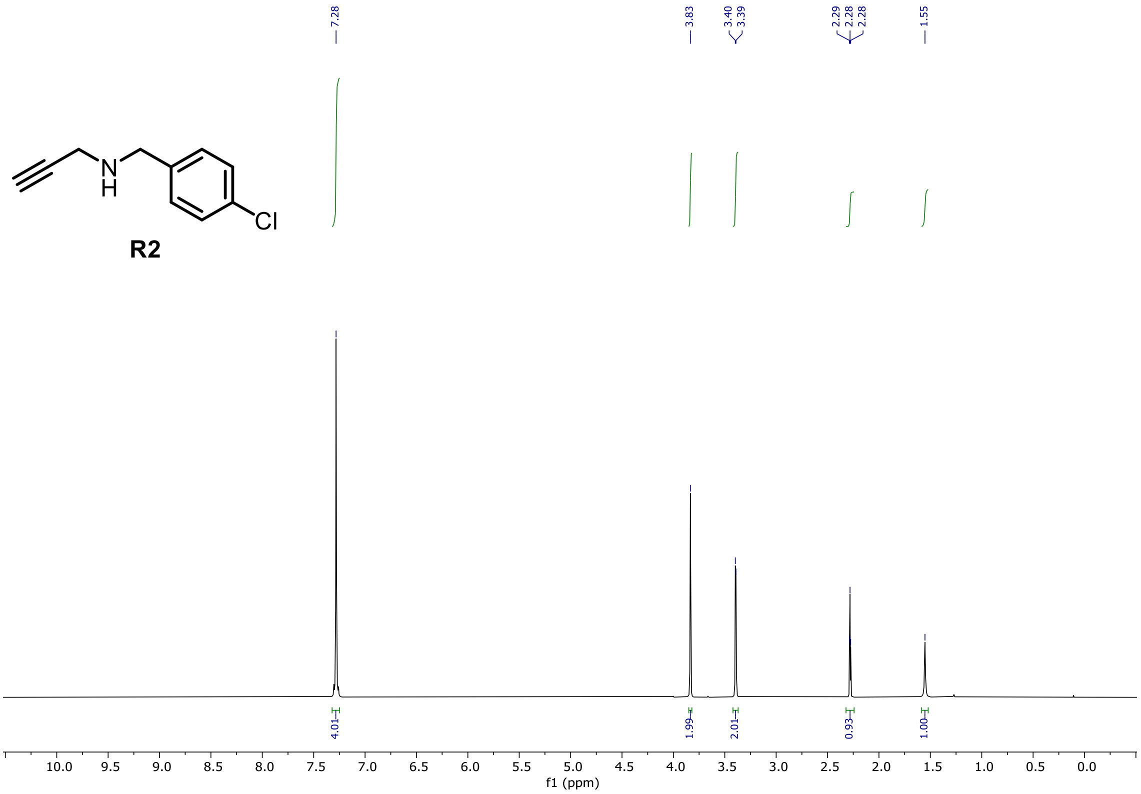

^1H NMR spectrum of compound **R2** (400 MHz, $CDCl_3$)

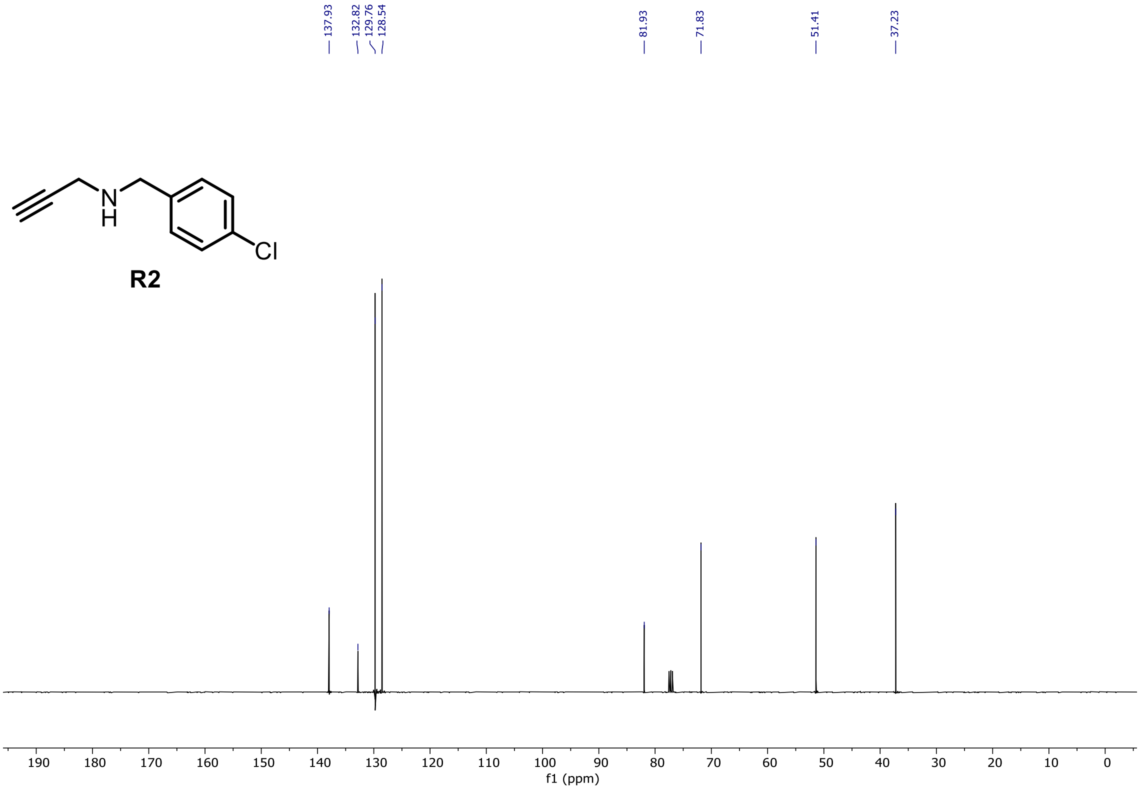

$^{13}C\{^1H\}$ NMR spectrum of compound **R2** (101 MHz, $CDCl_3$)

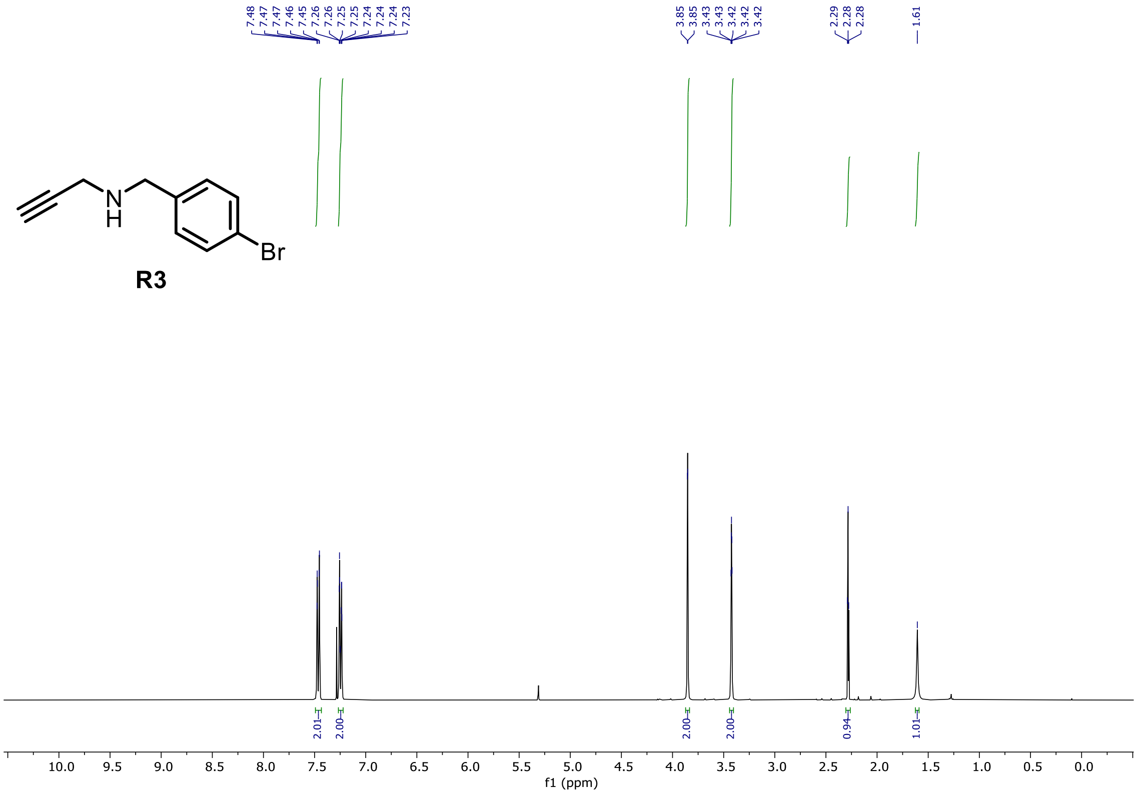

^{1}H NMR spectrum of compound **R3** (400 MHz, $CDCl_3$)

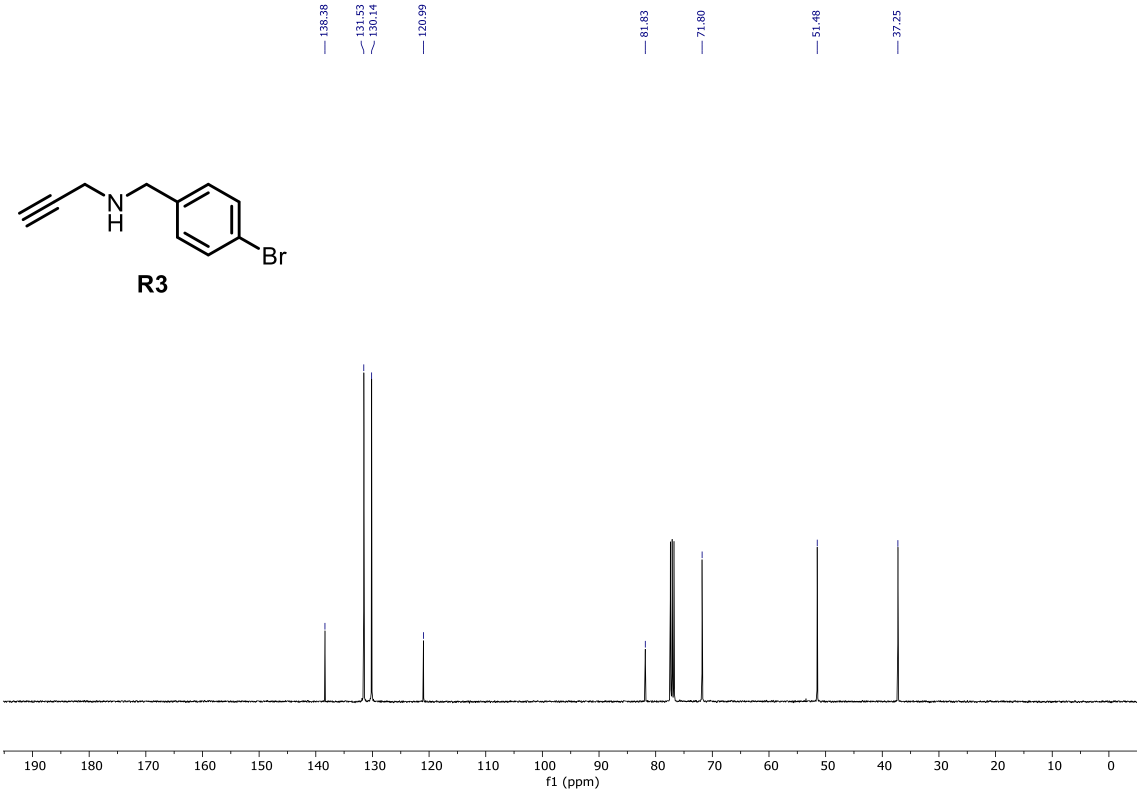

^{13}C{^{1}H} NMR spectrum of compound **R3** (101 MHz, $CDCl_3$)

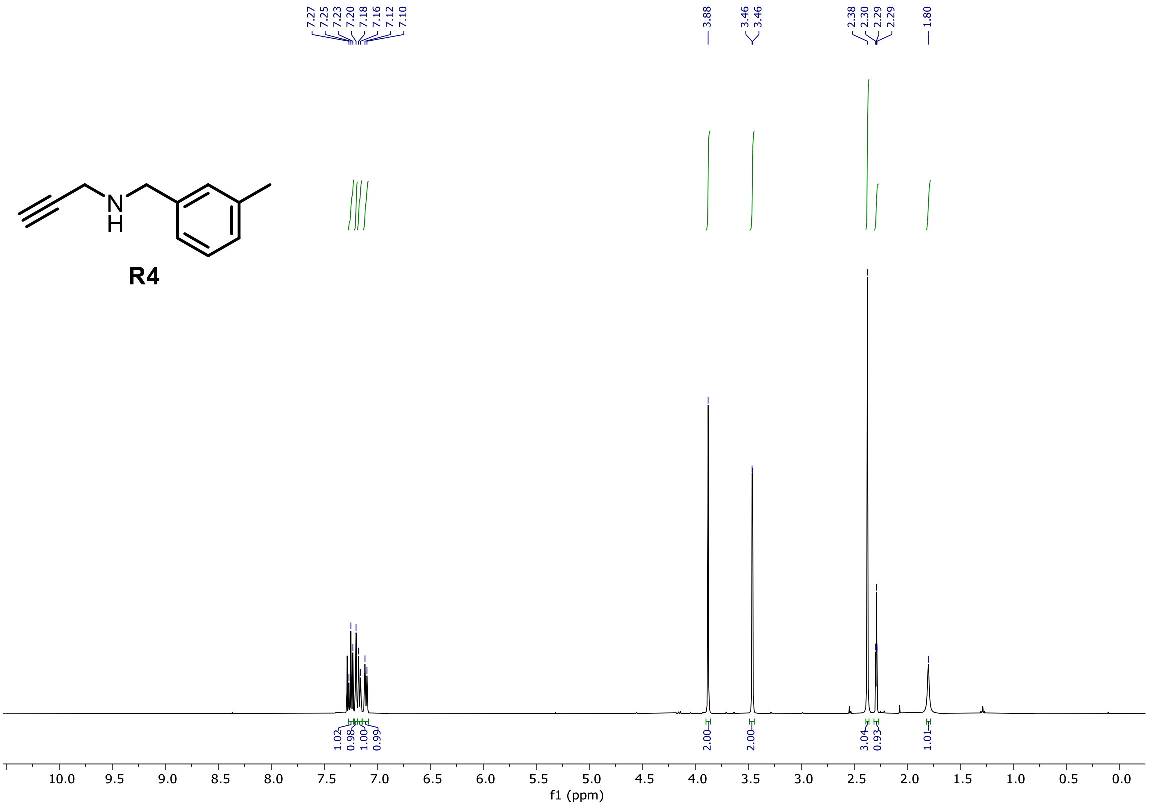

^{1}H NMR spectrum of compound **R4** (400 MHz, $CDCl_3$)

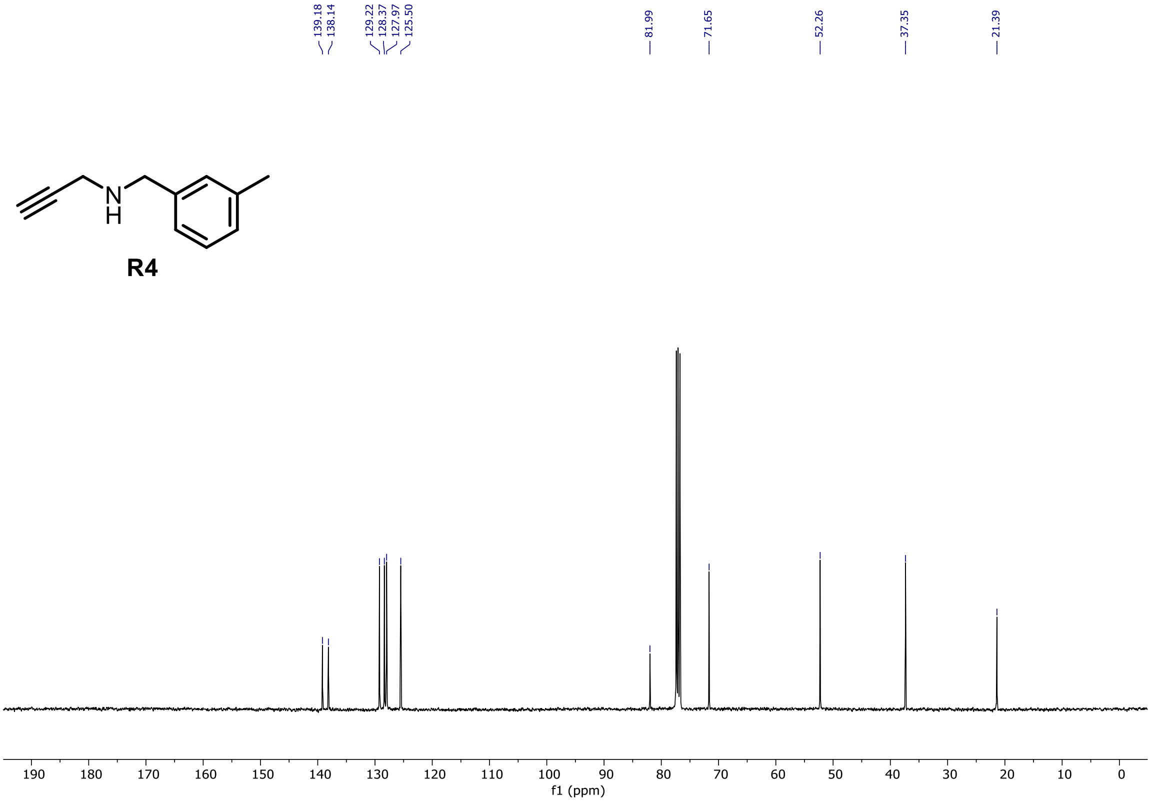

$^{13}C\{^1H\}$ NMR spectrum of compound **R4** (101 MHz, $CDCl_3$)

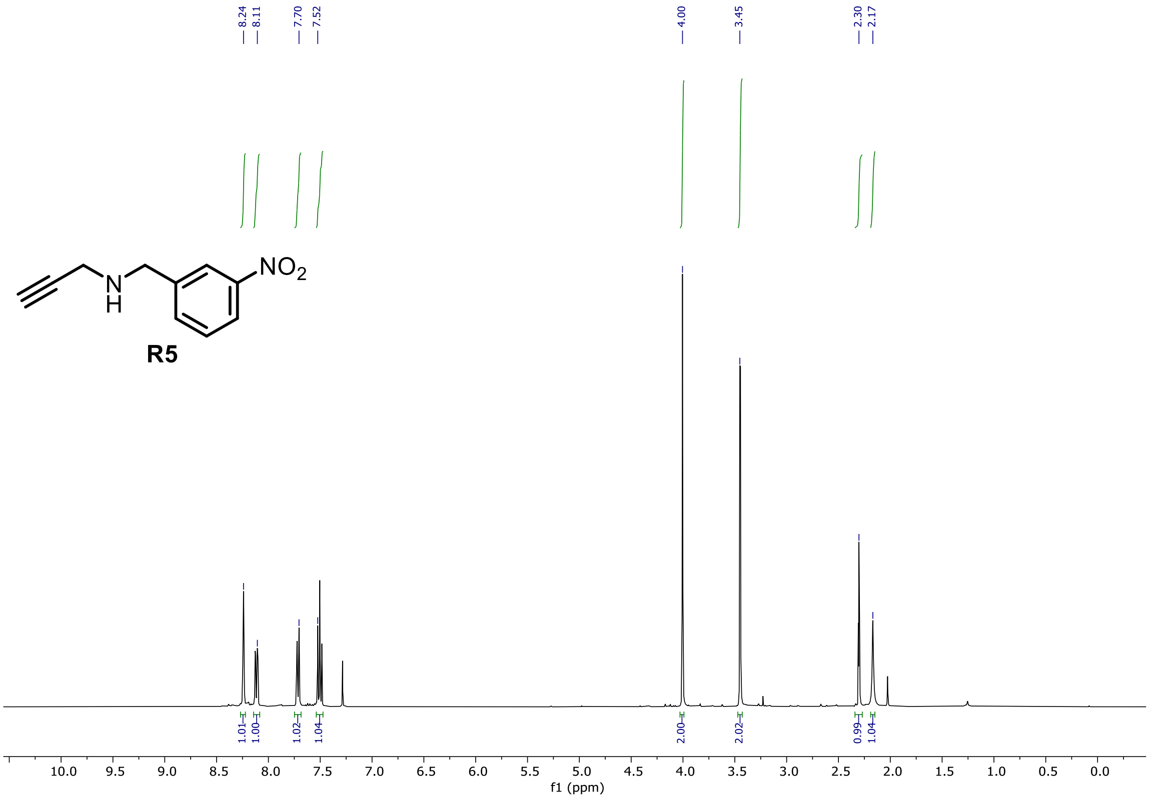

^{1}H NMR spectrum of compound **R5** (400 MHz, $CDCl_3$)

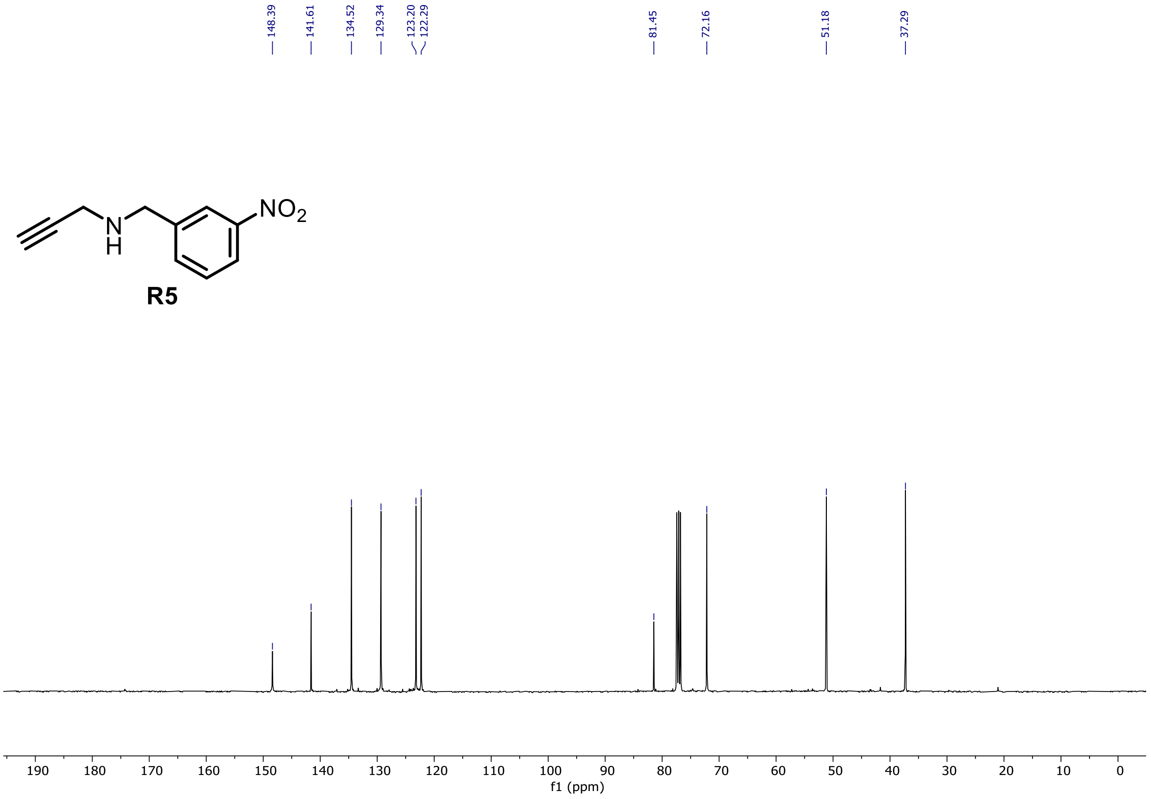

^{13}C{^{1}H} NMR spectrum of compound **R5** (101 MHz, $CDCl_3$)

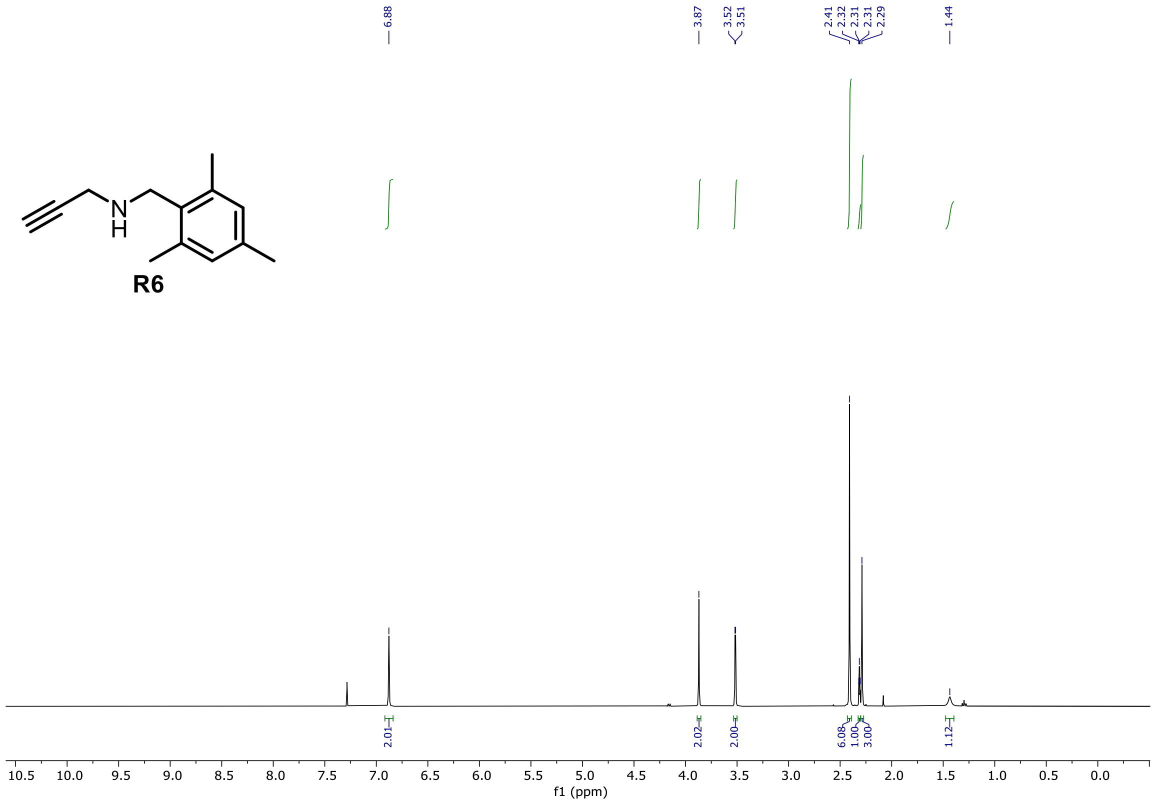

^{1}H NMR spectrum of compound **R6** (400 MHz, $CDCl_3$)

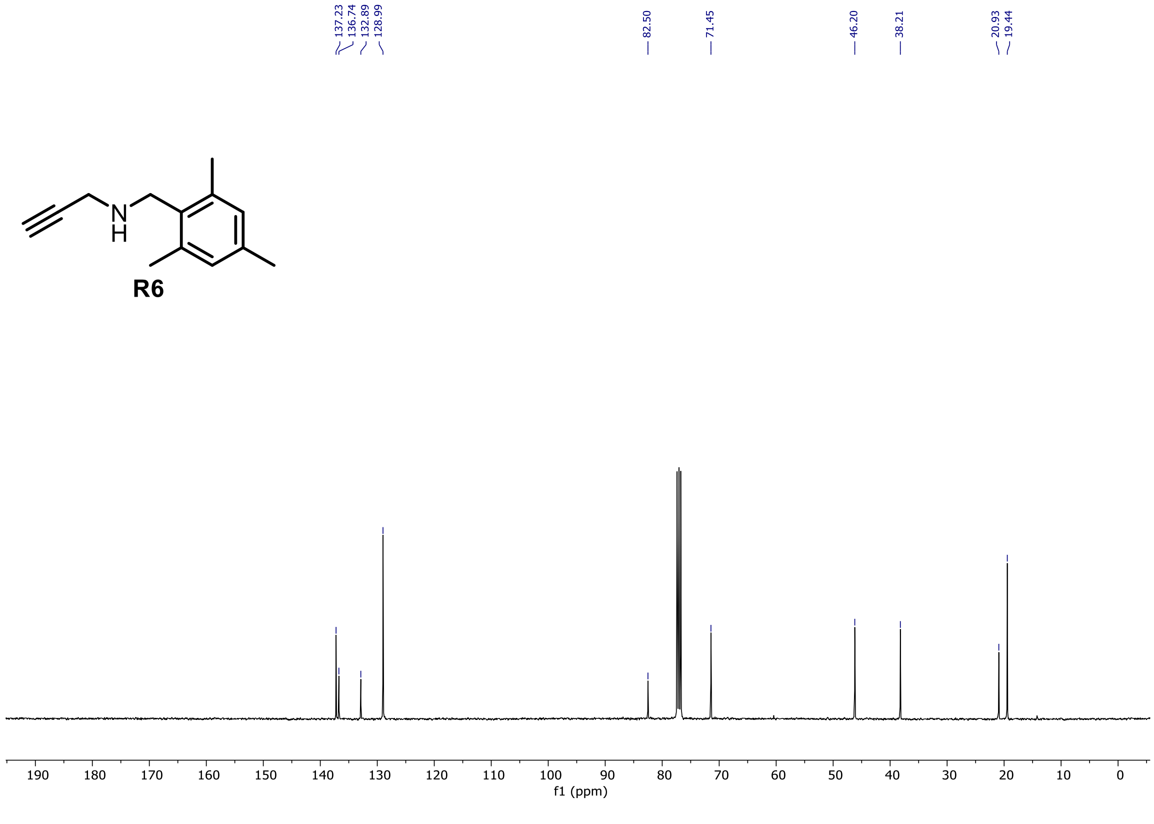

$^{13}C\{^{1}H\}$ NMR spectrum of compound **R6** (101 MHz, $CDCl_3$)

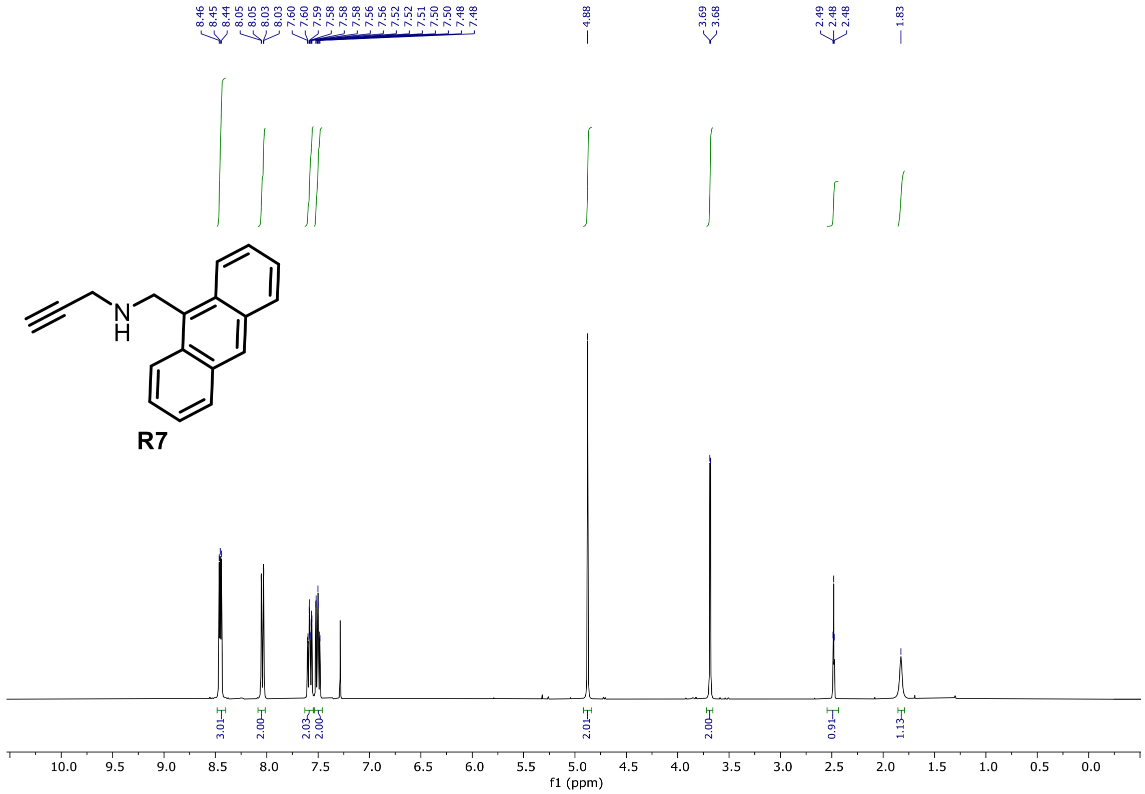

^{1}H NMR spectrum of compound **R7** (400 MHz, $CDCl_3$)

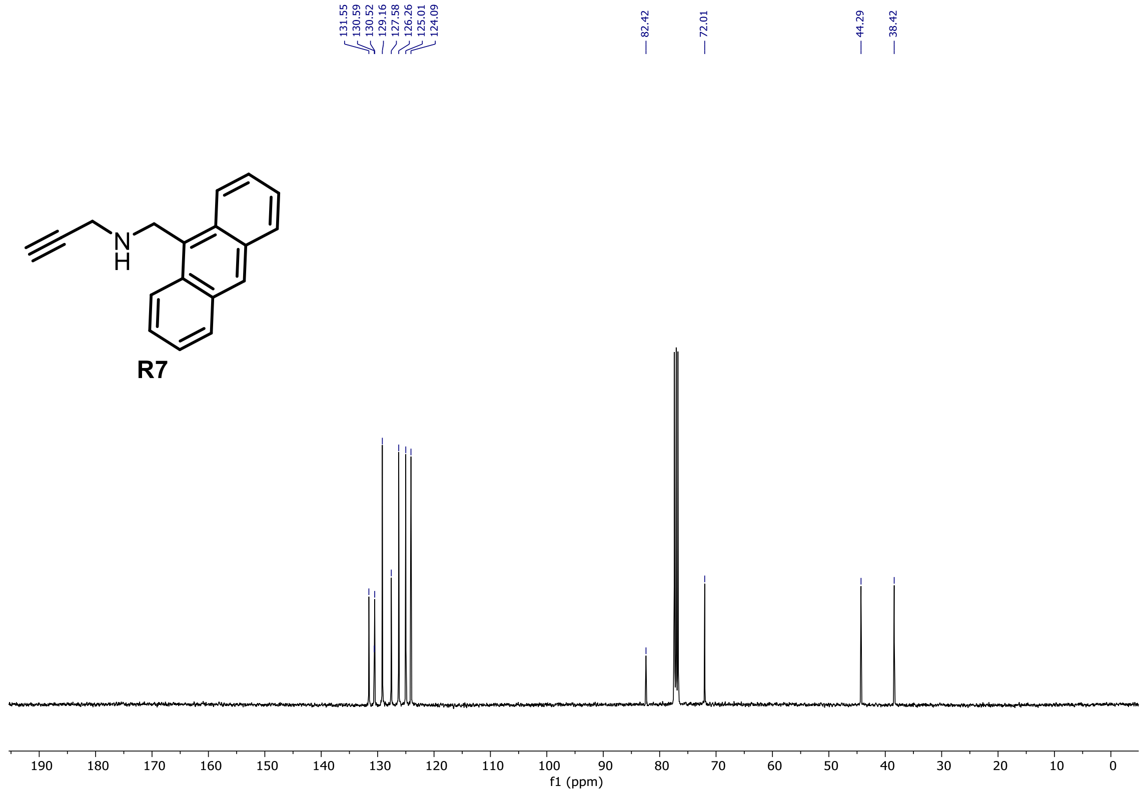

$^{13}C\{^{1}H\}$ NMR spectrum of compound **R7** (101 MHz, $CDCl_3$)

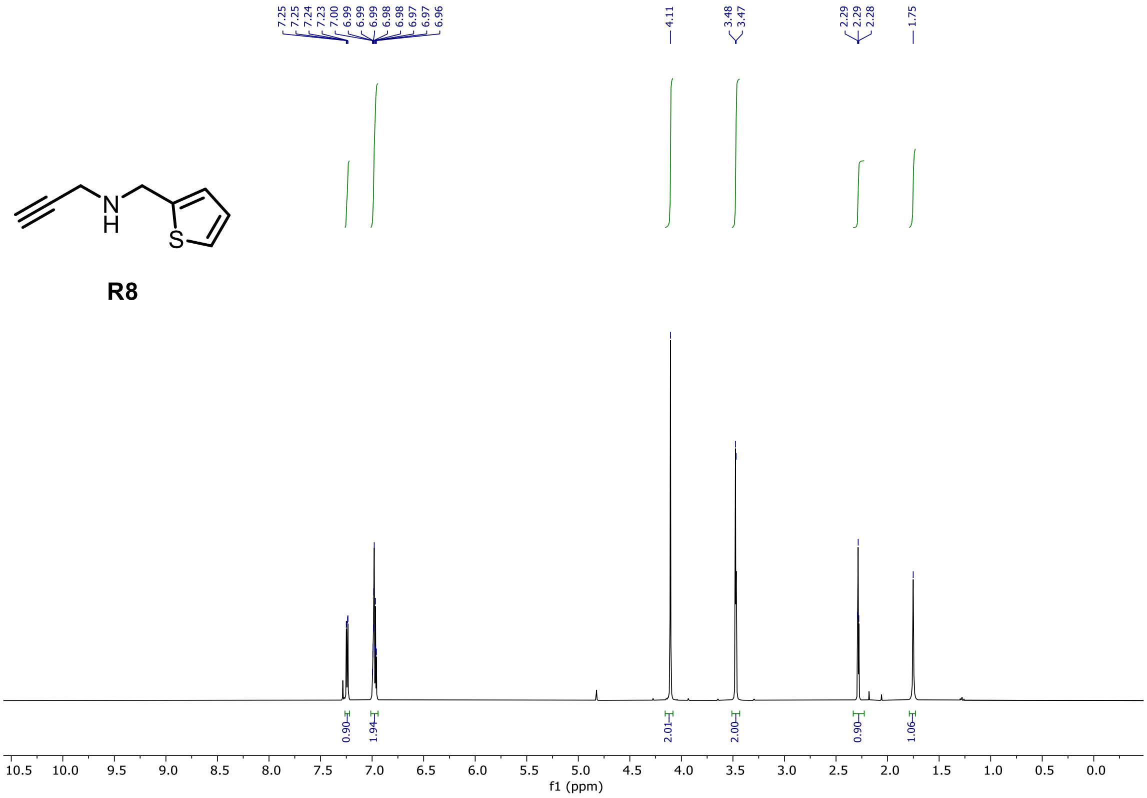

^{1}H NMR spectrum of compound **R8** (400 MHz, $CDCl_3$)

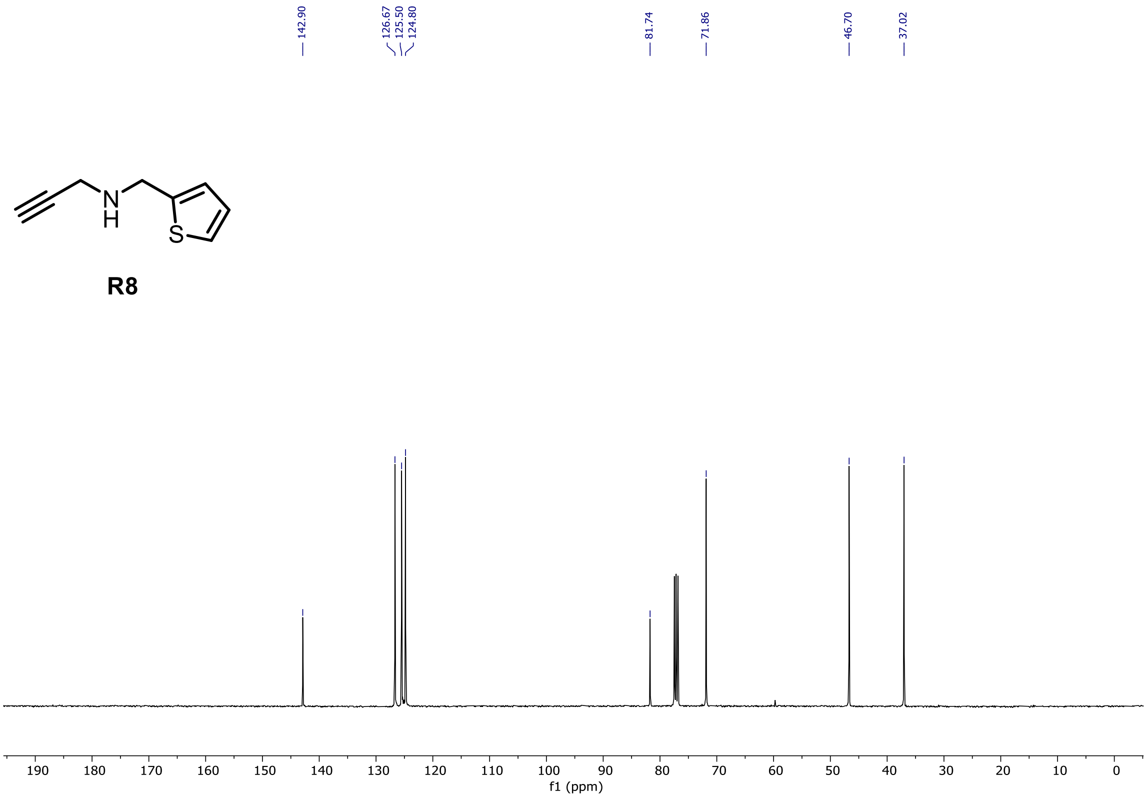

$^{13}C\{^{1}H\}$ NMR spectrum of compound **R8** (101 MHz, $CDCl_3$)

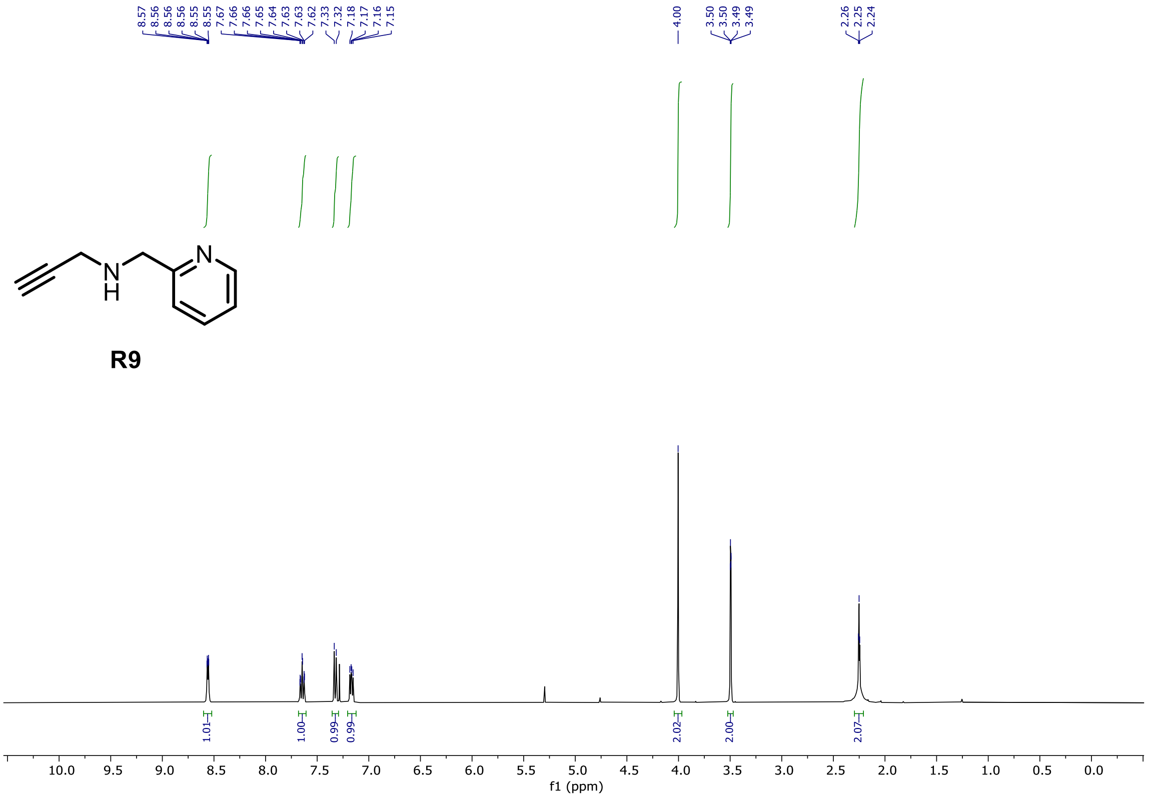

R9

^{1}H NMR spectrum of compound **R9** (400 MHz, $CDCl_3$)

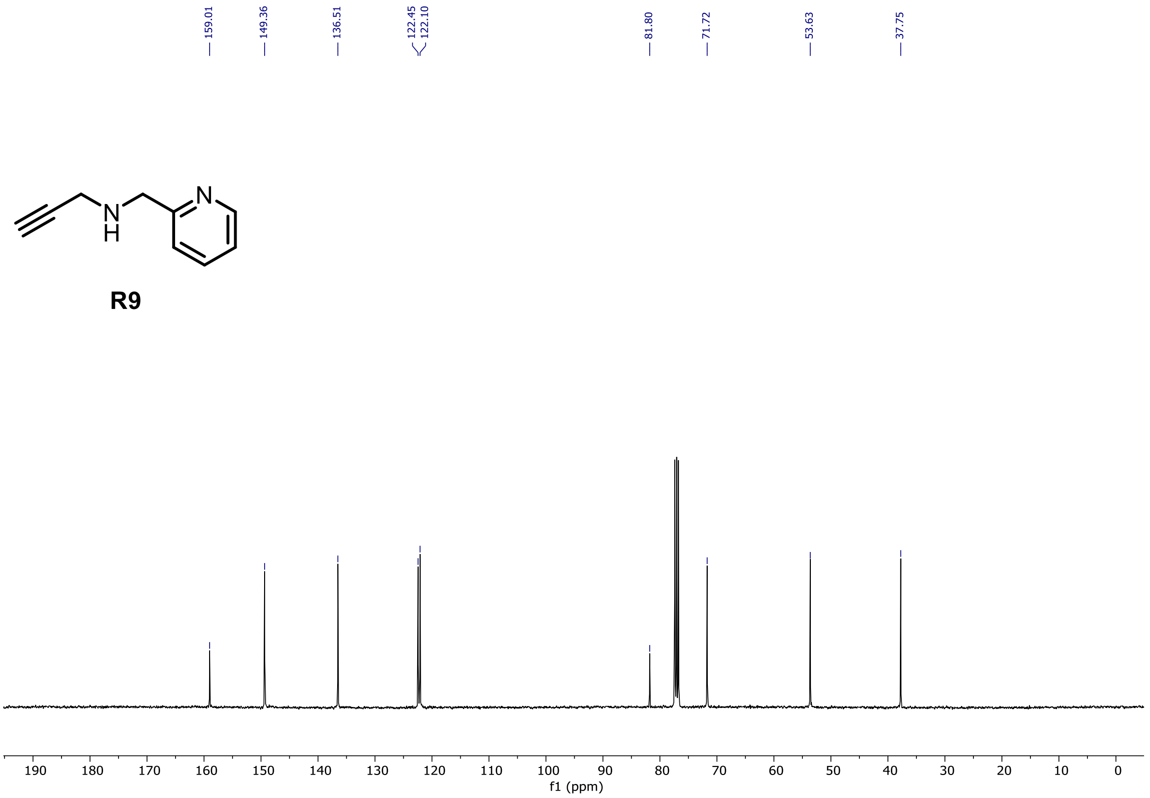

R9

^{13}C{^{1}H} NMR spectrum of compound **R9** (101 MHz, $CDCl_3$)

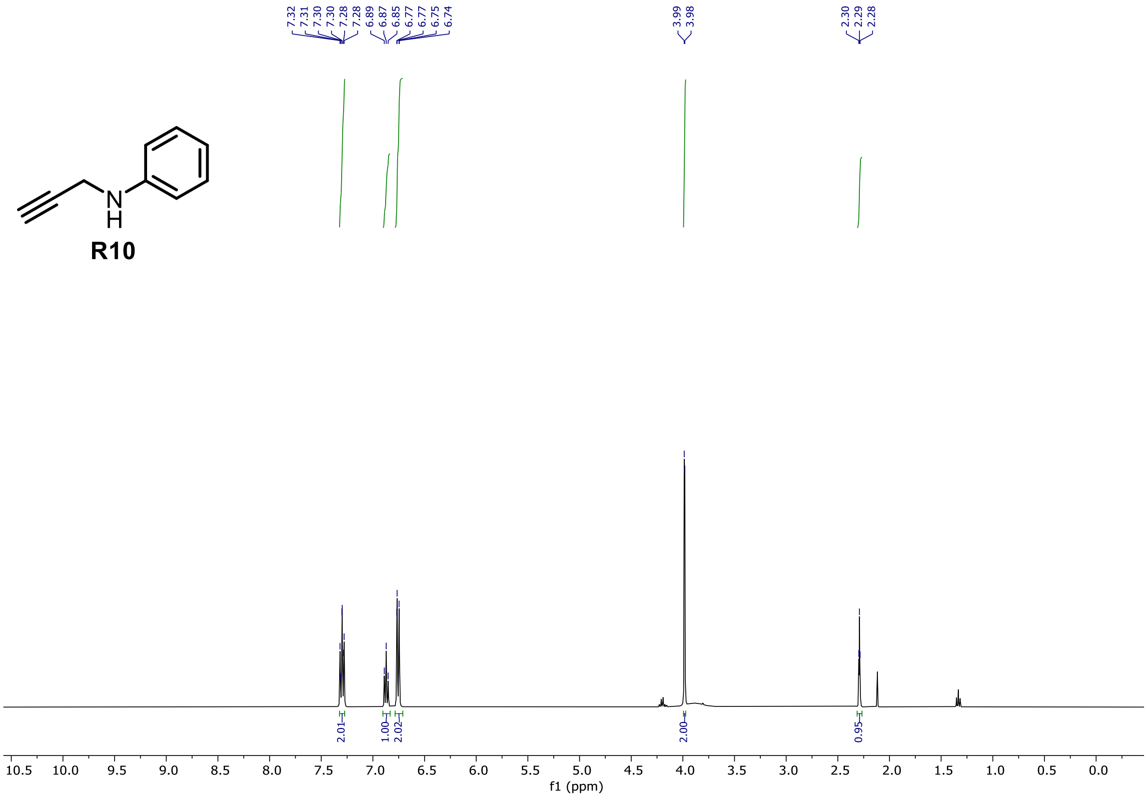

^{1}H NMR spectrum of compound **R10** (400 MHz, $CDCl_3$)

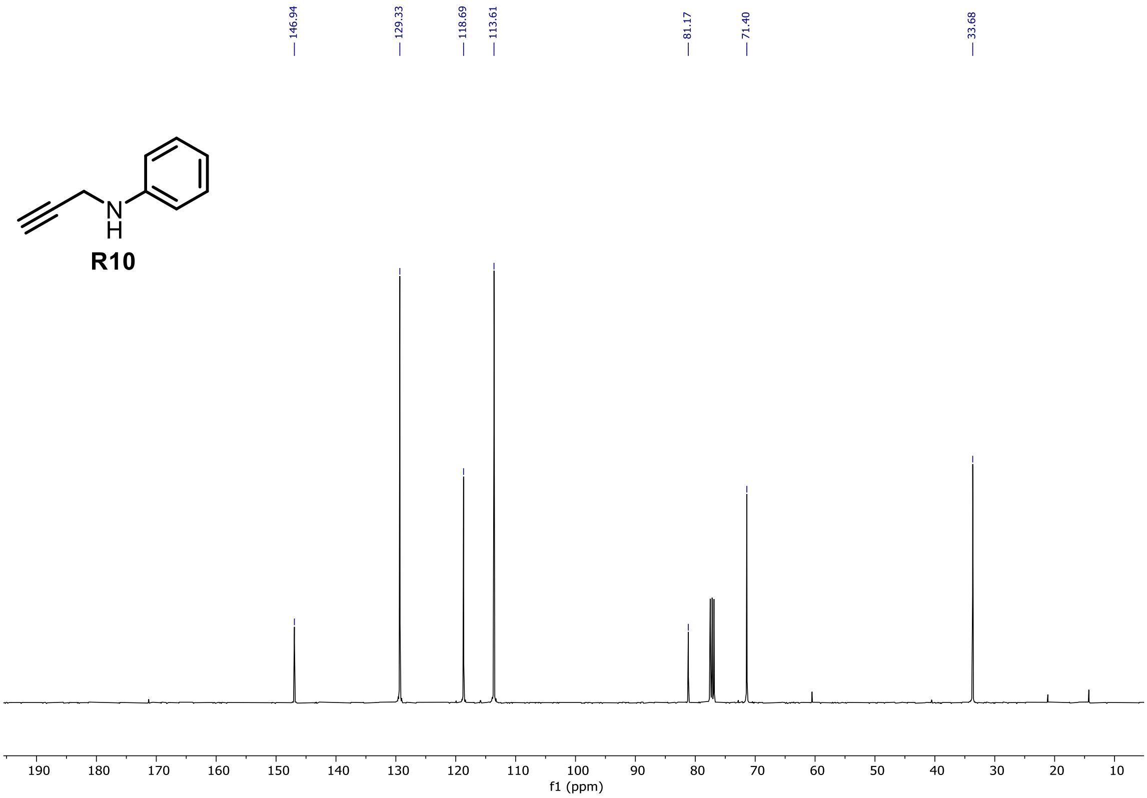

$^{13}C\{^{1}H\}$ NMR spectrum of compound **R10** (101 MHz, $CDCl_3$)

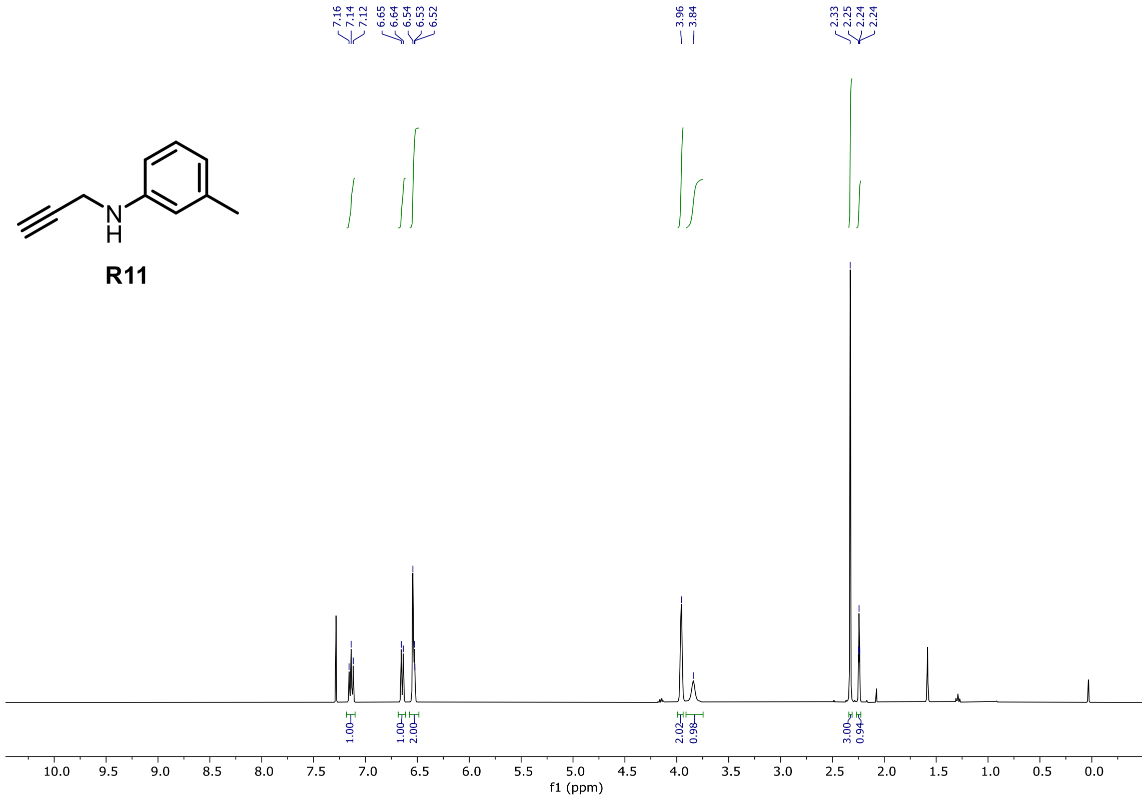

^{1}H NMR spectrum of compound **R11** (400 MHz, $CDCl_3$)

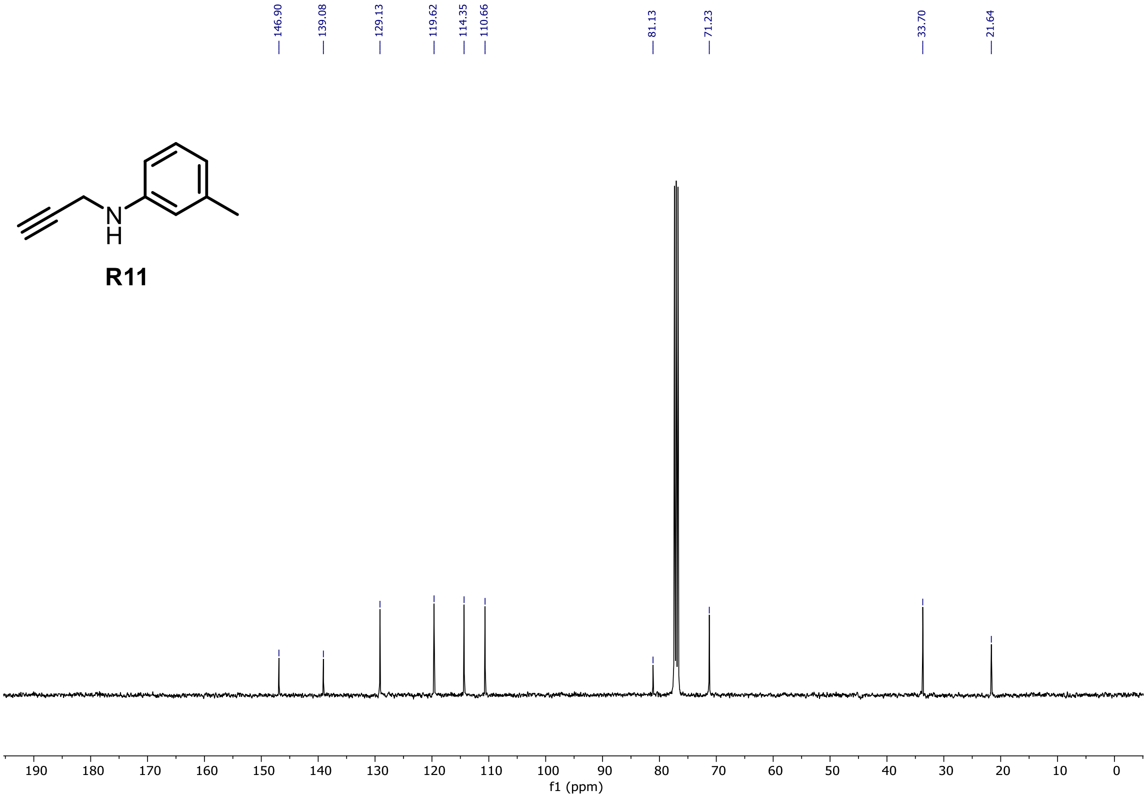

$^{13}C\{^{1}H\}$ NMR spectrum of compound **R11** (101 MHz, $CDCl_3$)

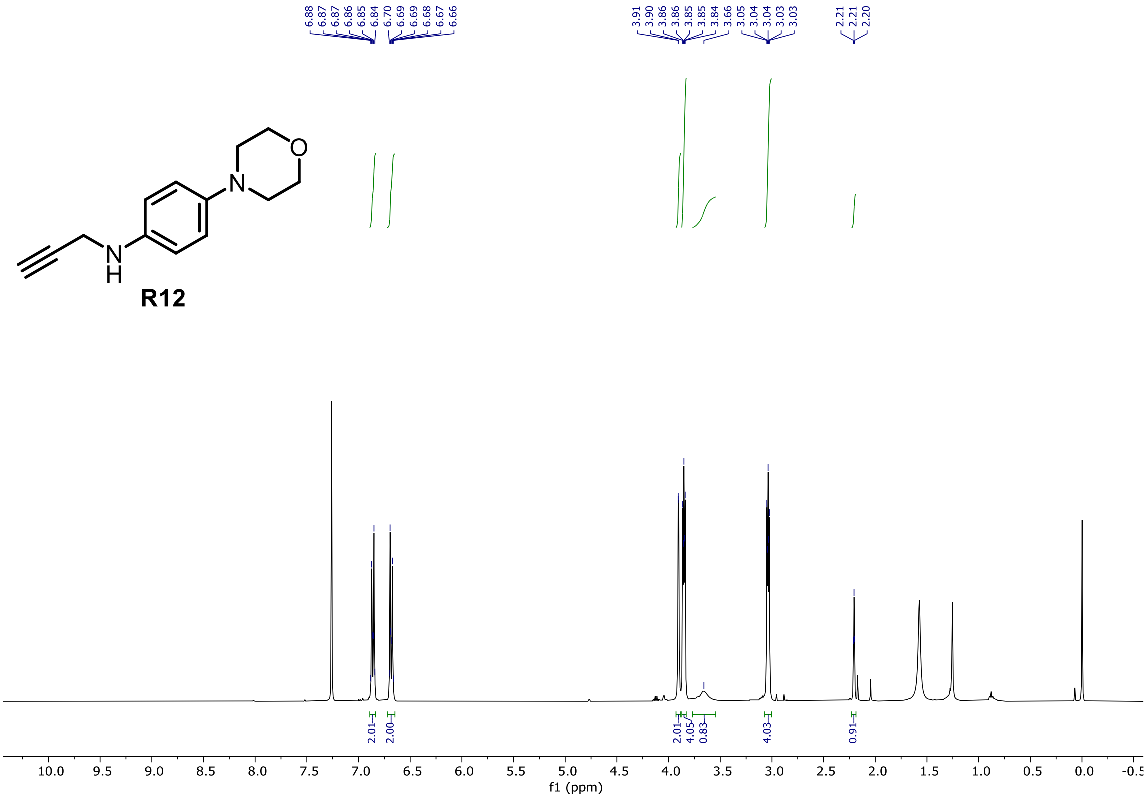

^1H NMR spectrum of compound **R12** (400 MHz, $CDCl_3$)

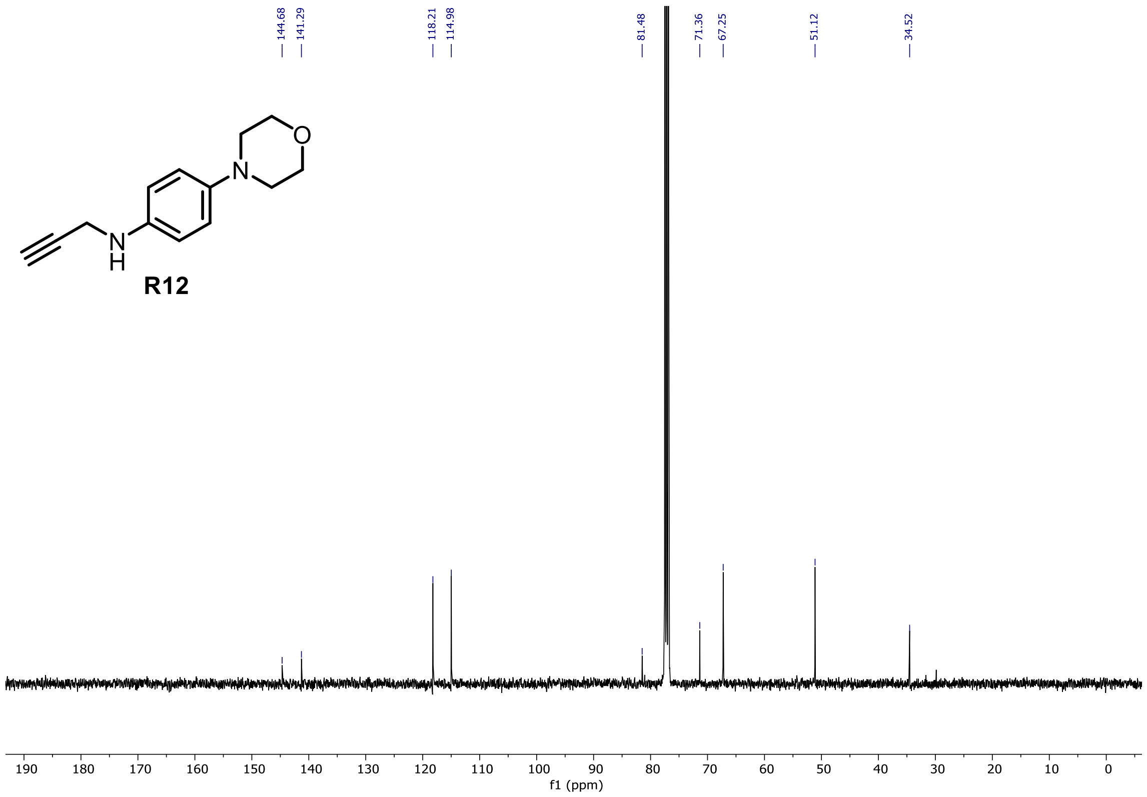

$^{13}C\{^1H\}$ NMR spectrum of compound **R12** (101 MHz, $CDCl_3$)

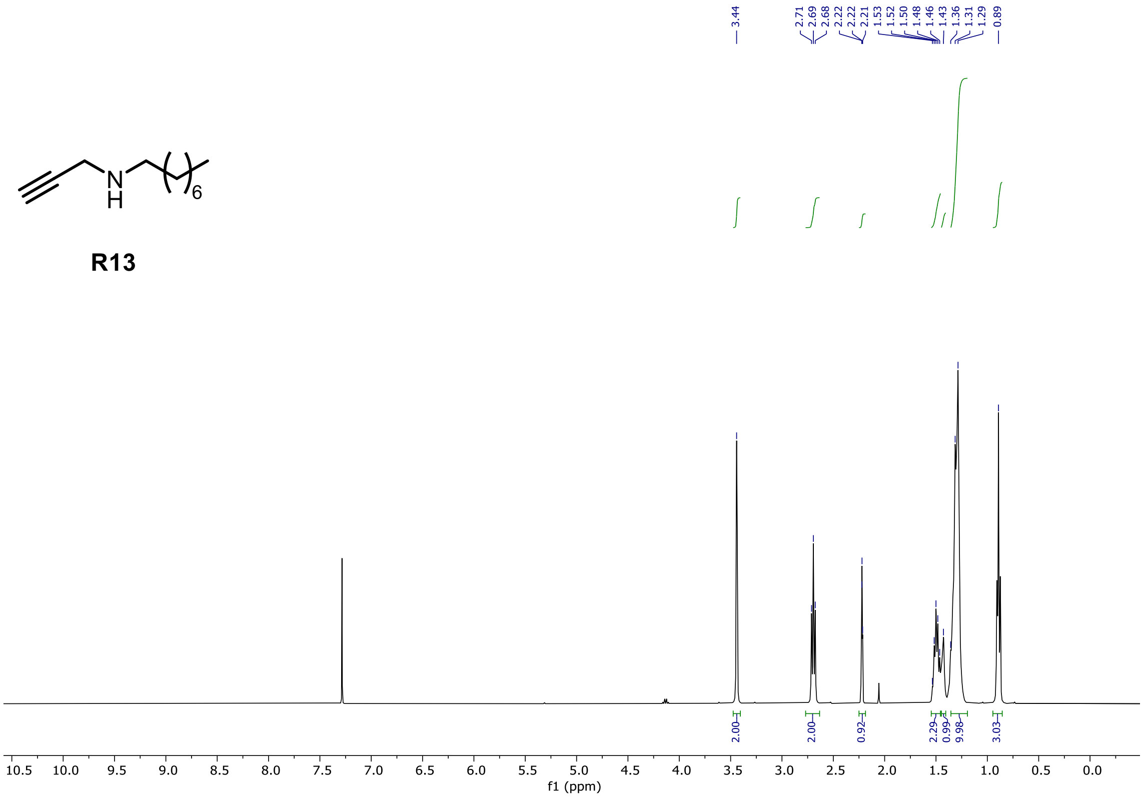

^{1}H NMR spectrum of compound **R13** (400 MHz, $CDCl_3$)

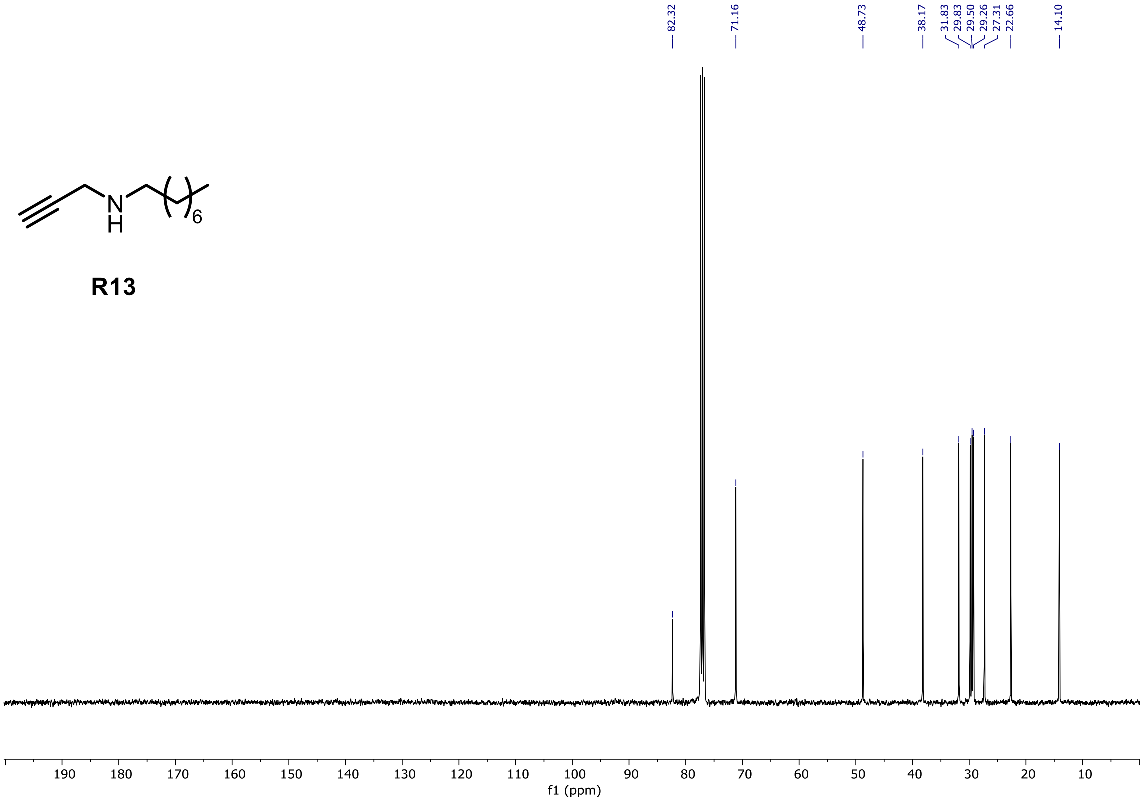

$^{13}C\{^{1}H\}$ NMR spectrum of compound **R13** (101 MHz, $CDCl_3$)

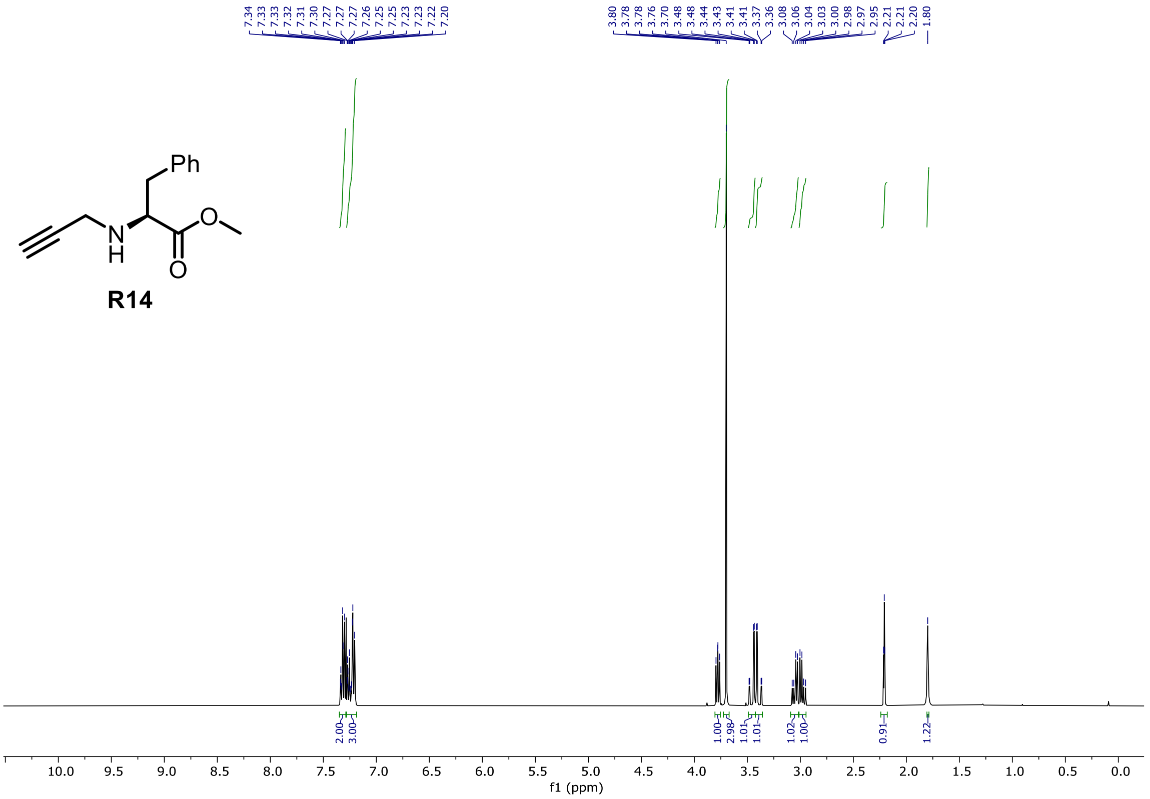

^{1}H NMR spectrum of compound **R14** (400 MHz, $CDCl_3$)

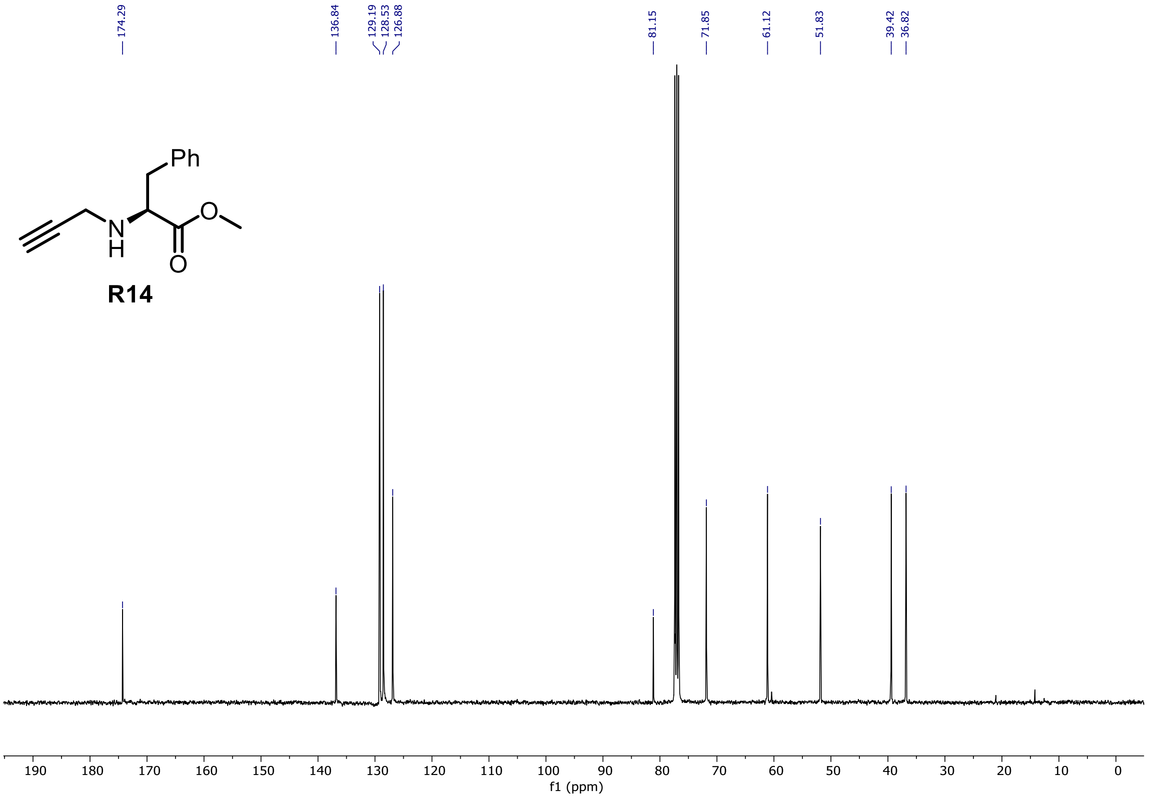

$^{13}C\{^{1}H\}$ NMR spectrum of compound **R14** (101 MHz, $CDCl_3$)

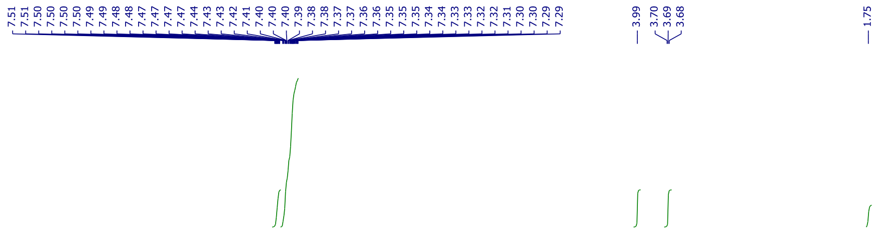

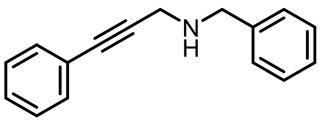

R15

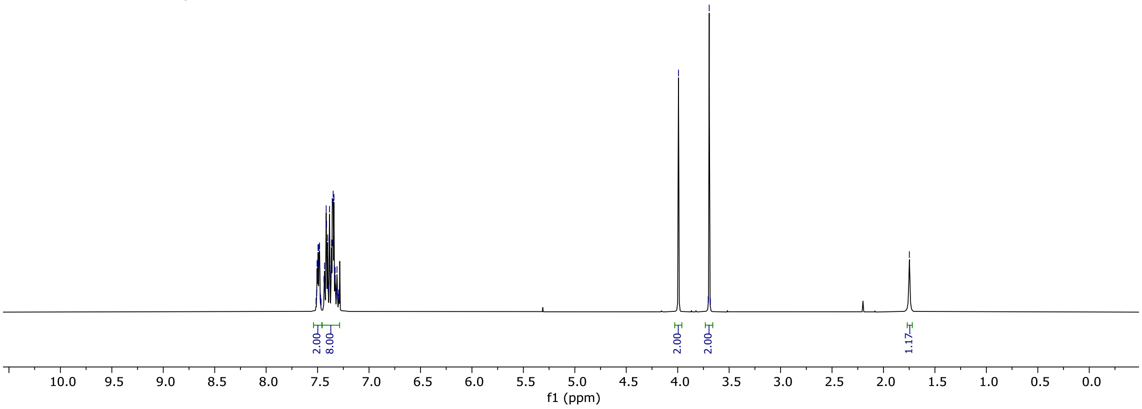

^{1}H NMR spectrum of compound **R15** (400 MHz, $CDCl_3$)

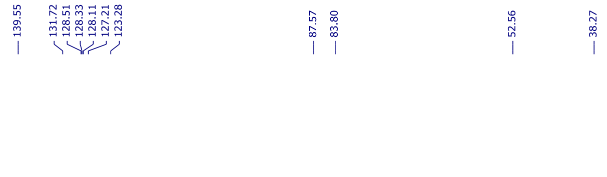

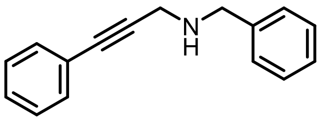

R15

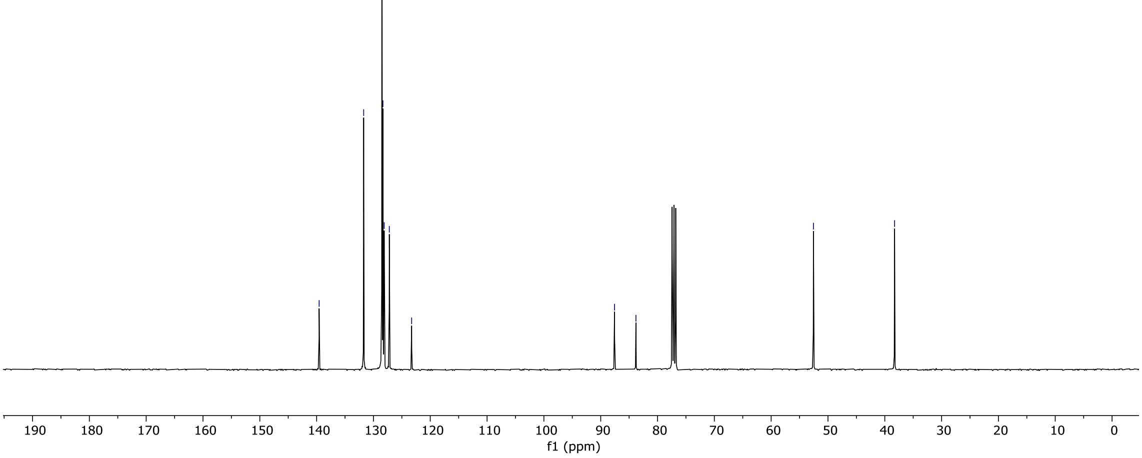

$^{13}C\{^1H\}$ NMR spectrum of compound **R15** (101 MHz, $CDCl_3$)

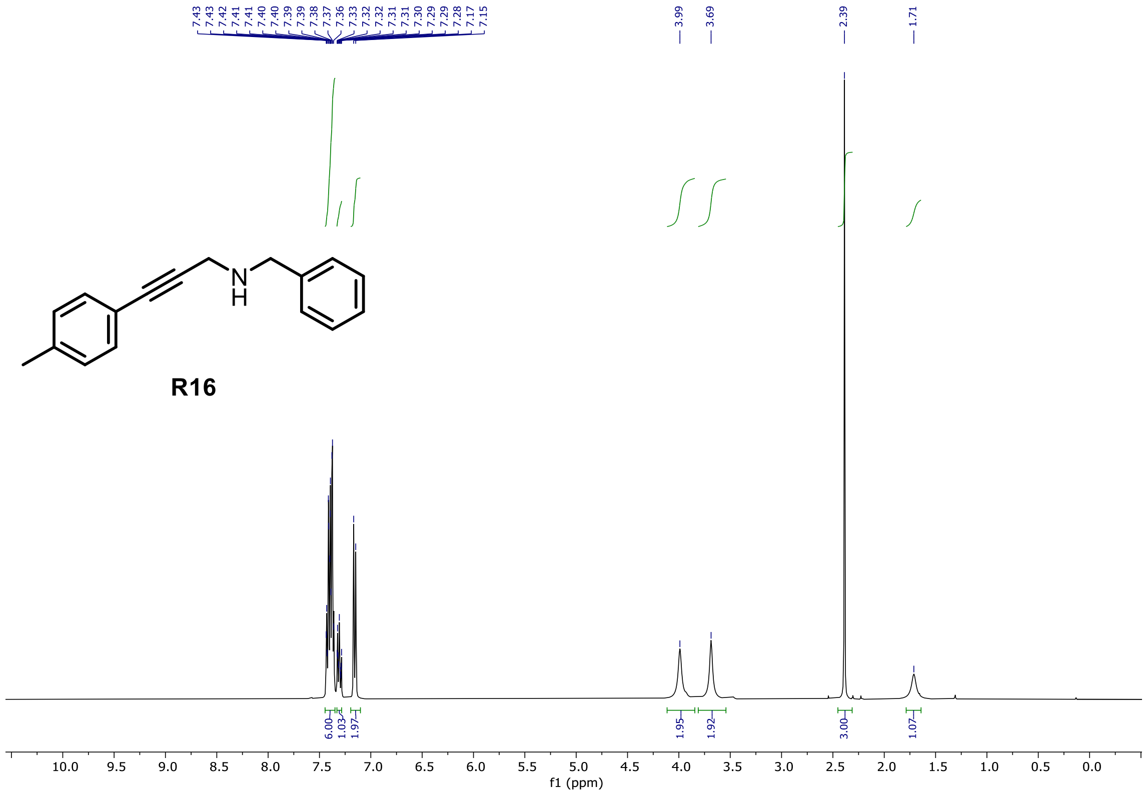

^{1}H NMR spectrum of compound **R16** (400 MHz, $CDCl_3$)

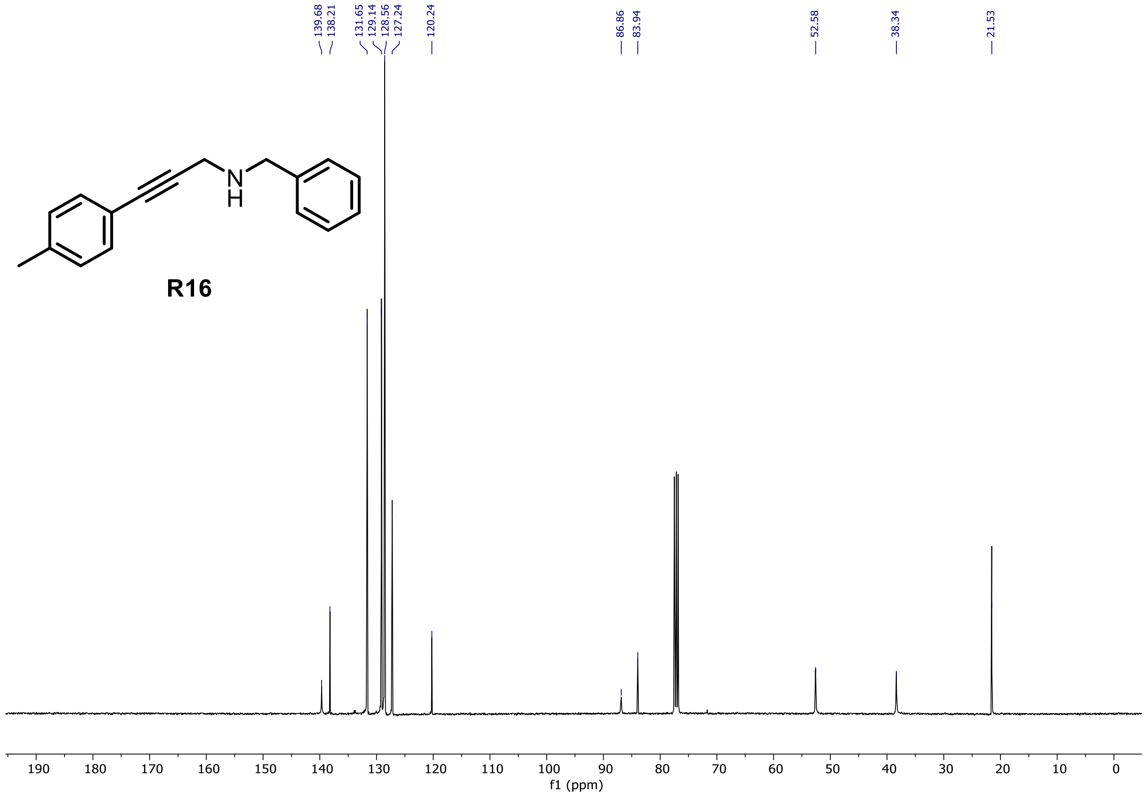

^{13}C{^{1}H} NMR spectrum of compound **R16** (101 MHz, $CDCl_3$)

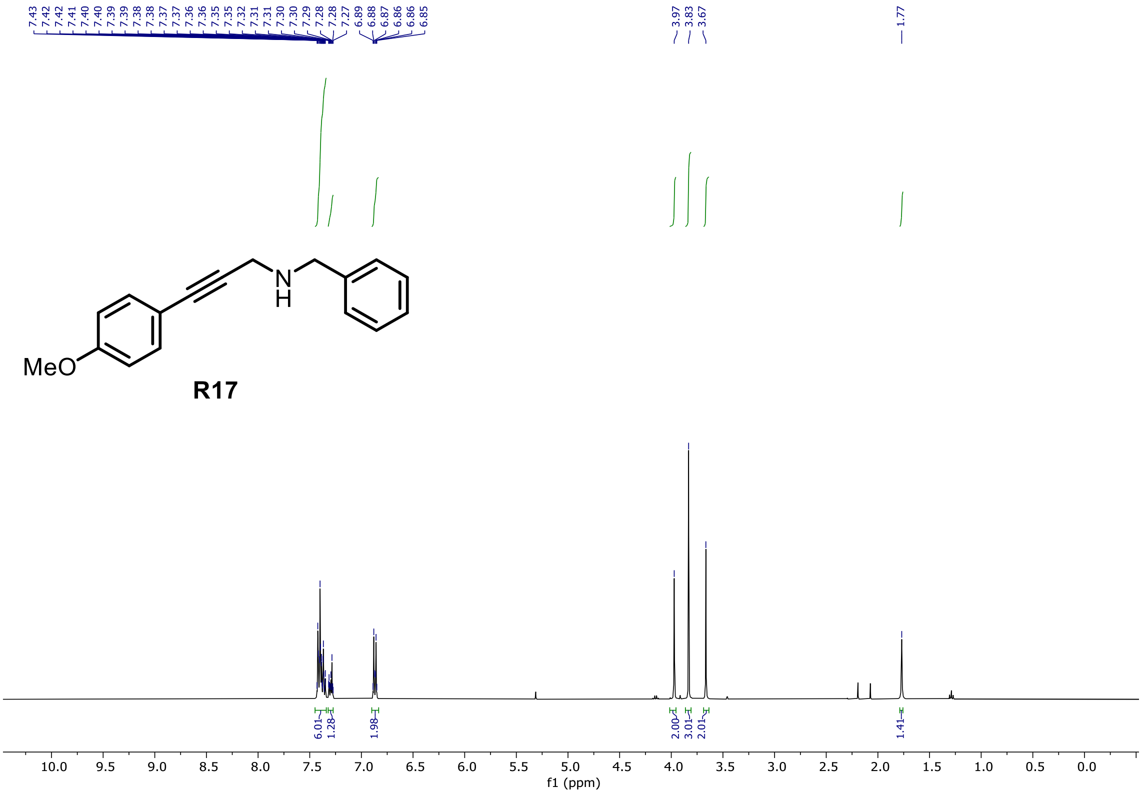

^{1}H NMR spectrum of compound **R17** (400 MHz, $CDCl_3$)

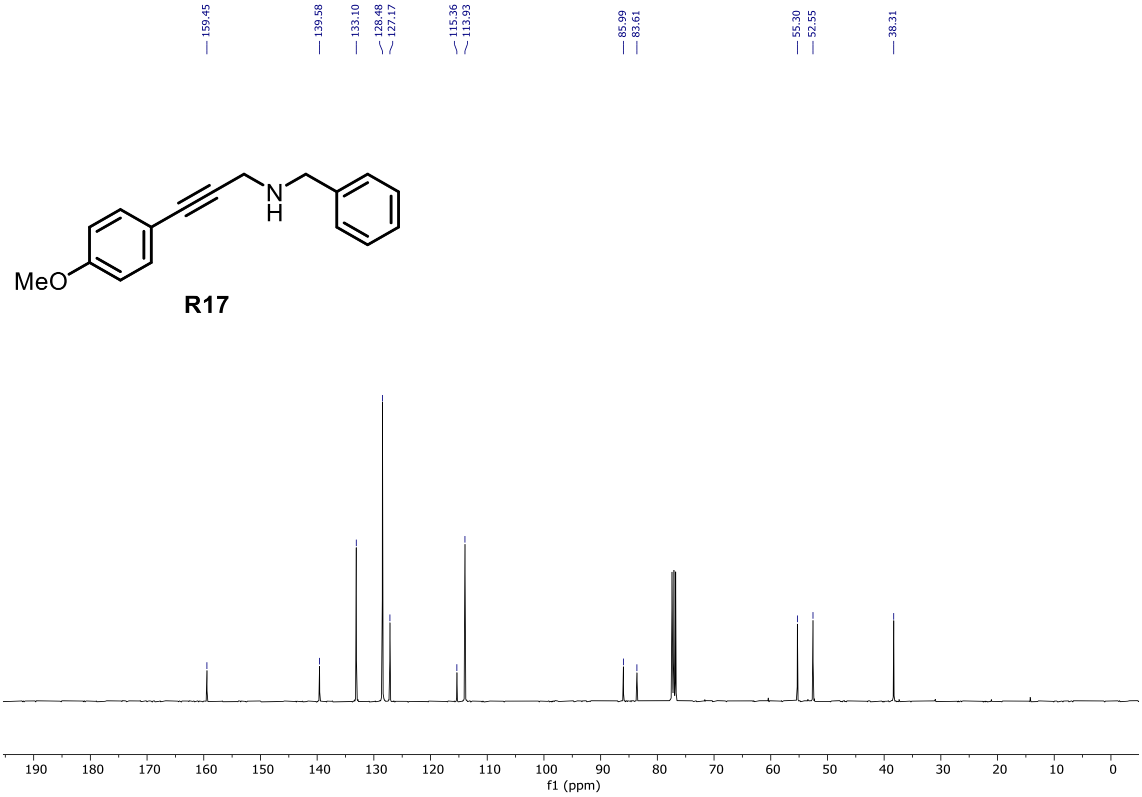

^{13}C{^{1}H} NMR spectrum of compound **R17** (101 MHz, $CDCl_3$)

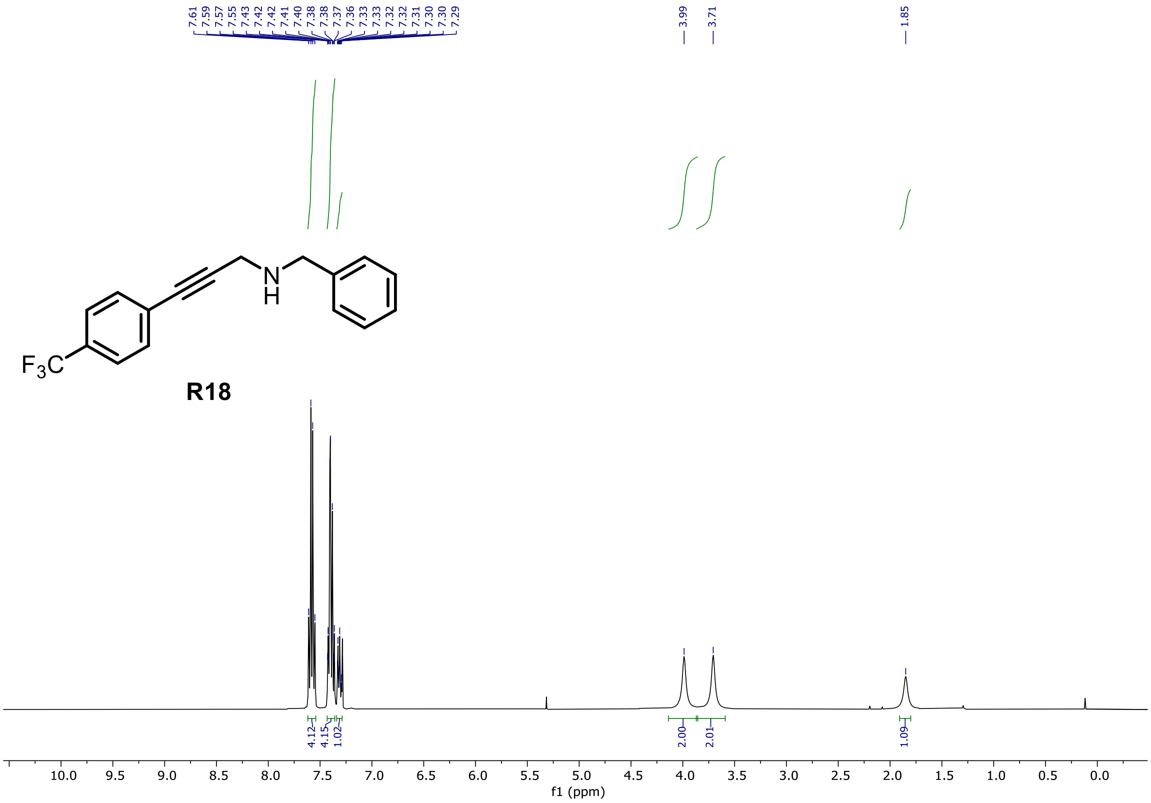

^{1}H NMR spectrum of compound **R18** (400 MHz, $CDCl_3$)

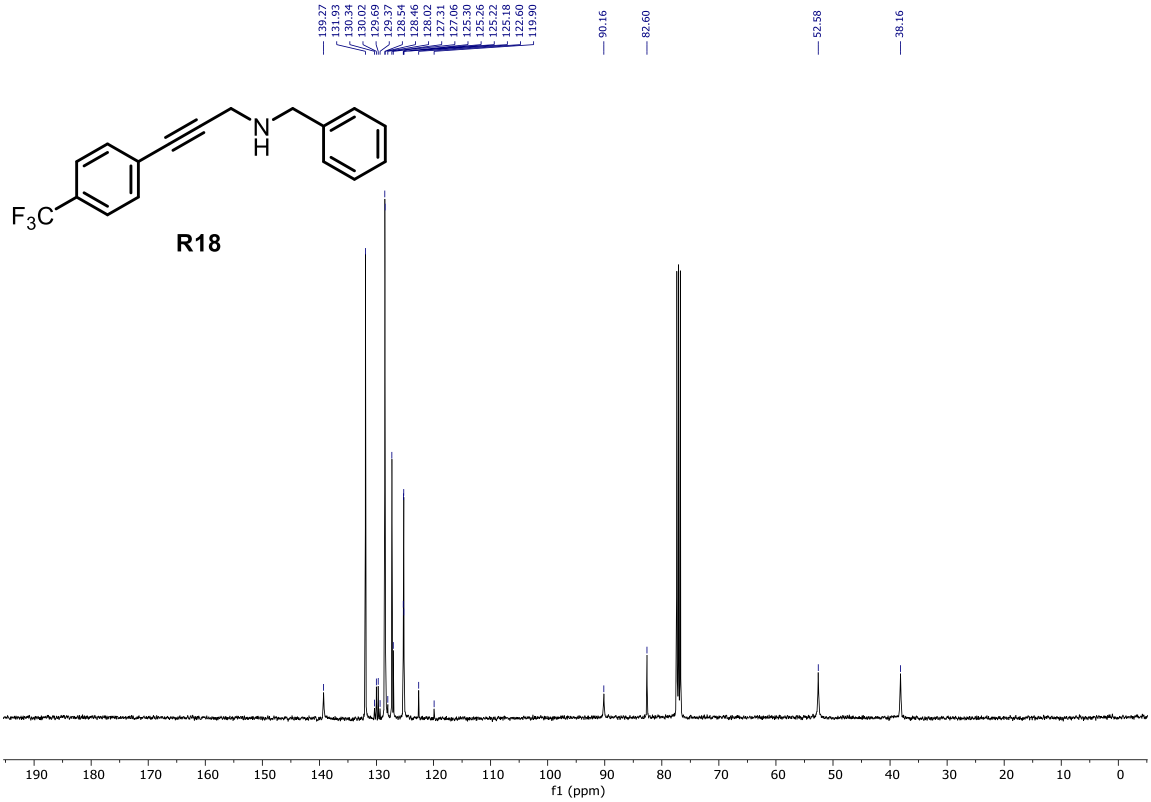

$^{13}C\{^{1}H\}$ NMR spectrum of compound **R18** (101 MHz, $CDCl_3$)

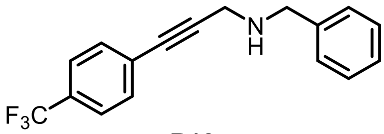
R18

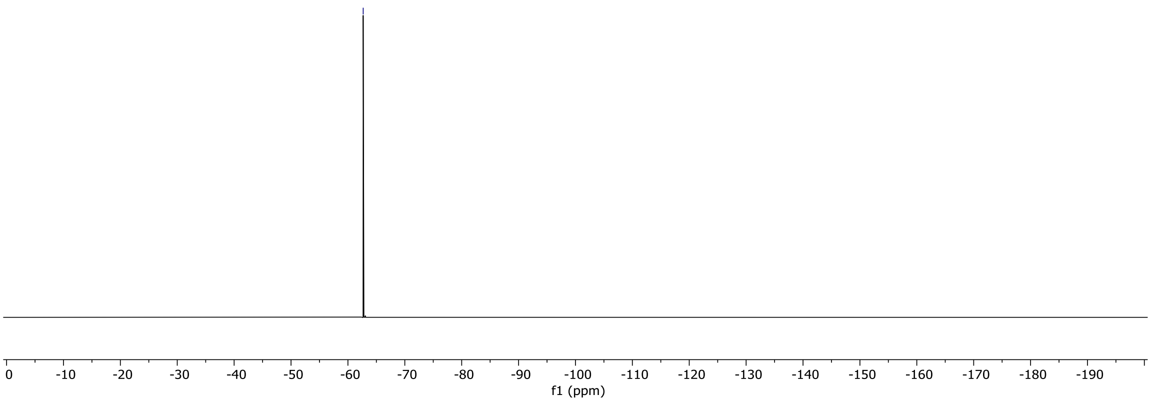

^{19}F NMR spectrum of compound **R18** (565 MHz, $CDCl_3$)

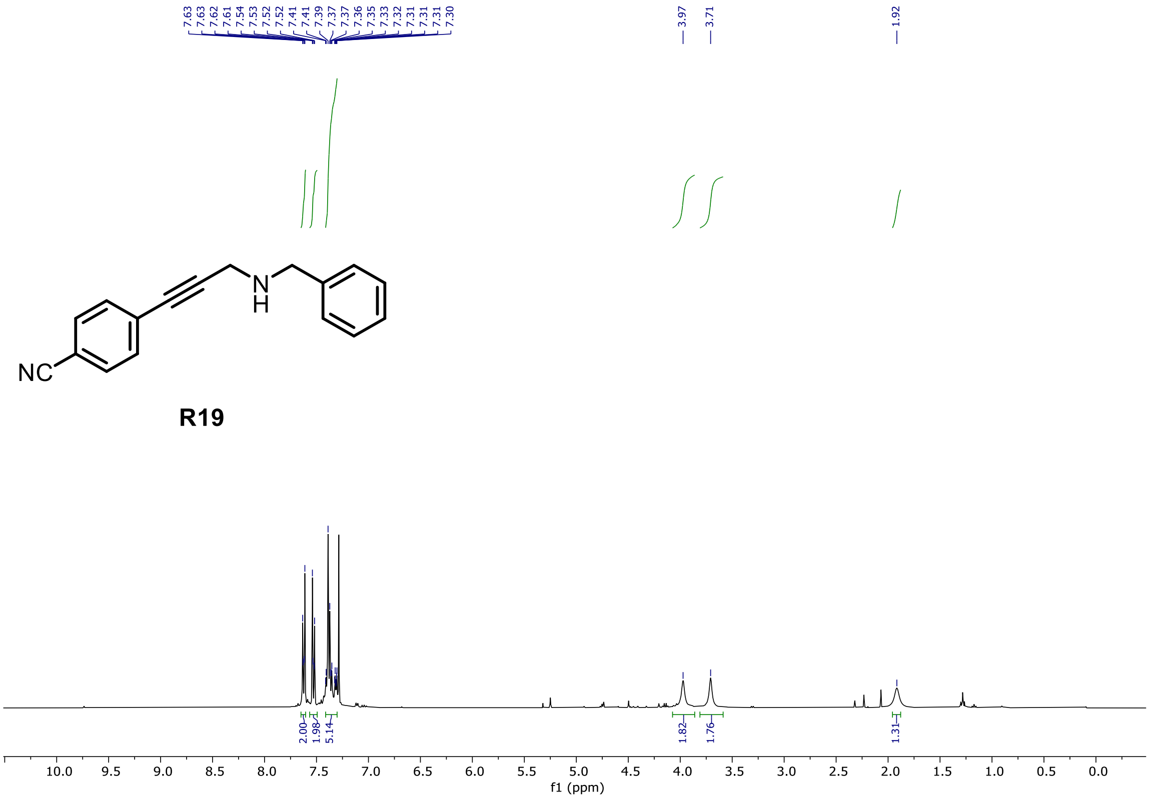

^{1}H NMR spectrum of compound **R19** (400 MHz, $CDCl_3$)

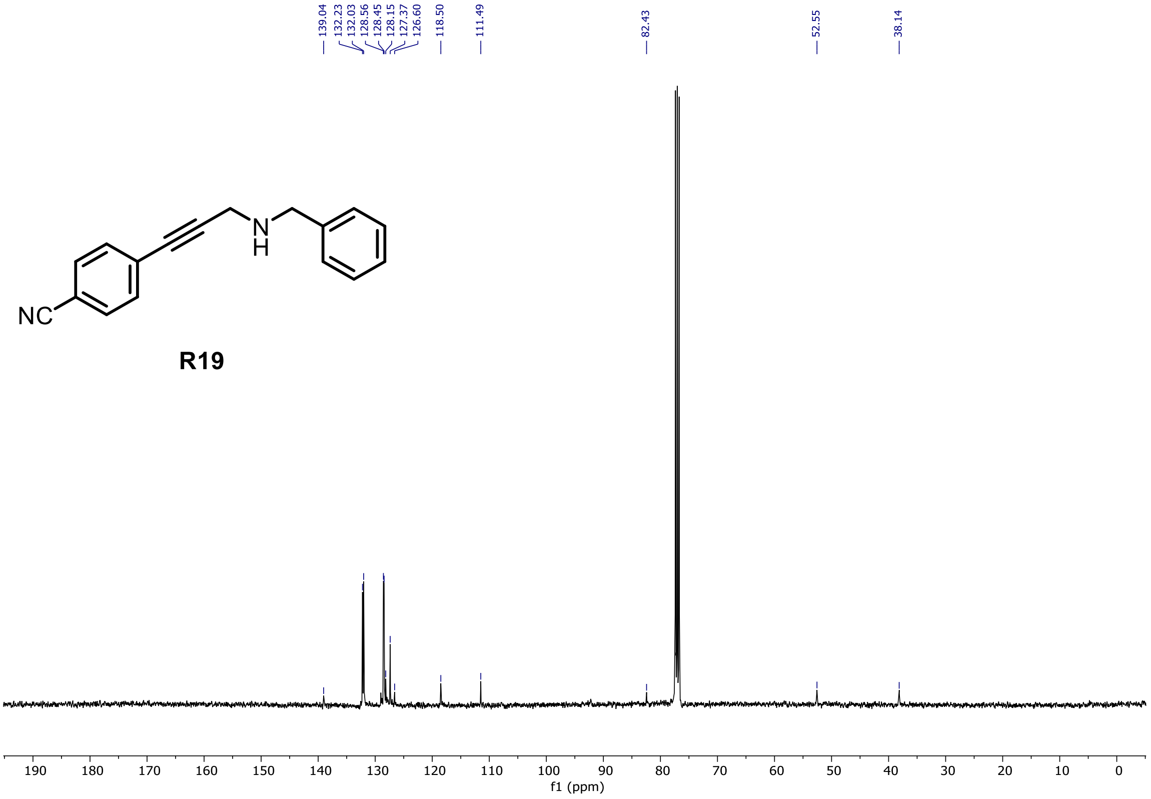

$^{13}C\{^1H\}$ NMR spectrum of compound **R19** (101 MHz, $CDCl_3$)

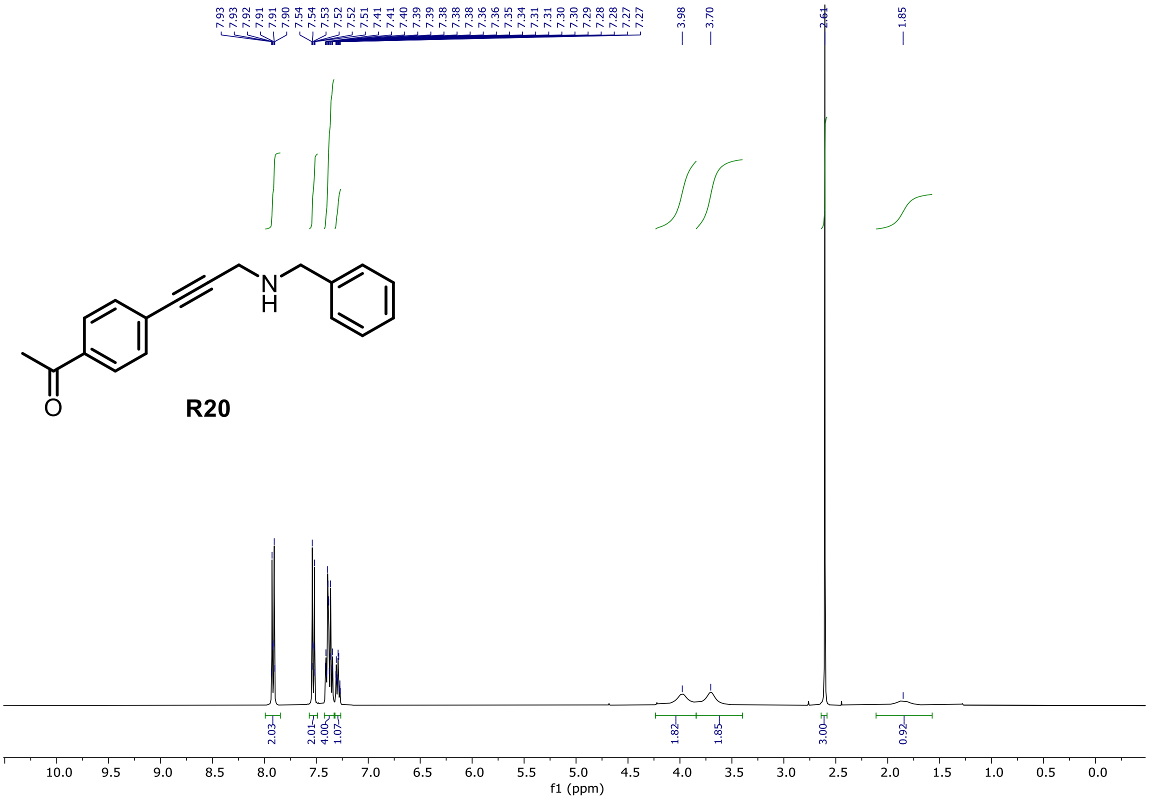

^1H NMR spectrum of compound **R20** (400 MHz, $CDCl_3$)

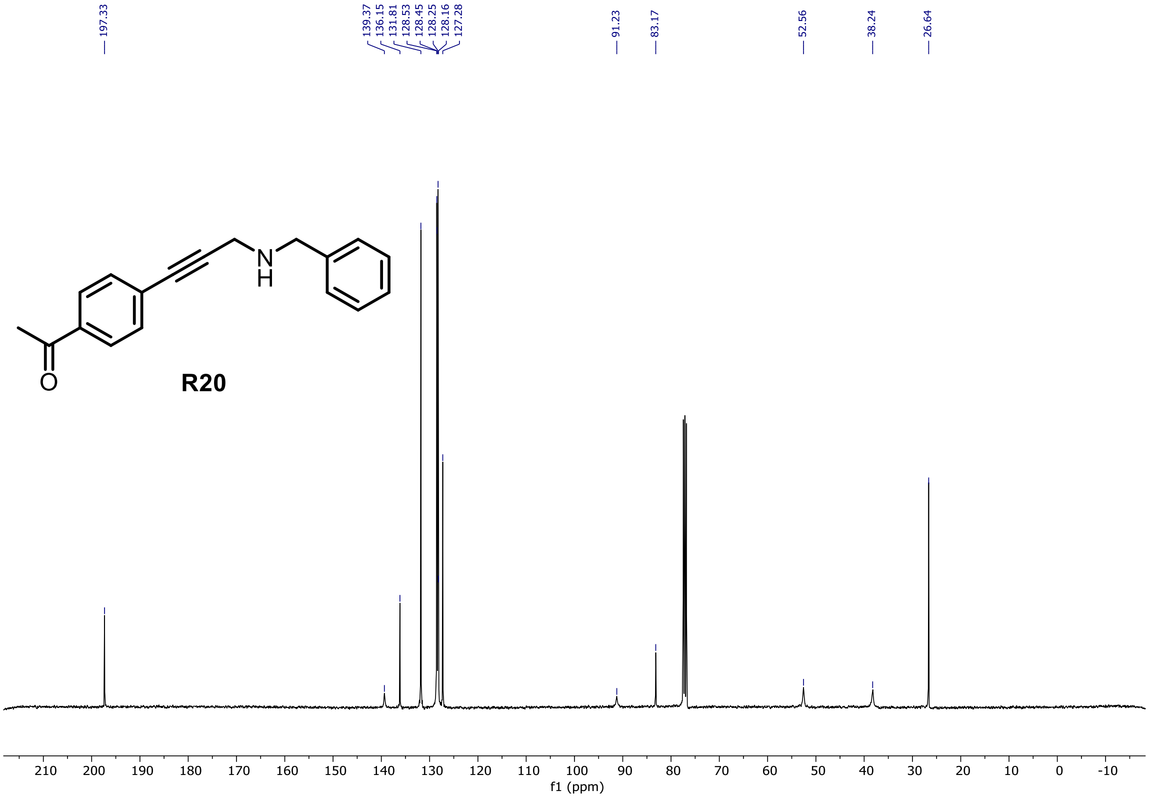

$^{13}C\{^1H\}$ NMR spectrum of compound **R20** (101 MHz, $CDCl_3$)

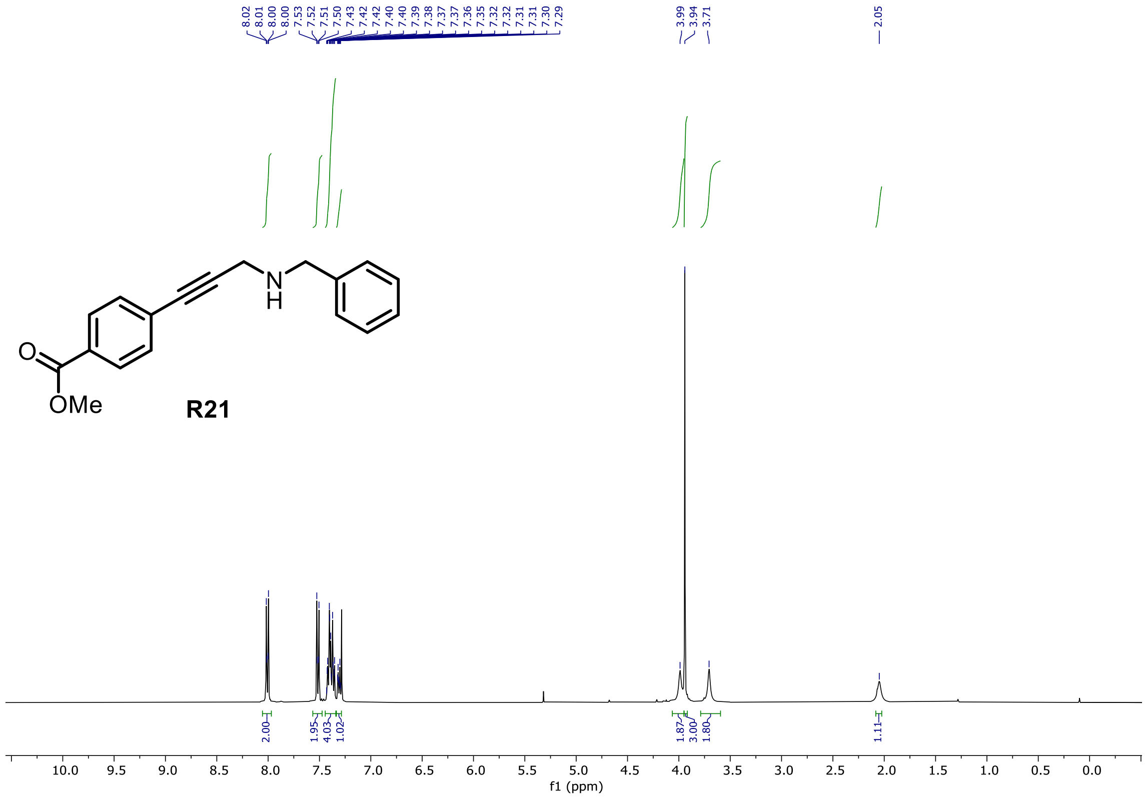

^{1}H NMR spectrum of compound **R21** (400 MHz, $CDCl_3$)

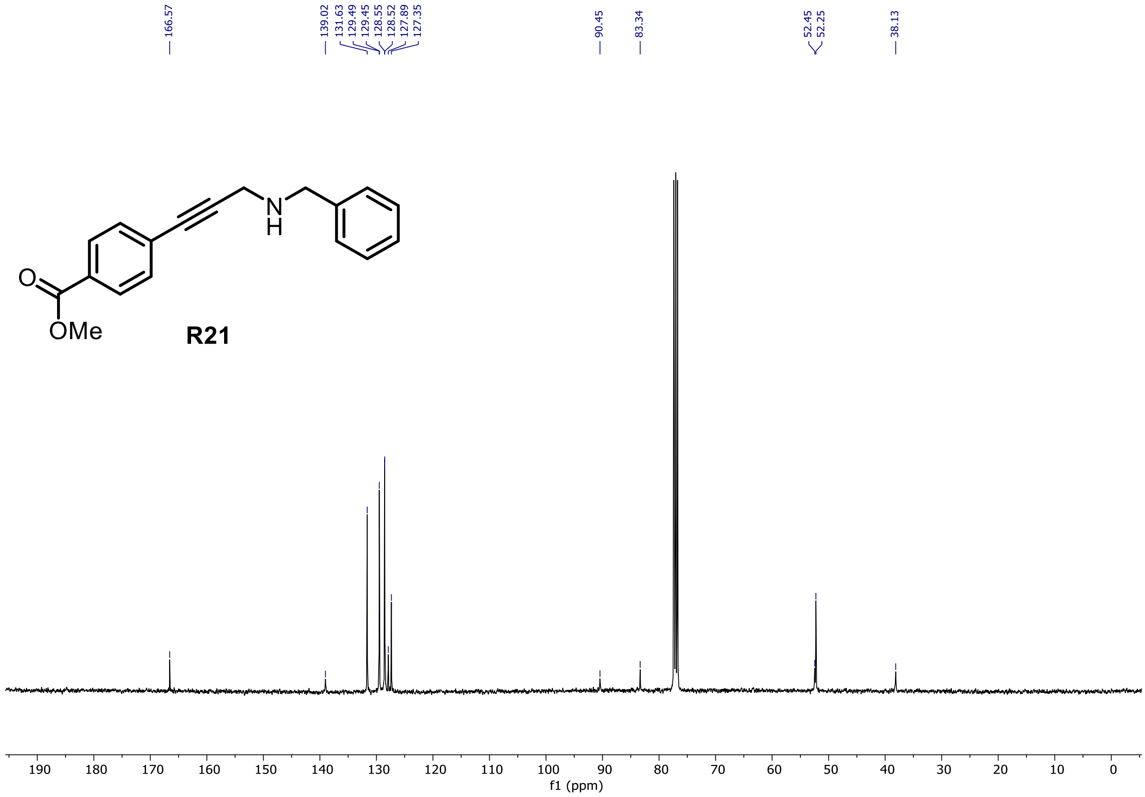

^{13}C{^{1}H} NMR spectrum of compound **R21** (101 MHz, $CDCl_3$)

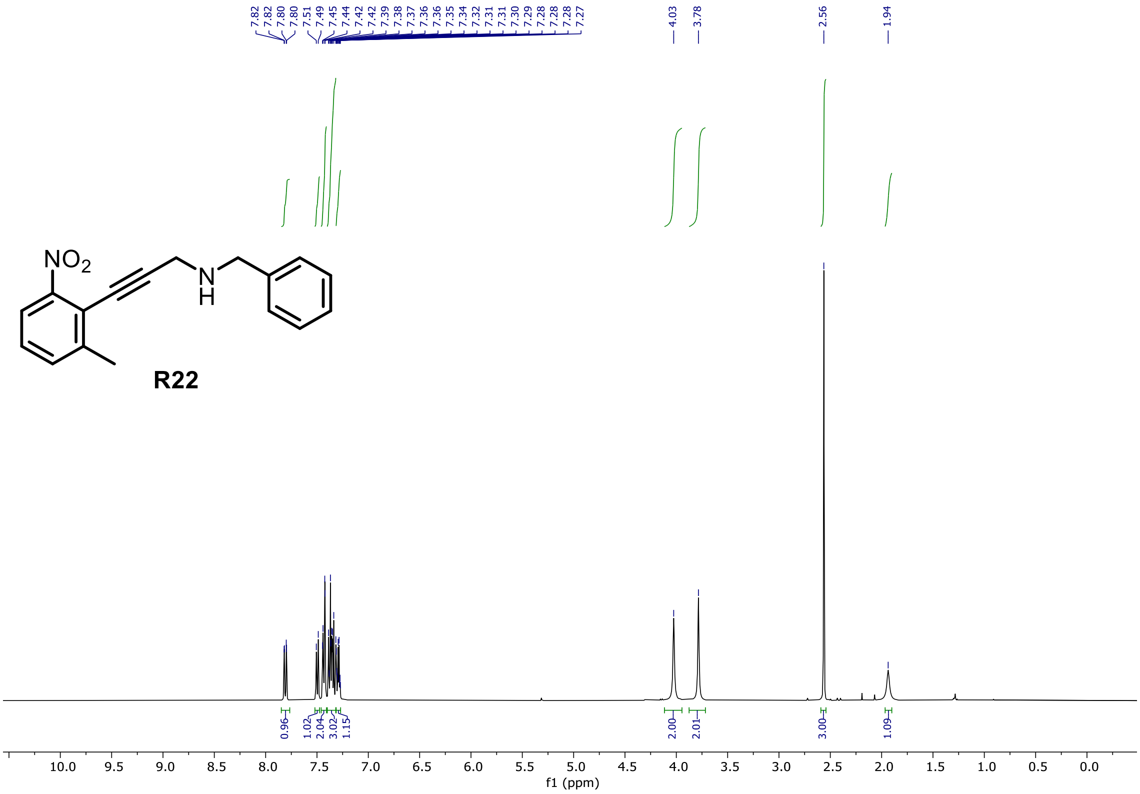

^{1}H NMR spectrum of compound **R22** (400 MHz, $CDCl_3$)

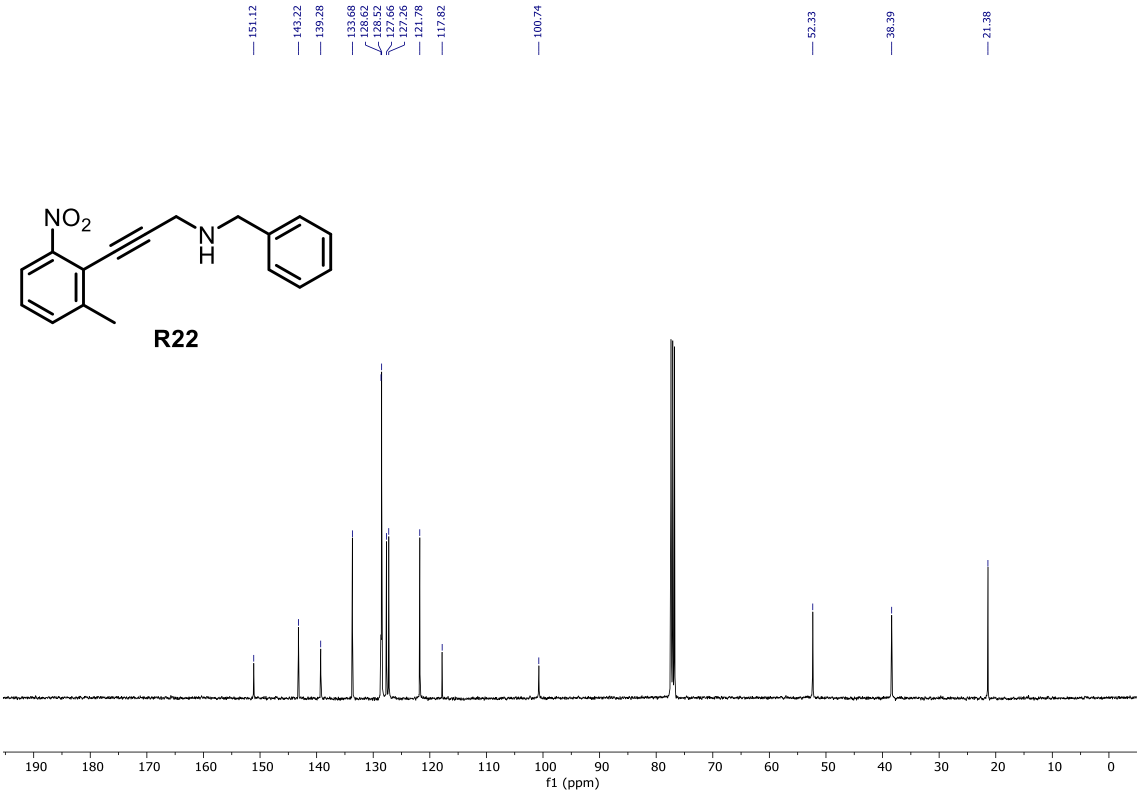

^{13}C{^{1}H} NMR spectrum of compound **R22** (101 MHz, $CDCl_3$)

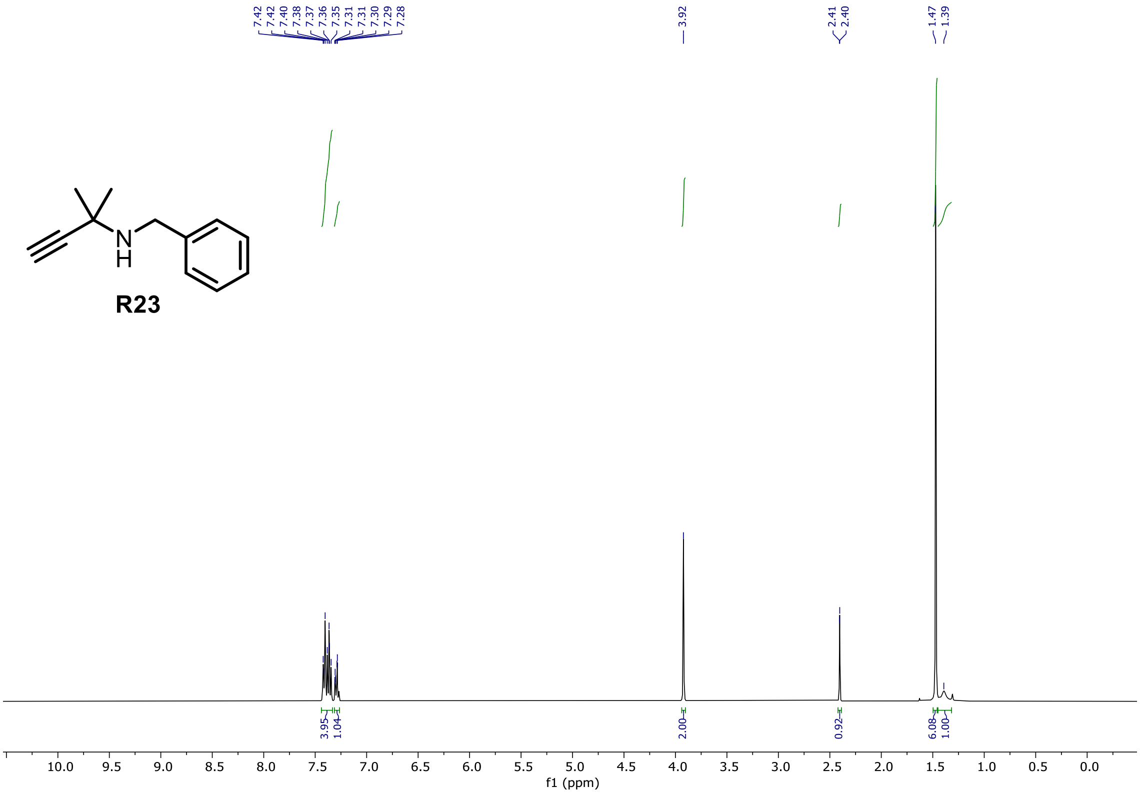

^{1}H NMR spectrum of compound **R23** (400 MHz, $CDCl_3$)

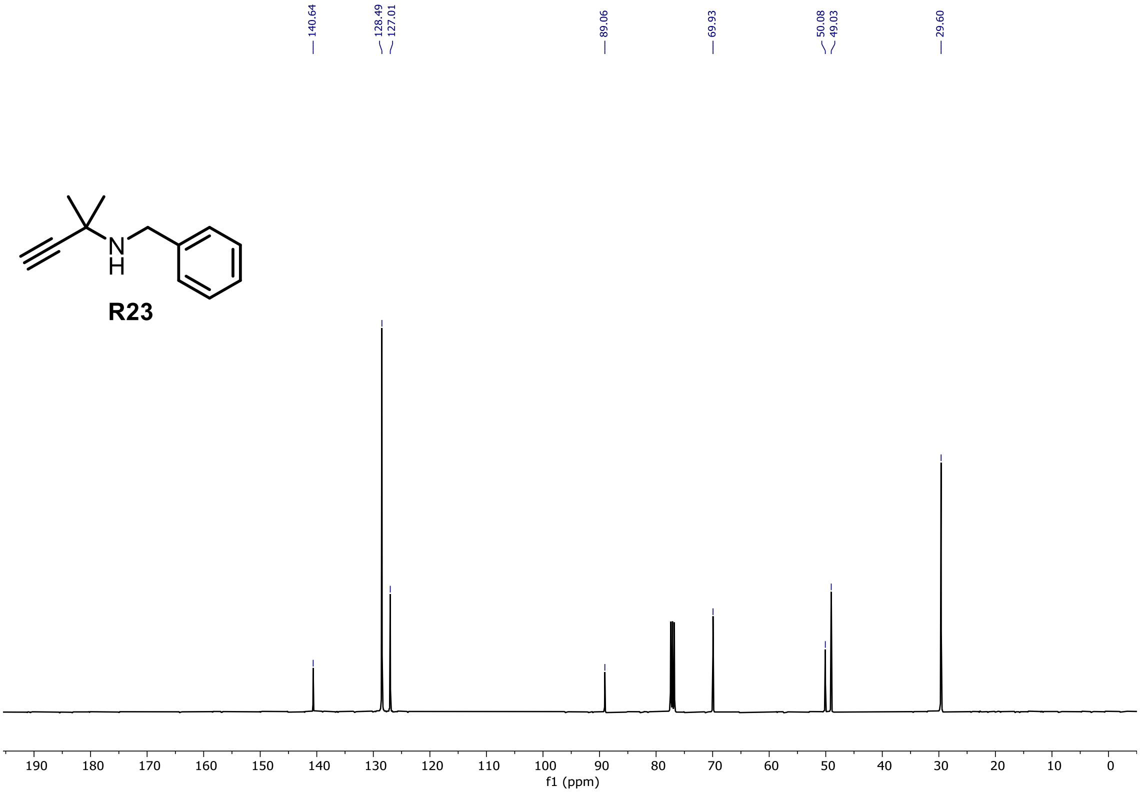

^{13}C{^{1}H} NMR spectrum of compound **R23** (101 MHz, $CDCl_3$)

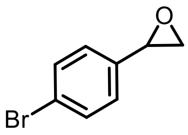

R29

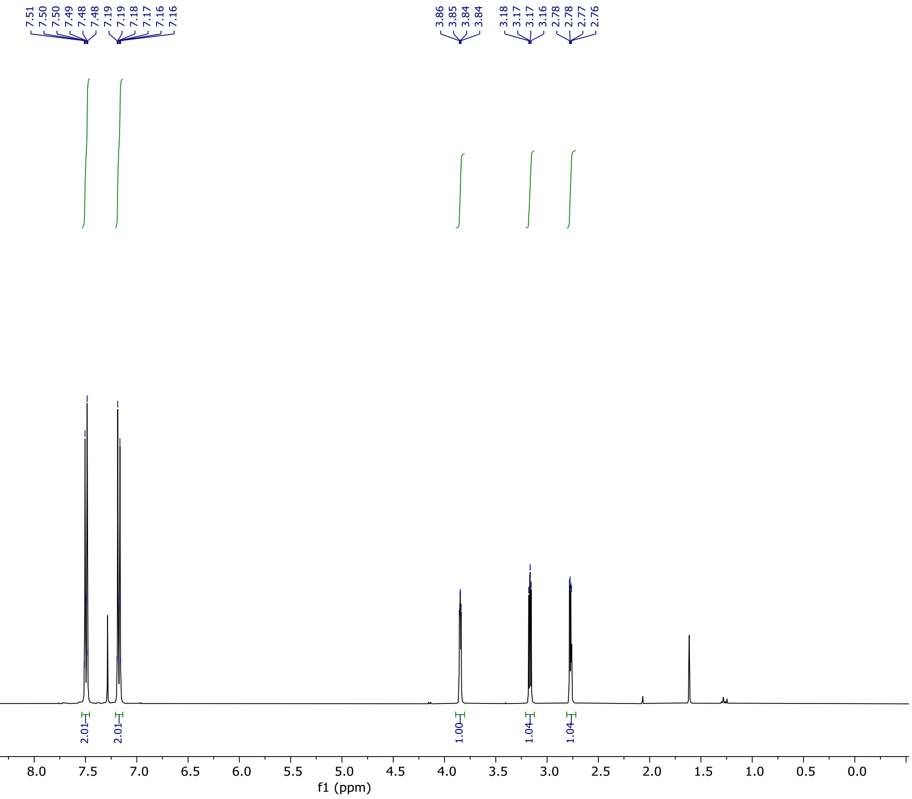

^{1}H NMR spectrum of compound **R29** (400 MHz, $CDCl_3$)

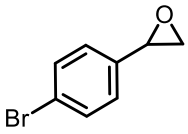

R29

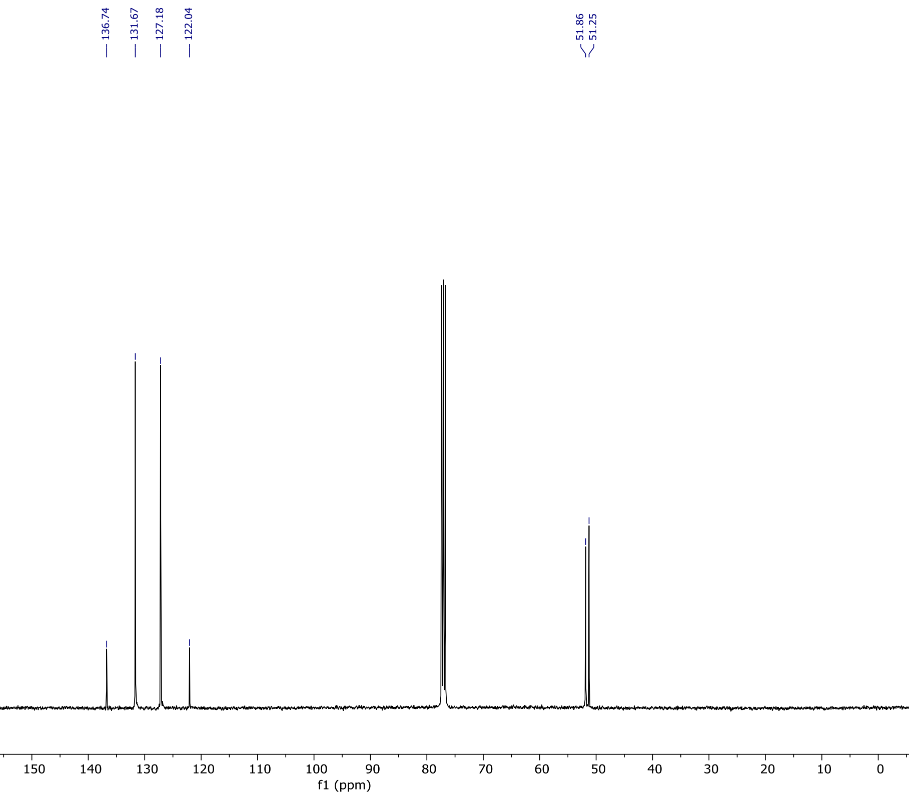

$^{13}C\{^{1}H\}$ NMR spectrum of compound **R29** (101 MHz, $CDCl_3$)

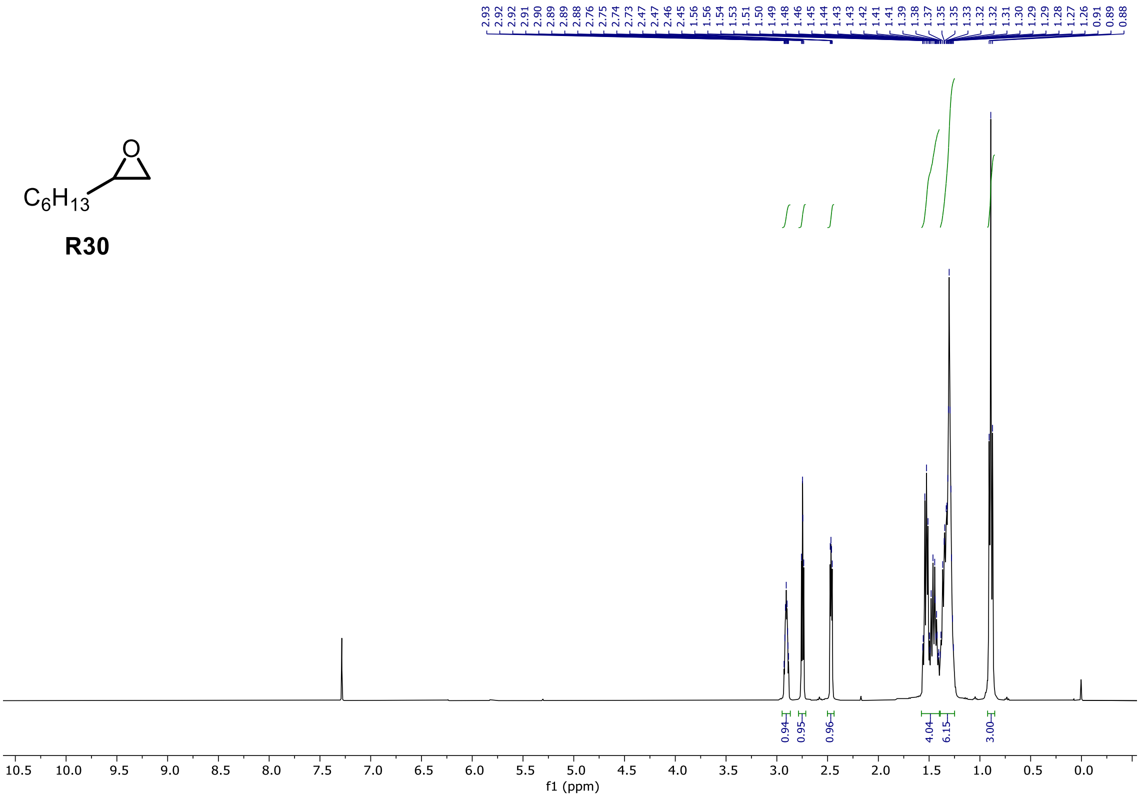

^{1}H NMR spectrum of compound **R30** (400 MHz, $CDCl_3$)

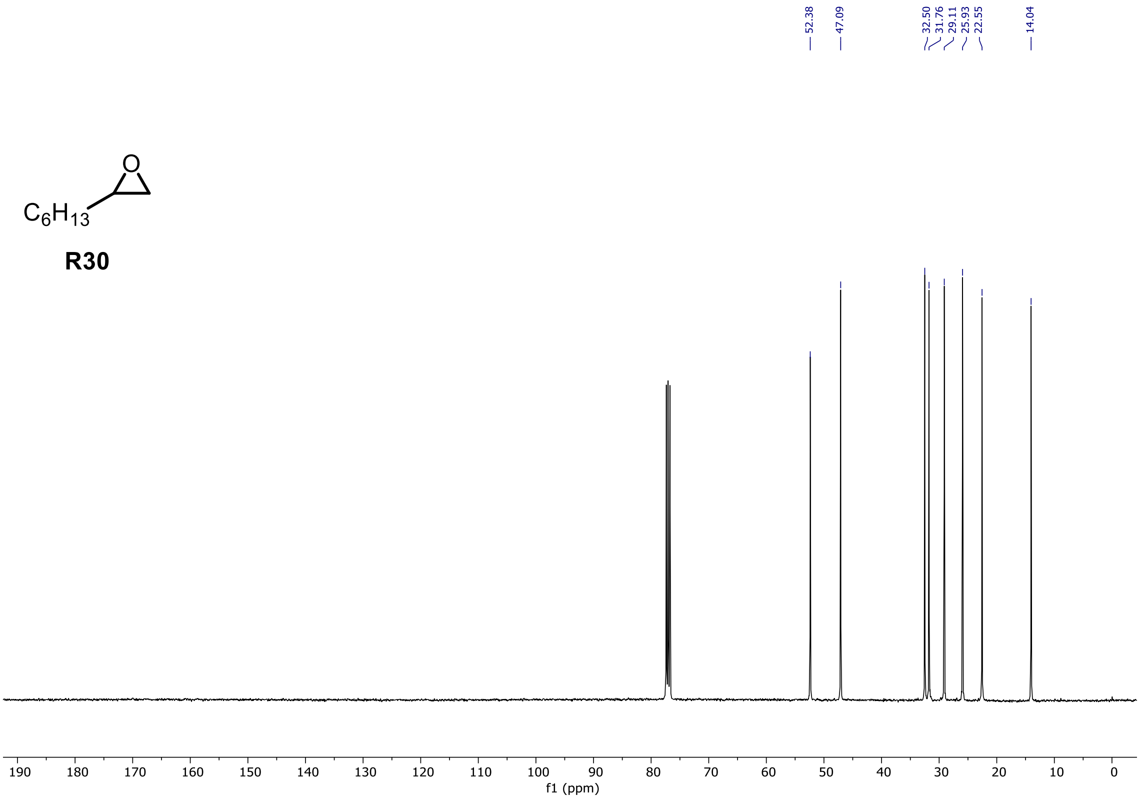

^{13}C{^{1}H} NMR spectrum of compound **R30** (101 MHz, $CDCl_3$)

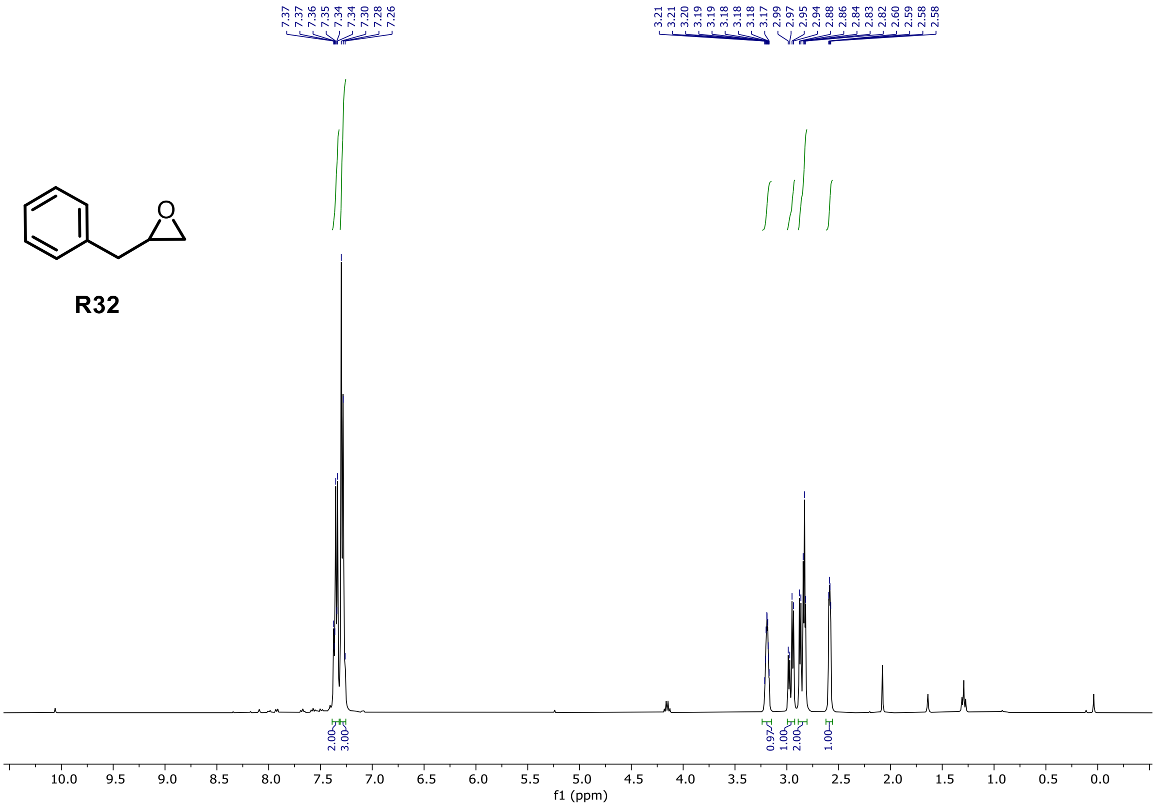

^{1}H NMR spectrum of compound **R32** (400 MHz, $CDCl_3$)

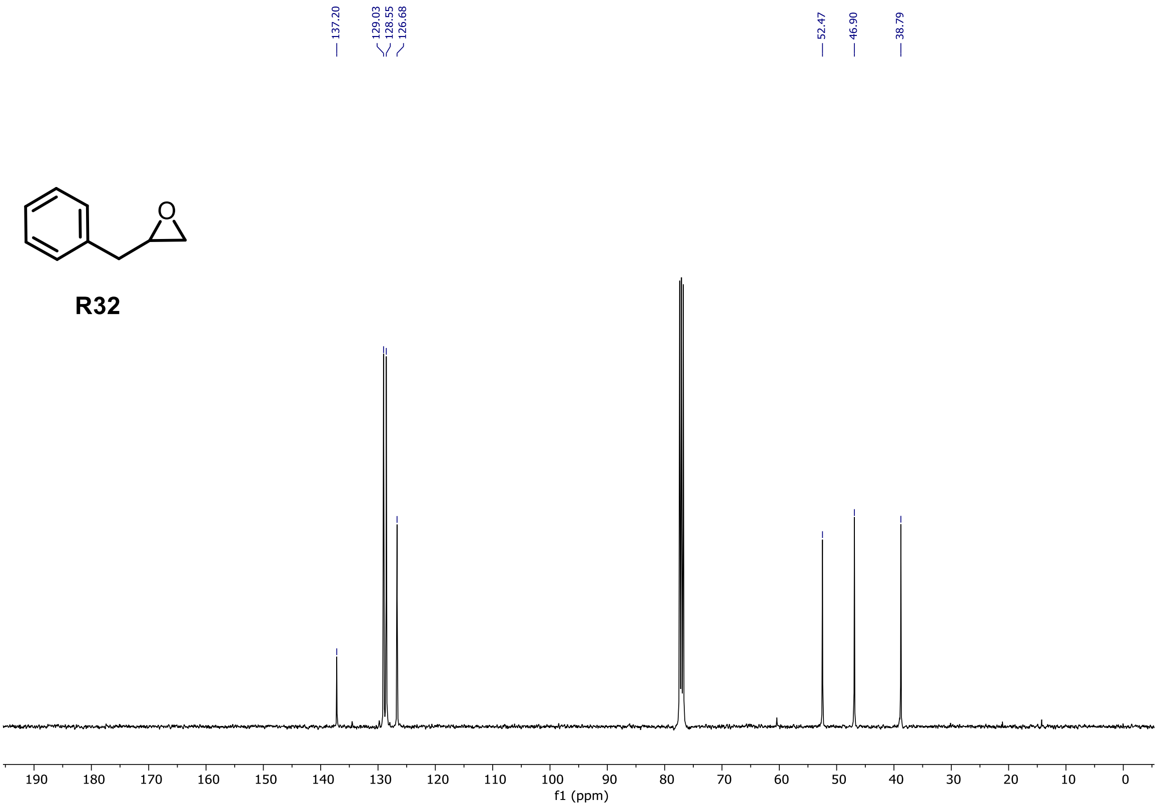

$^{13}C\{^{1}H\}$ NMR spectrum of compound **R32** (101 MHz, $CDCl_3$)

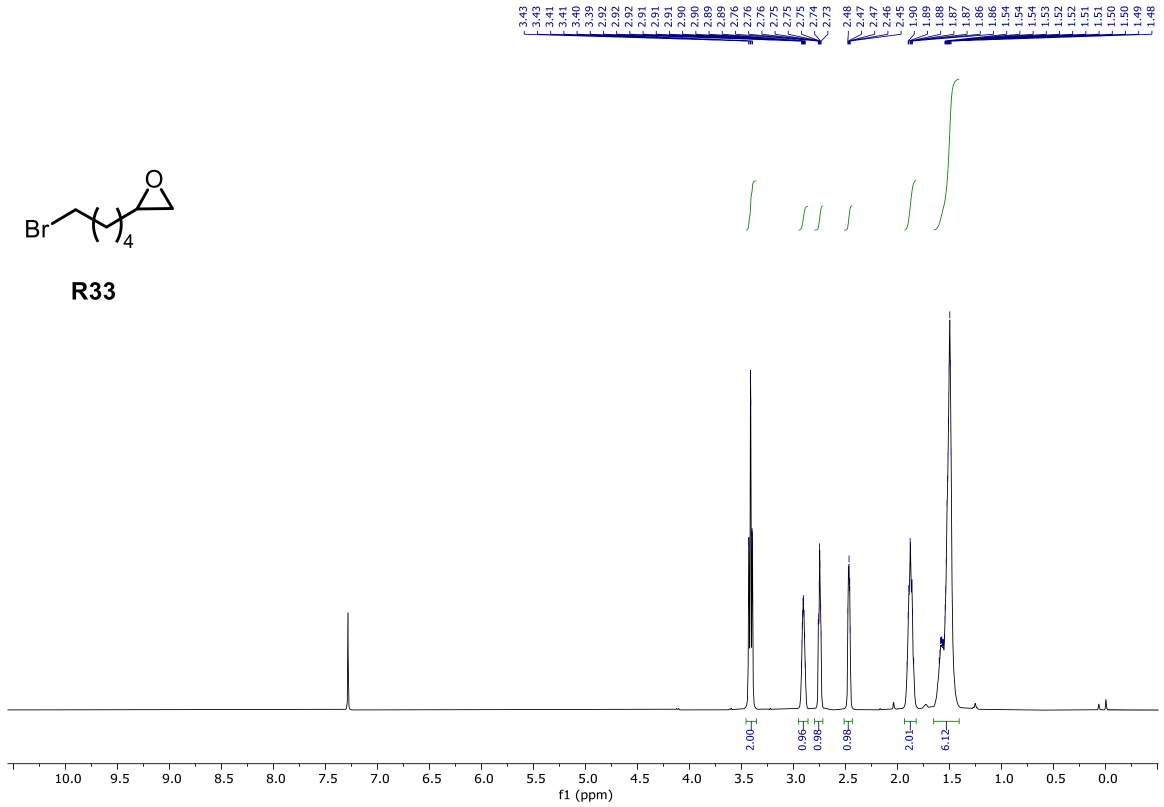

^{1}H NMR spectrum of compound **R33** (400 MHz, $CDCl_3$)

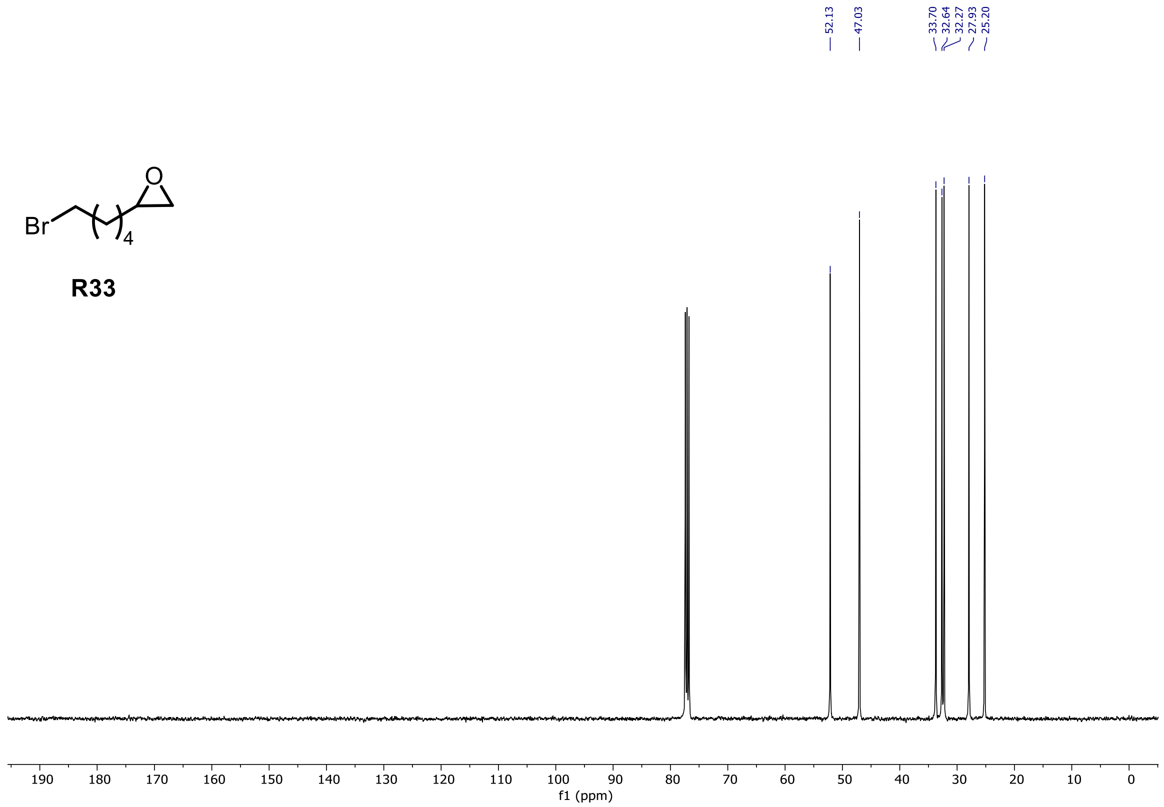

$^{13}C\{^{1}H\}$ NMR spectrum of compound **R33** (101 MHz, $CDCl_3$)

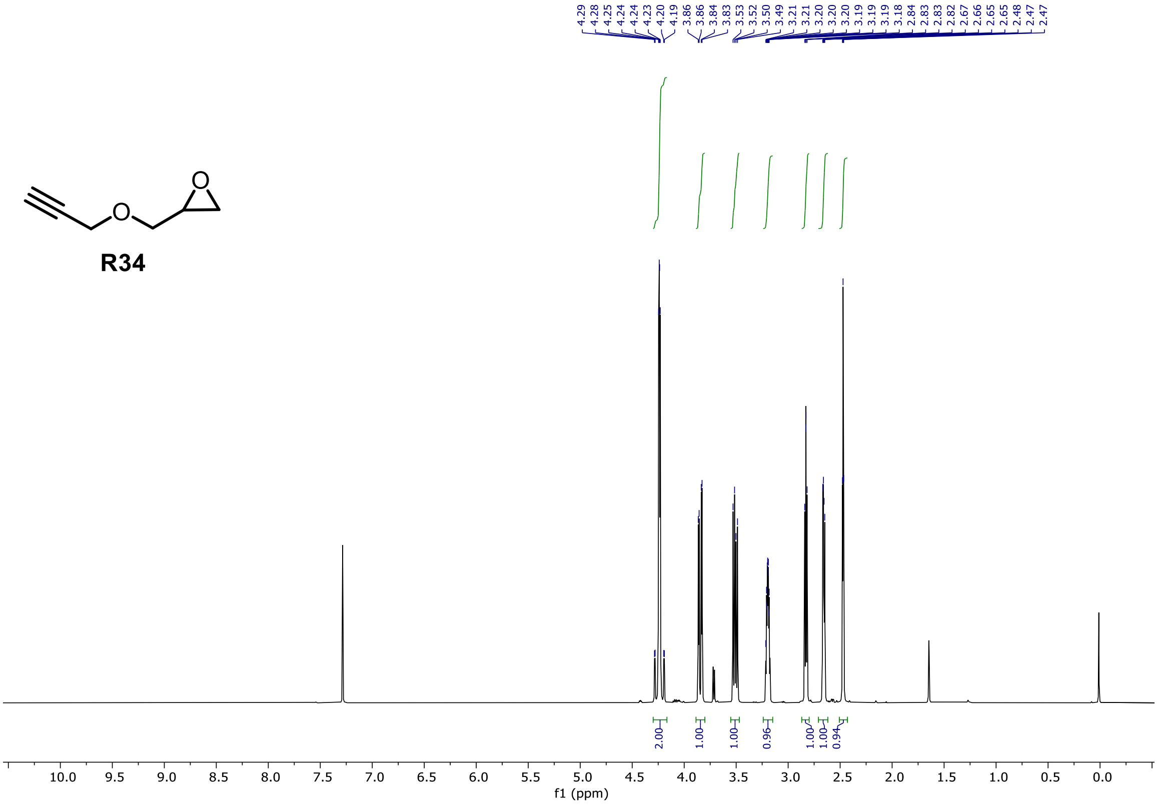

R34

^{1}H NMR spectrum of compound **R34** (400 MHz, $CDCl_3$)

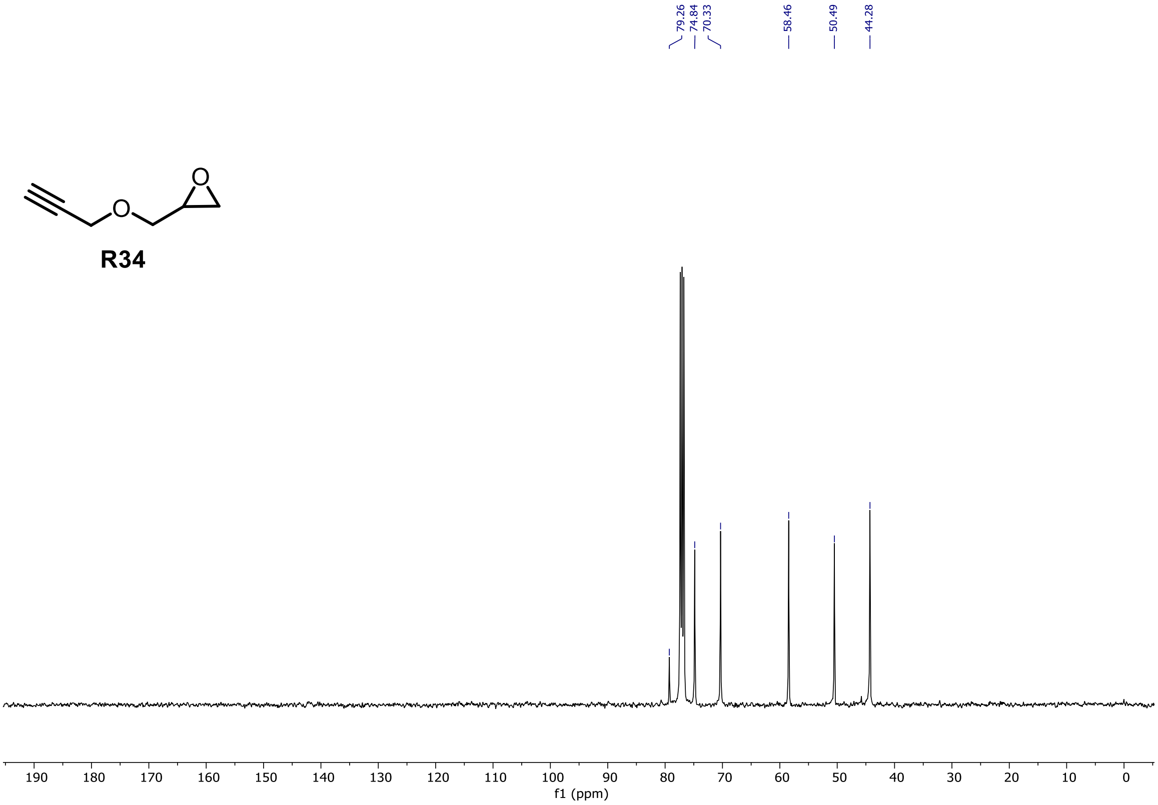

R34

$^{13}C\{^1H\}$ NMR spectrum of compound **R34** (101 MHz, $CDCl_3$)

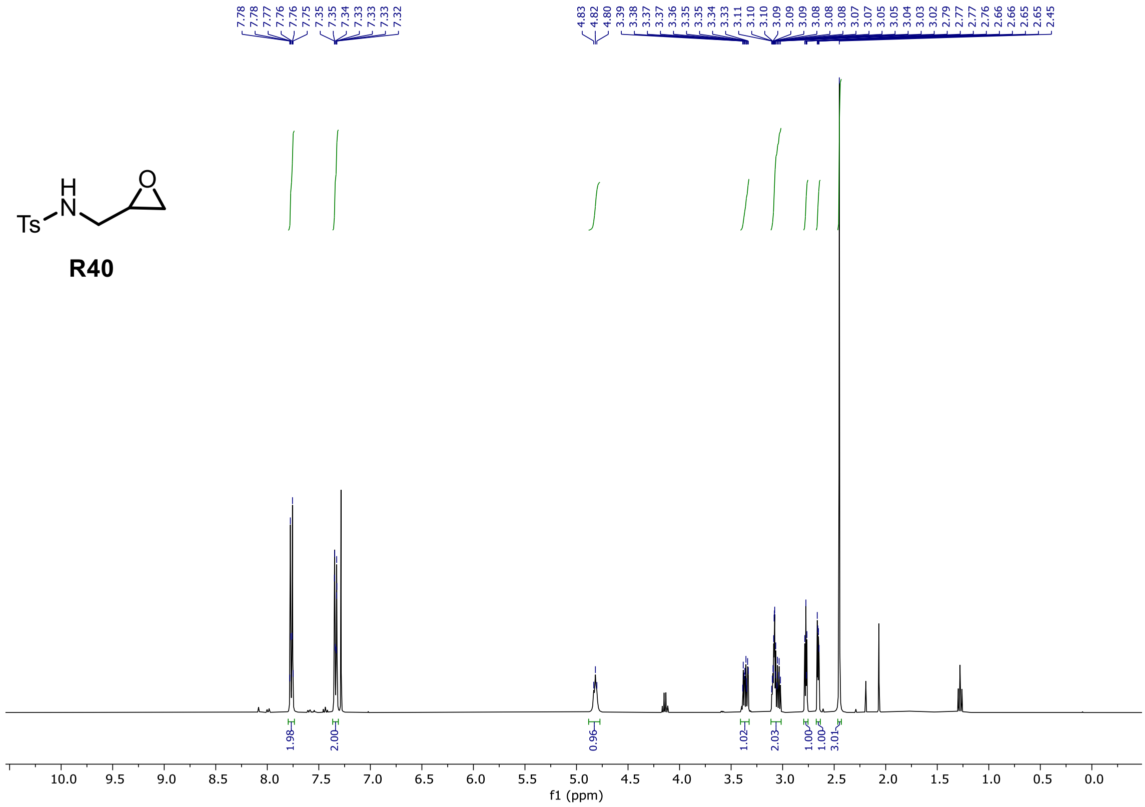

^{1}H NMR spectrum of compound **R40** (400 MHz, $CDCl_3$)

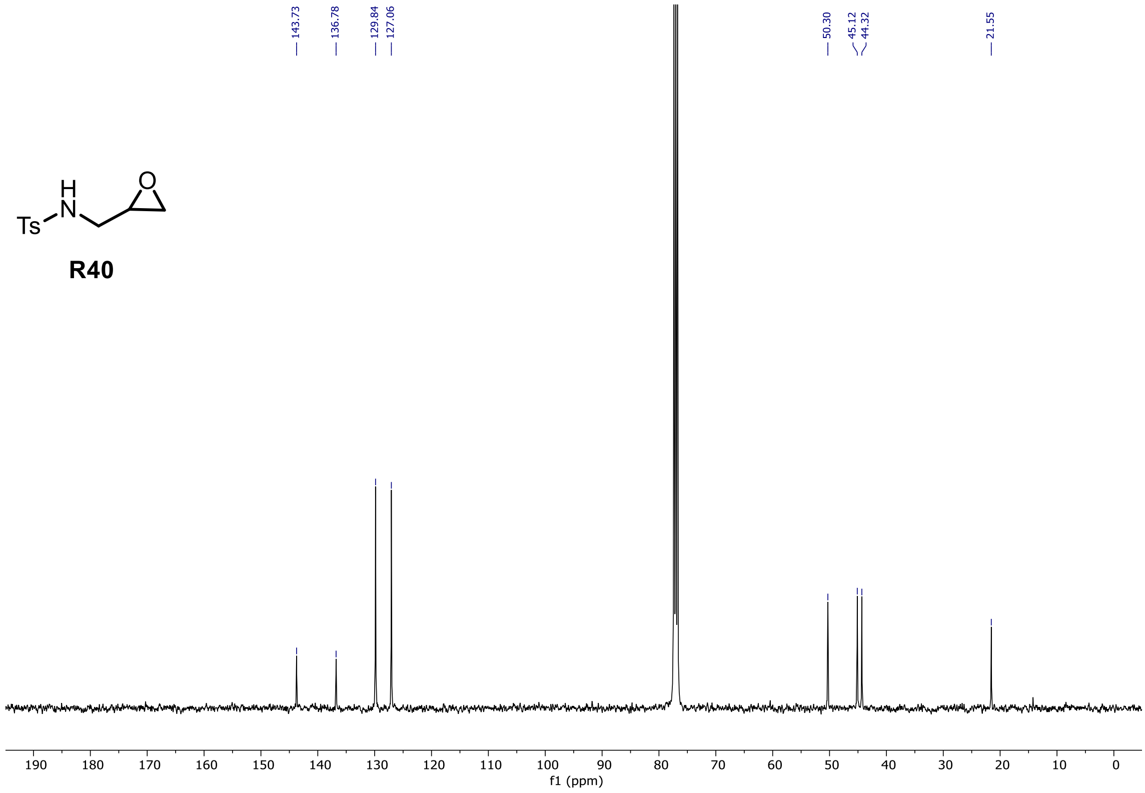

$^{13}C\{^{1}H\}$ NMR spectrum of compound **R40** (101 MHz, $CDCl_3$)

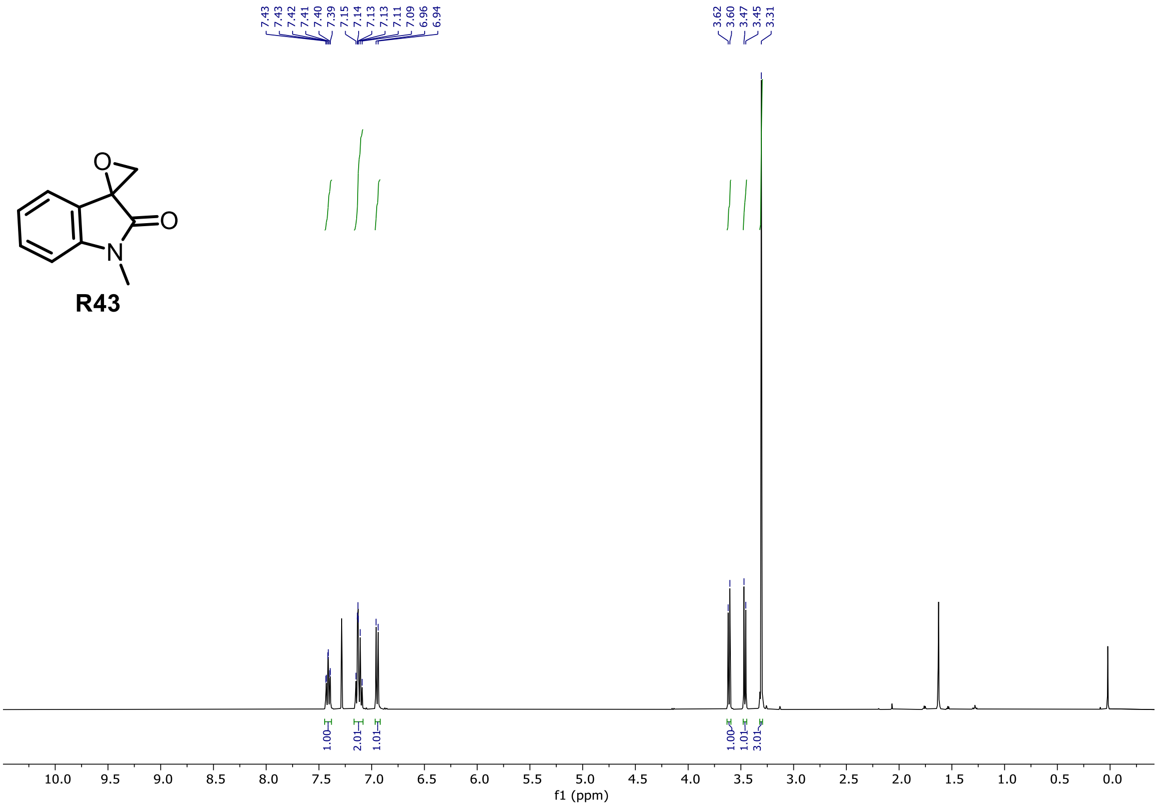

^{1}H NMR spectrum of compound **R43** (400 MHz, $CDCl_3$)

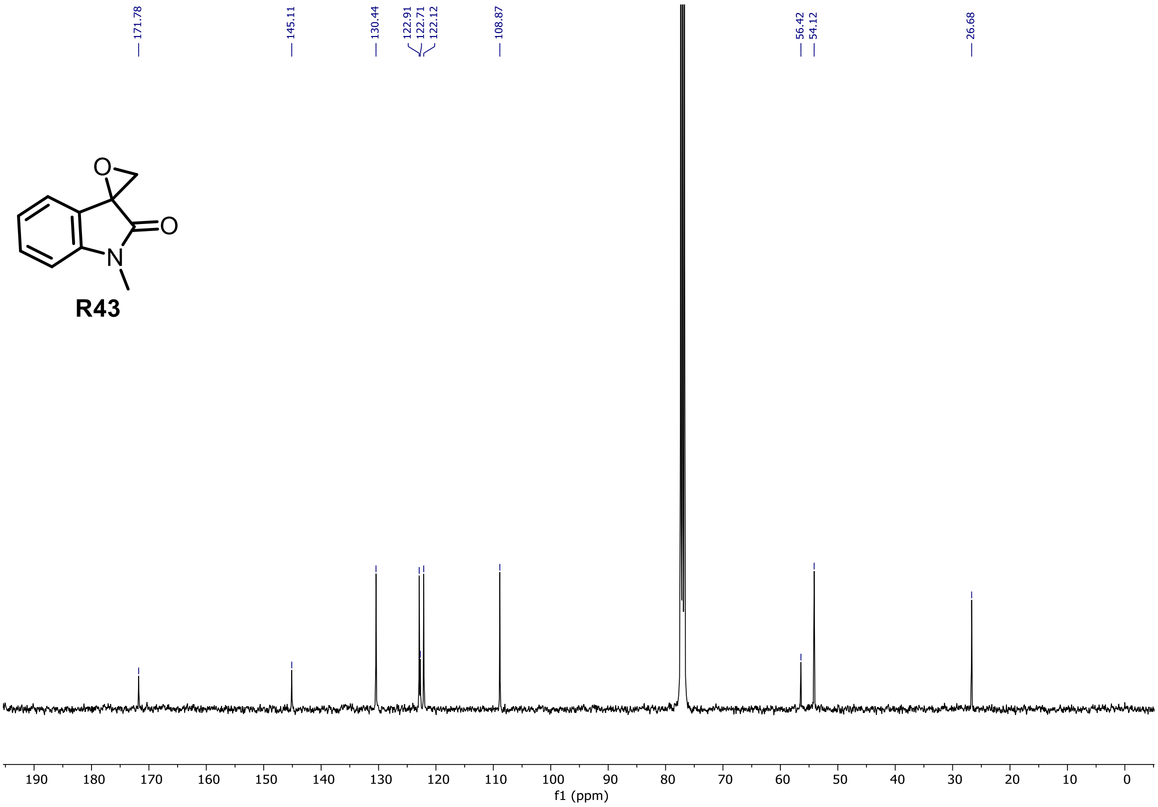

$^{13}C\{^{1}H\}$ NMR spectrum of compound **R43** (101 MHz, $CDCl_3$)

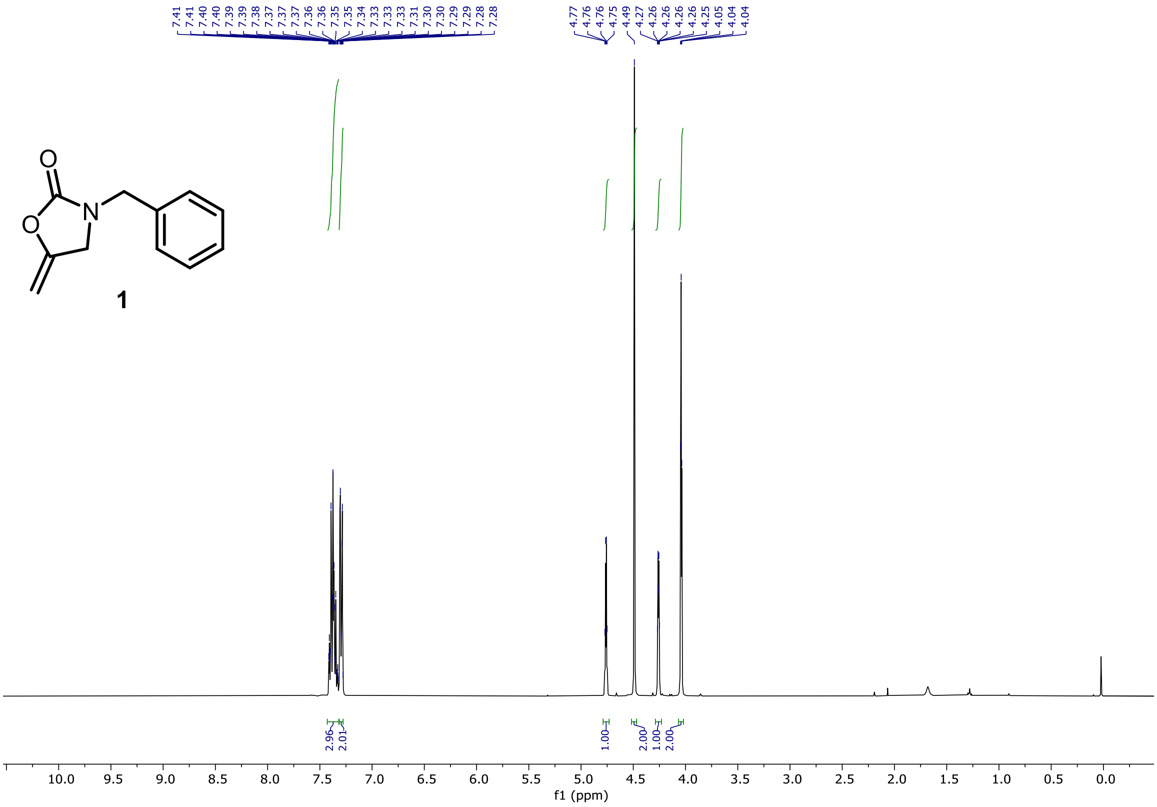

^{1}H NMR spectrum of compound **1** (400 MHz, $CDCl_3$)

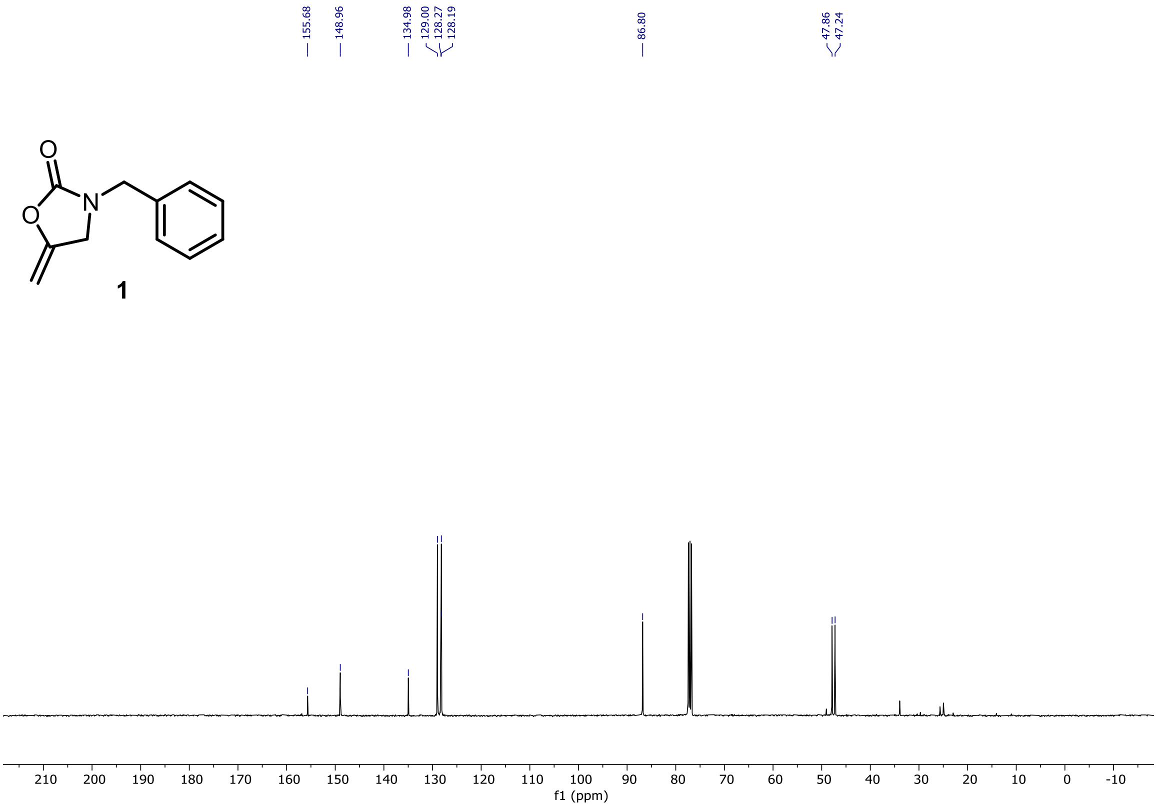

^{13}C{^{1}H} NMR spectrum of compound **1** (101 MHz, $CDCl_3$)

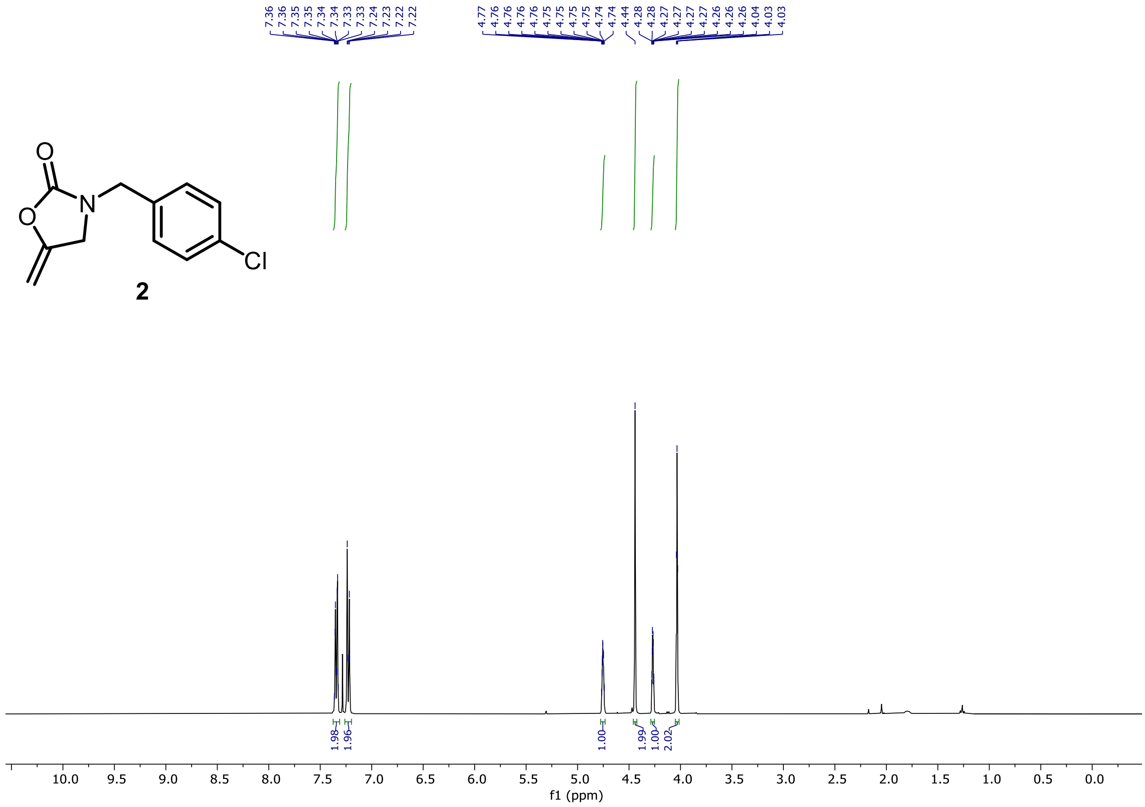

^{1}H NMR spectrum of compound **2** (400 MHz, $CDCl_3$)

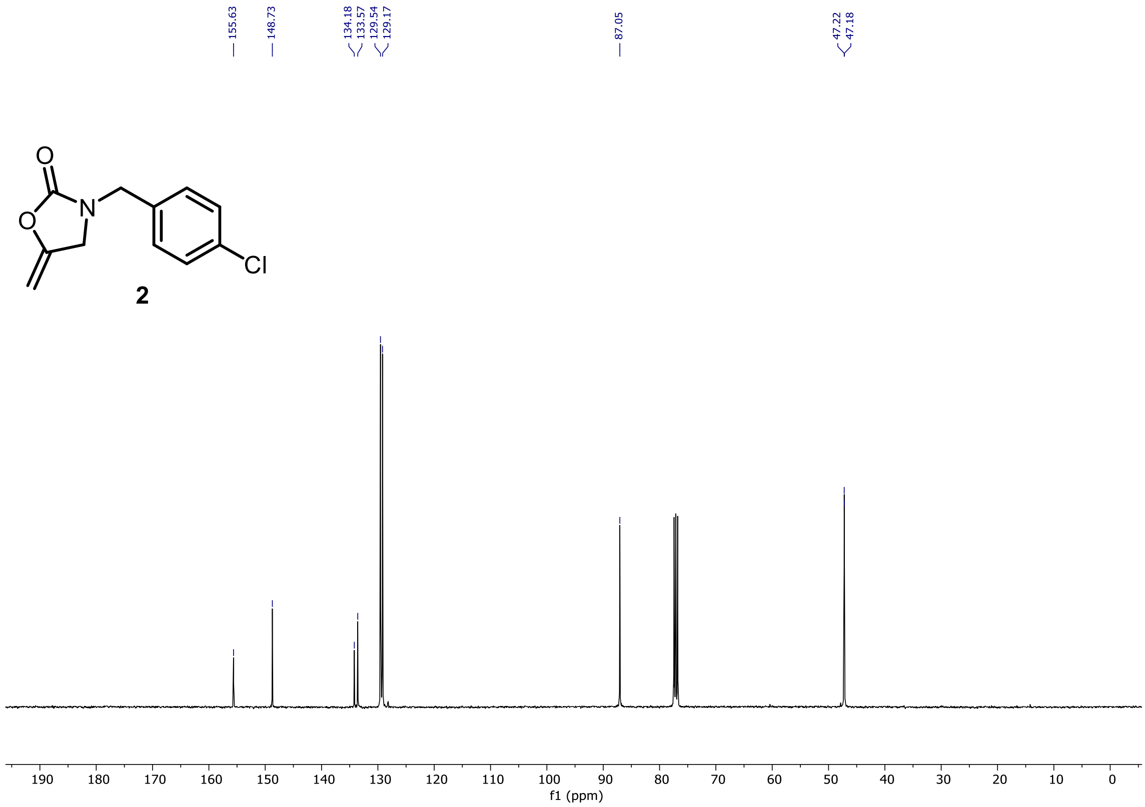

^{13}C{^{1}H} NMR spectrum of compound **2** (101 MHz, $CDCl_3$)

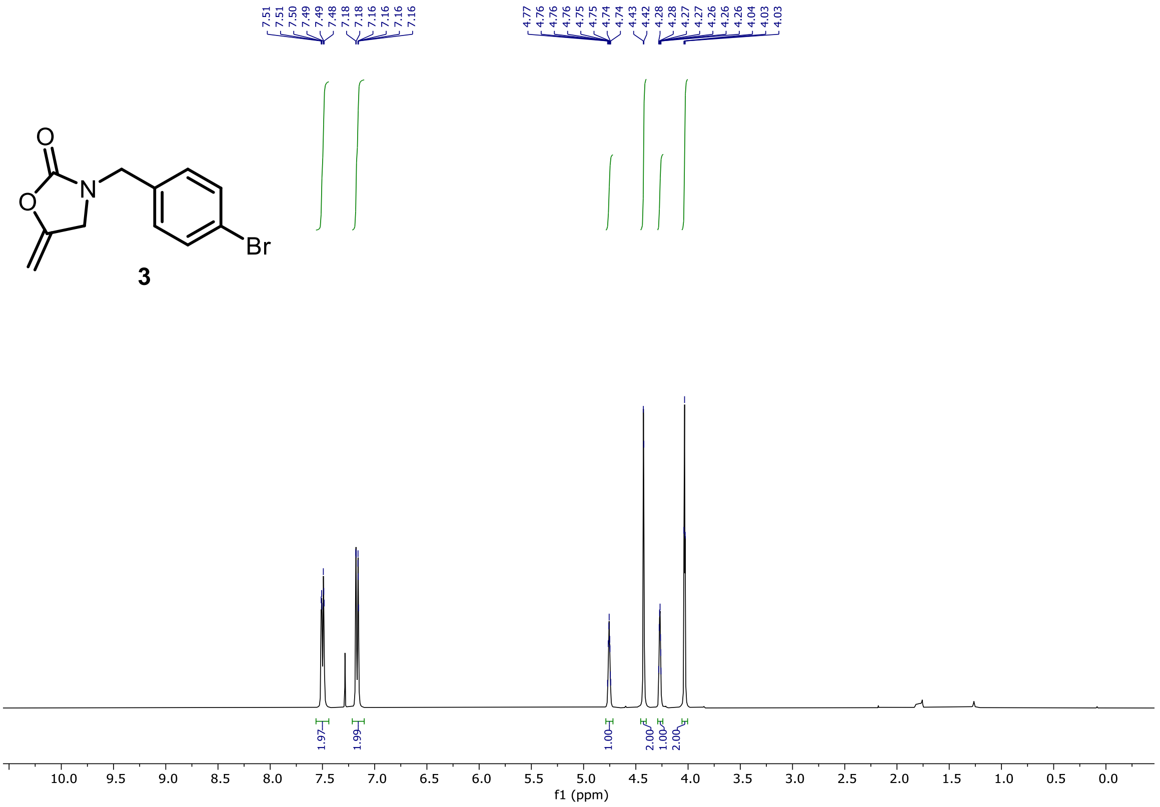

^{1}H NMR spectrum of compound **3** (400 MHz, $CDCl_3$)

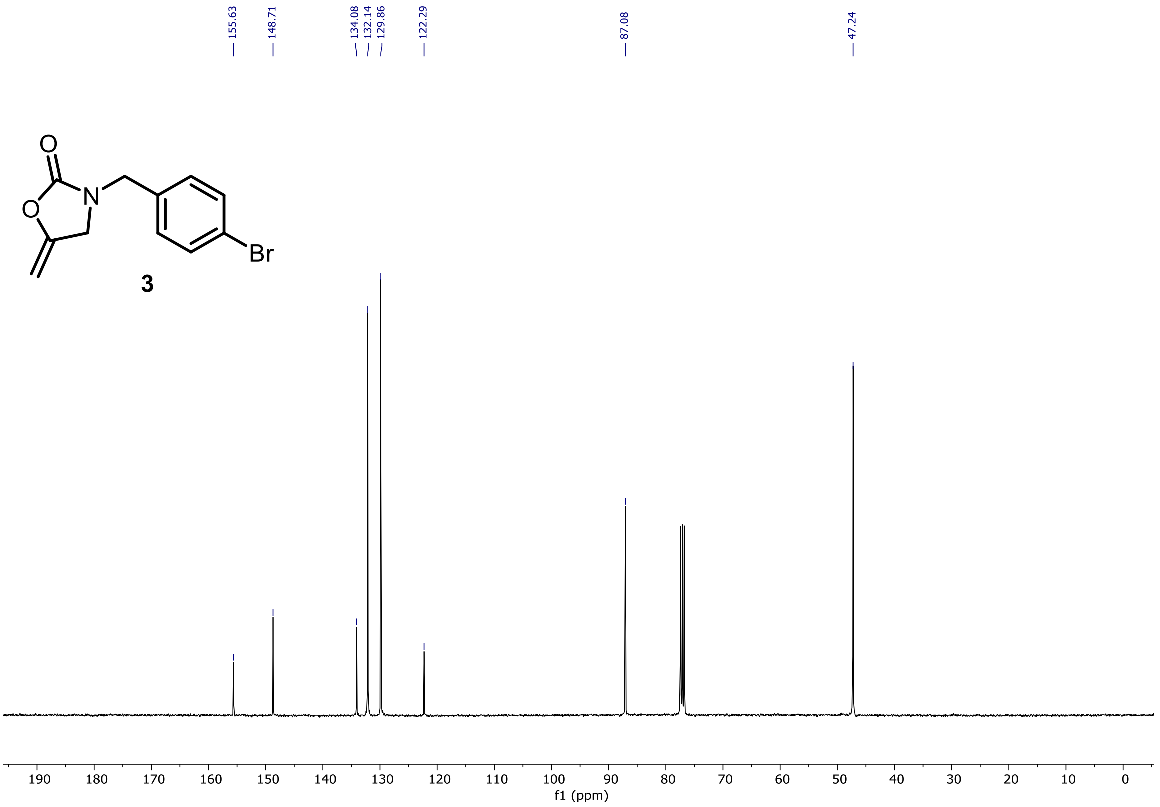

$^{13}C\{^{1}H\}$ NMR spectrum of compound **3** (101 MHz, $CDCl_3$)

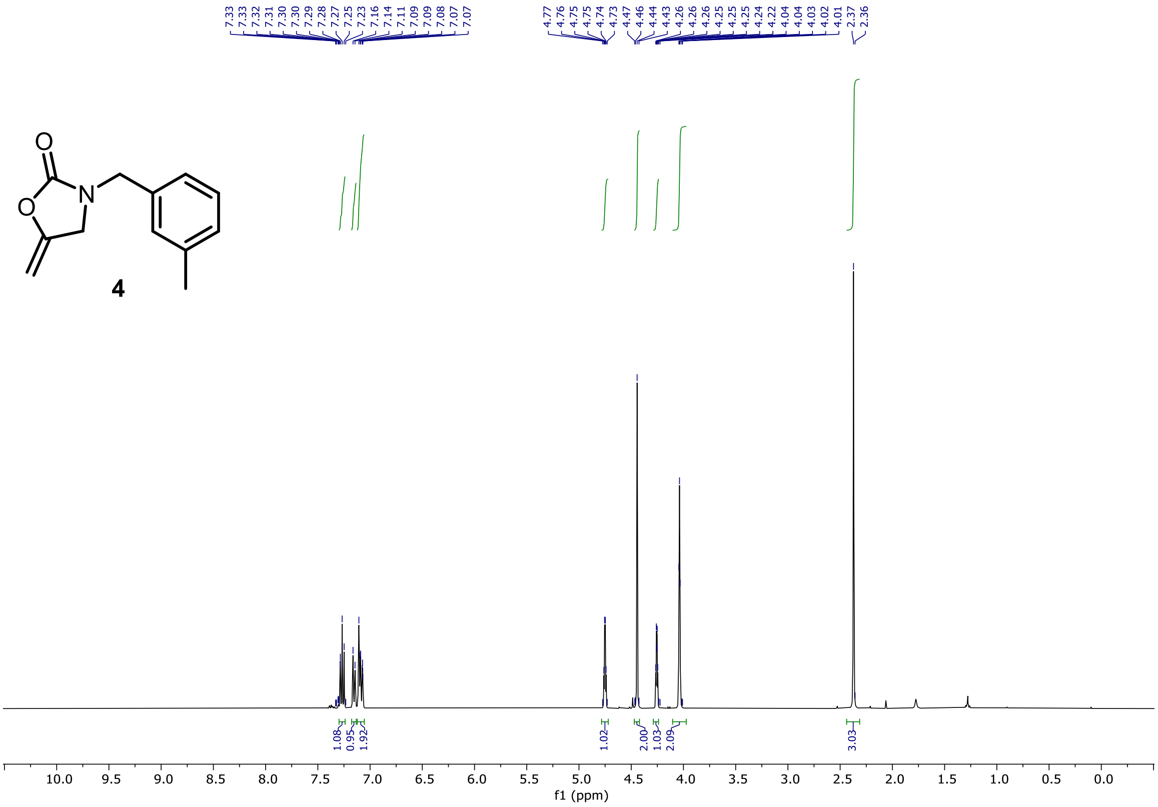

^{1}H NMR spectrum of compound **4** (400 MHz, $CDCl_3$)

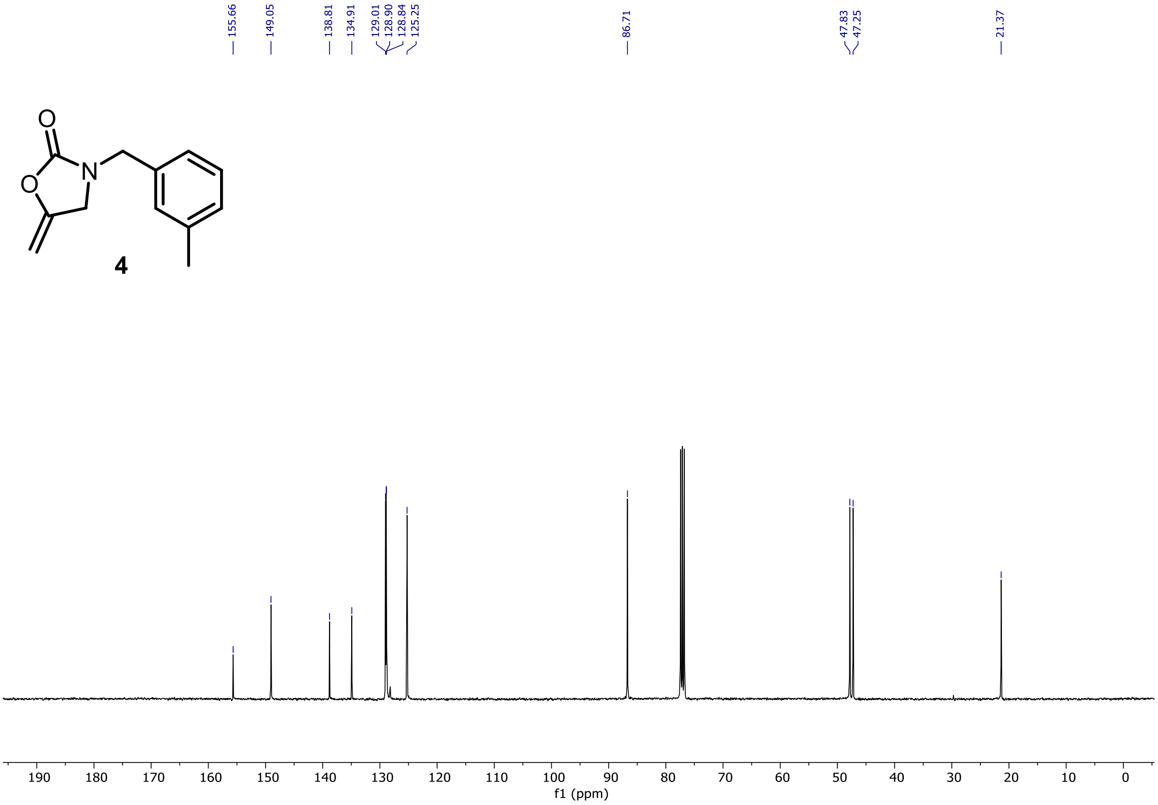

^{13}C{^{1}H} NMR spectrum of compound **4** (101 MHz, $CDCl_3$)

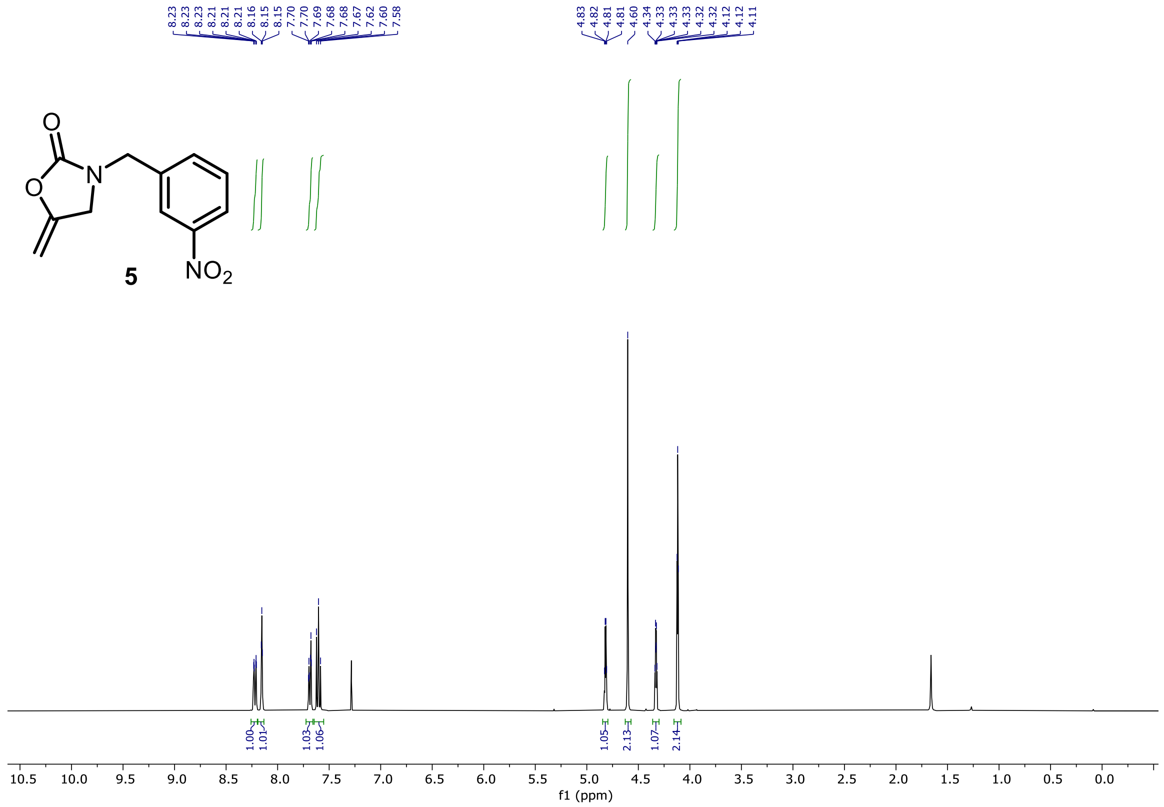

^{1}H NMR spectrum of compound **5** (400 MHz, $CDCl_3$)

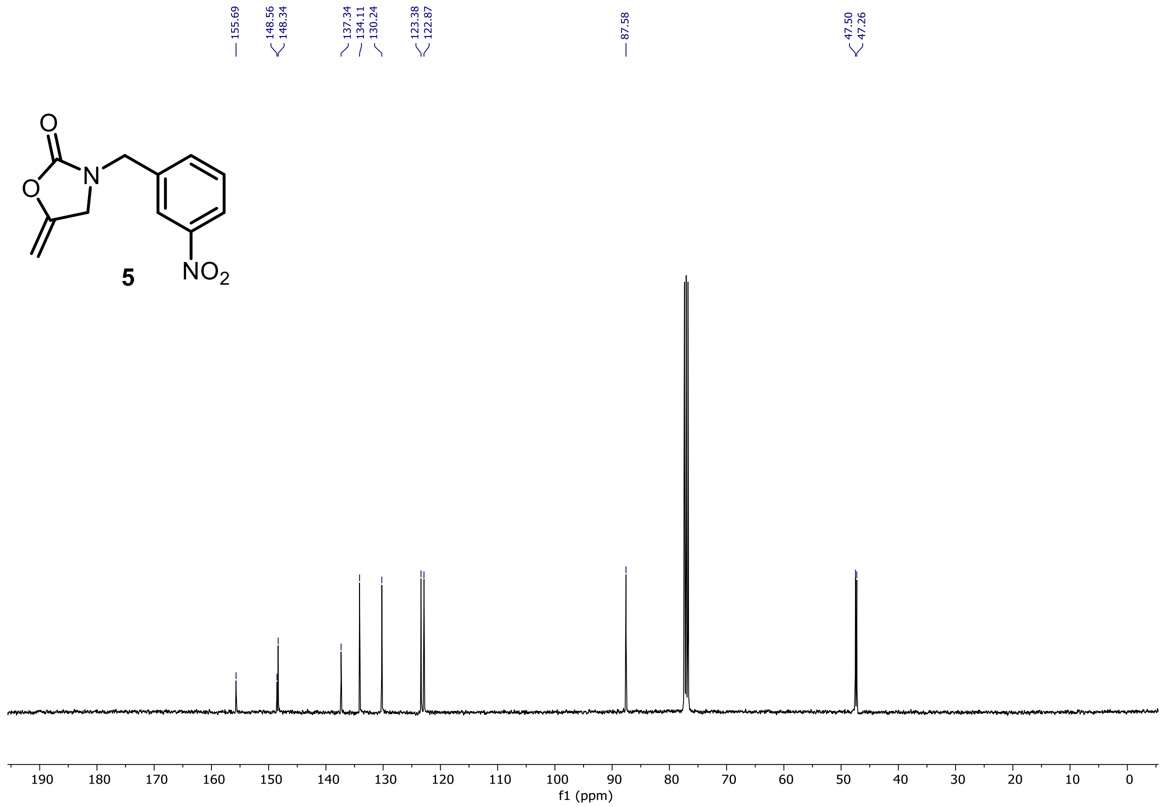

$^{13}C\{^{1}H\}$ NMR spectrum of compound **5** (101 MHz, $CDCl_3$)

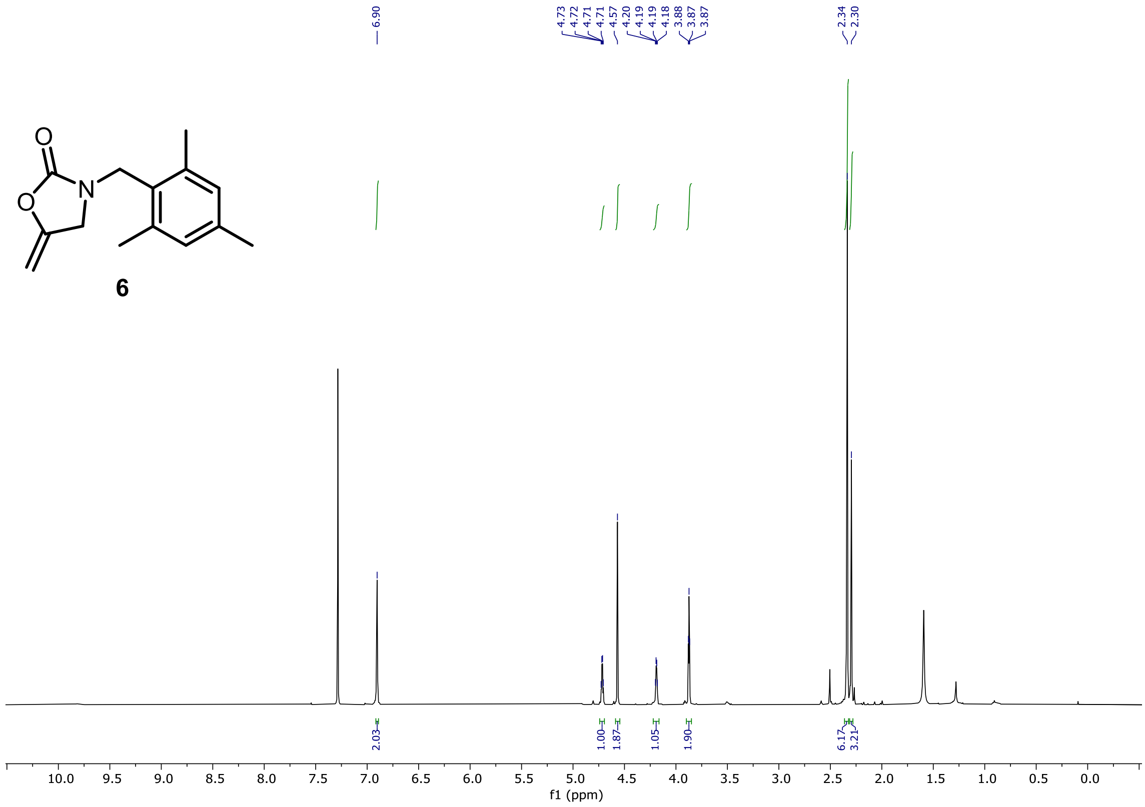

^{1}H NMR spectrum of compound **6** (400 MHz, $CDCl_3$)

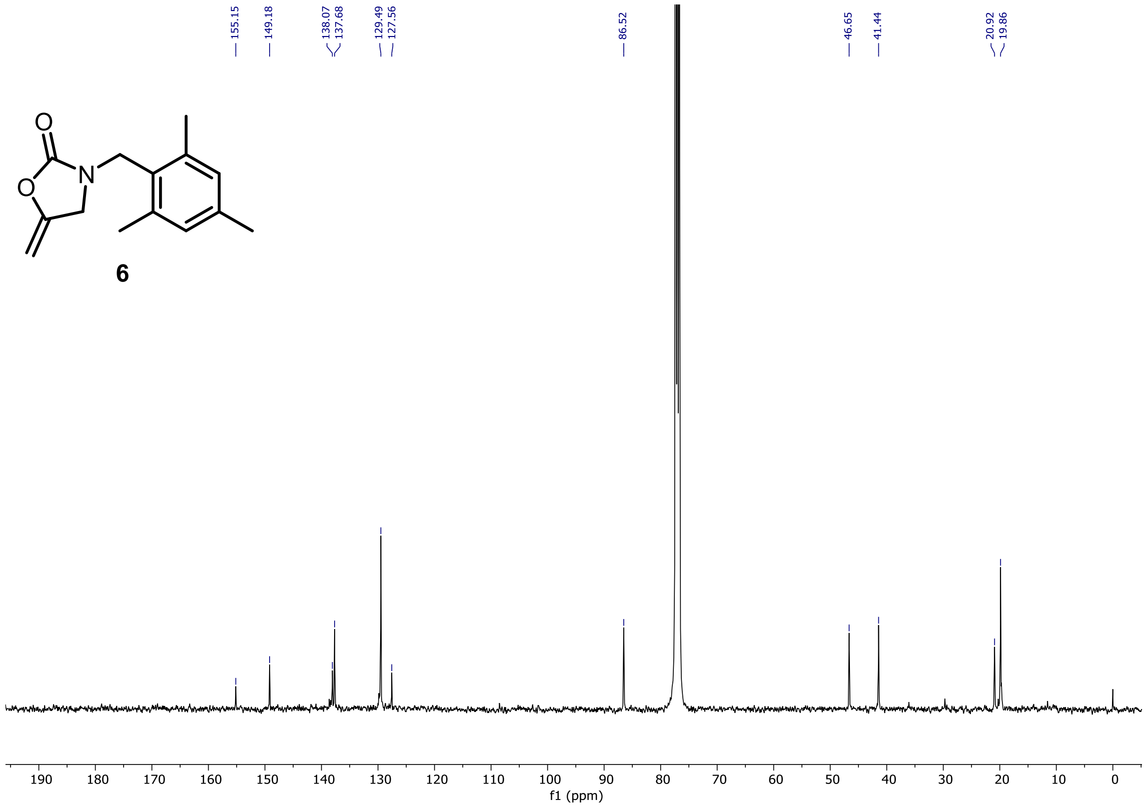

$^{13}C\{^{1}H\}$ NMR spectrum of compound **6** (101 MHz, $CDCl_3$)

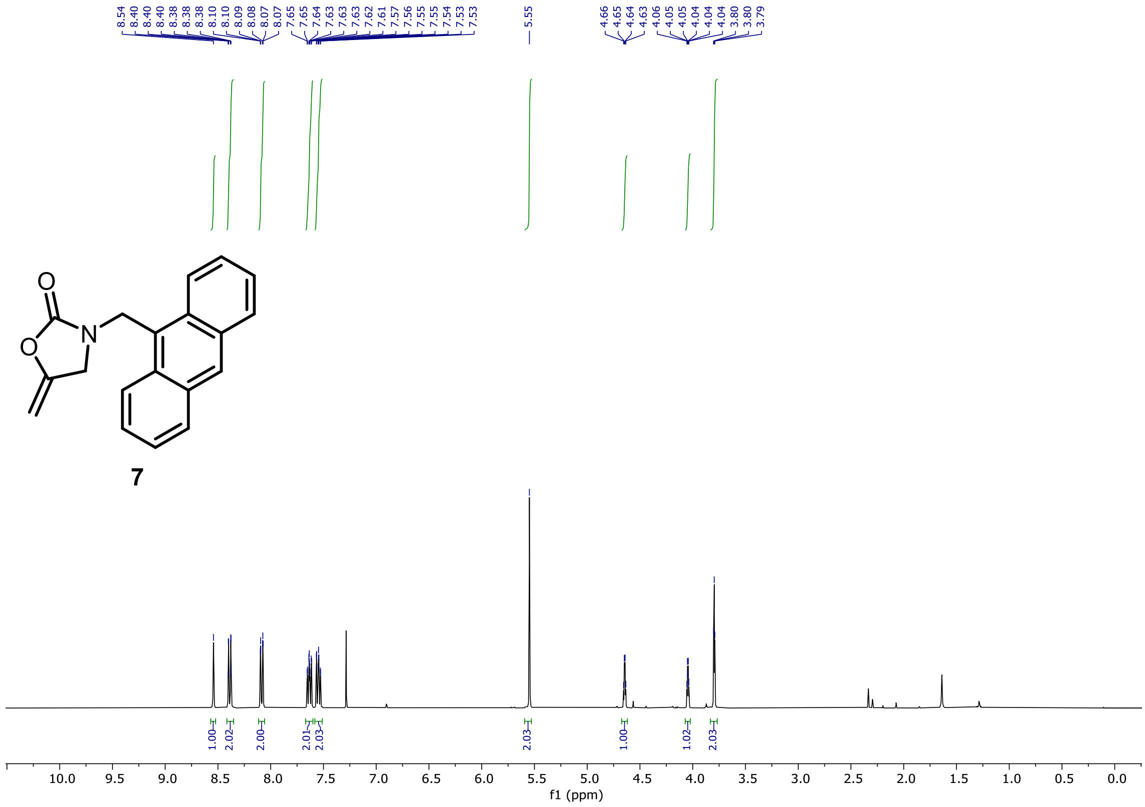

^{1}H NMR spectrum of compound **7** (400 MHz, $CDCl_3$)

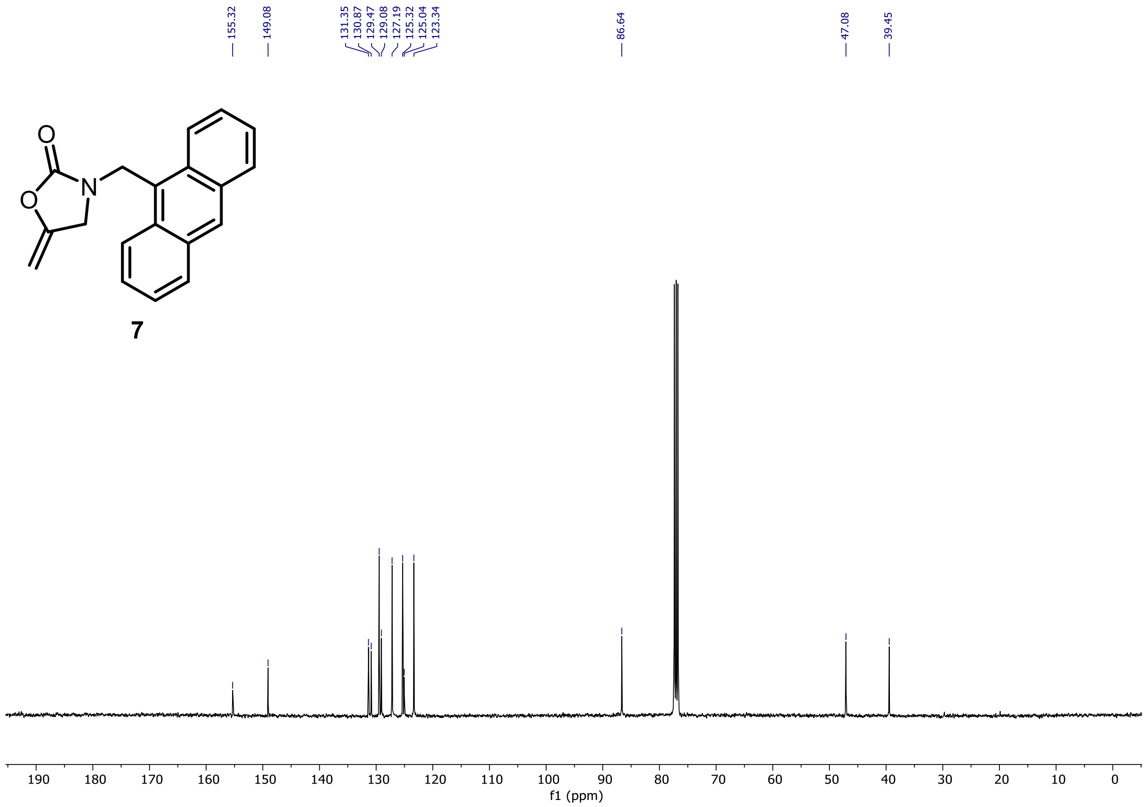

^{13}C{^{1}H} NMR spectrum of compound **7** (101 MHz, $CDCl_3$)

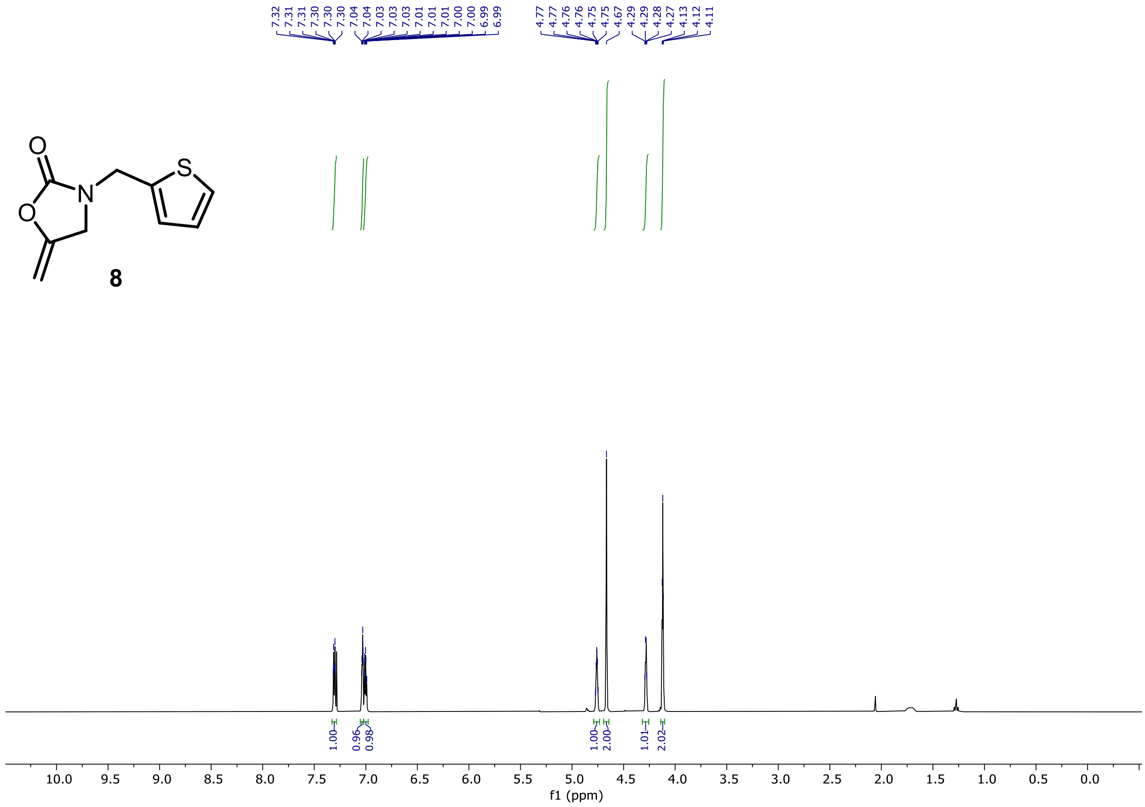

^{1}H NMR spectrum of compound **8** (400 MHz, $CDCl_3$)

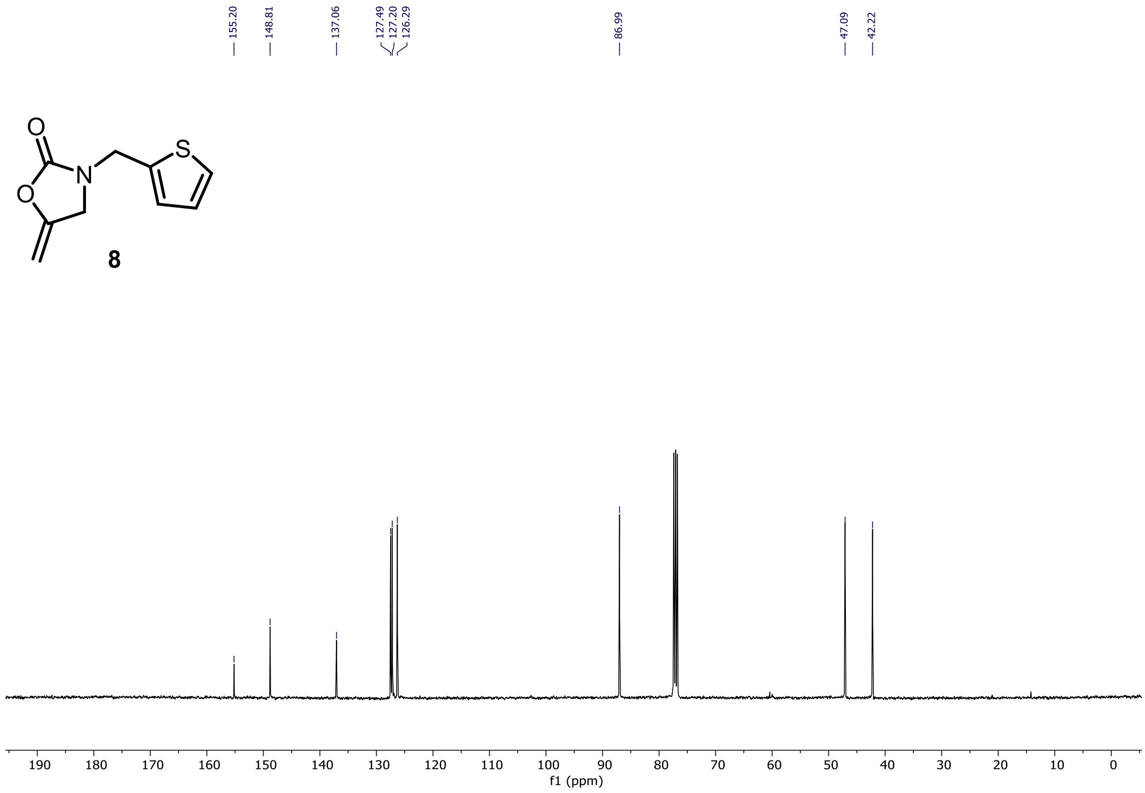

^{13}C{^{1}H} NMR spectrum of compound **8** (101 MHz, $CDCl_3$)

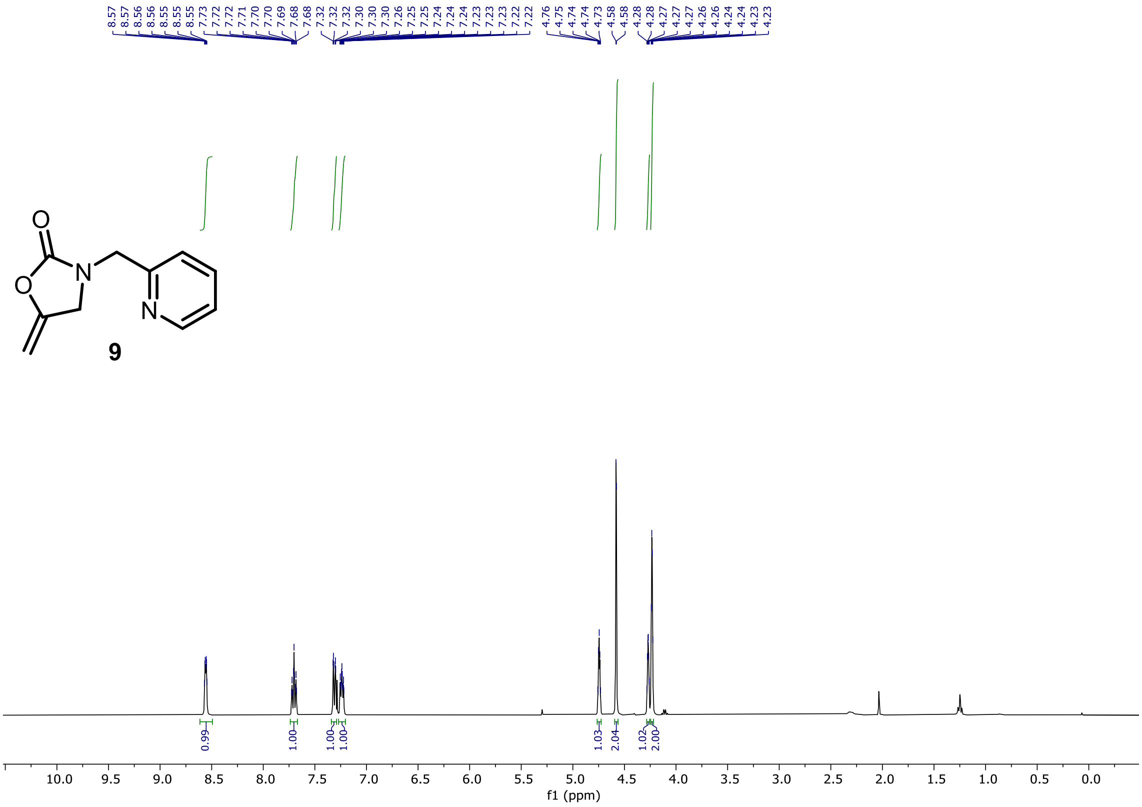

^1H NMR spectrum of compound **9** (400 MHz, $CDCl_3$)

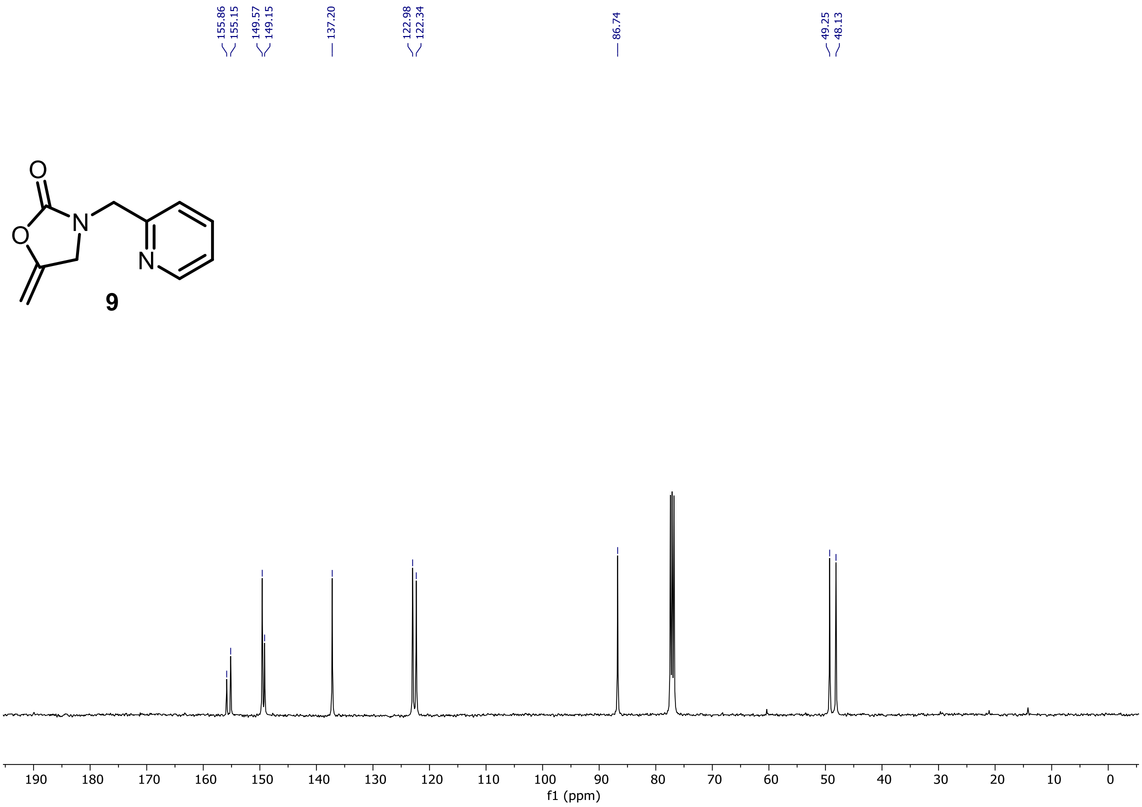

$^{13}C\{^1H\}$ NMR spectrum of compound **9** (101 MHz, $CDCl_3$)

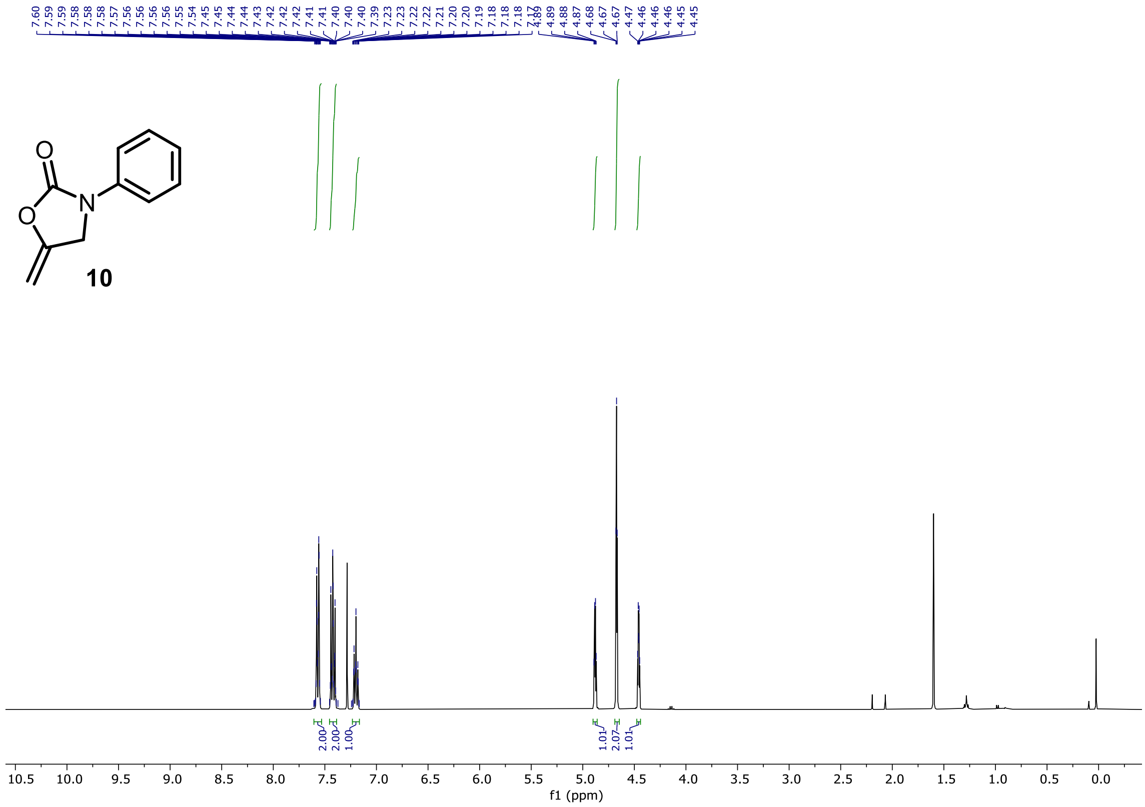

^1H NMR spectrum of compound **10** (400 MHz, $CDCl_3$)

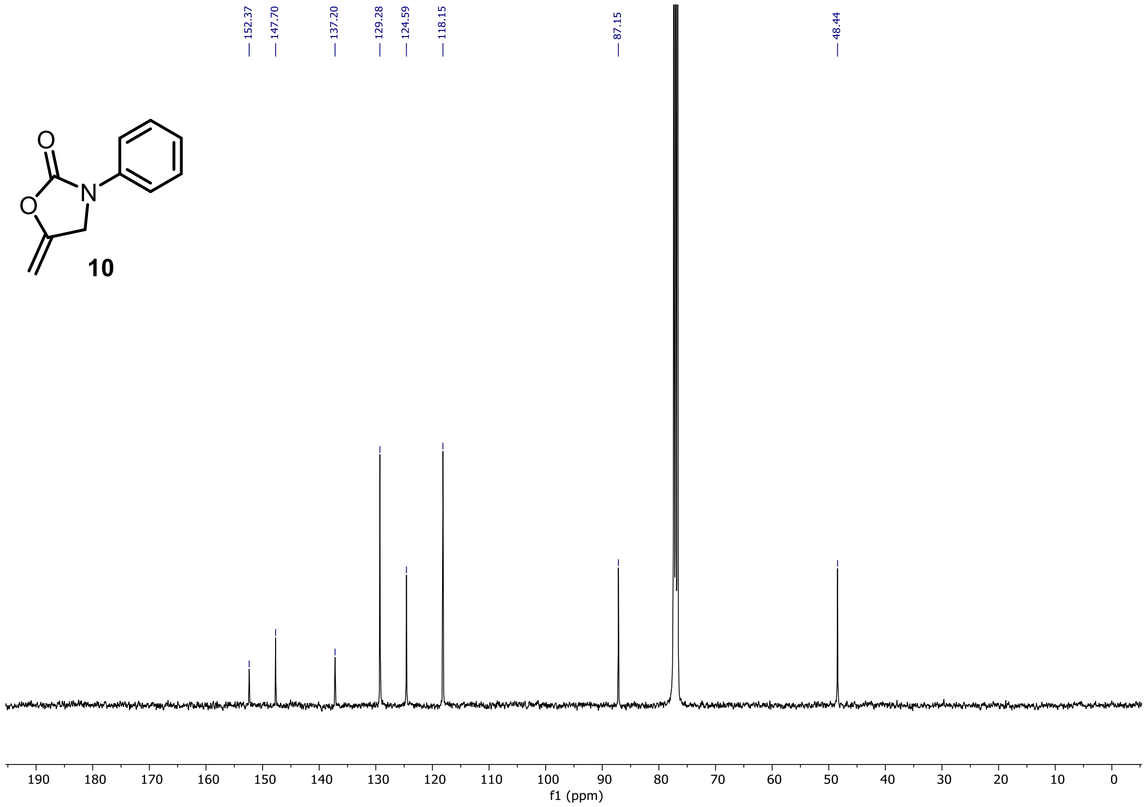

$^{13}C\{^1H\}$ NMR spectrum of compound **10** (101 MHz, $CDCl_3$)

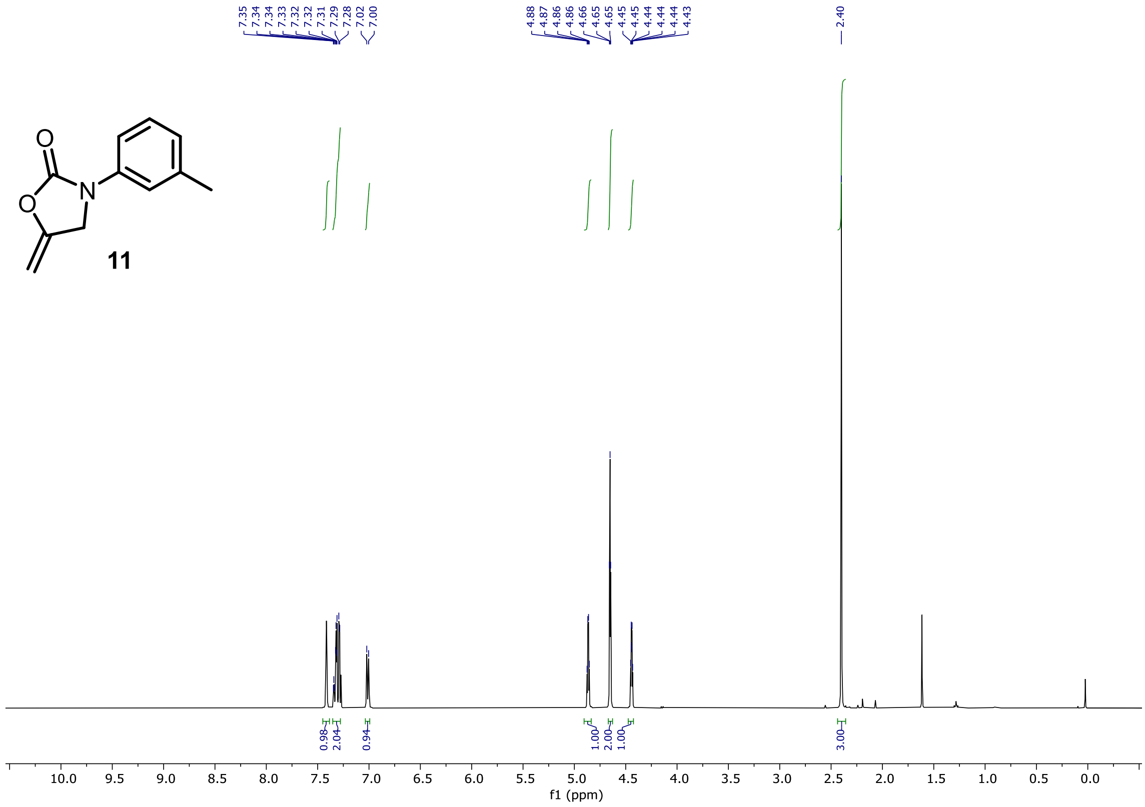

^{1}H NMR spectrum of compound **11** (400 MHz, $CDCl_3$)

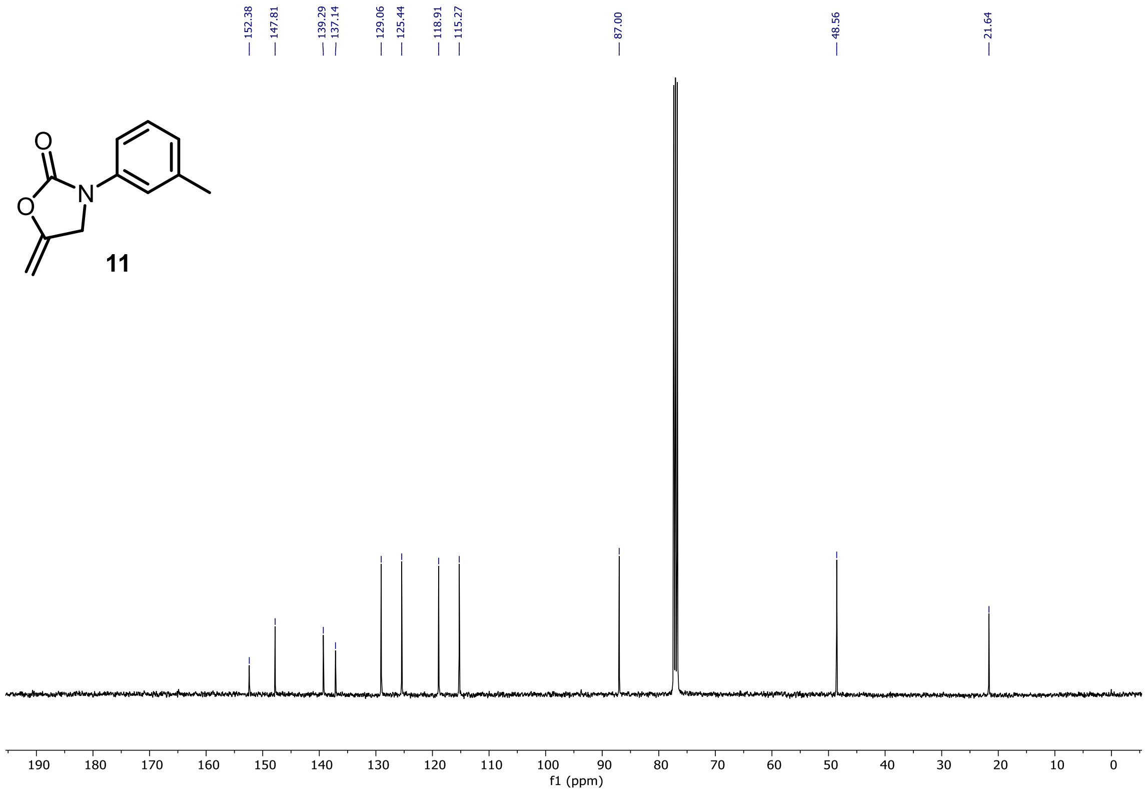

^{13}C{^{1}H} NMR spectrum of compound **11** (101 MHz, $CDCl_3$)

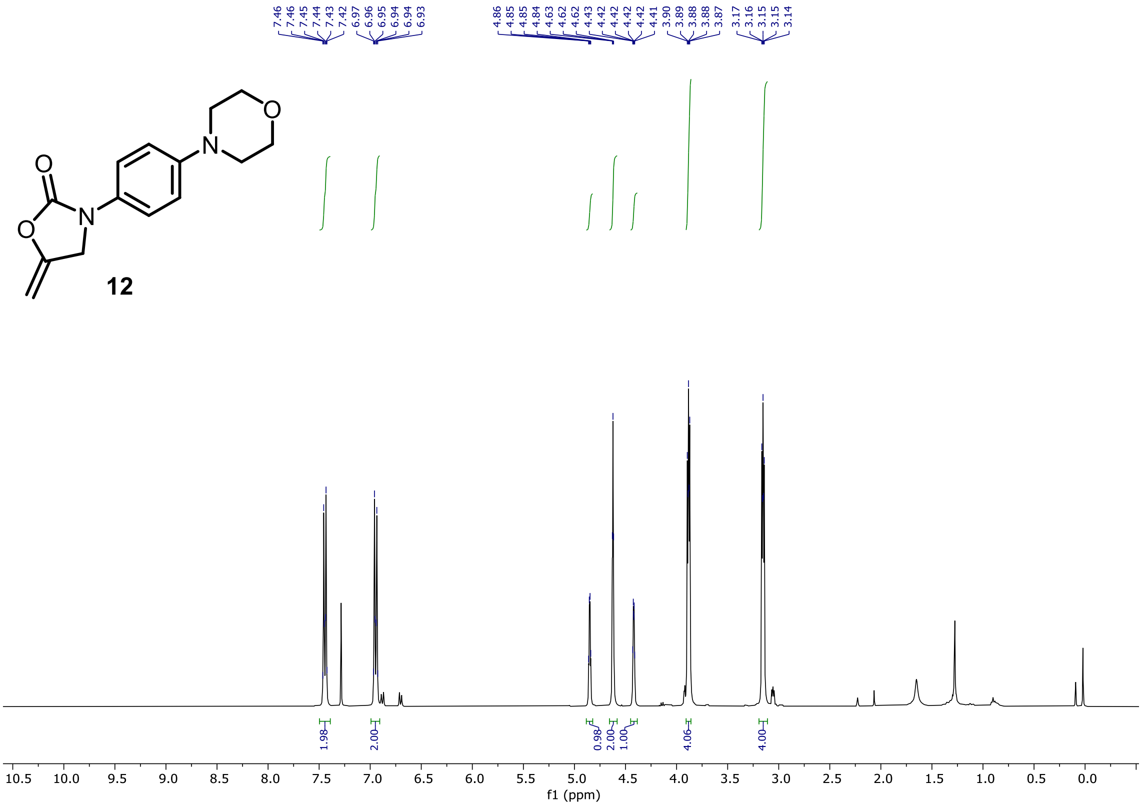

^{1}H NMR spectrum of compound **12** (400 MHz, $CDCl_3$)

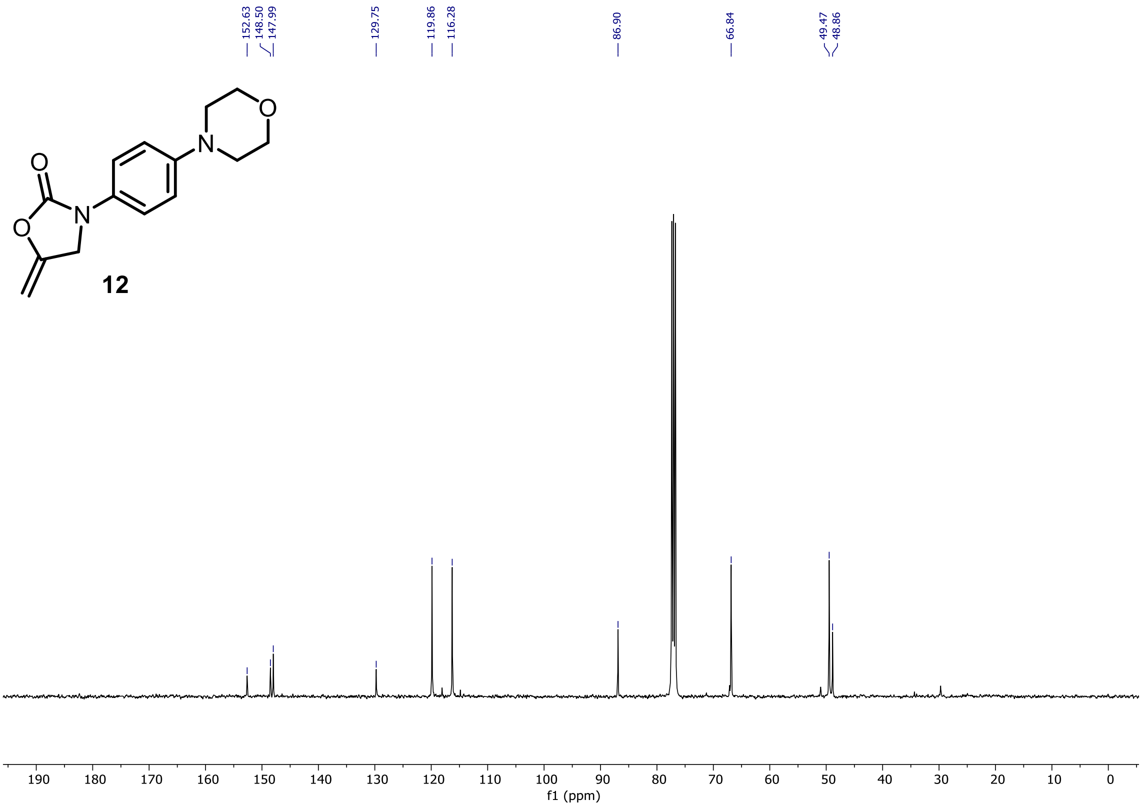

$^{13}C\{^{1}H\}$ NMR spectrum of compound **12** (101 MHz, $CDCl_3$)

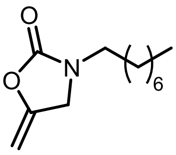

13

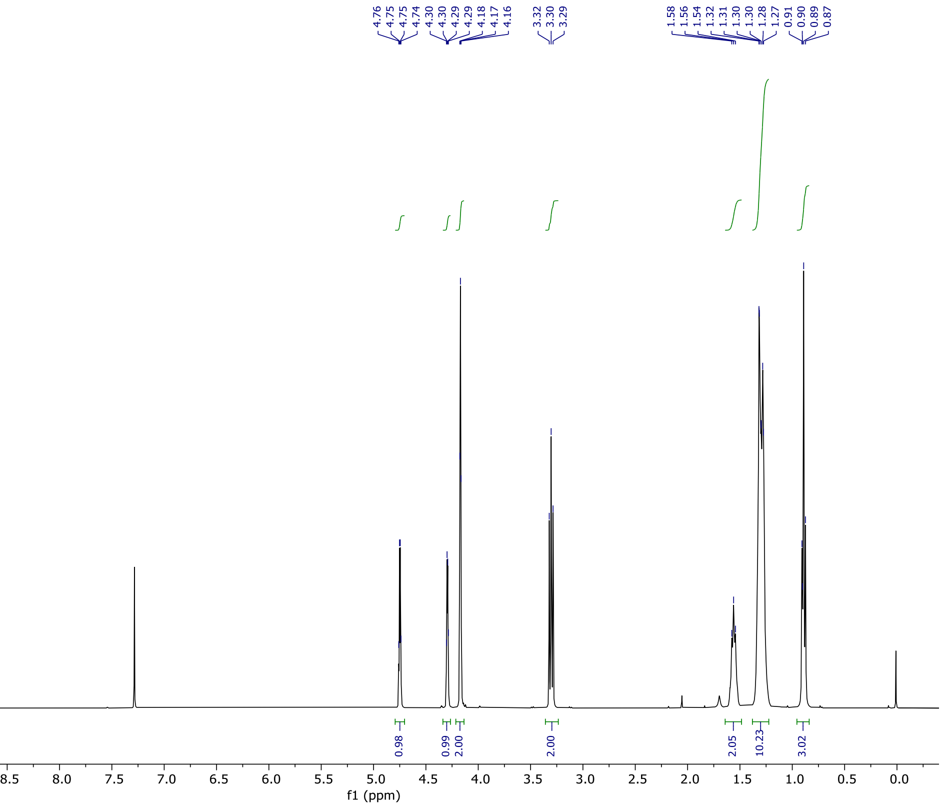

^{1}H NMR spectrum of compound **13** (400 MHz, $CDCl_3$)

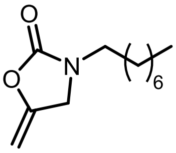

13

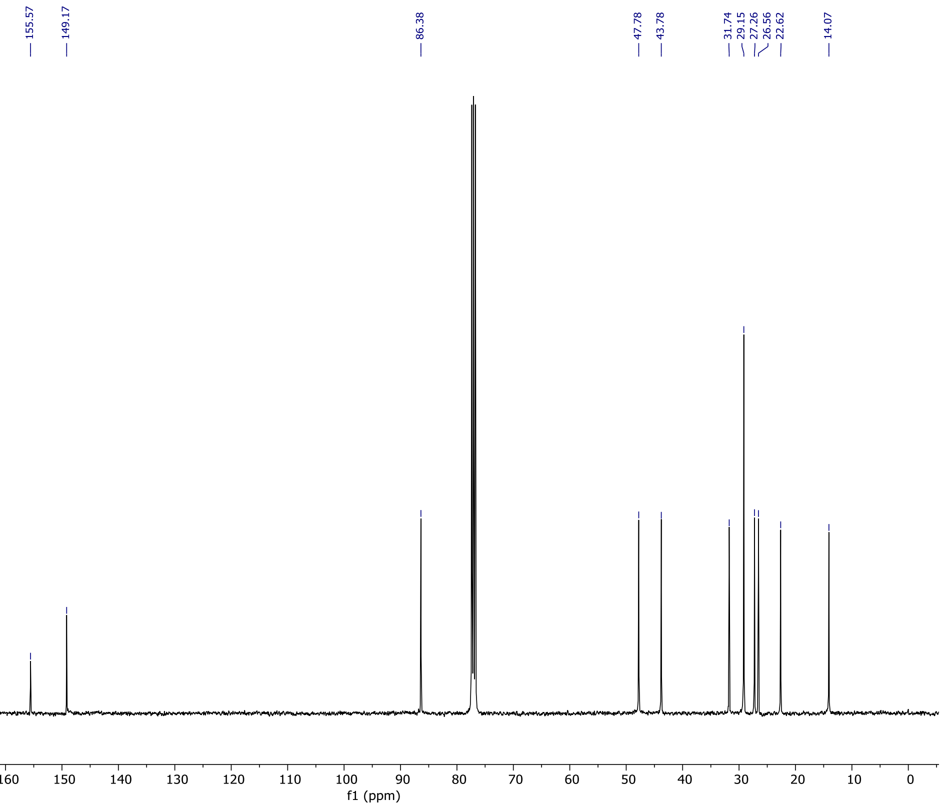

$^{13}C\{^{1}H\}$ NMR spectrum of compound **13** (101 MHz, $CDCl_3$)

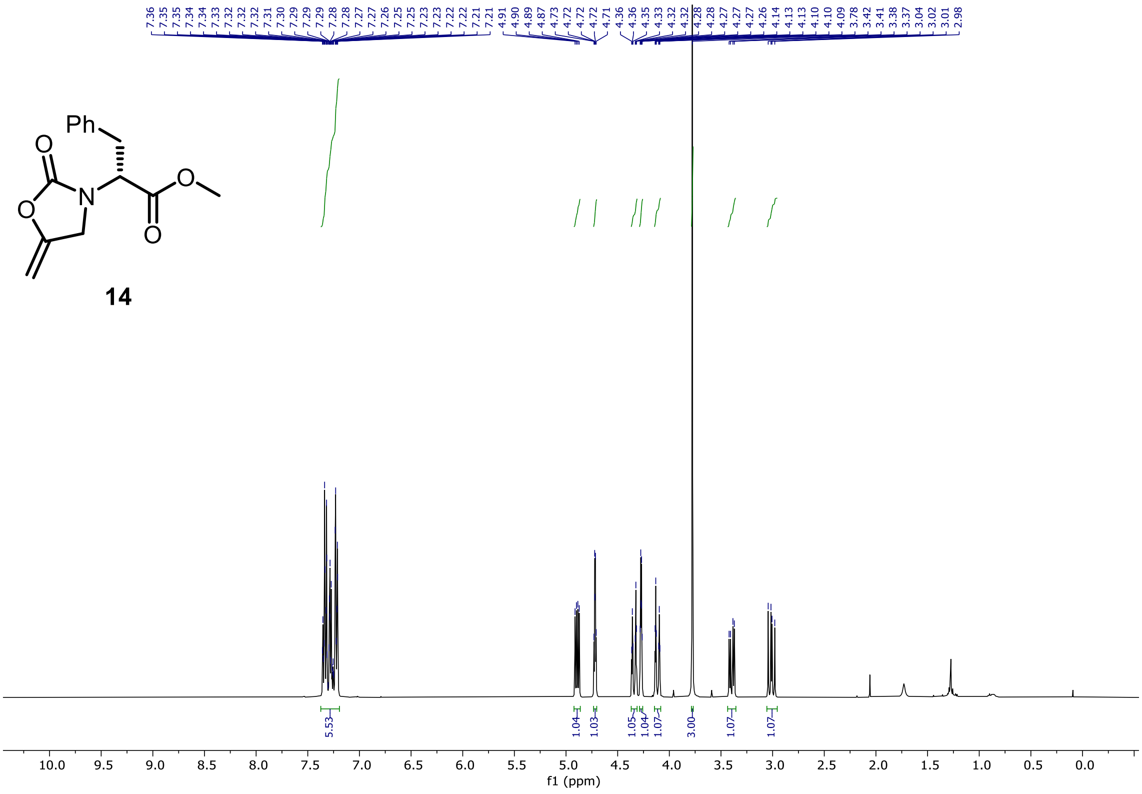

^{1}H NMR spectrum of compound **14** (400 MHz, $CDCl_3$)

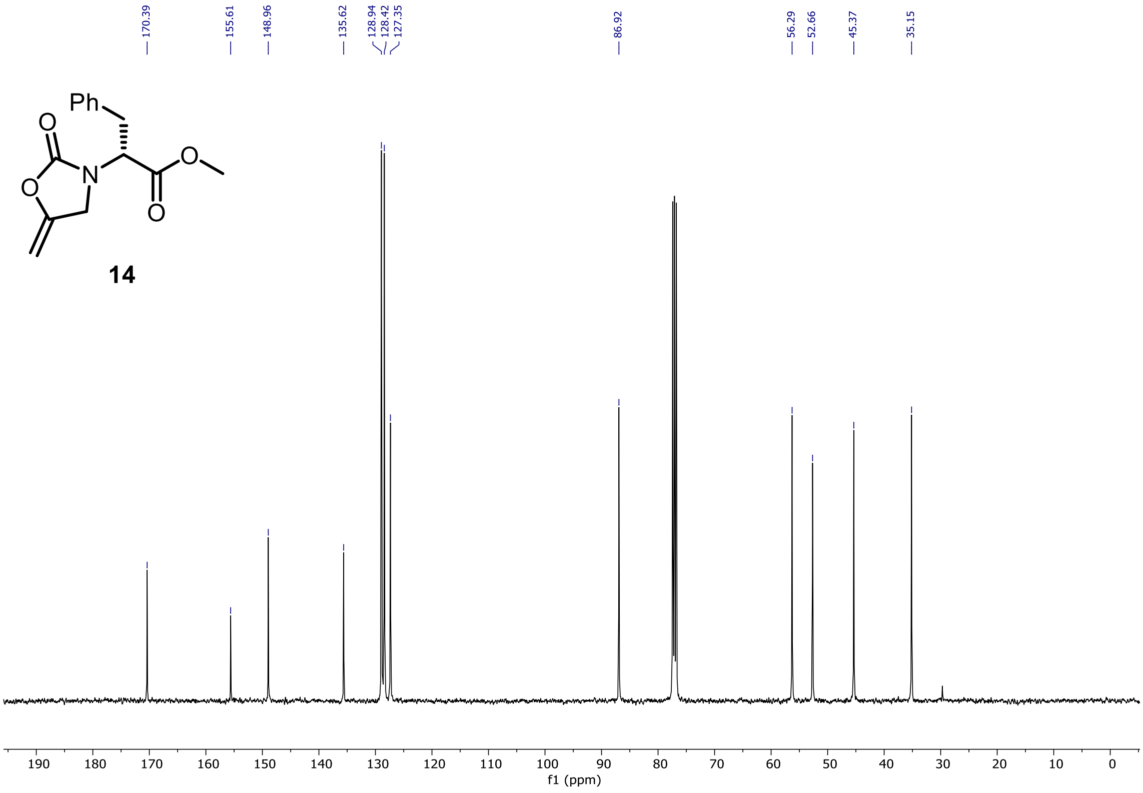

$^{13}C\{^1H\}$ NMR spectrum of compound **14** (101 MHz, $CDCl_3$)

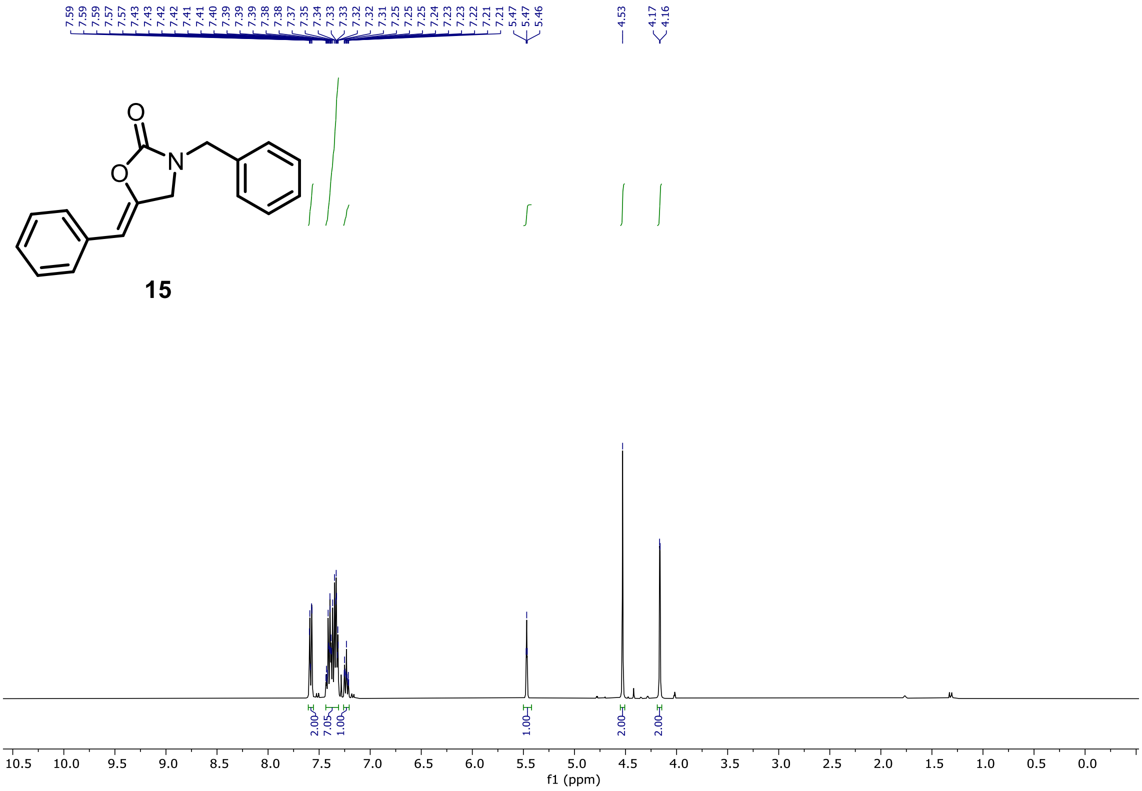

^{1}H NMR spectrum of compound **15** (400 MHz, $CDCl_3$)

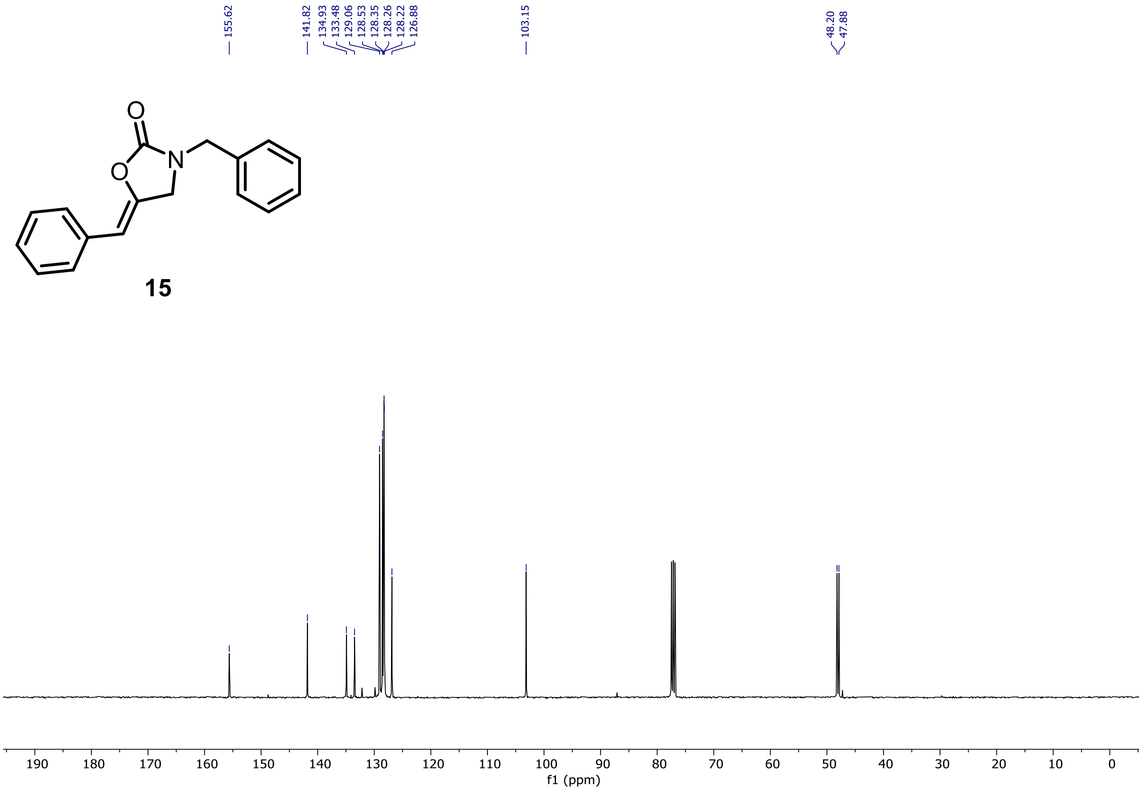

$^{13}C\{^1H\}$ NMR spectrum of compound **15** (101 MHz, $CDCl_3$)

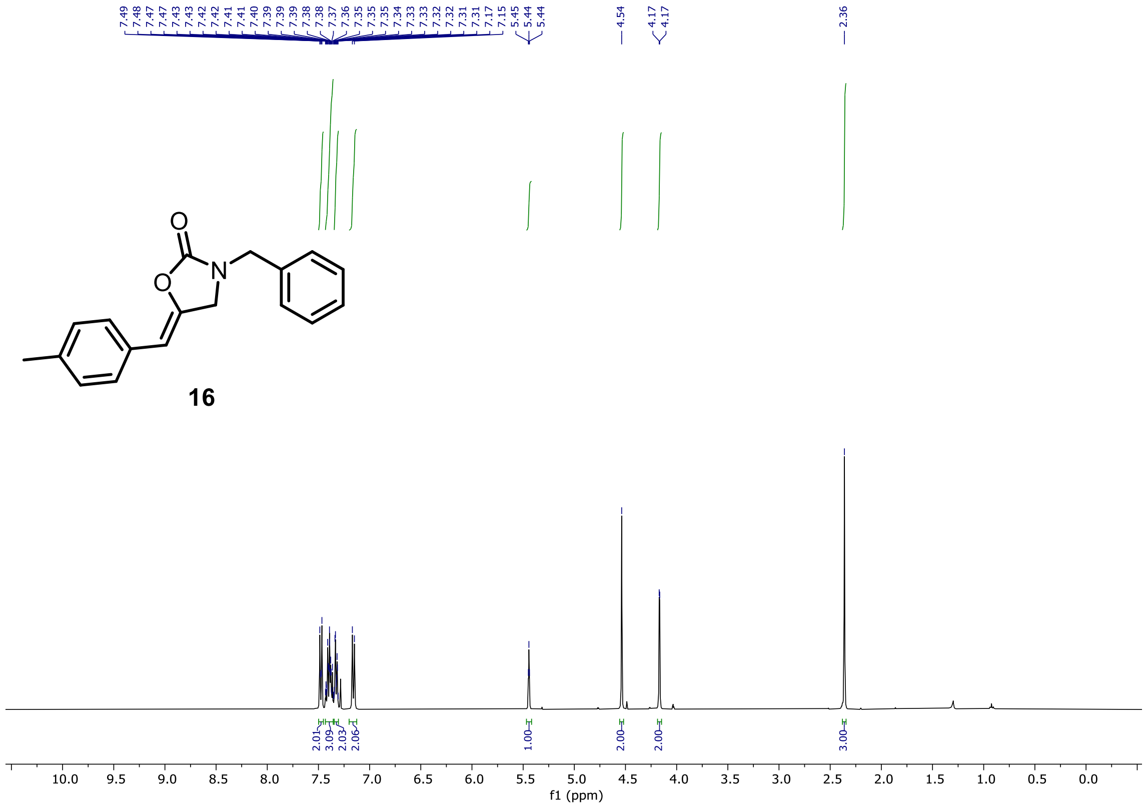

^{1}H NMR spectrum of compound **16** (400 MHz, $CDCl_3$)

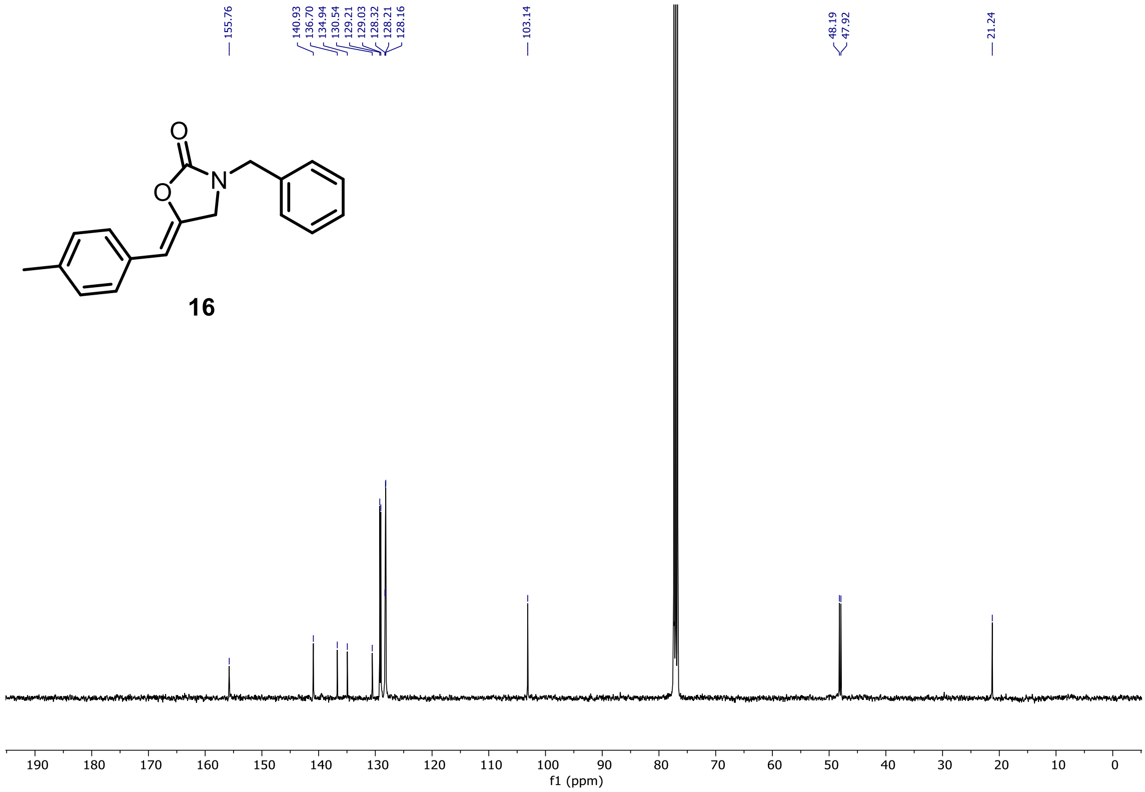

$^{13}C\{^{1}H\}$ NMR spectrum of compound **16** (101 MHz, $CDCl_3$)

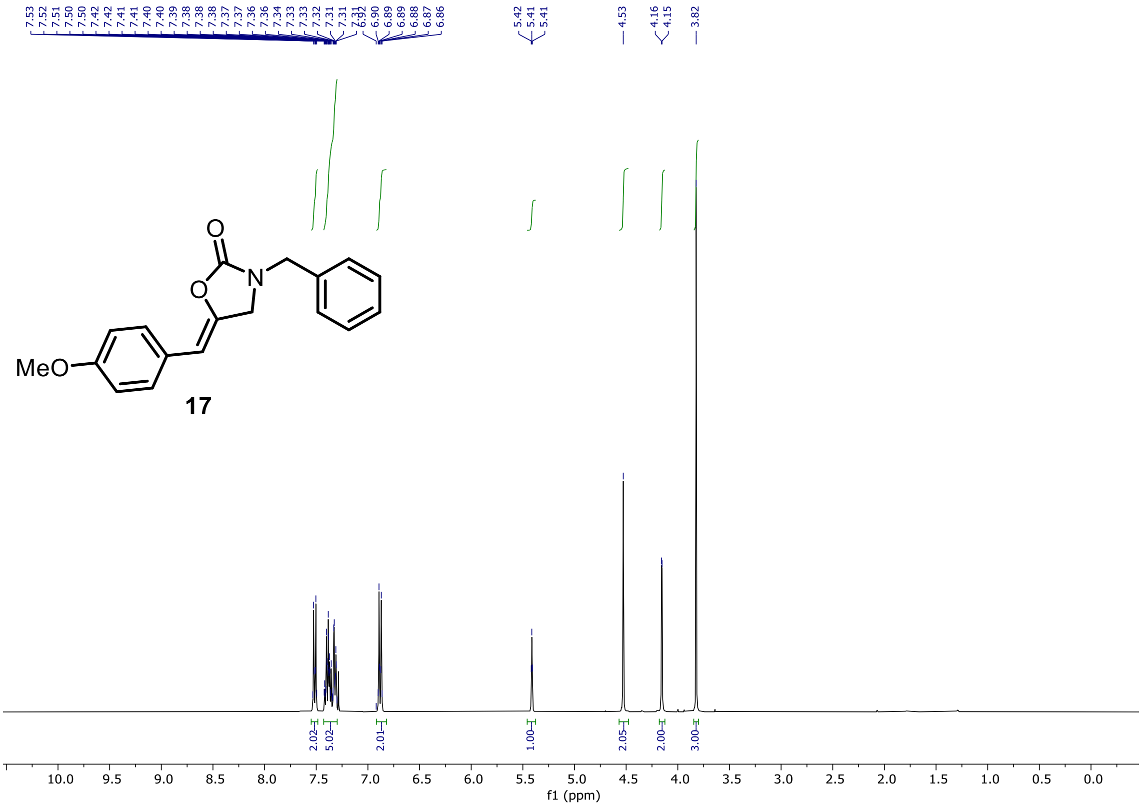

^{1}H NMR spectrum of compound **17** (400 MHz, $CDCl_3$)

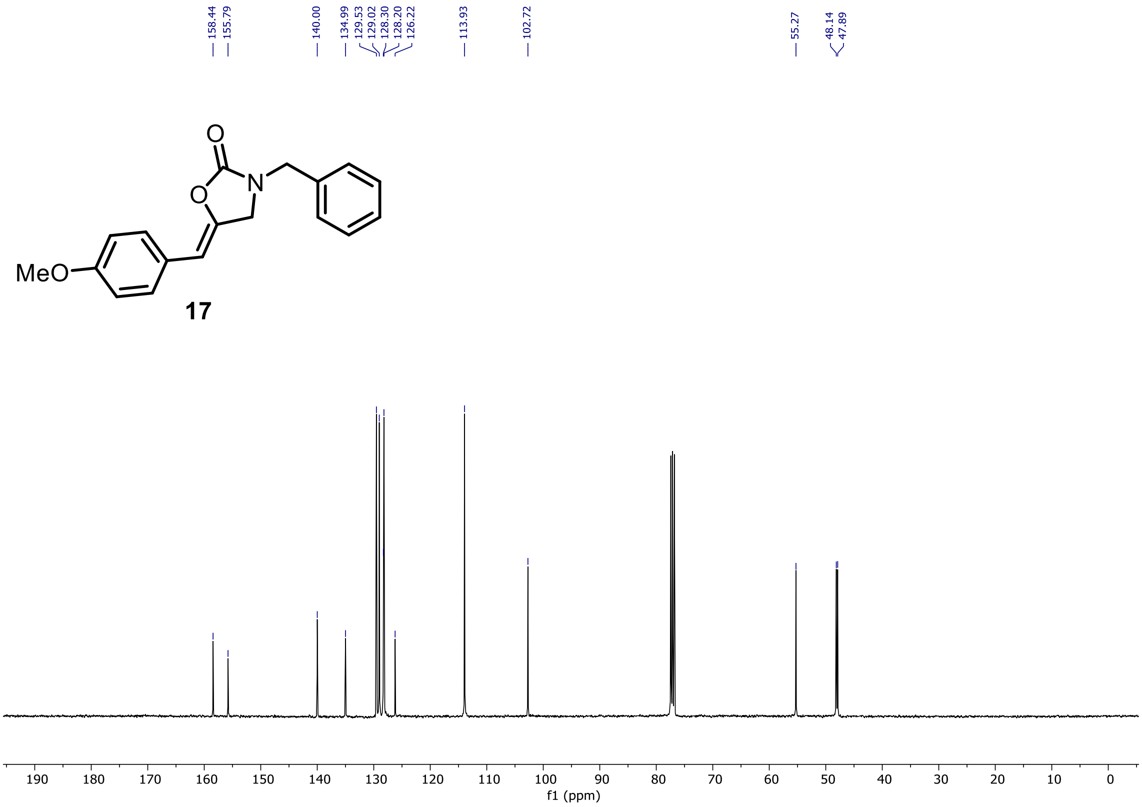

$^{13}C\{^1H\}$ NMR spectrum of compound **17** (101 MHz, $CDCl_3$)

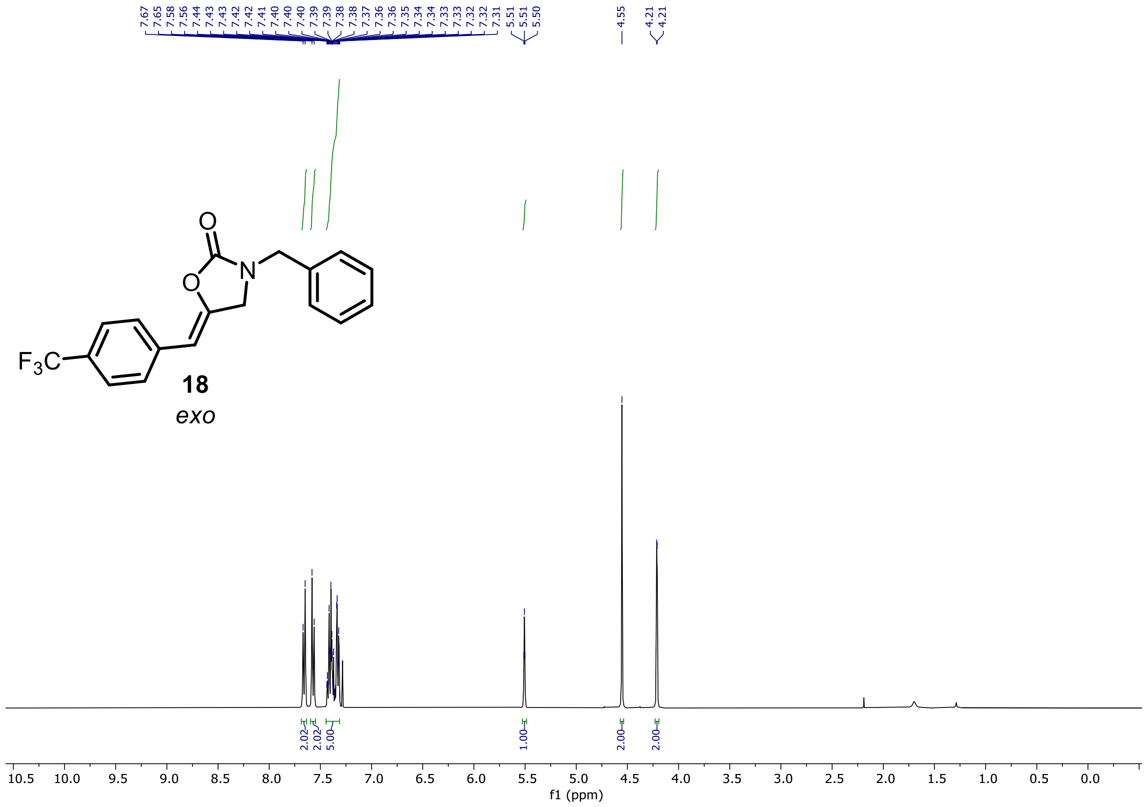

^{1}H NMR spectrum of compound **18 exo** (400 MHz, $CDCl_3$)

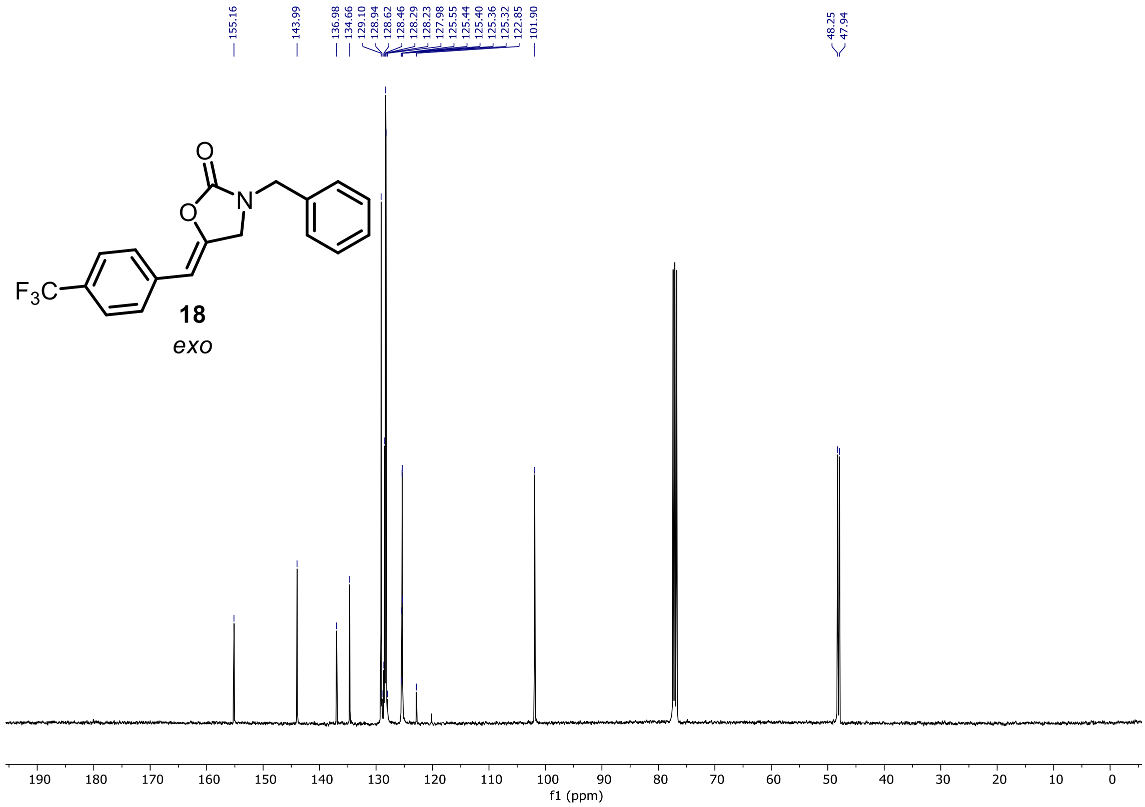

$^{13}C\{^{1}H\}$ NMR spectrum of compound **18 exo** (101 MHz, $CDCl_3$)

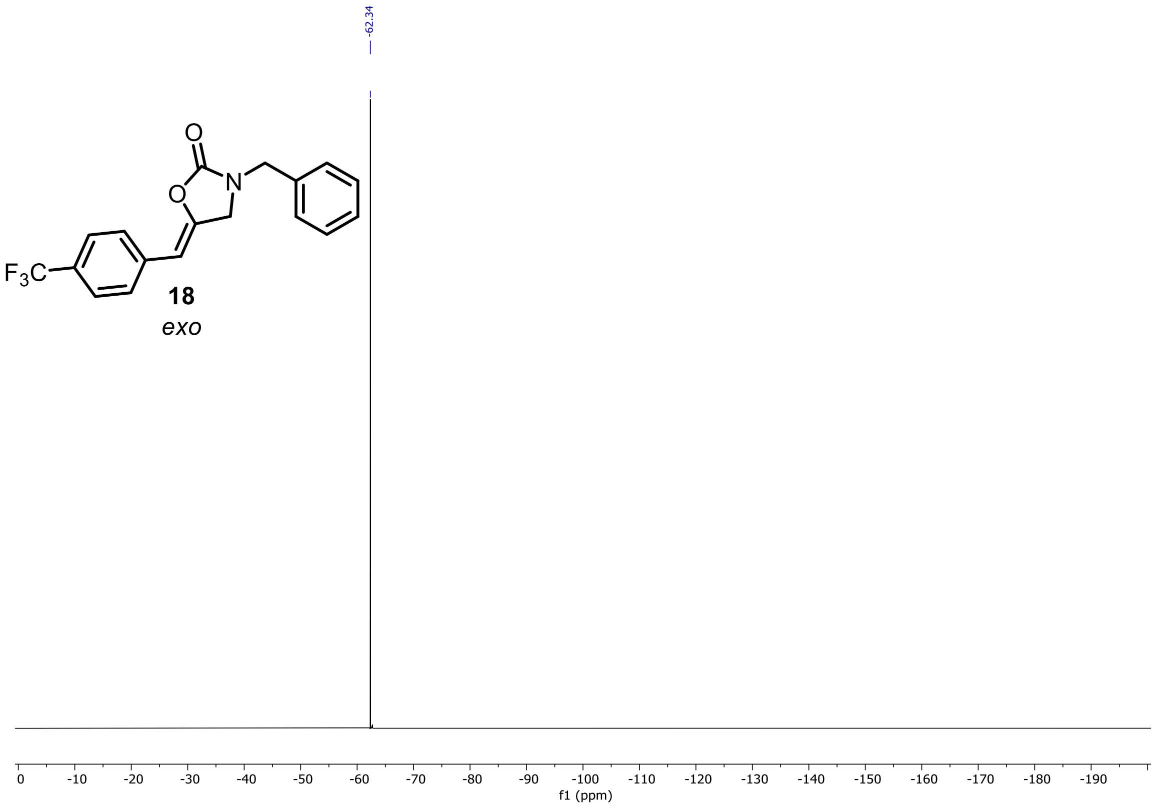

^{19}F NMR spectrum of compound **18 exo** (565 MHz, $CDCl_3$)

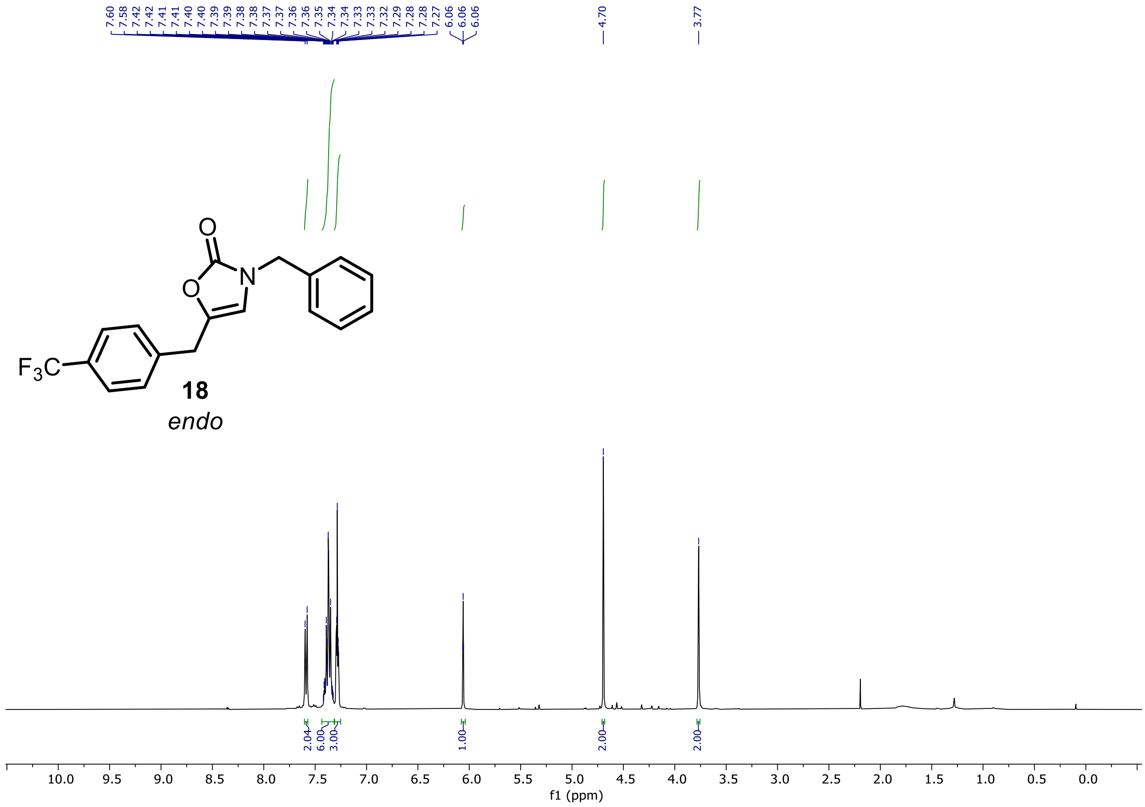

^{1}H NMR spectrum of compound **18 endo** (400 MHz, $CDCl_3$)

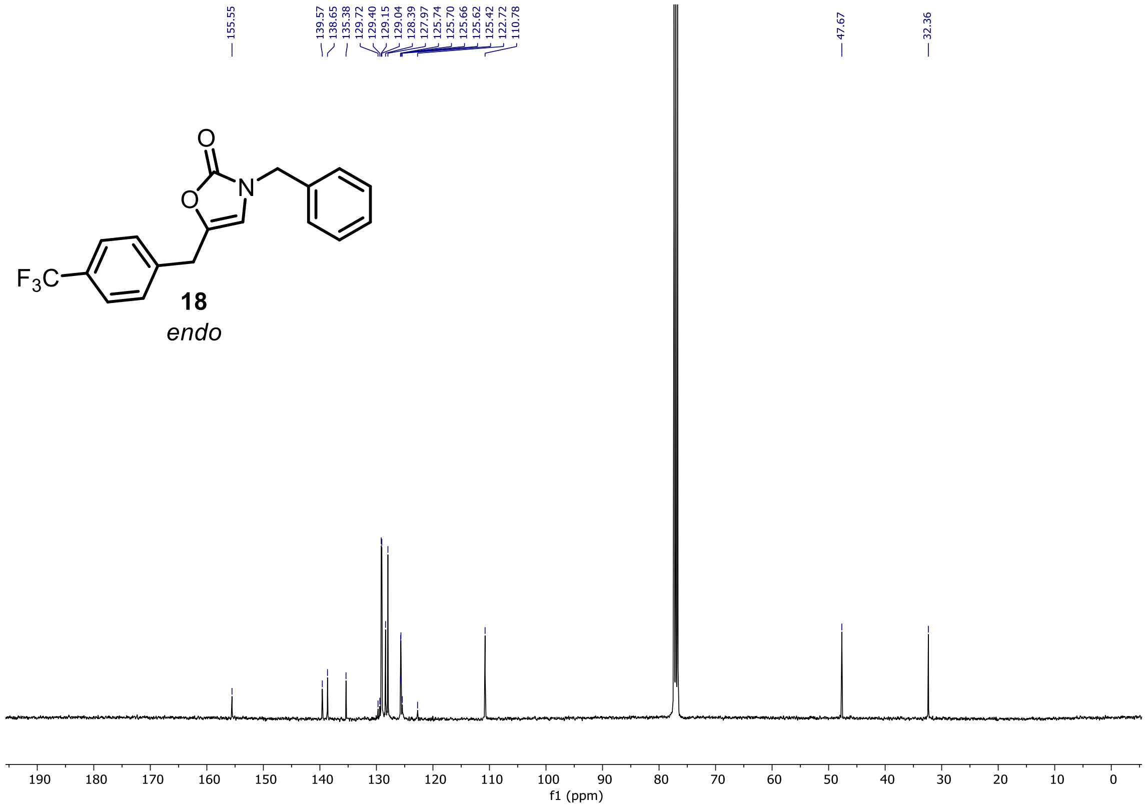

$^{13}C\{^{1}H\}$ NMR spectrum of compound **18 endo** (101 MHz, $CDCl_3$)

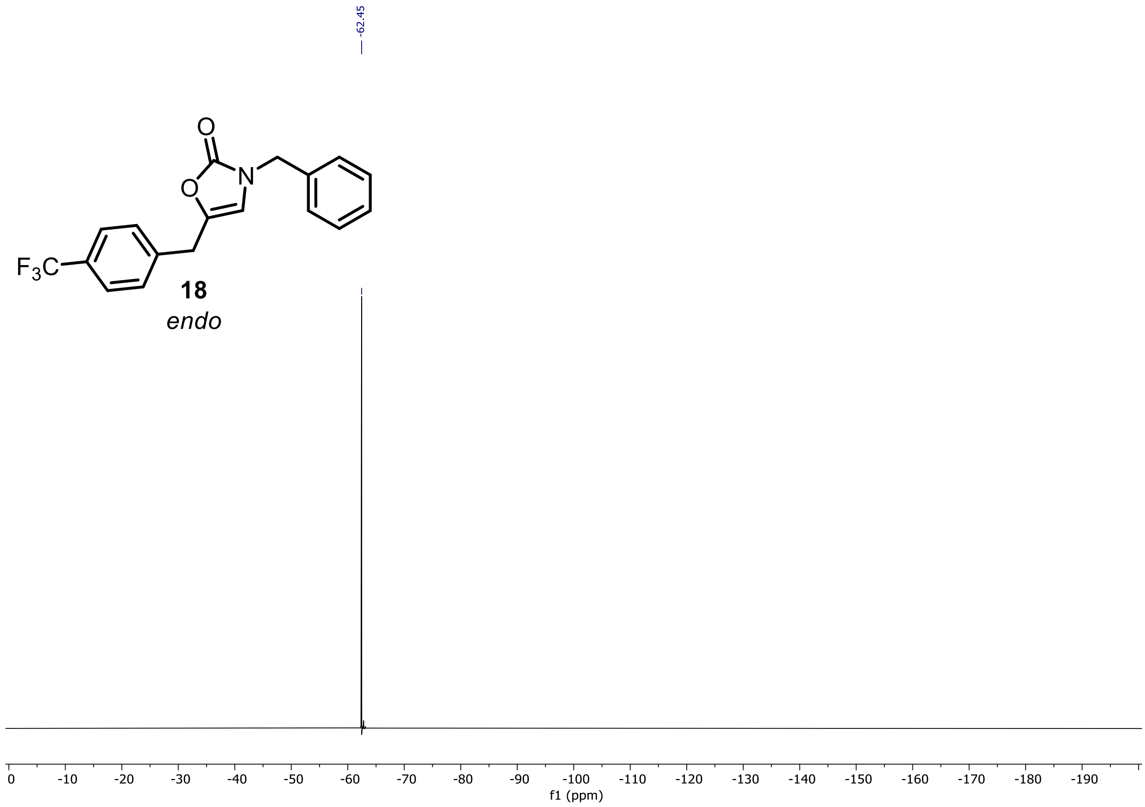

^{19}F NMR spectrum of compound **18 endo** (565 MHz, $CDCl_3$)

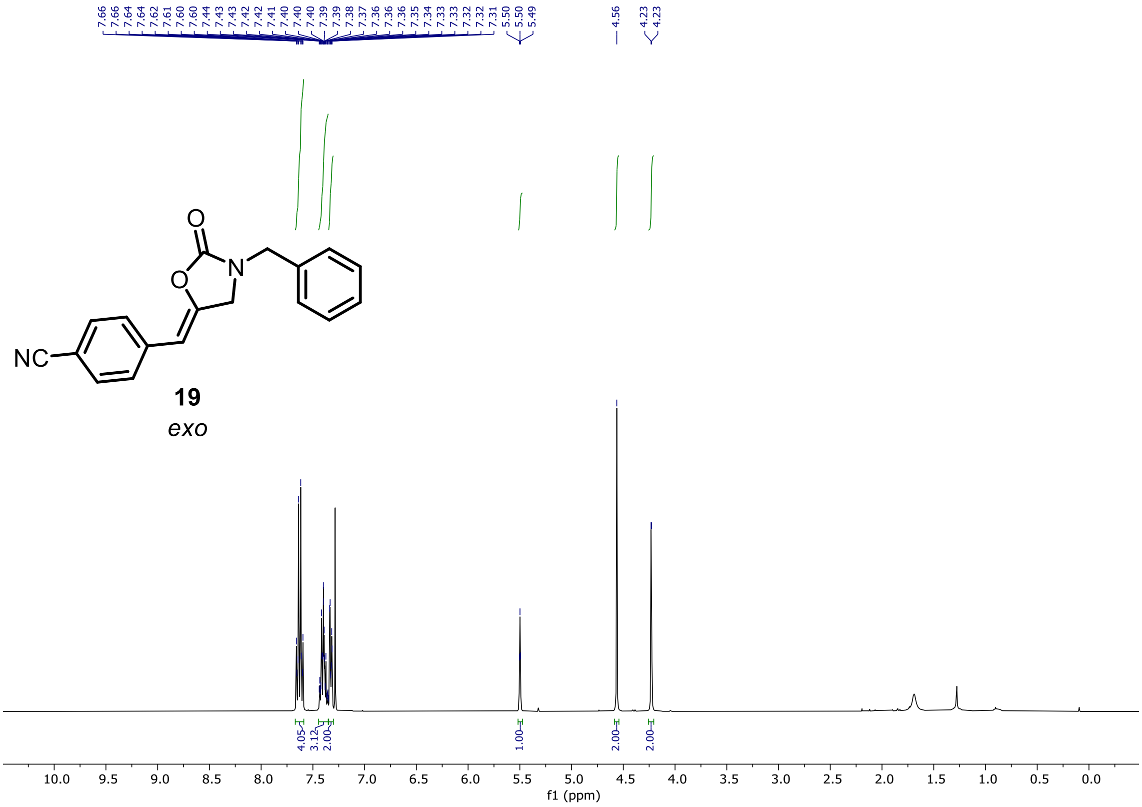

^{1}H NMR spectrum of compound **19 exo** (400 MHz, $CDCl_3$)

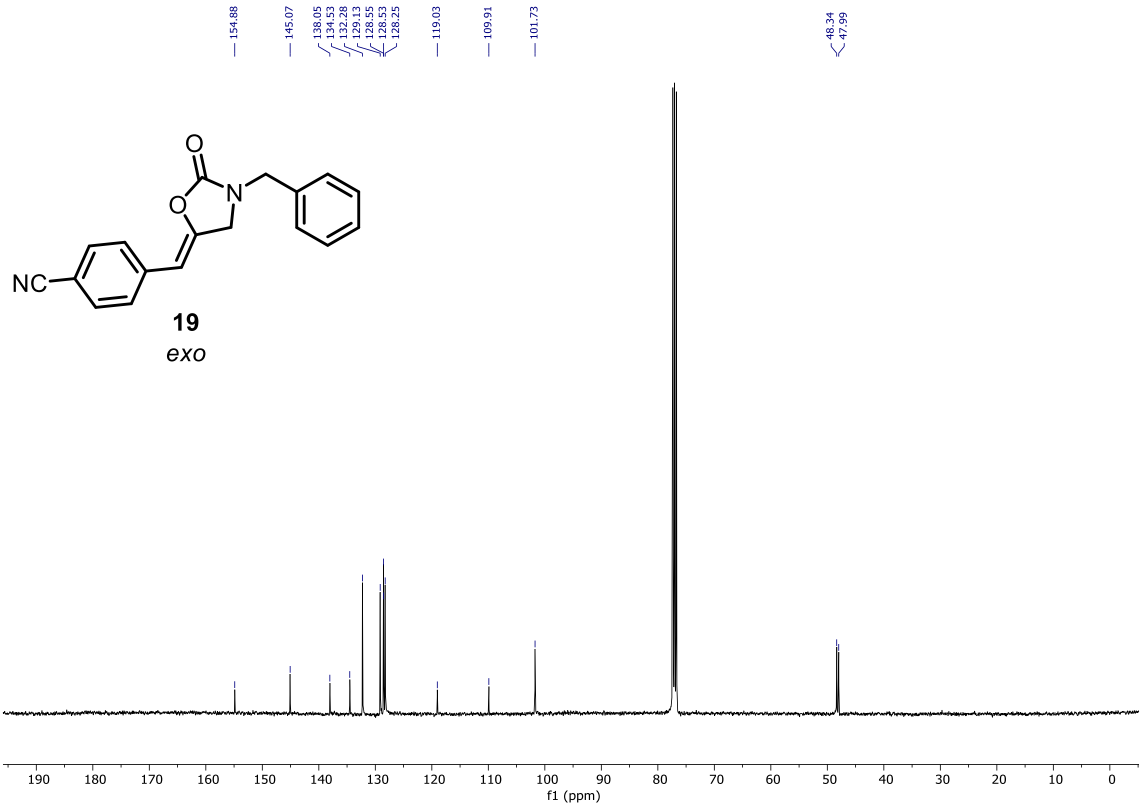

$^{13}C\{^1H\}$ NMR spectrum of compound **19 exo** (101 MHz, $CDCl_3$)

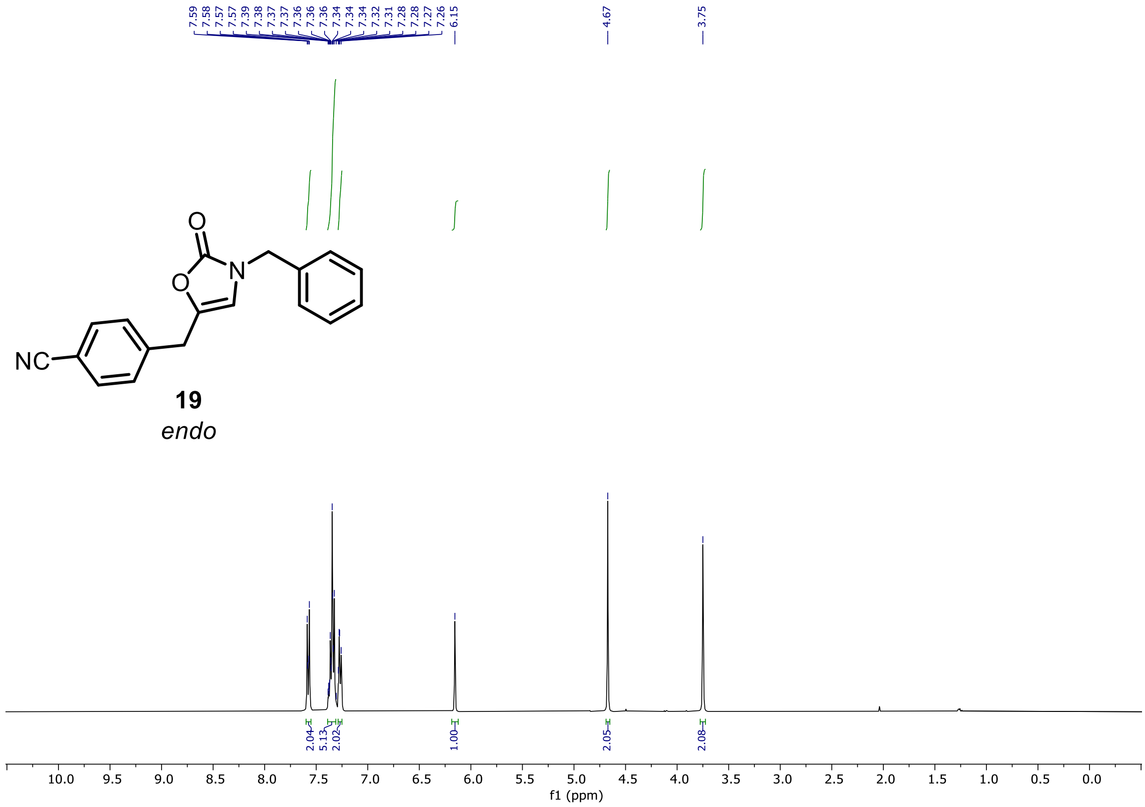

^{1}H NMR spectrum of compound **19 endo** (400 MHz, $CDCl_3$)

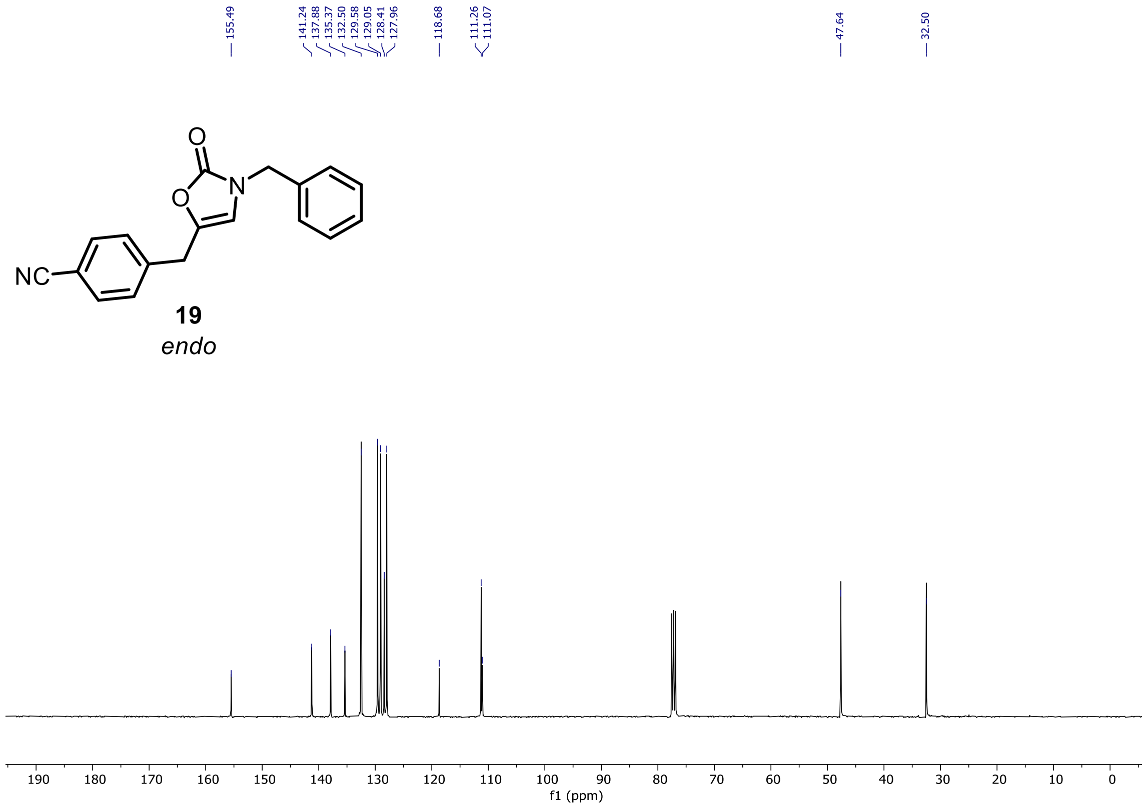

$^{13}C\{^{1}H\}$ NMR spectrum of compound **19 endo** (101 MHz, $CDCl_3$)

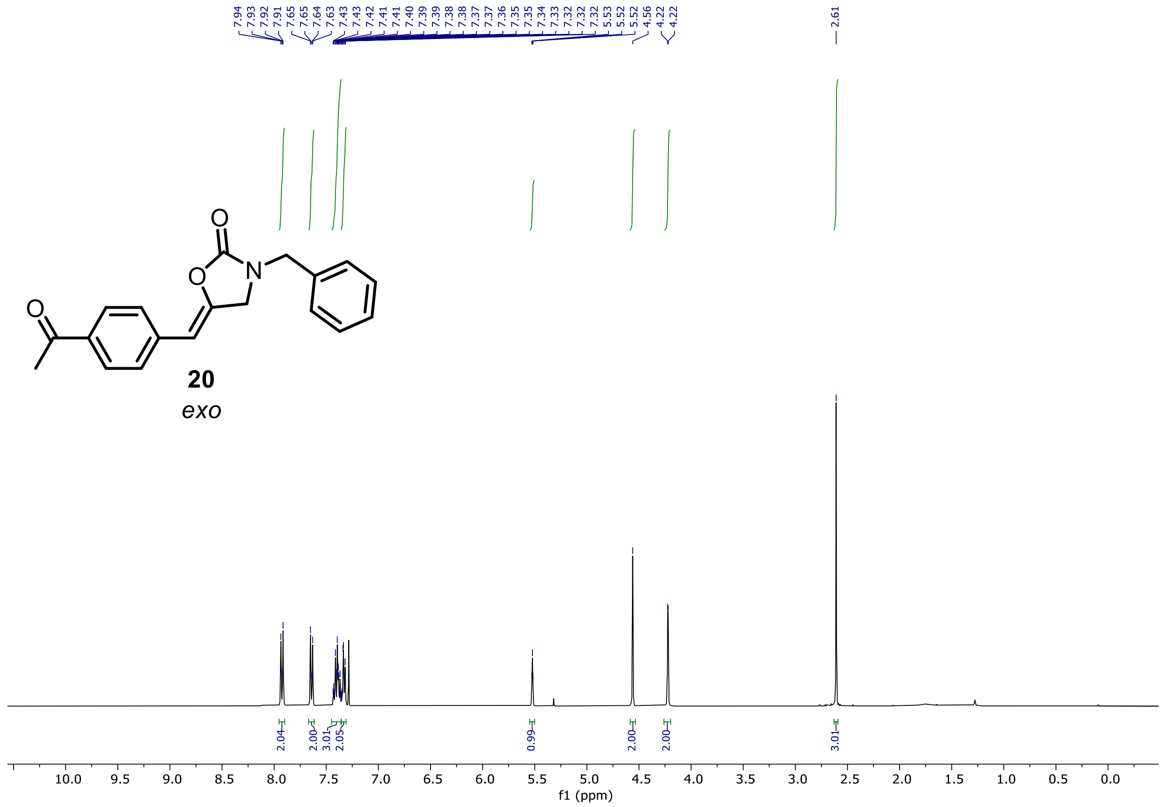

^{1}H NMR spectrum of compound **20 exo** (400 MHz, $CDCl_3$)

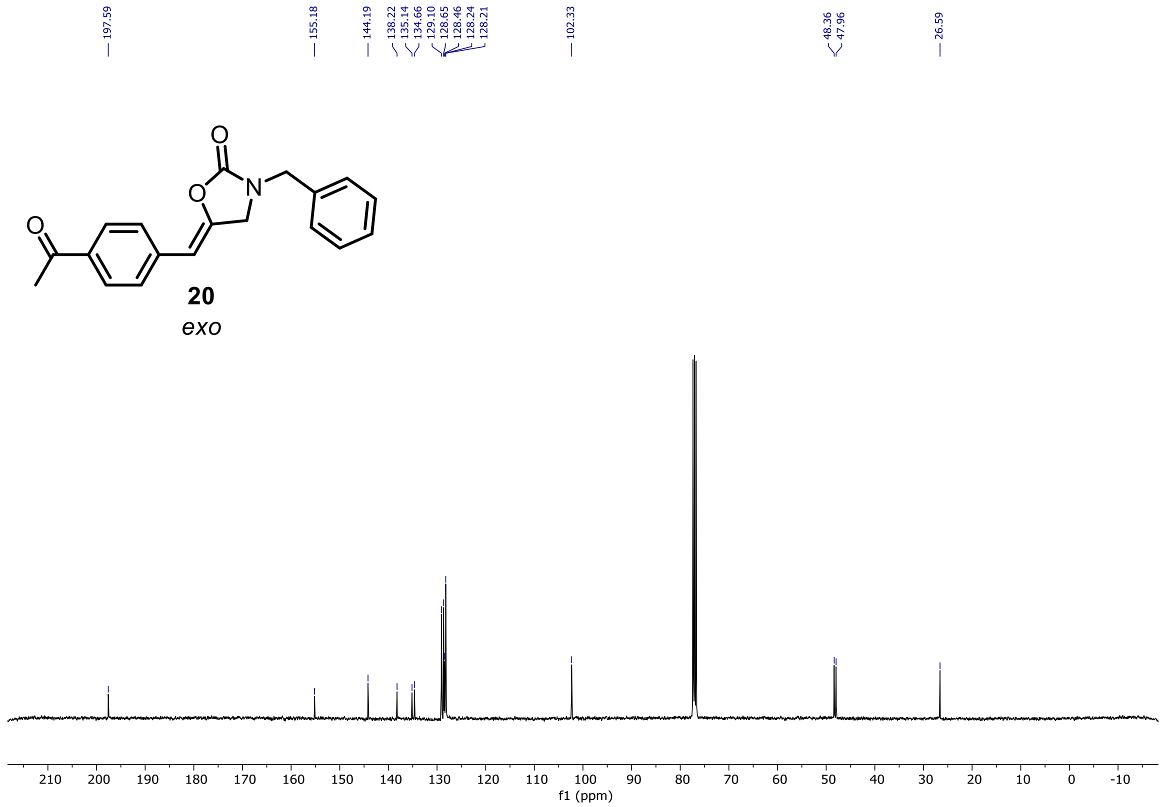

$^{13}C\{^1H\}$ NMR spectrum of compound **20 exo** (101 MHz, $CDCl_3$)

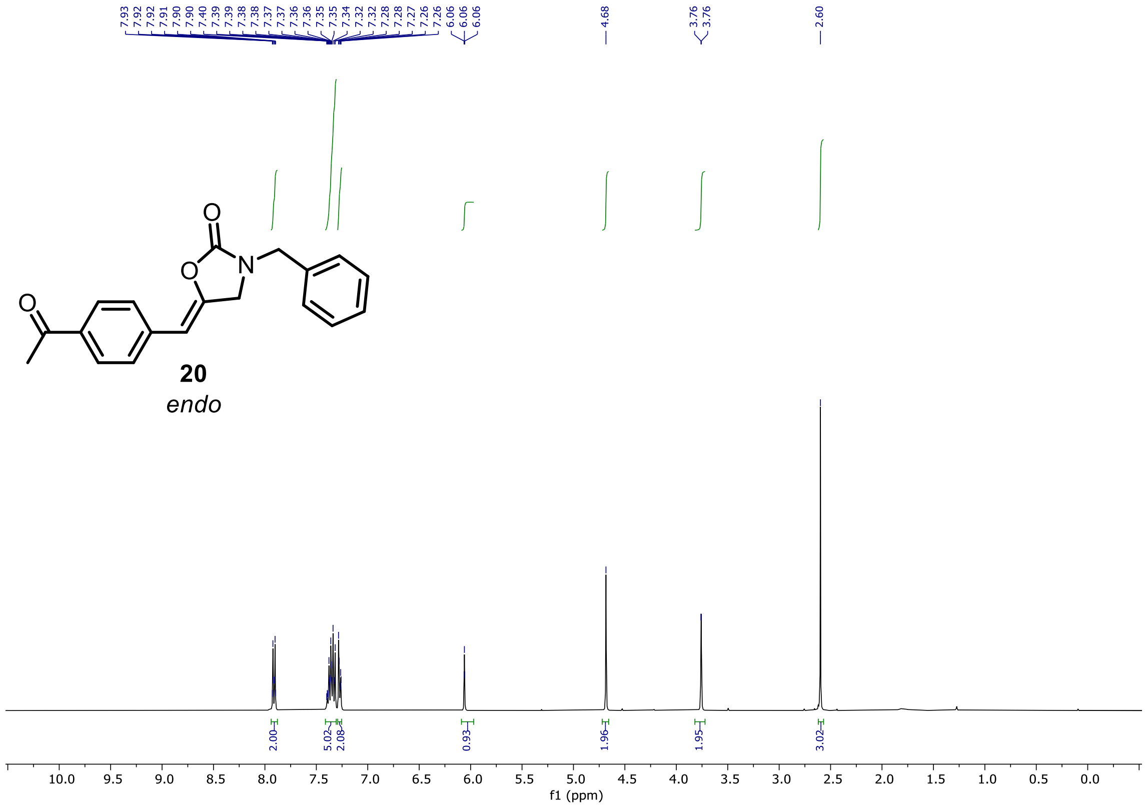

^{1}H NMR spectrum of compound **20 endo** (400 MHz, $CDCl_3$)

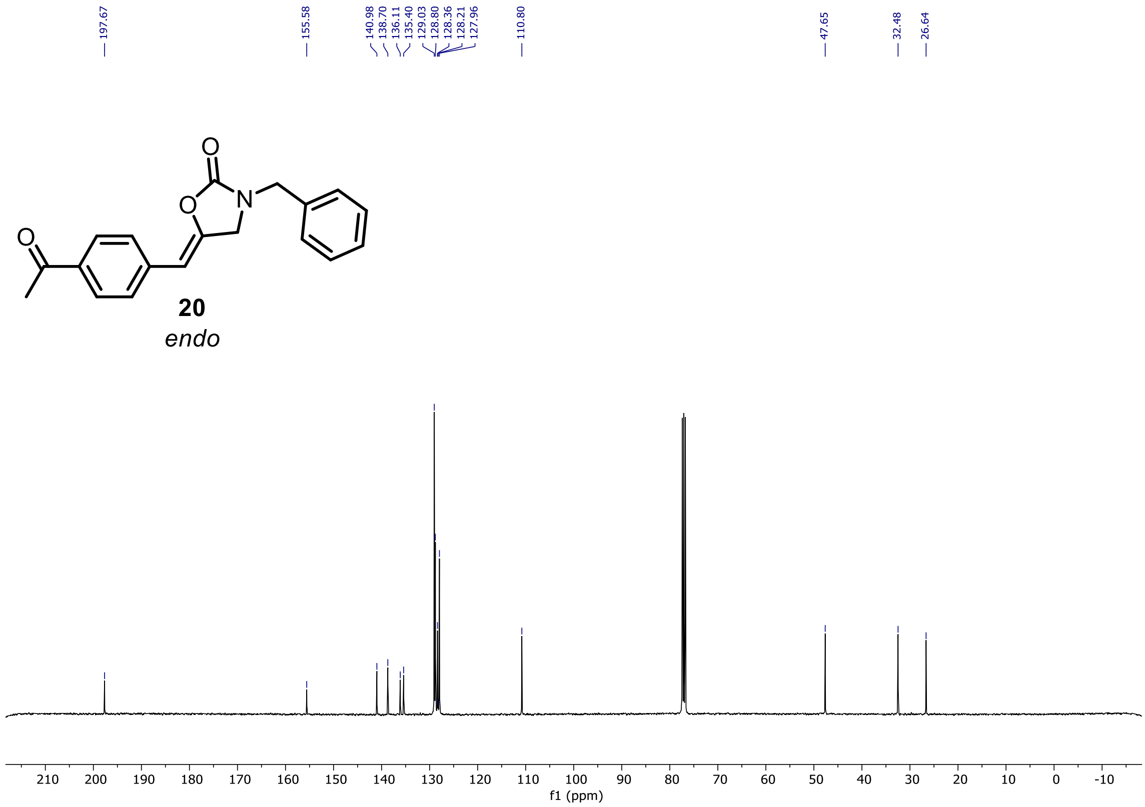

^{13}C{^{1}H} NMR spectrum of compound **20 endo** (101 MHz, $CDCl_3$)

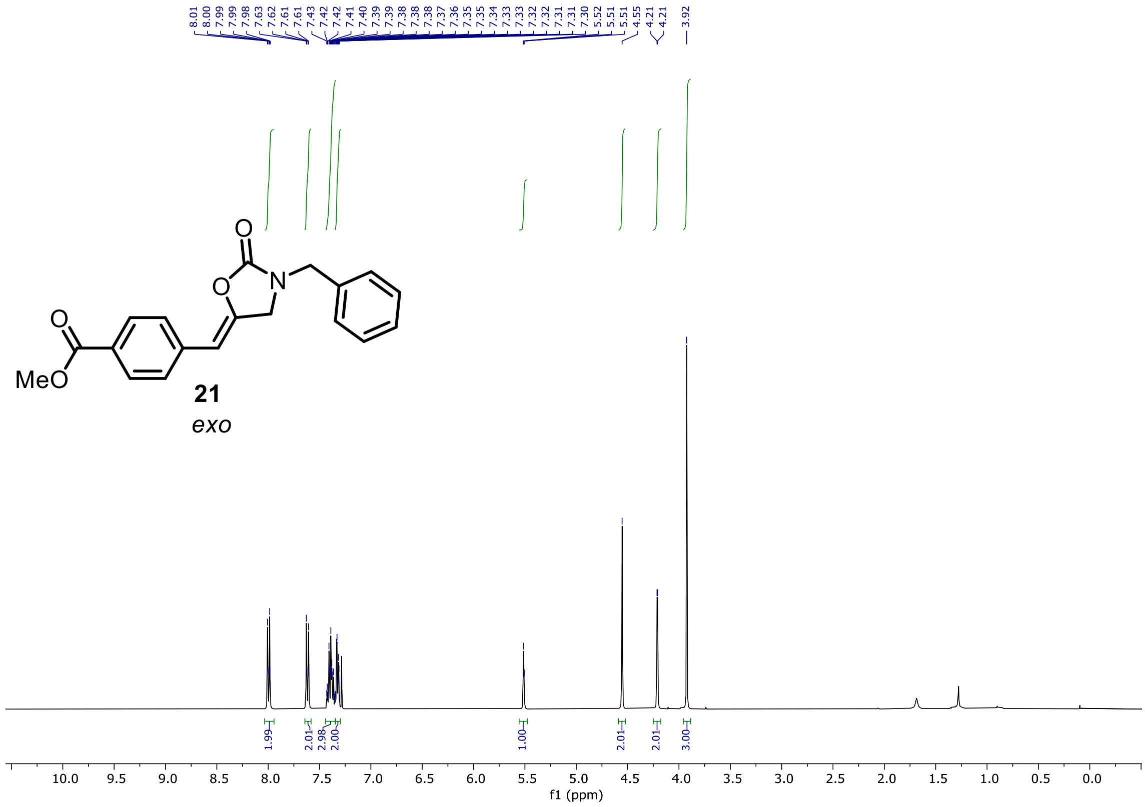

^{1}H NMR spectrum of compound **21 exo** (400 MHz, $CDCl_3$)

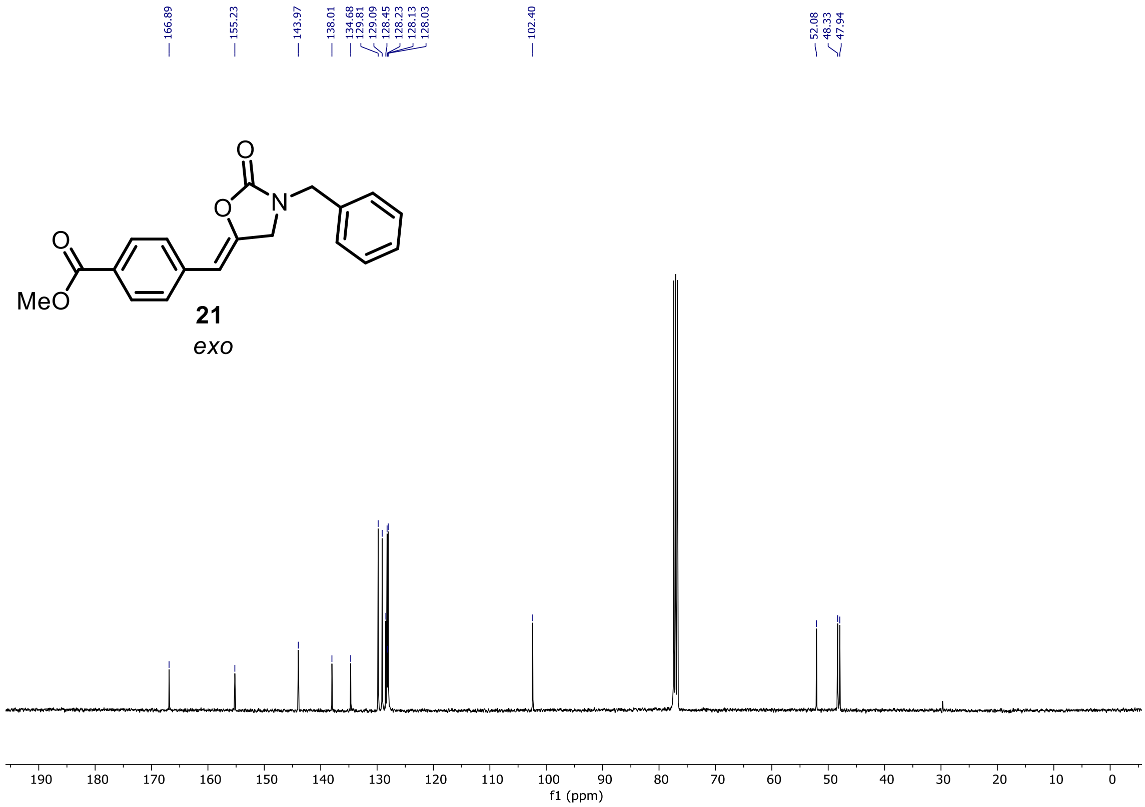

$^{13}C\{^{1}H\}$ NMR spectrum of compound **21 exo** (101 MHz, $CDCl_3$)

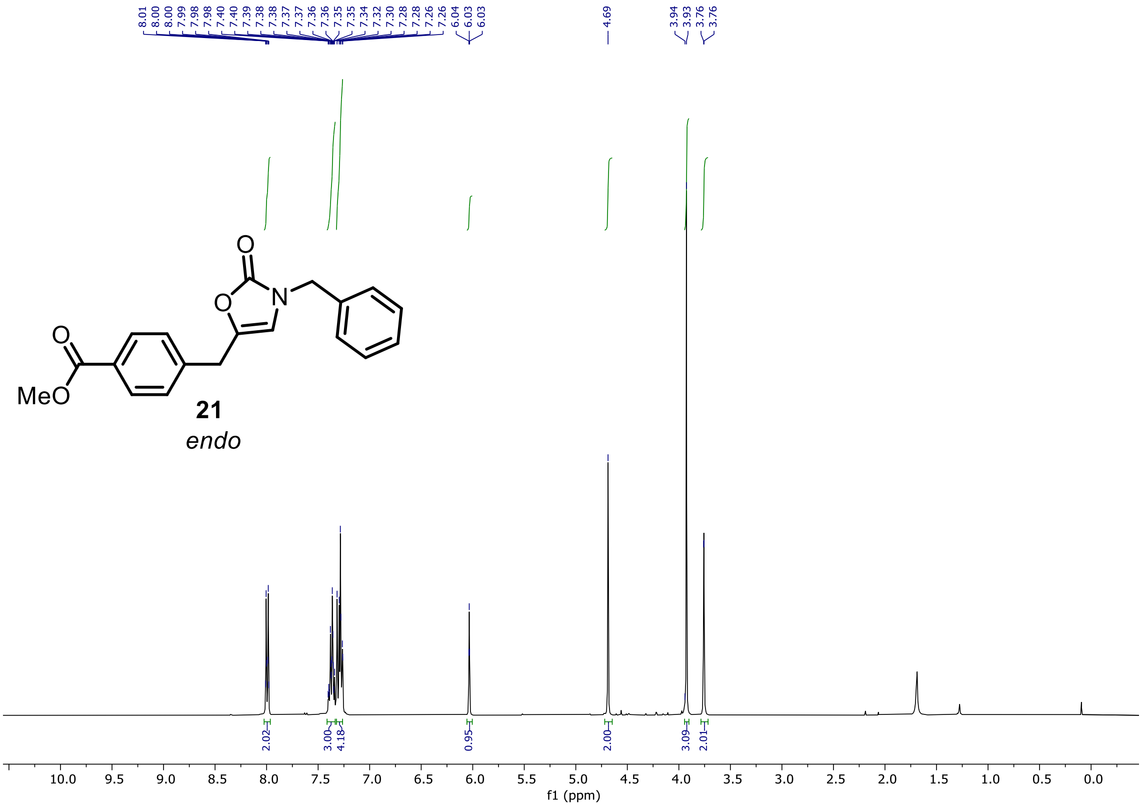

^{1}H NMR spectrum of compound **21 endo** (400 MHz, $CDCl_3$)

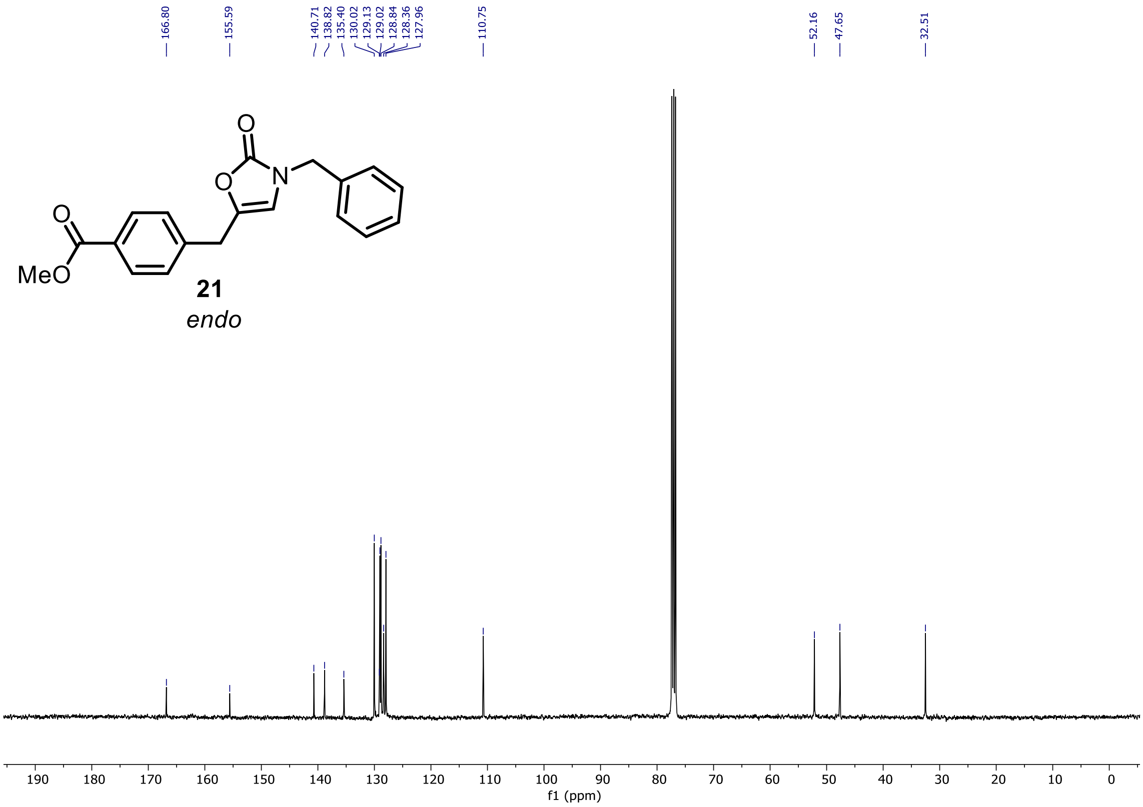

$^{13}C\{^1H\}$ NMR spectrum of compound **21 endo** (101 MHz, $CDCl_3$)

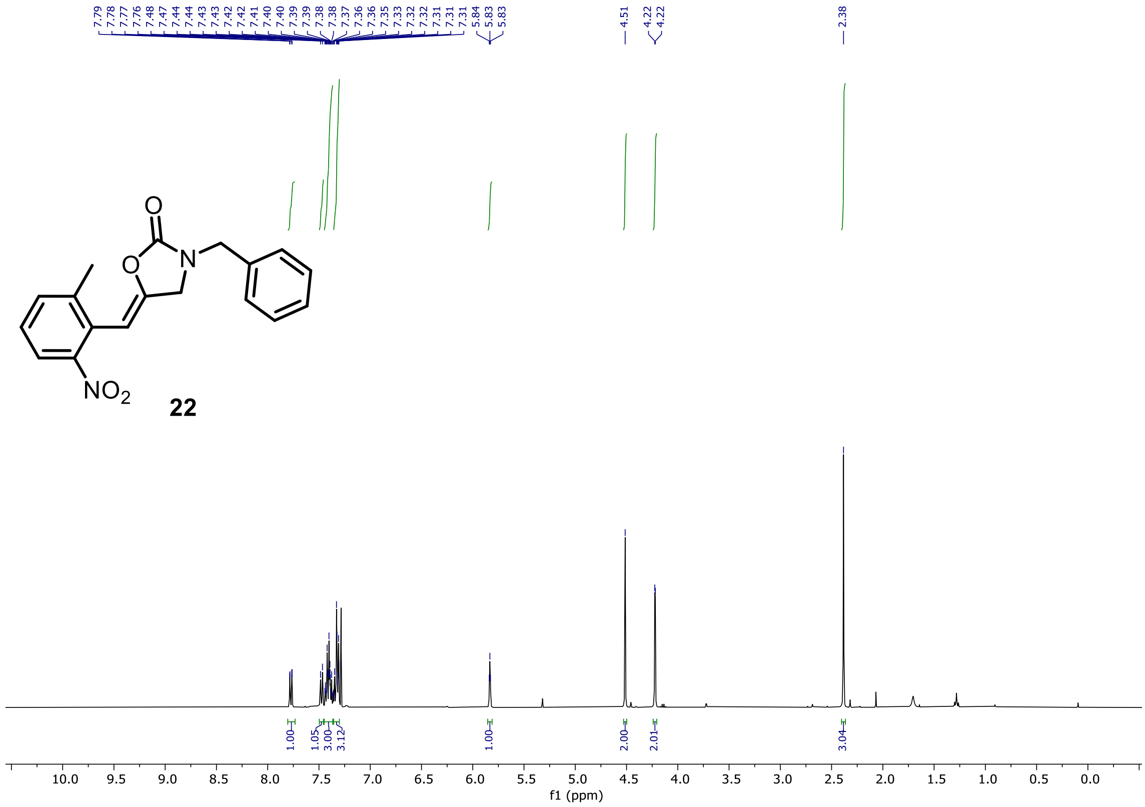

^{1}H NMR spectrum of compound **22** (400 MHz, $CDCl_3$)

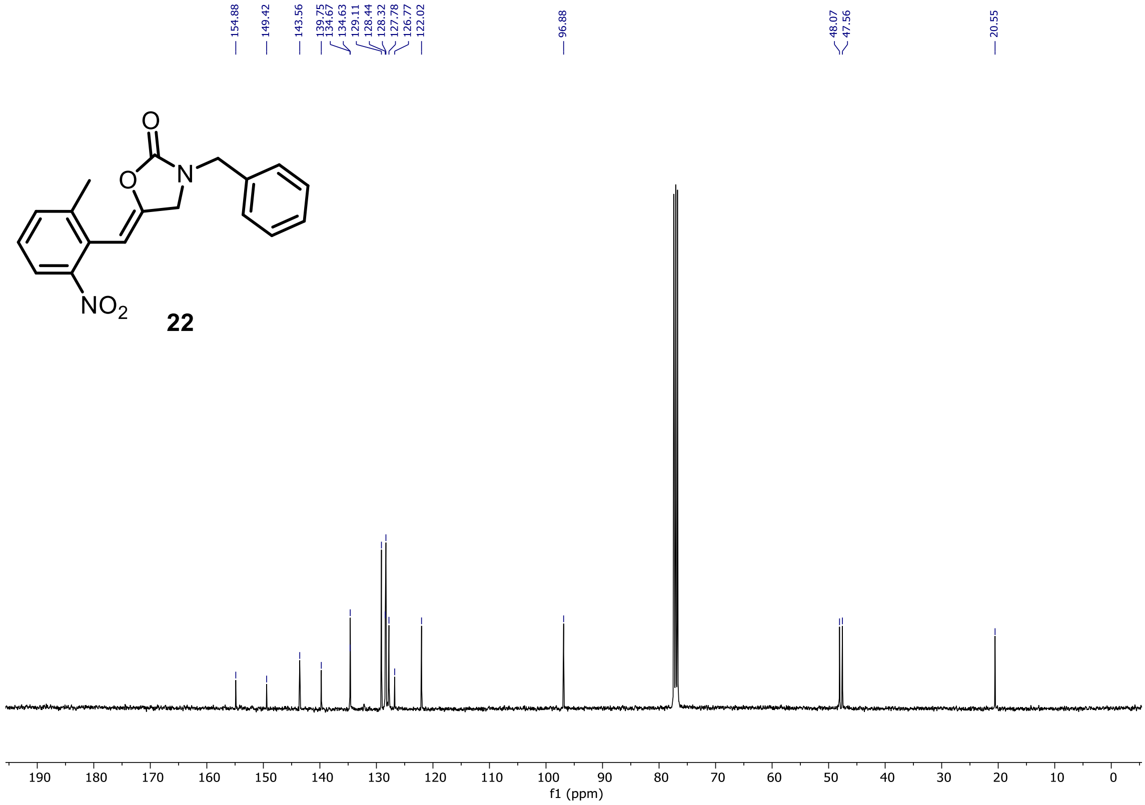

$^{13}C\{^{1}H\}$ NMR spectrum of compound **22** (101 MHz, $CDCl_3$)

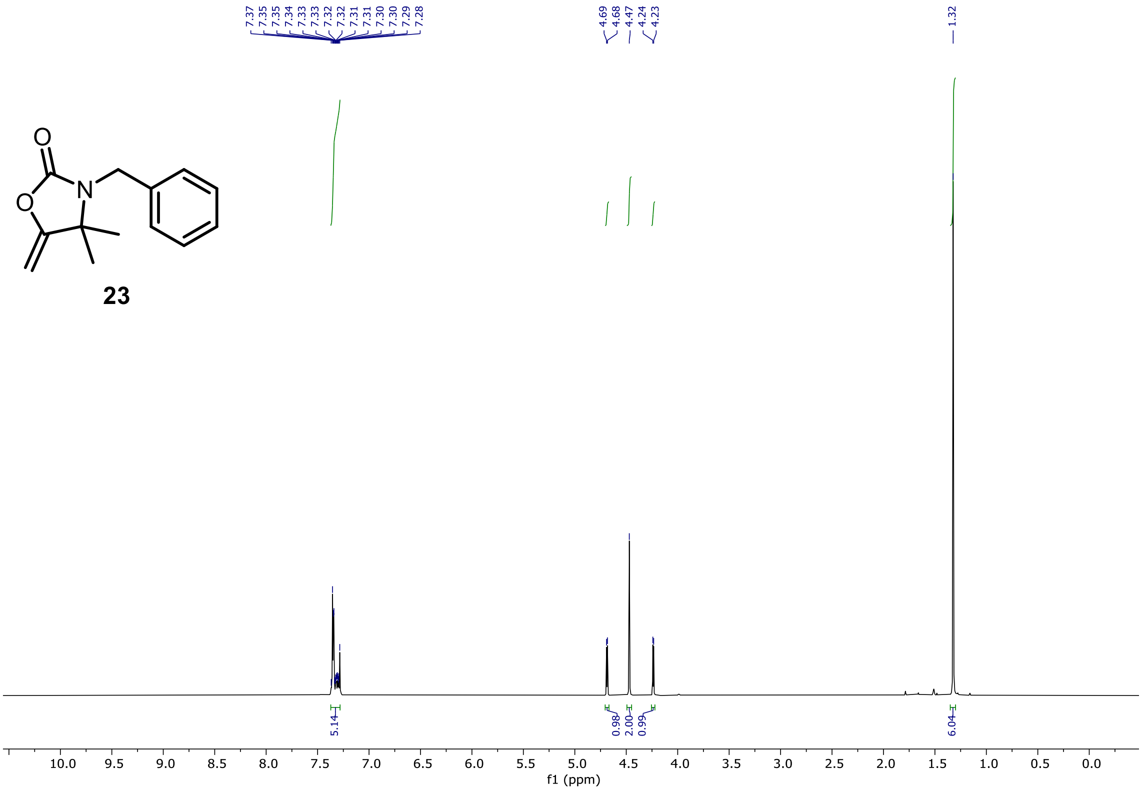

^{1}H NMR spectrum of compound **23** (400 MHz, $CDCl_3$)

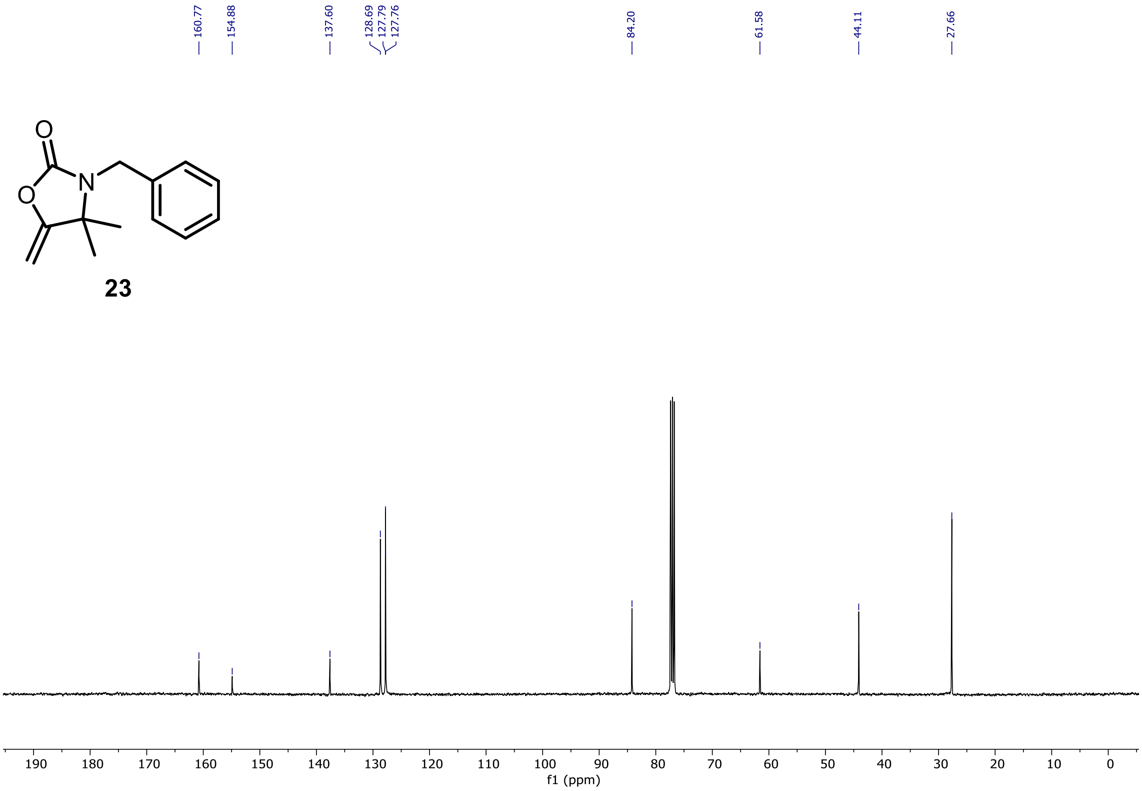

$^{13}C\{^{1}H\}$ NMR spectrum of compound **23** (101 MHz, $CDCl_3$)

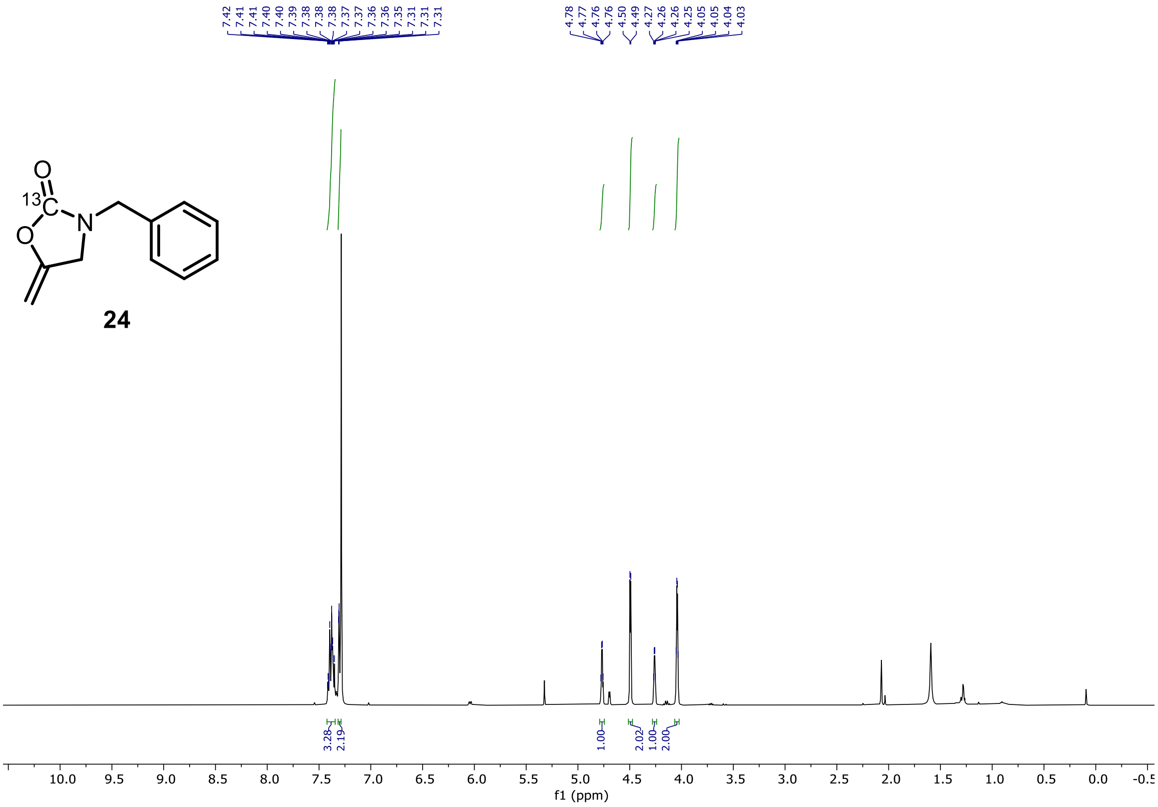

^{1}H NMR spectrum of compound **24** (400 MHz, $CDCl_3$)

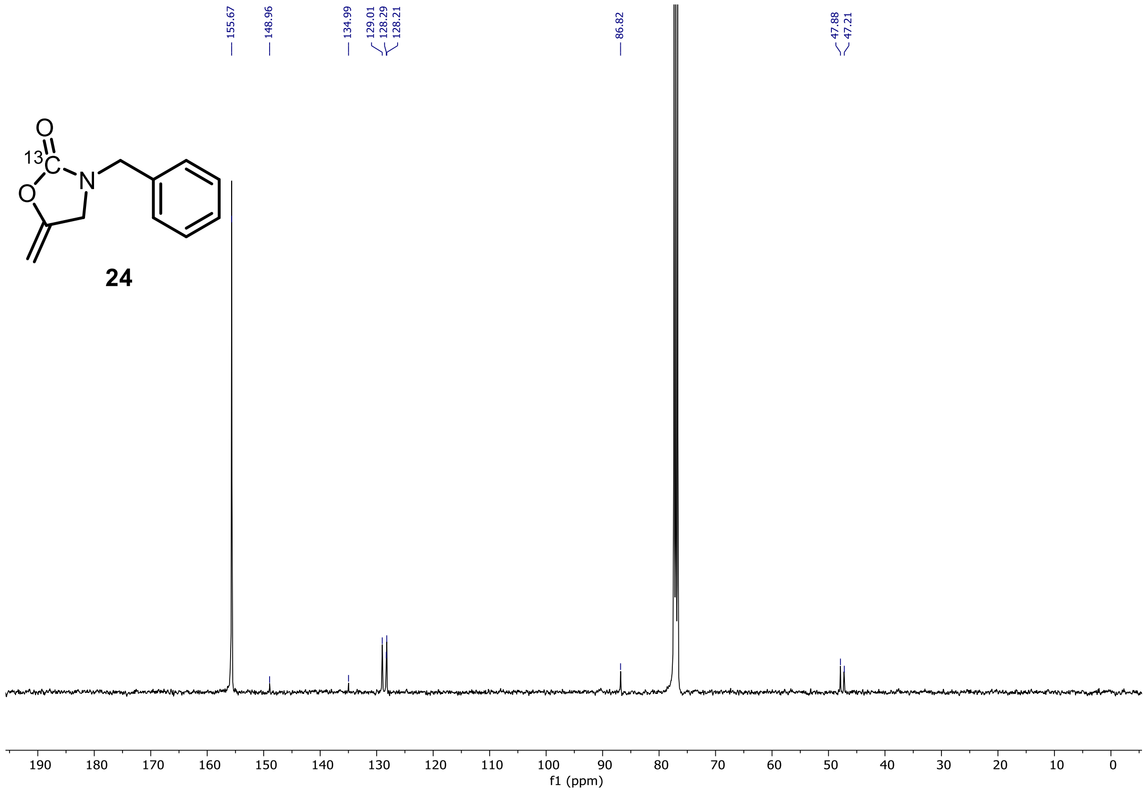

^{13}C{^{1}H} NMR spectrum of compound **24** (101 MHz, $CDCl_3$)

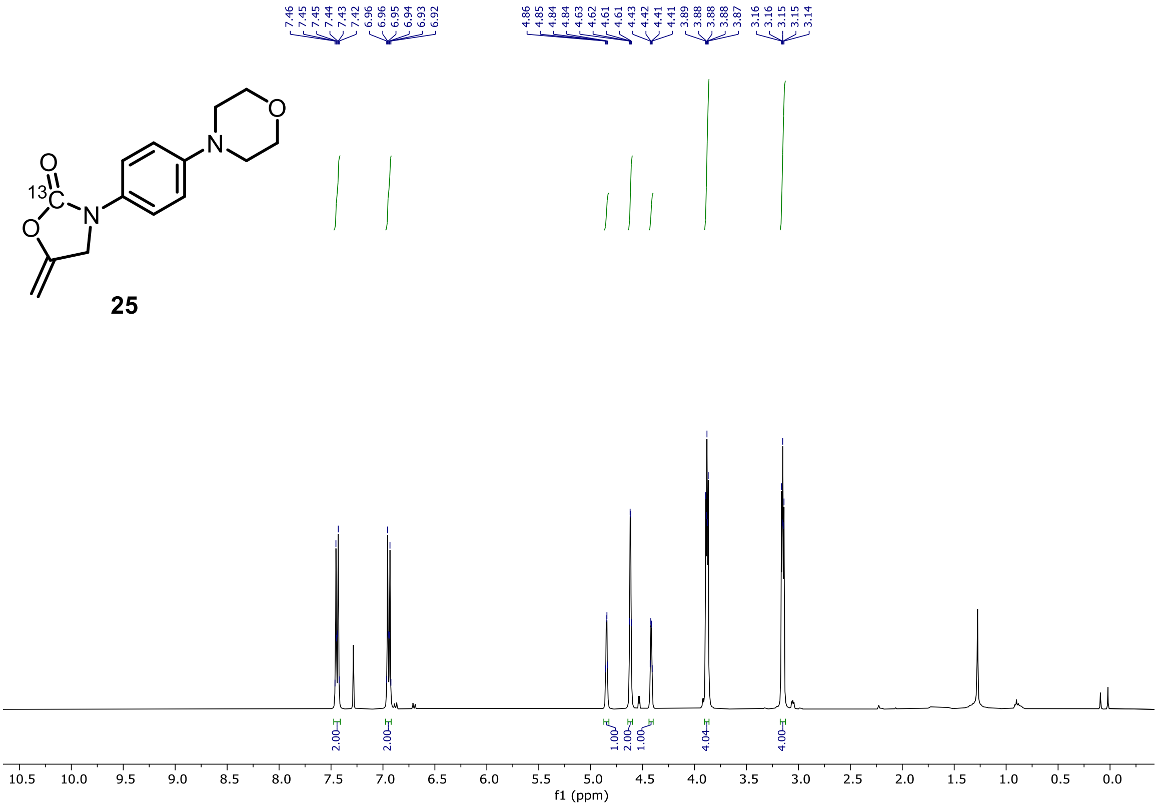

^{1}H NMR spectrum of compound **25** (400 MHz, $CDCl_3$)

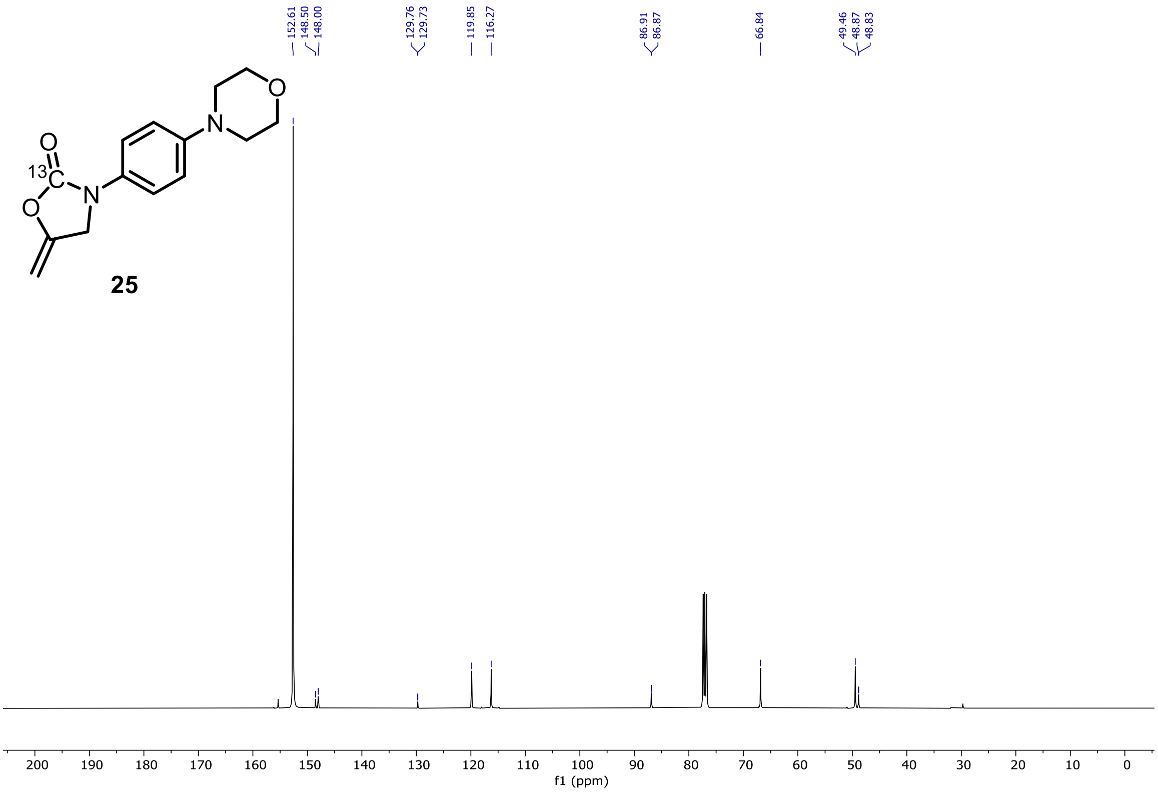

^{13}C{^{1}H} NMR spectrum of compound **25** (101 MHz, $CDCl_3$)

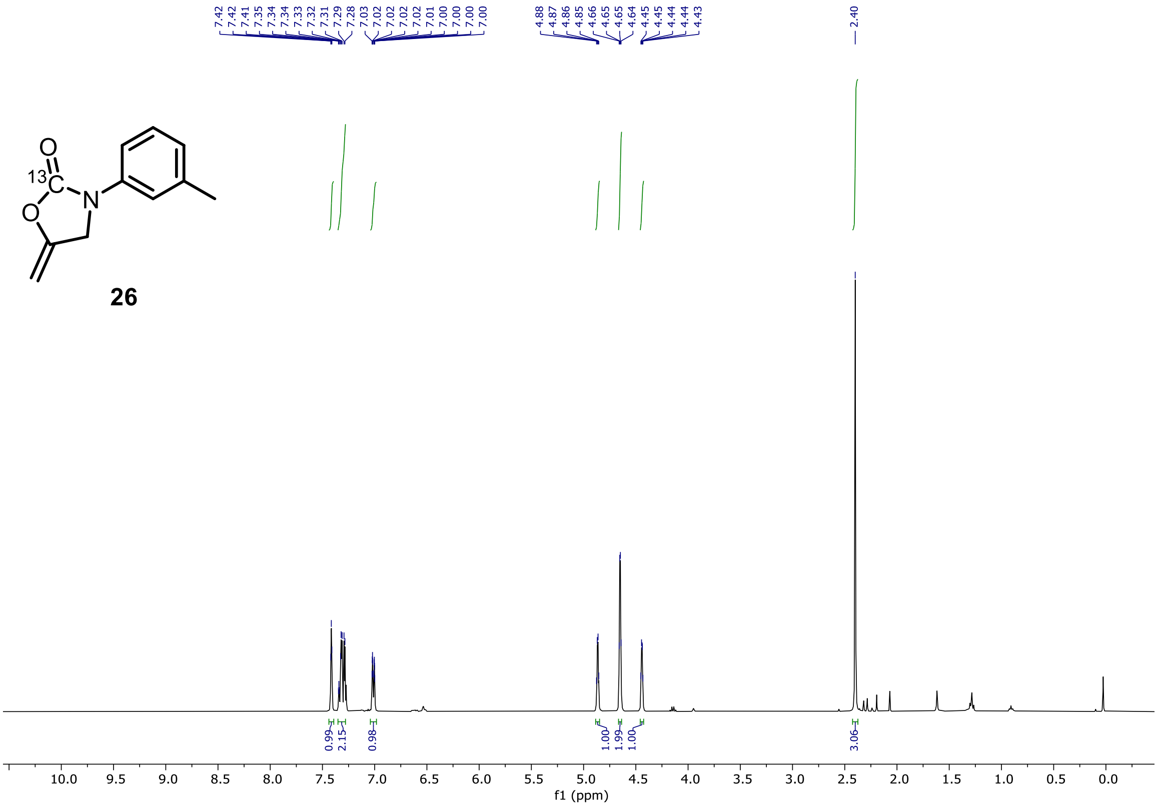

^{1}H NMR spectrum of compound **26** (400 MHz, $CDCl_3$)

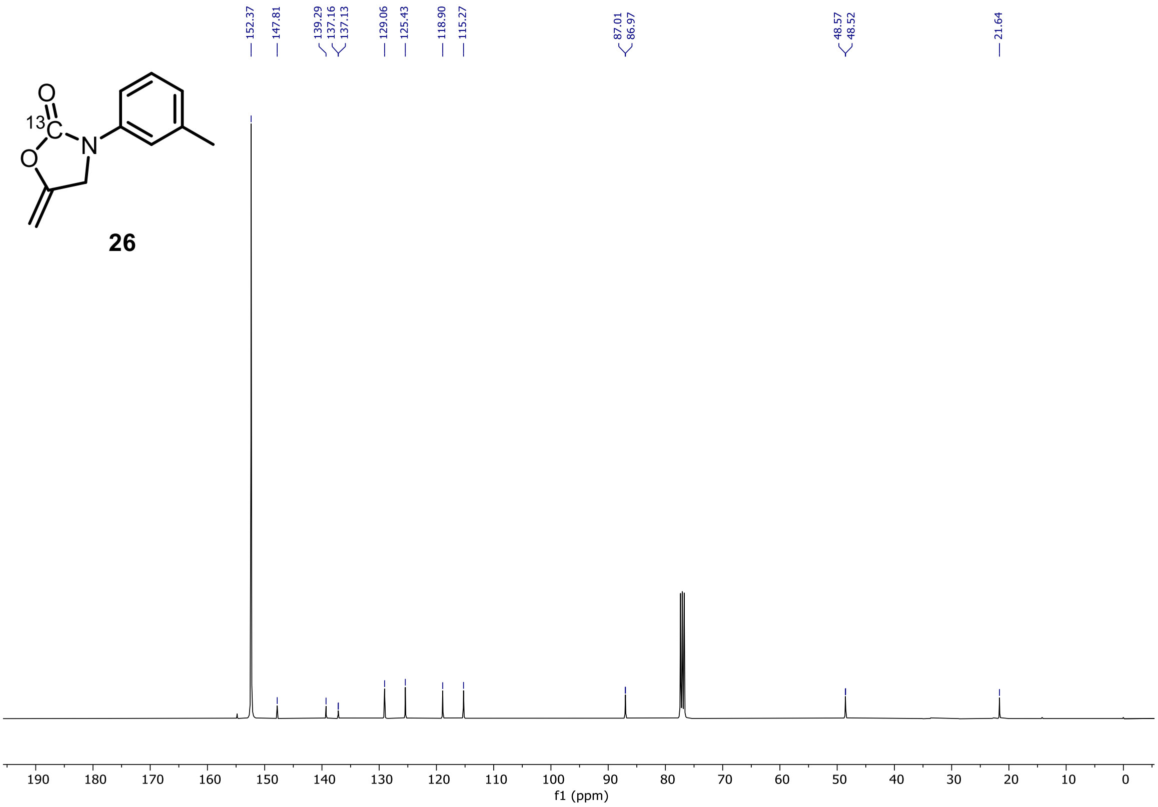

^{13}C{^{1}H} NMR spectrum of compound **26** (101 MHz, $CDCl_3$)

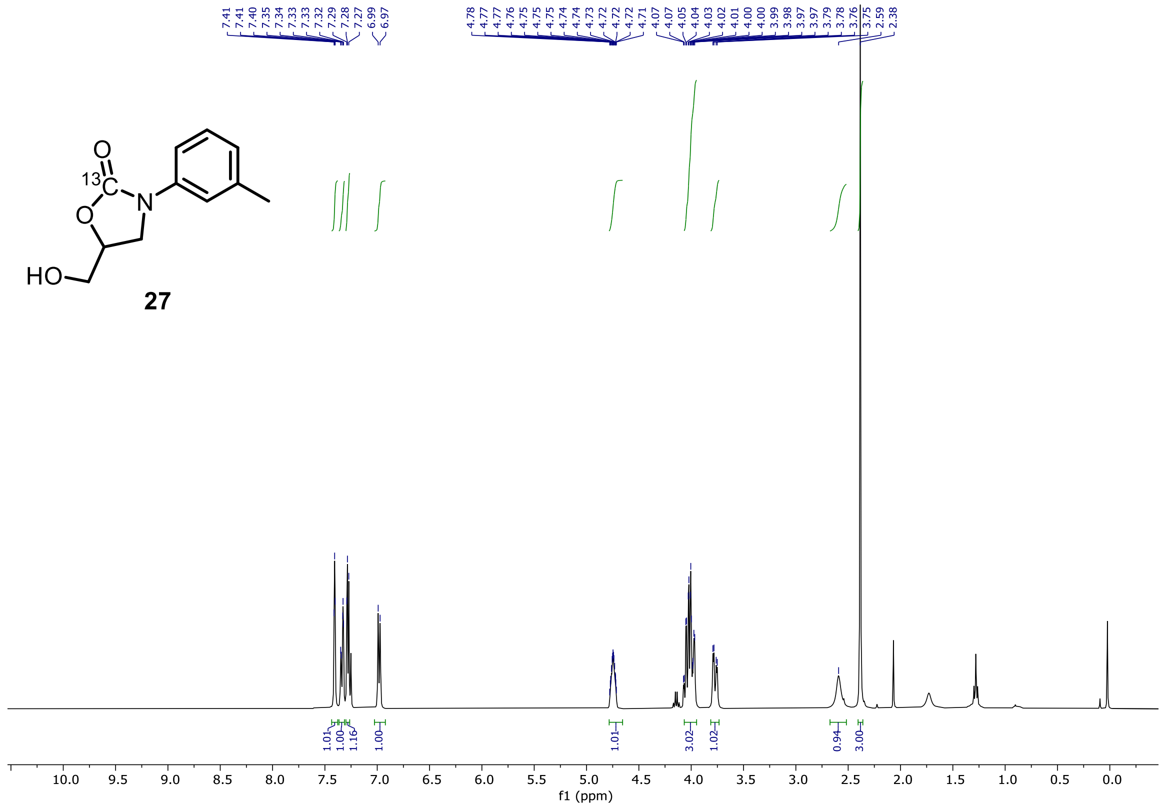

^{1}H NMR spectrum of compound **27** (400 MHz, $CDCl_3$)

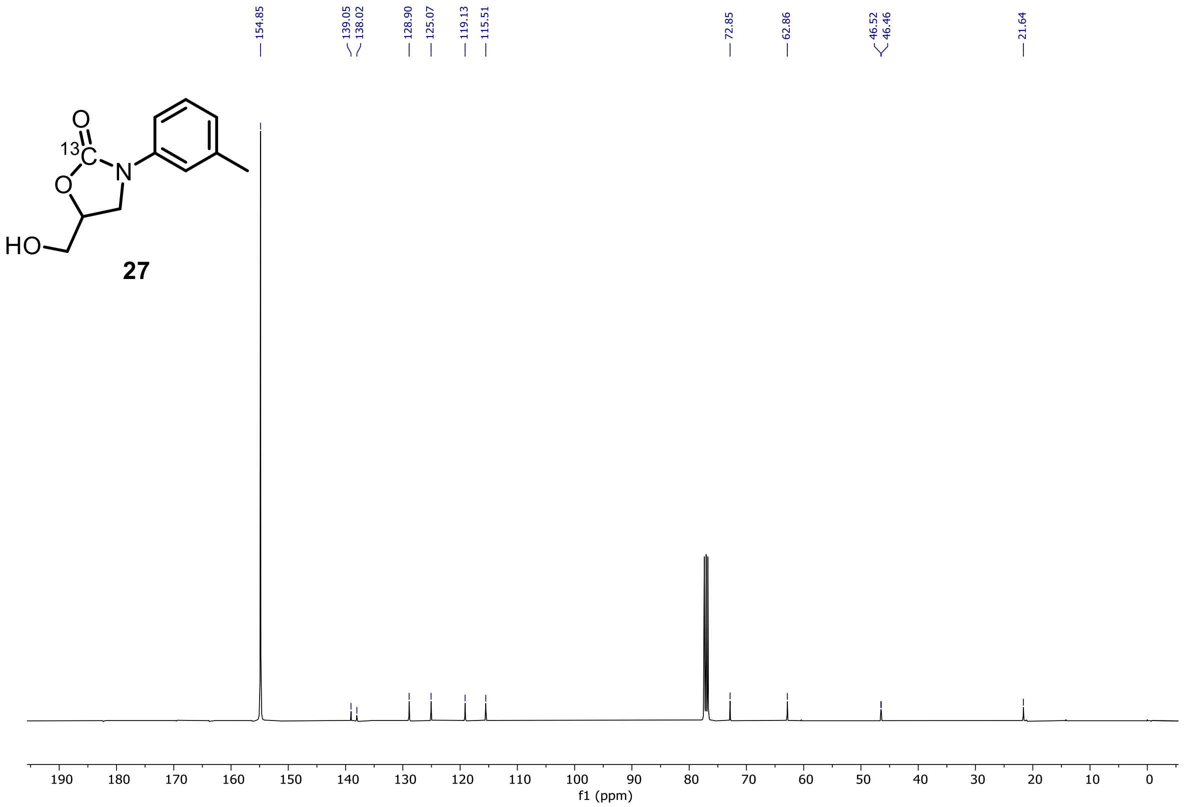

$^{13}C\{^{1}H\}$ NMR spectrum of compound **27** (101 MHz, $CDCl_3$)

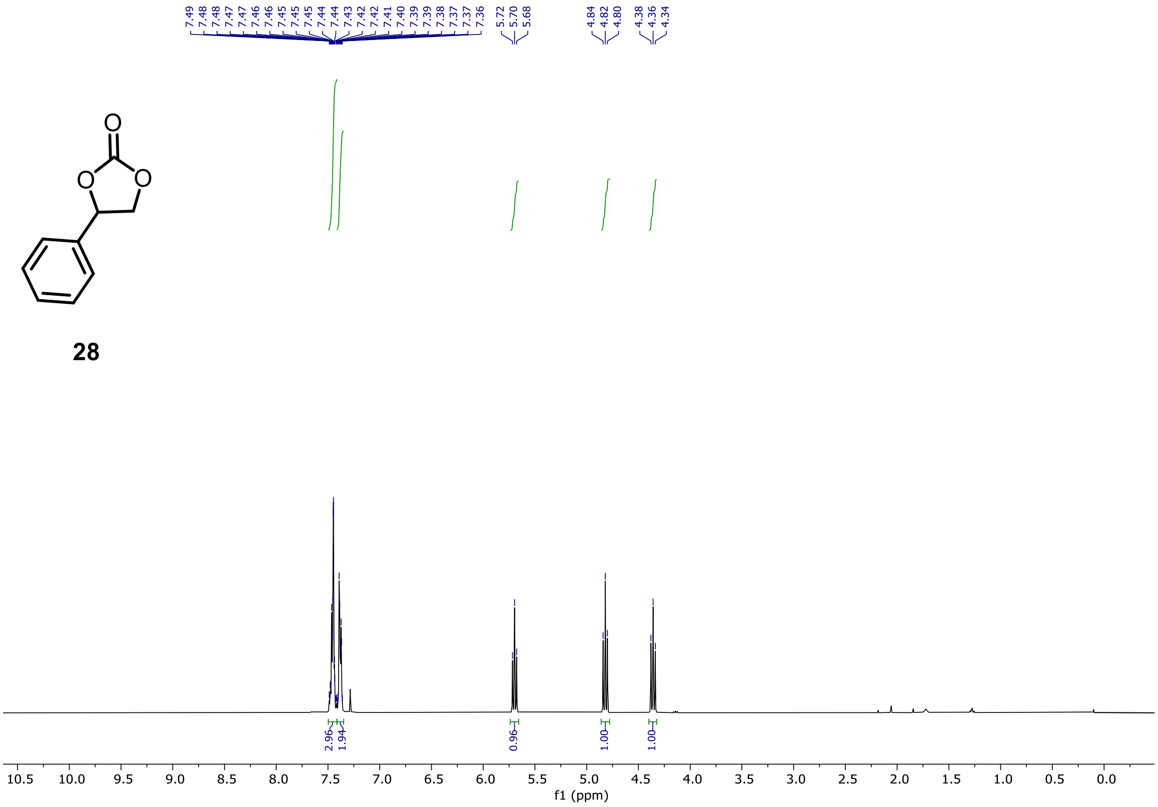

^{1}H NMR spectrum of compound **28** (400 MHz, $CDCl_3$)

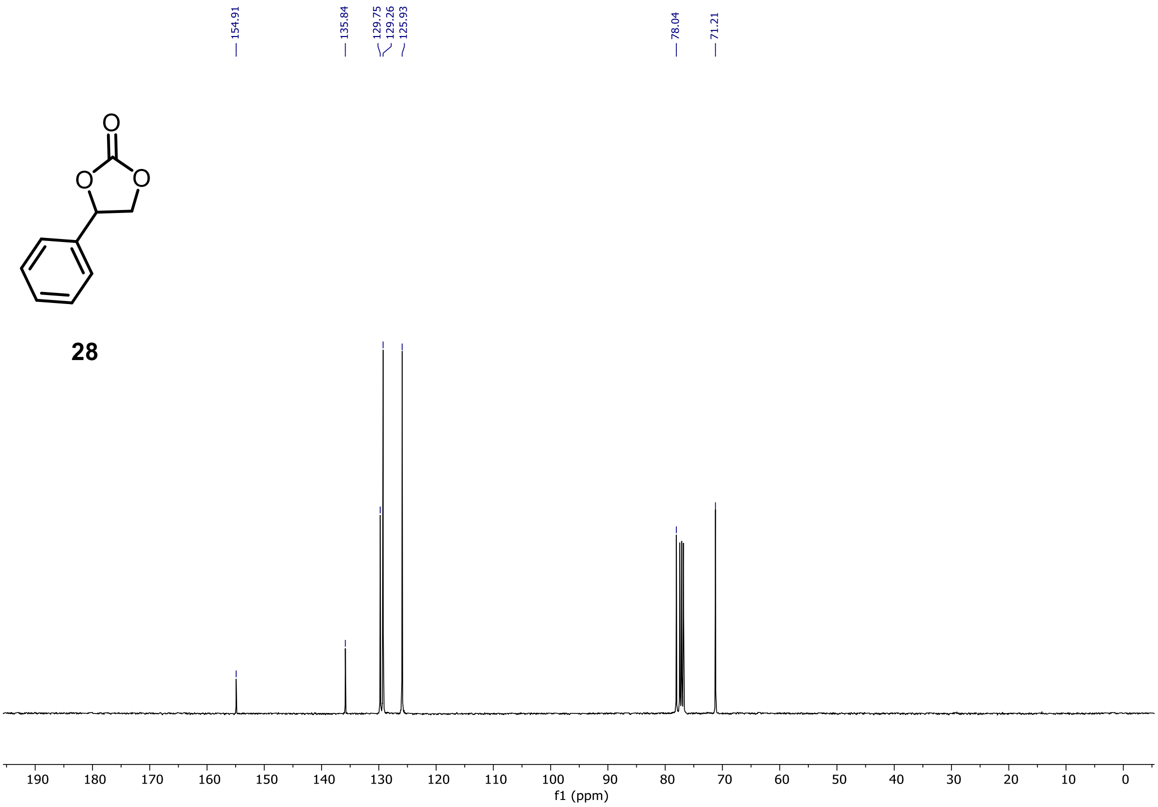

^{13}C{^{1}H} NMR spectrum of compound **28** (101 MHz, $CDCl_3$)

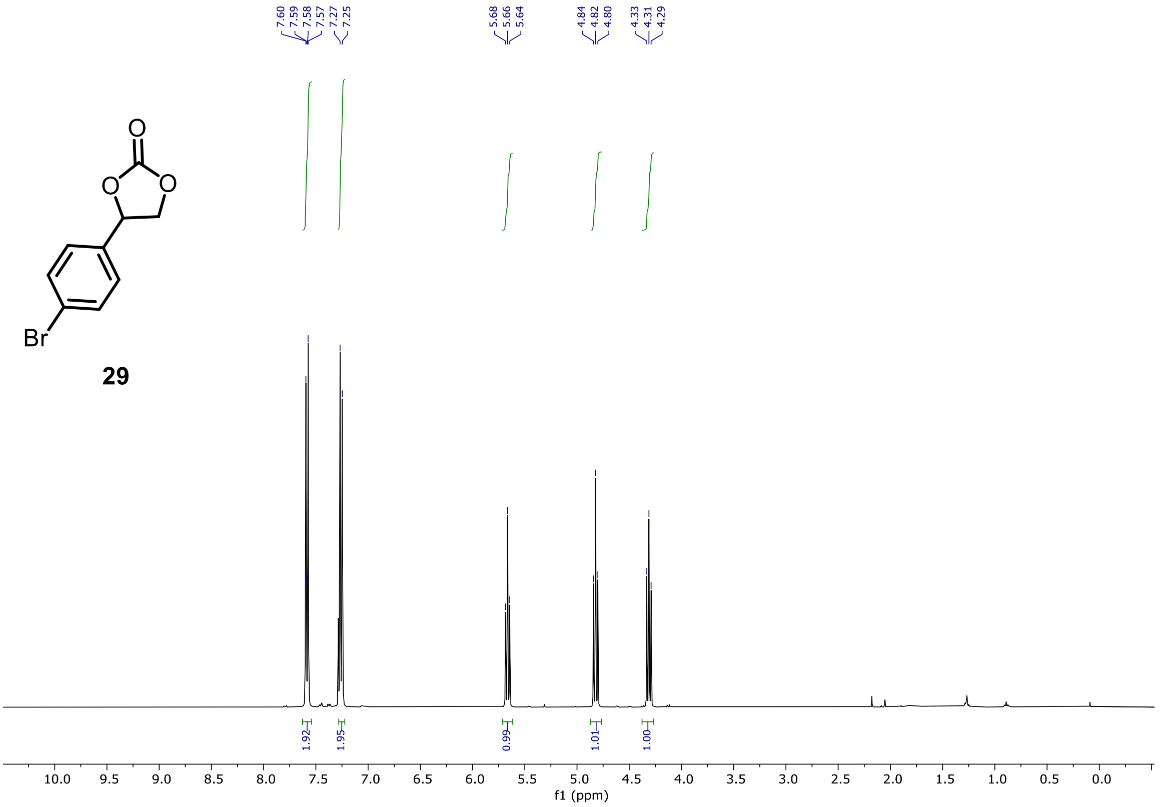

^{1}H NMR spectrum of compound **29** (400 MHz, $CDCl_3$)

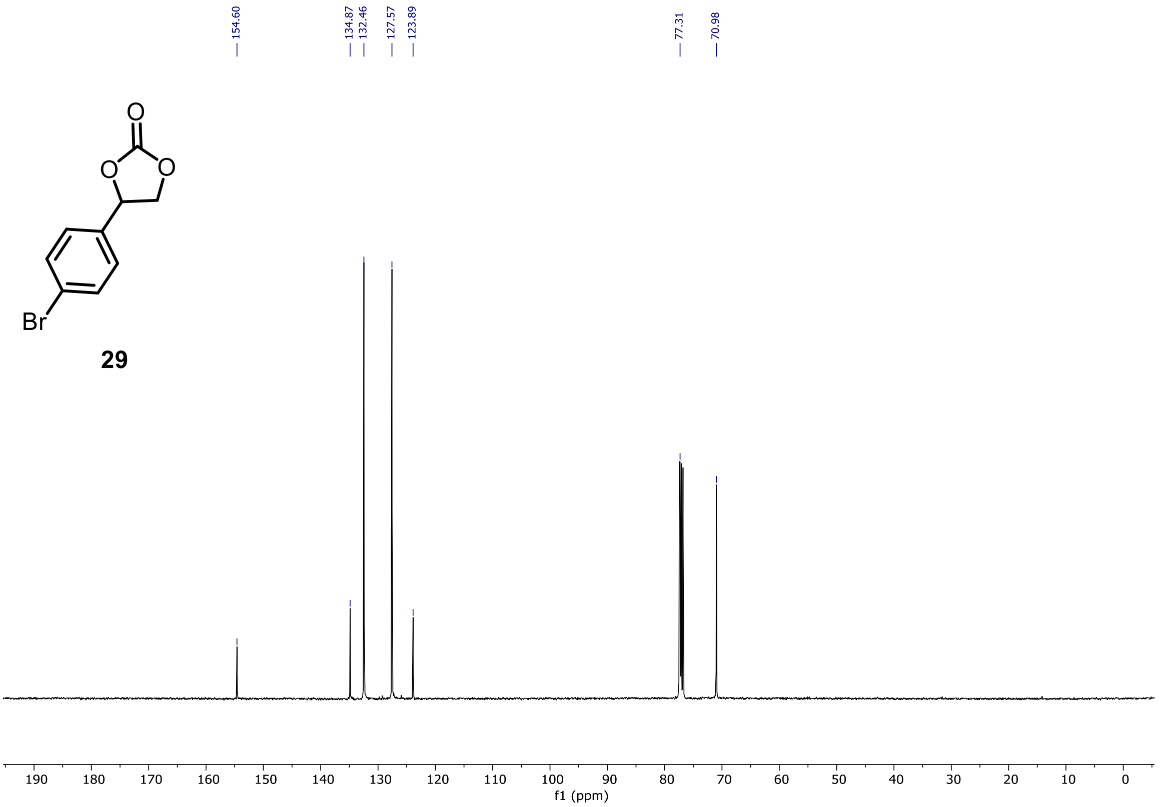

$^{13}C\{^{1}H\}$ NMR spectrum of compound **29** (101 MHz, $CDCl_3$)

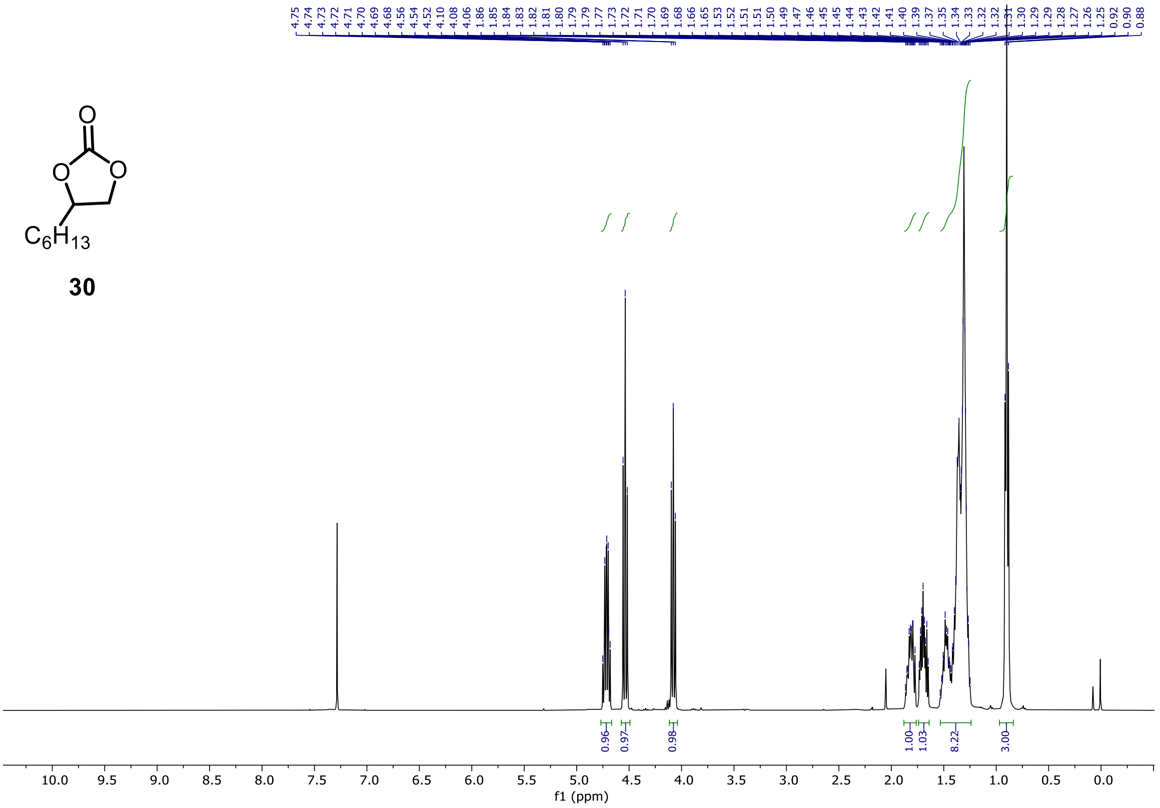

^{1}H NMR spectrum of compound **30** (400 MHz, $CDCl_3$)

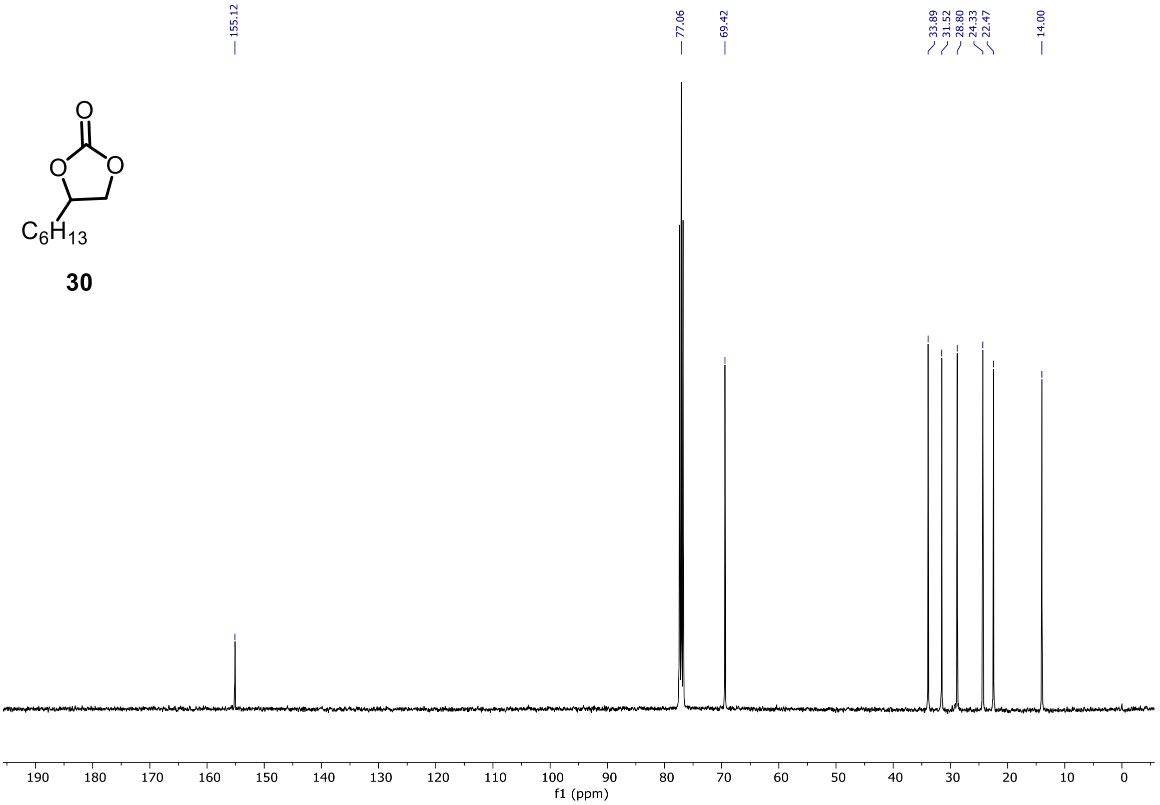

$^{13}C\{^{1}H\}$ NMR spectrum of compound **30** (101 MHz, $CDCl_3$)

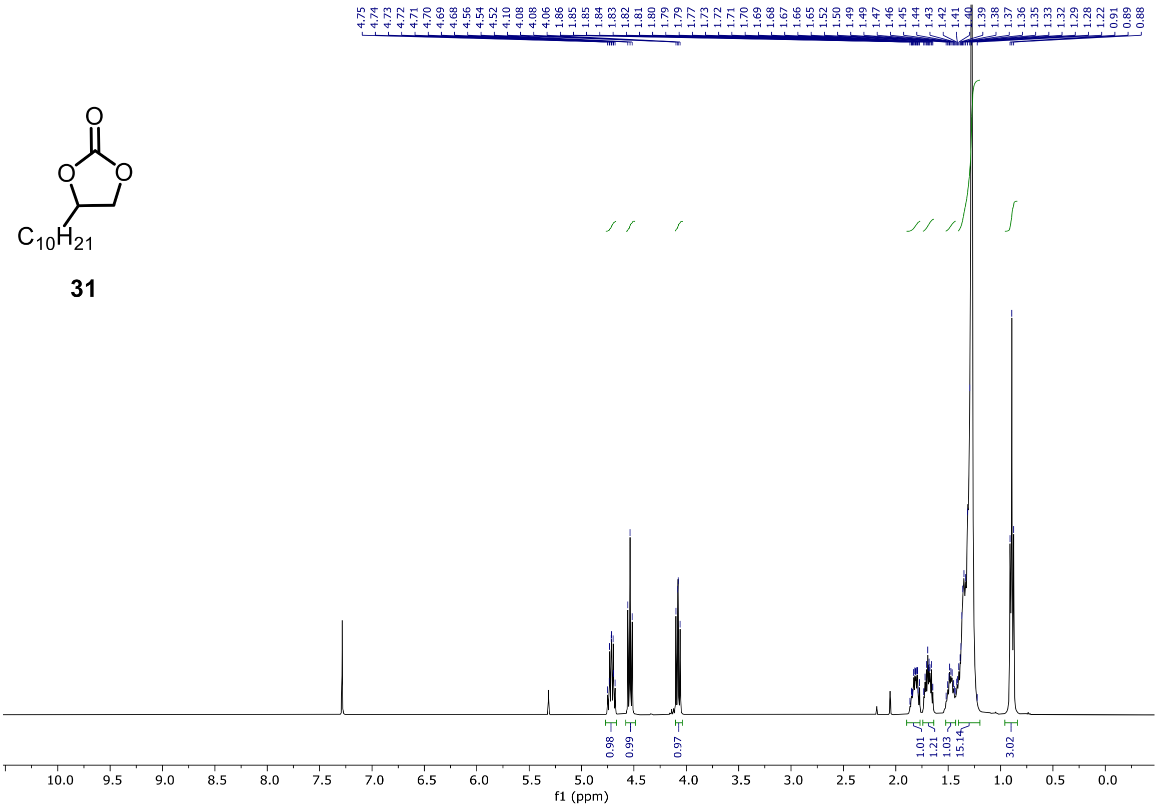

^{1}H NMR spectrum of compound **31** (400 MHz, $CDCl_3$)

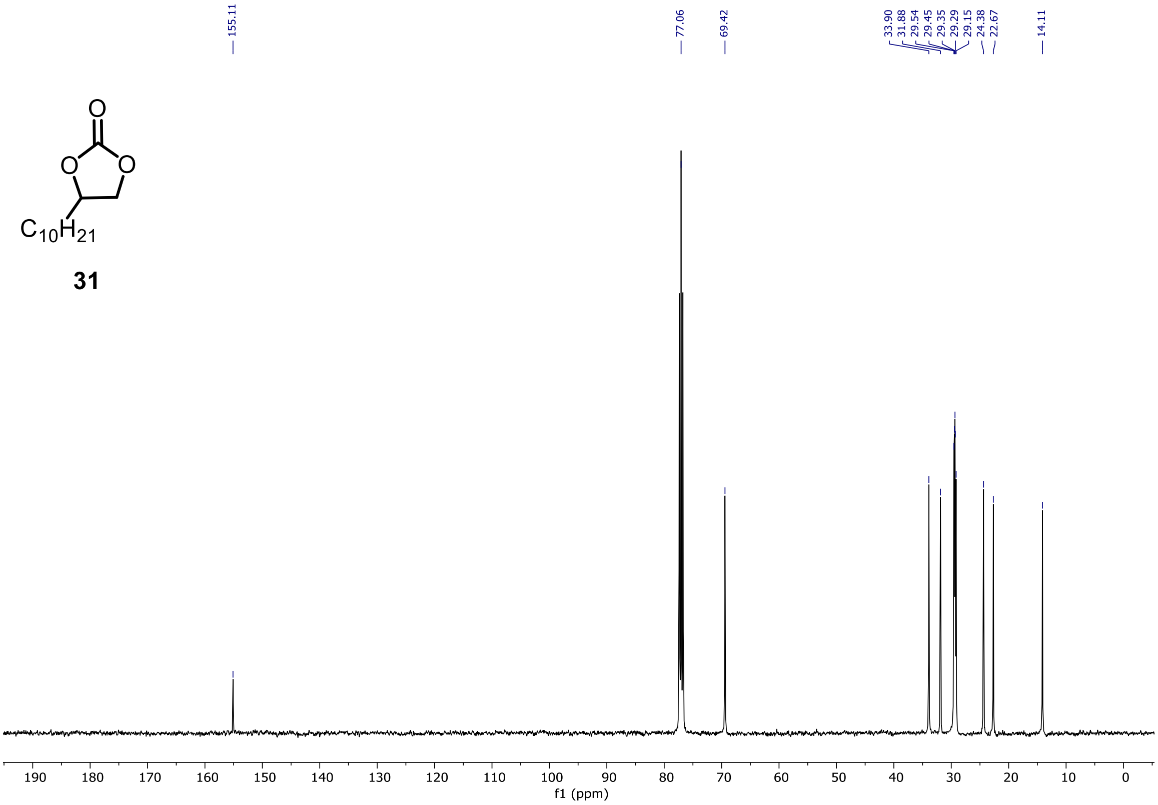

$^{13}C\{^1H\}$ NMR spectrum of compound **31** (101 MHz, $CDCl_3$)

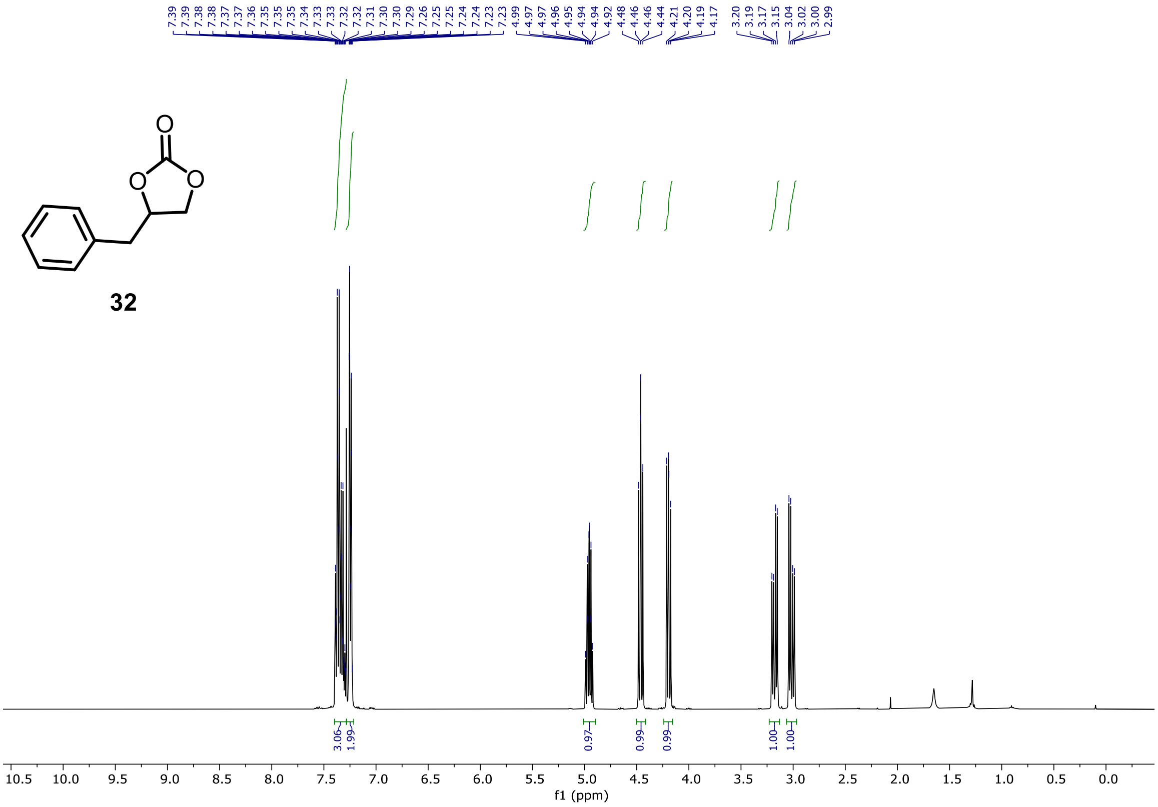

^{1}H NMR spectrum of compound **32** (400 MHz, $CDCl_3$)

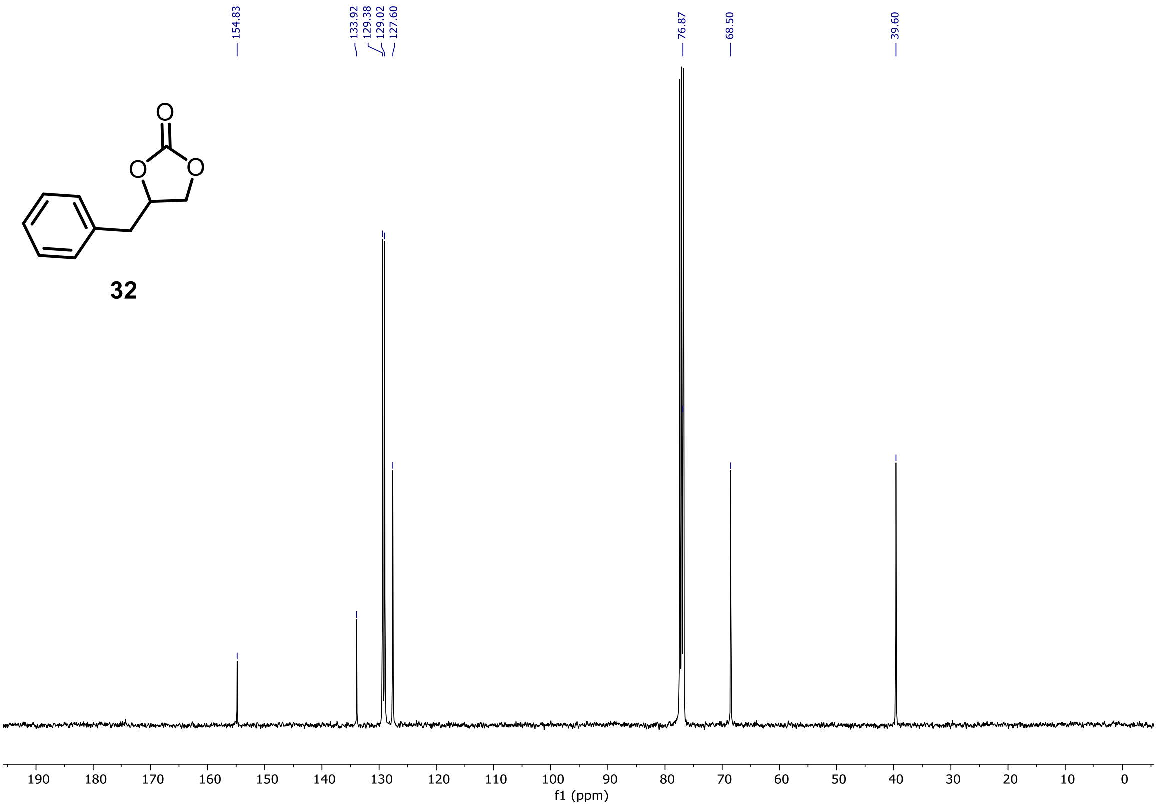

^{13}C{^{1}H} NMR spectrum of compound **32** (101 MHz, $CDCl_3$)

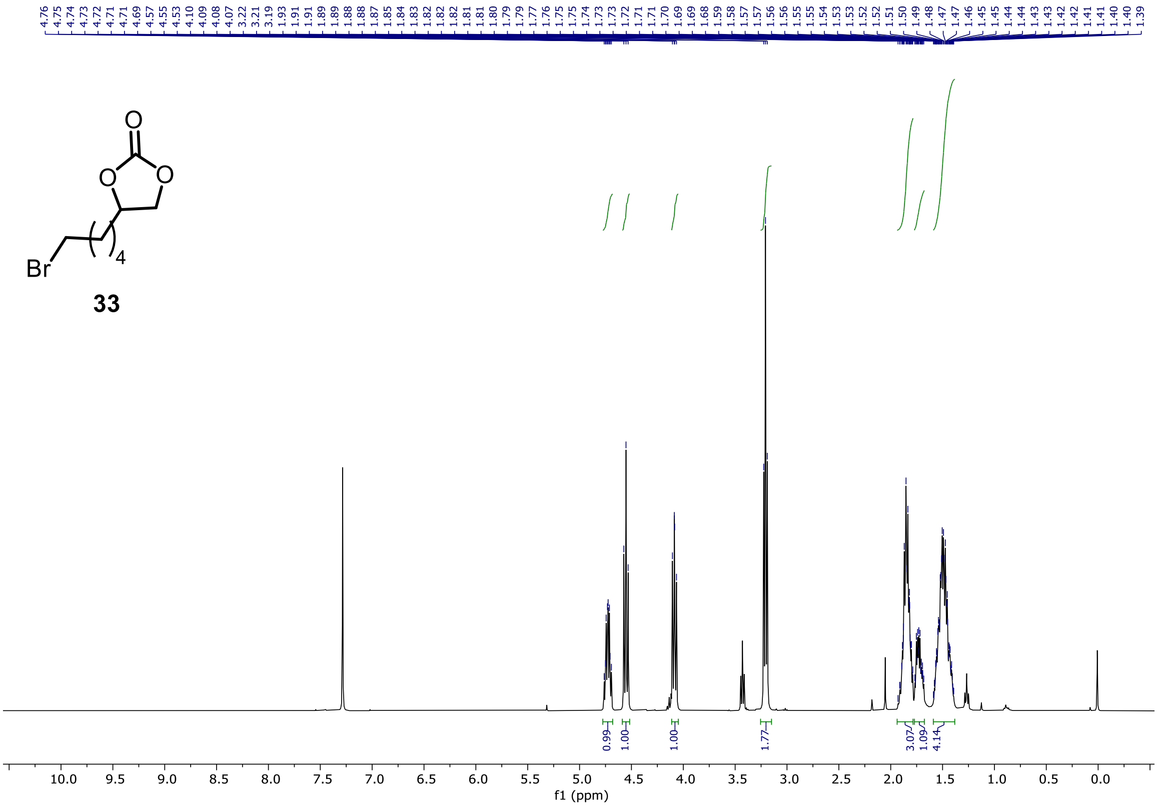

^{1}H NMR spectrum of compound **33** (400 MHz, $CDCl_3$)

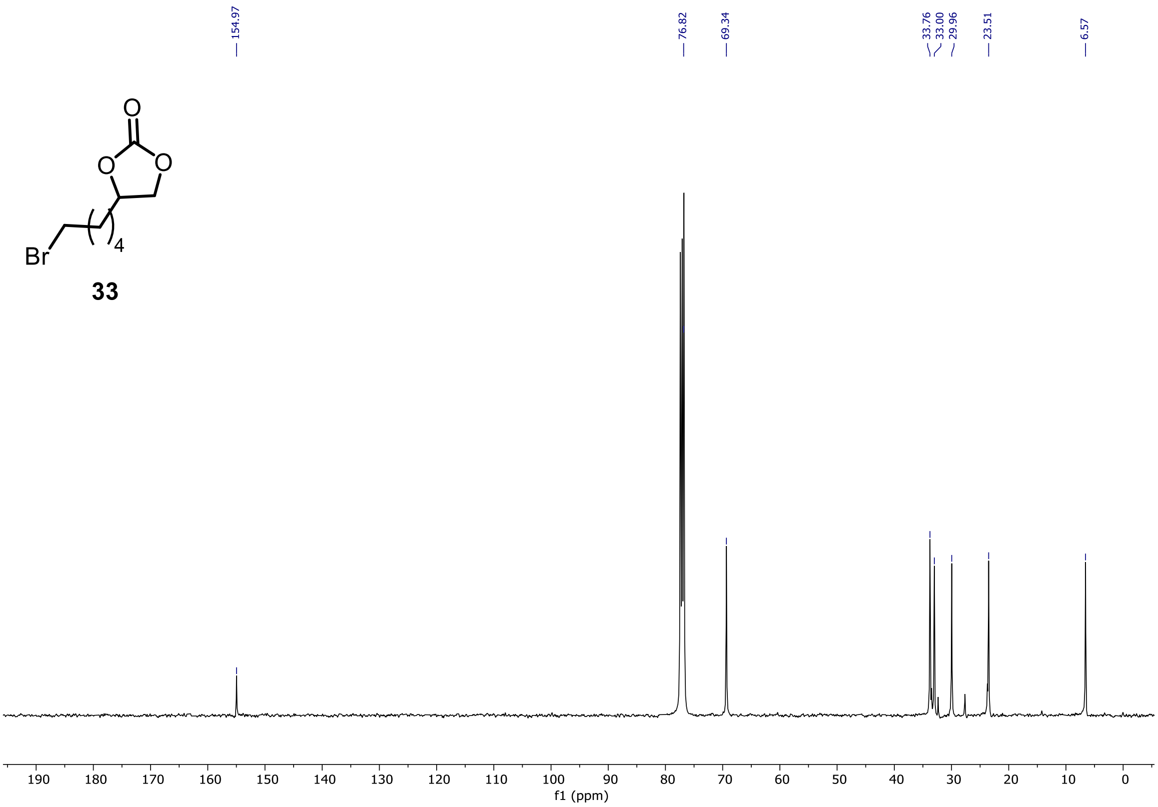

$^{13}C\{^1H\}$ NMR spectrum of compound **33** (101 MHz, $CDCl_3$)

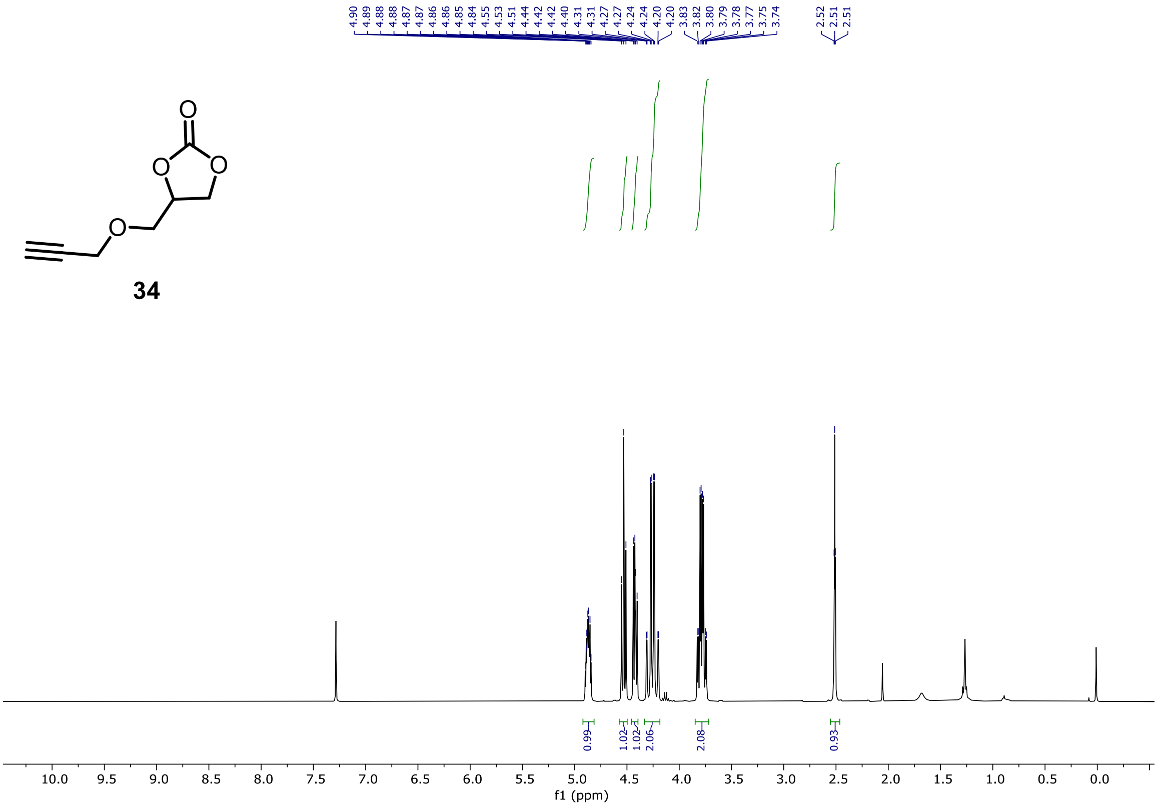

^{1}H NMR spectrum of compound **34** (400 MHz, $CDCl_3$)

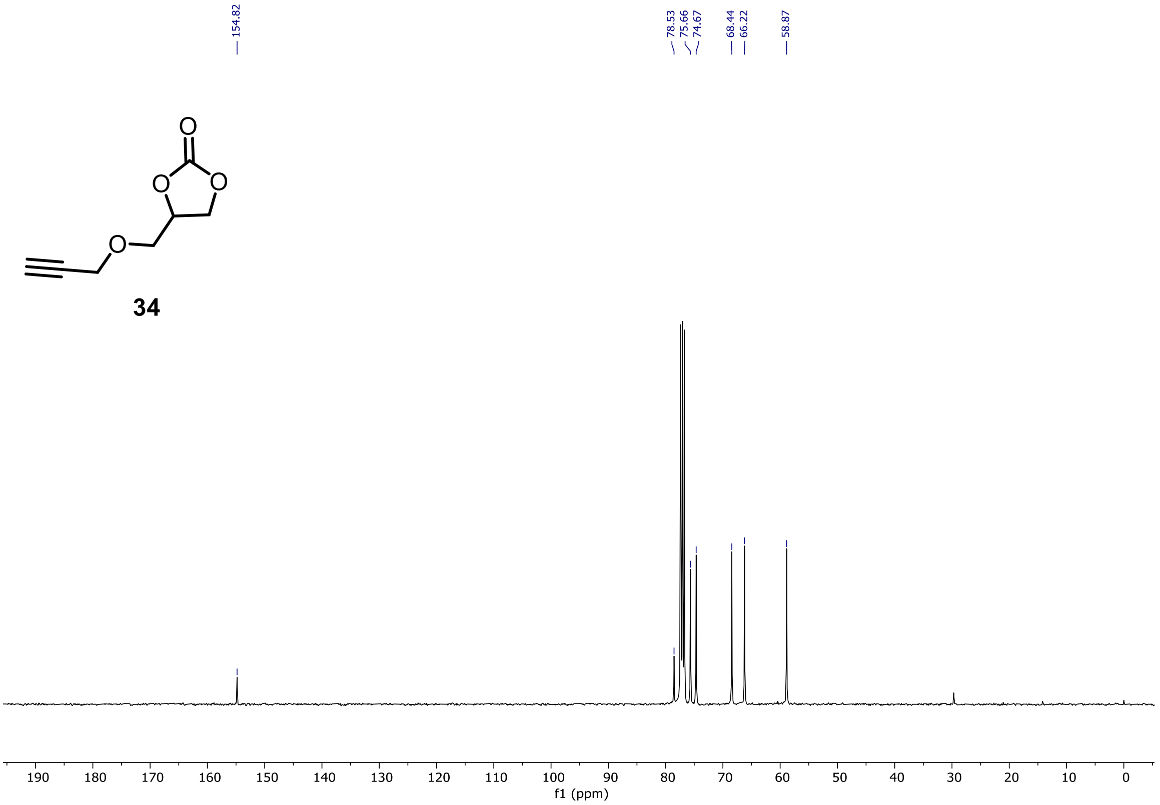

$^{13}C\{^{1}H\}$ NMR spectrum of compound **34** (101 MHz, $CDCl_3$)

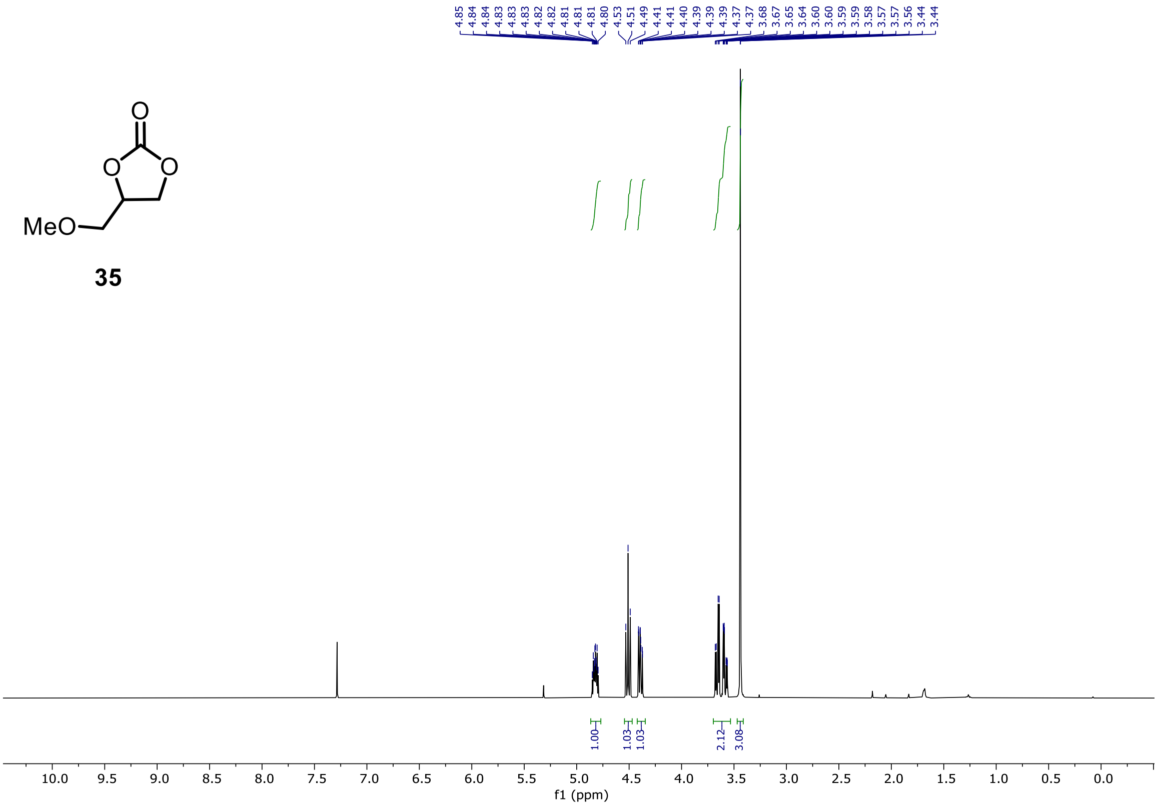

^{1}H NMR spectrum of compound **35** (400 MHz, $CDCl_3$)

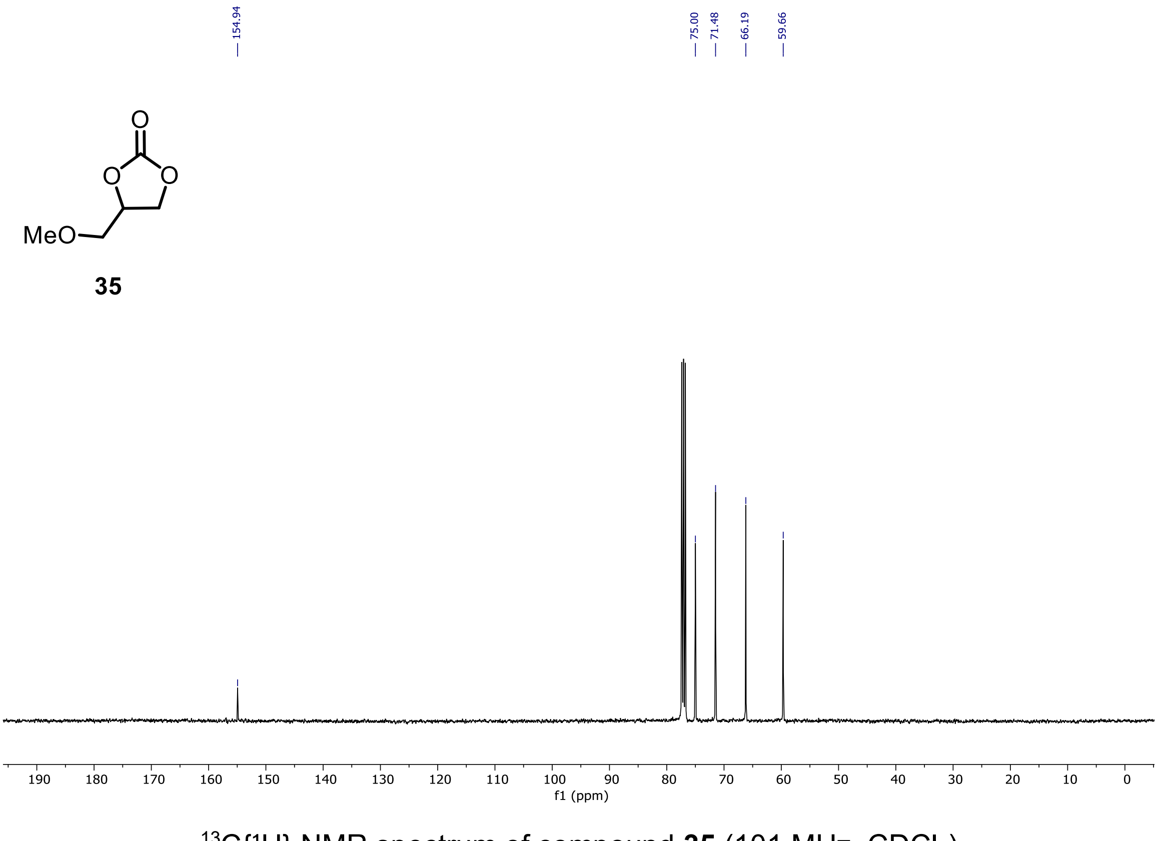

$^{13}C\{^{1}H\}$ NMR spectrum of compound **35** (101 MHz, $CDCl_3$)

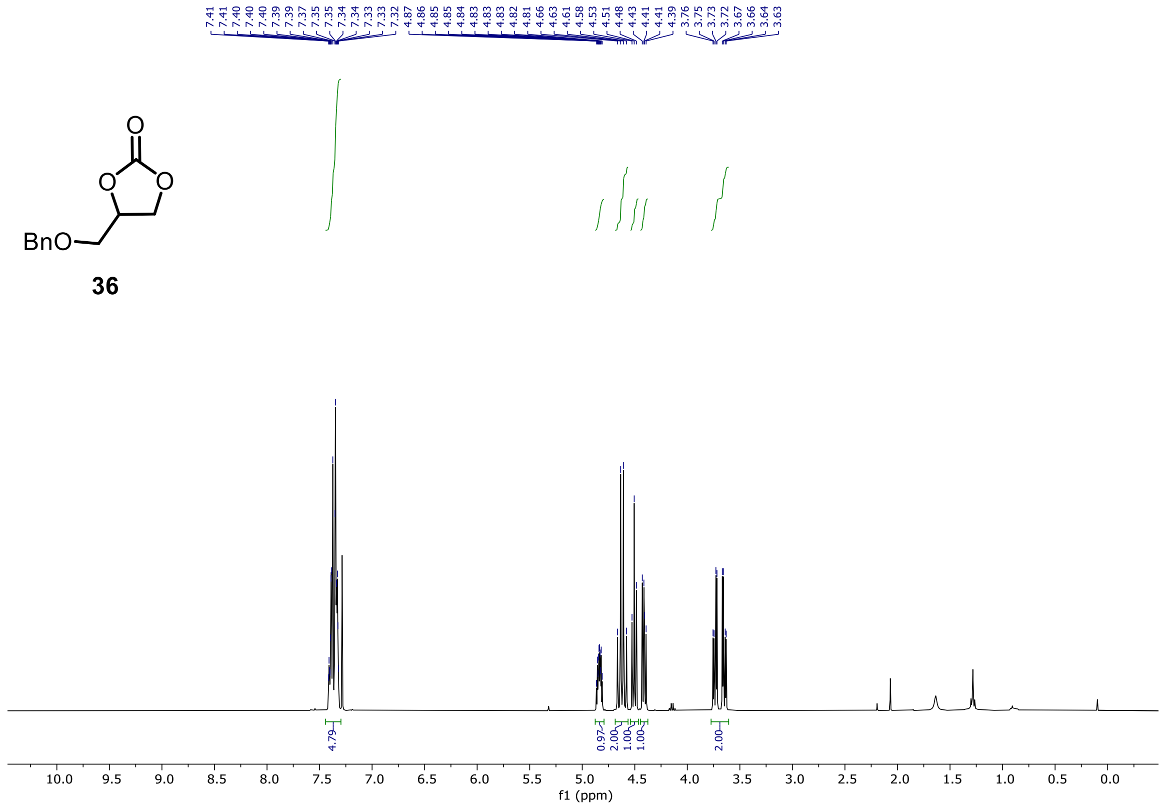

^{1}H NMR spectrum of compound **36** (400 MHz, $CDCl_3$)

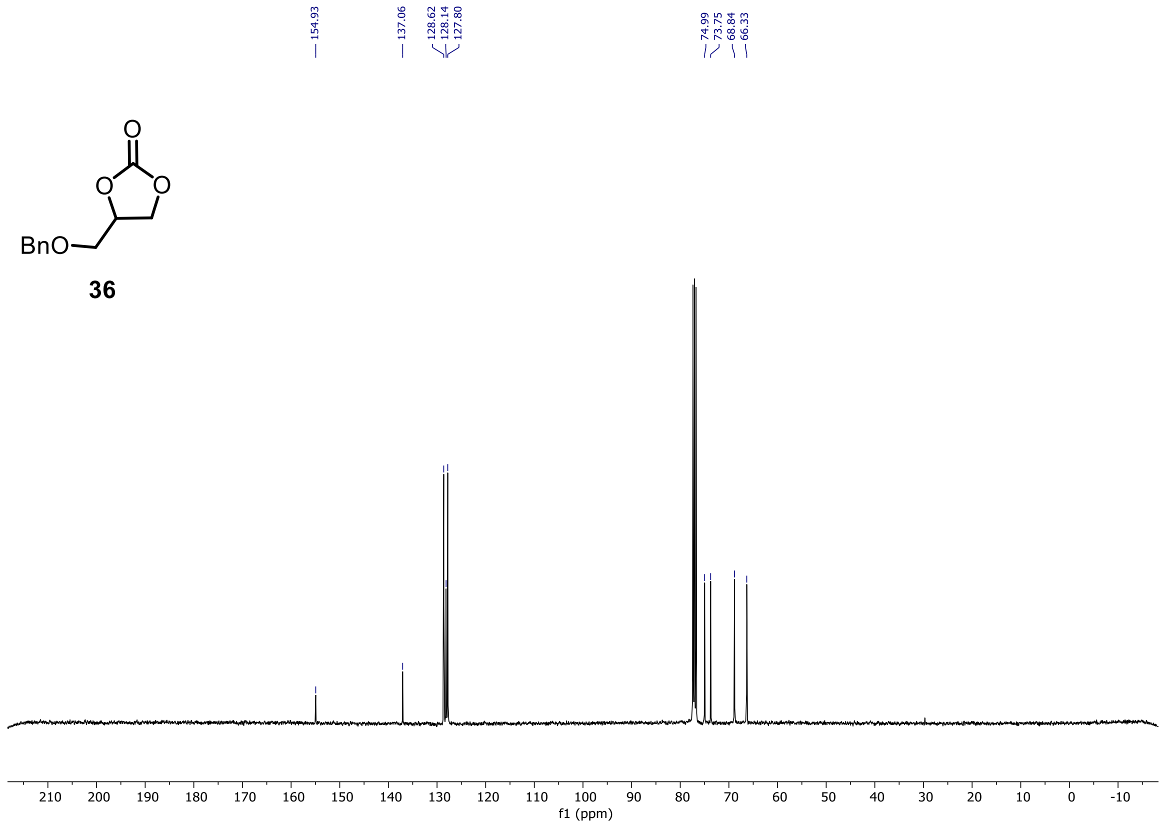

$^{13}C\{^{1}H\}$ NMR spectrum of compound **36** (101 MHz, $CDCl_3$)

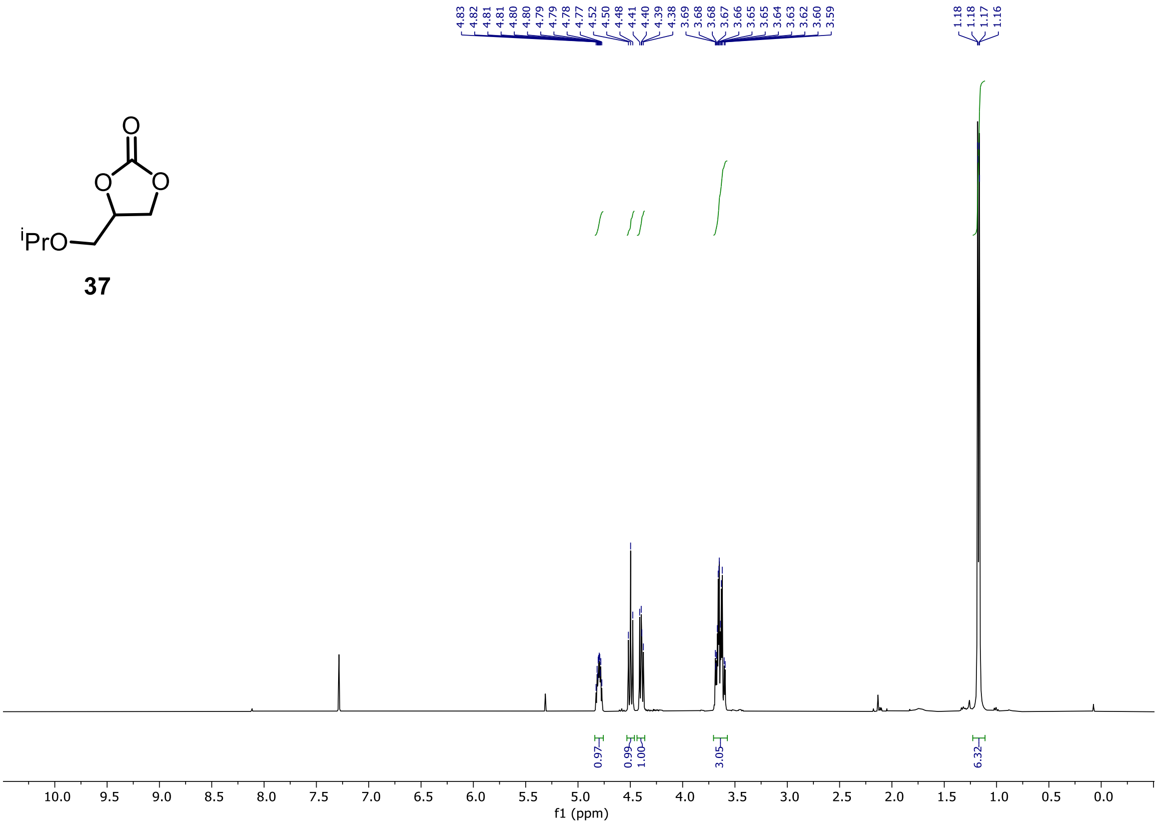

^{1}H NMR spectrum of compound **37** (400 MHz, $CDCl_3$)

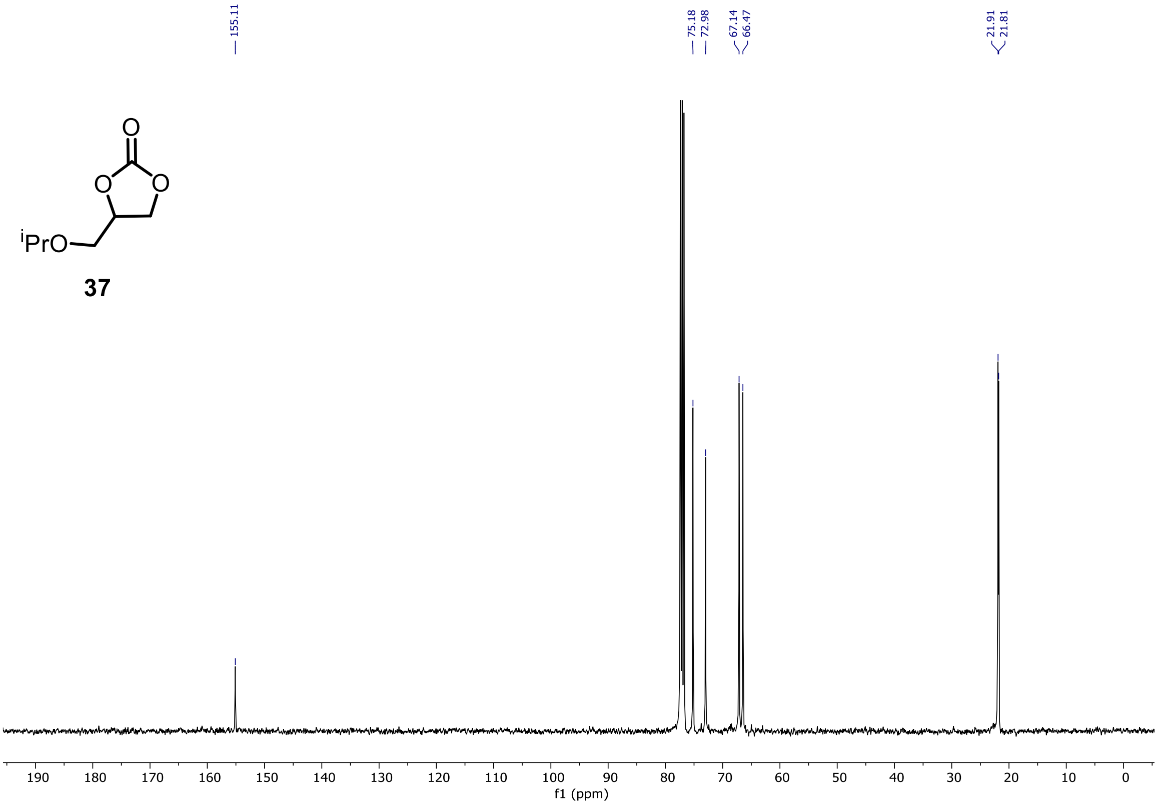

^{13}C{^{1}H} NMR spectrum of compound **37** (101 MHz, $CDCl_3$)

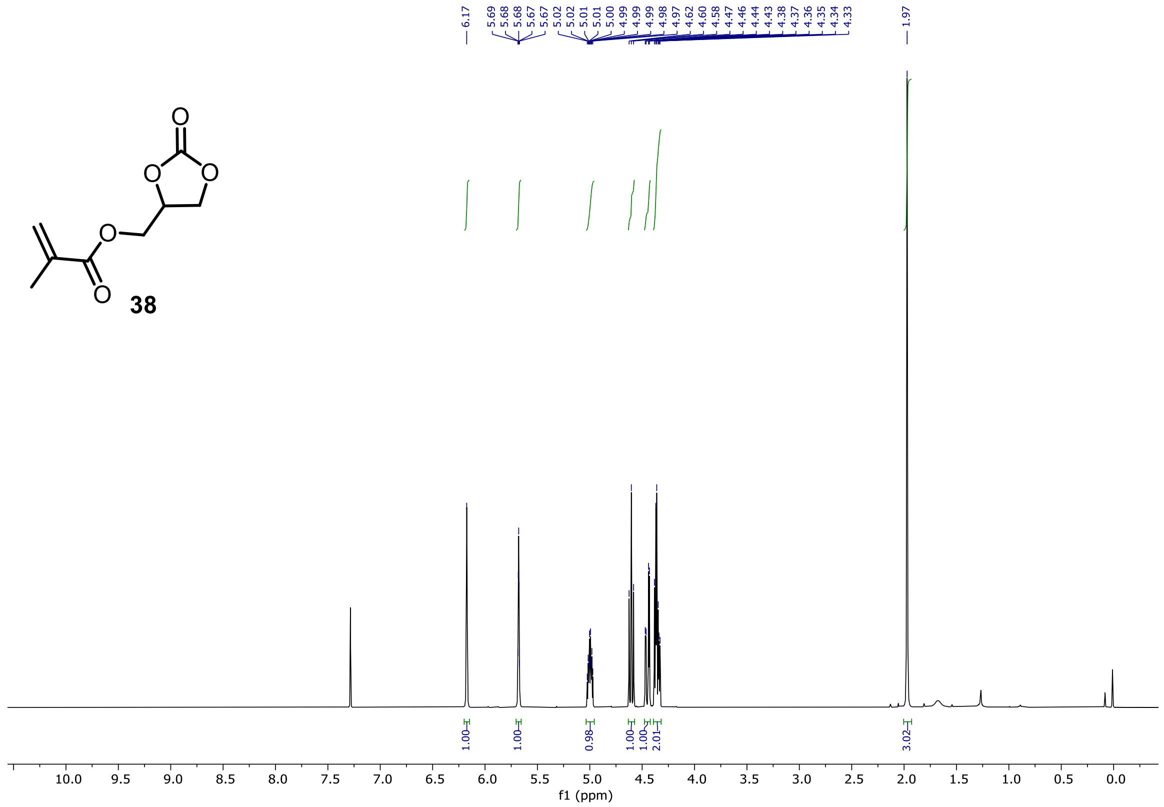

^{1}H NMR spectrum of compound **38** (400 MHz, $CDCl_3$)

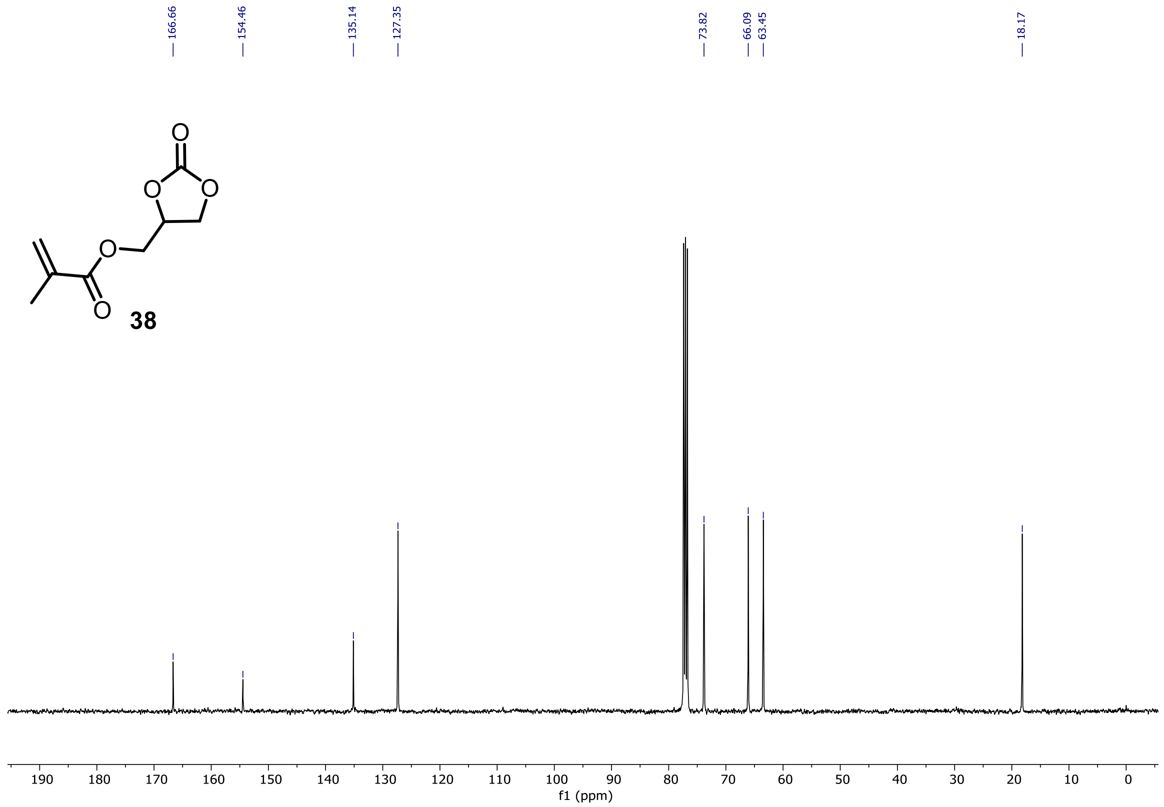

$^{13}C\{^{1}H\}$ NMR spectrum of compound **38** (101 MHz, $CDCl_3$)

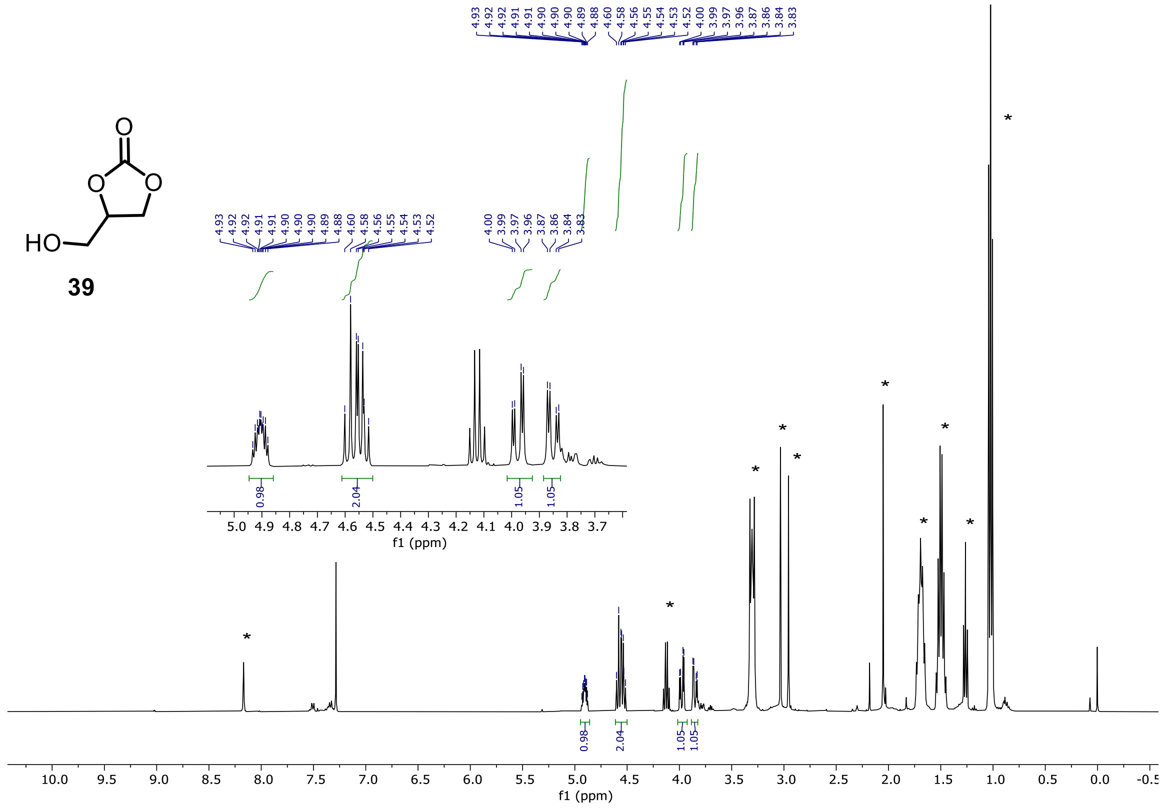

^{1}H NMR spectrum of compound **39** (400 MHz, $CDCl_3$). * represents the residual of DMF, EtOAc and TBAI

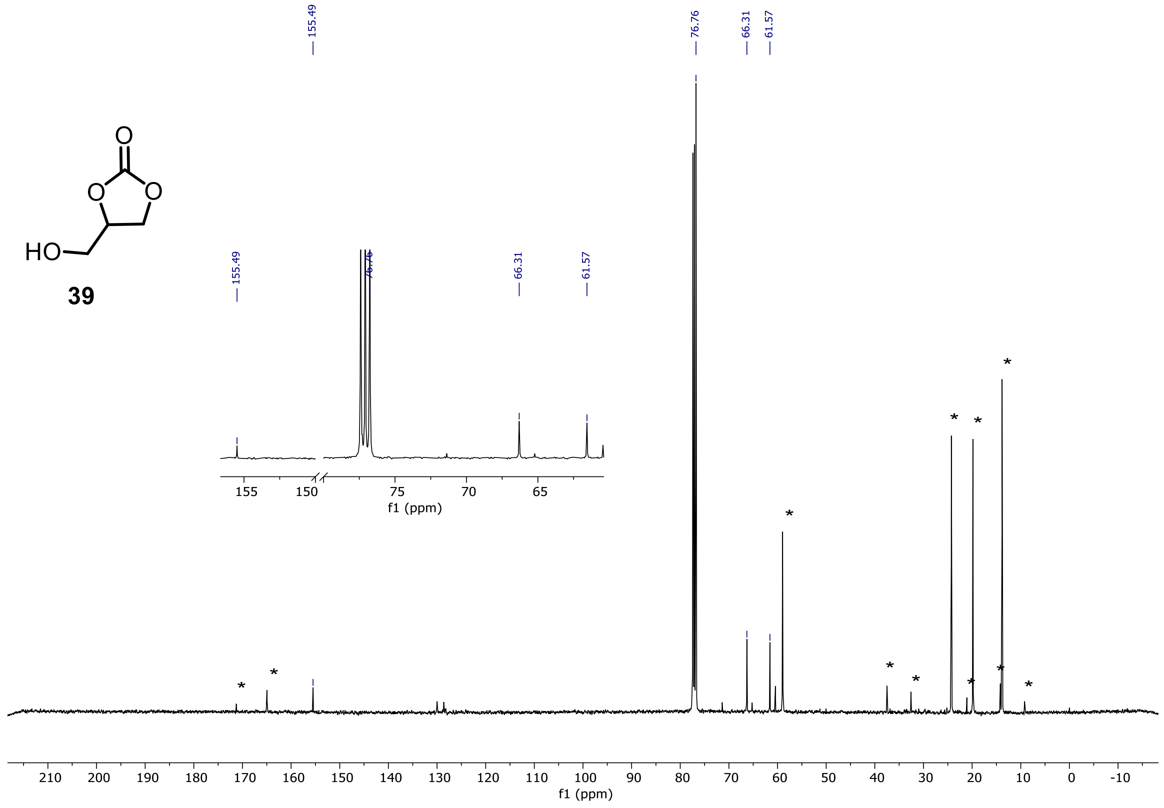

^{13}C{^{1}H} NMR spectrum of compound **39** (101 MHz, $CDCl_3$). * represents the residual of DMF, EtOAc and TBAI

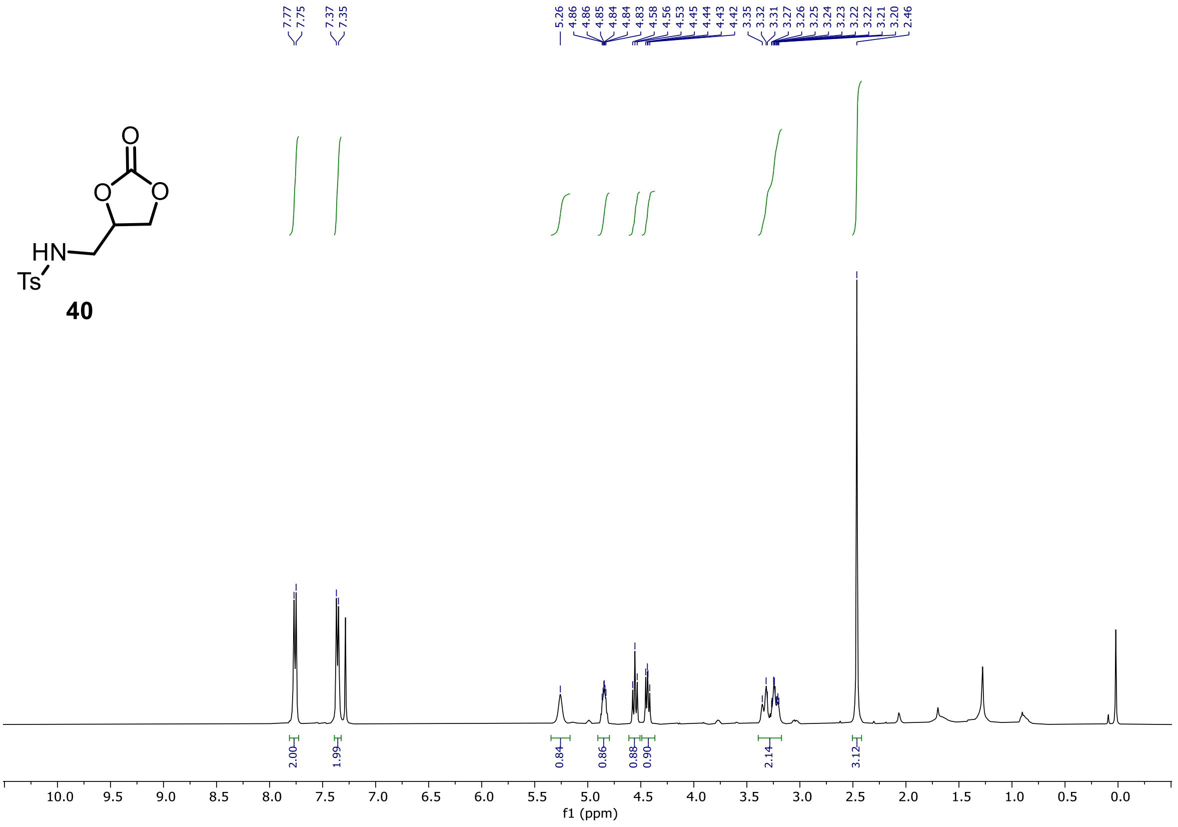

^{1}H NMR spectrum of compound **40** (400 MHz, $CDCl_3$)

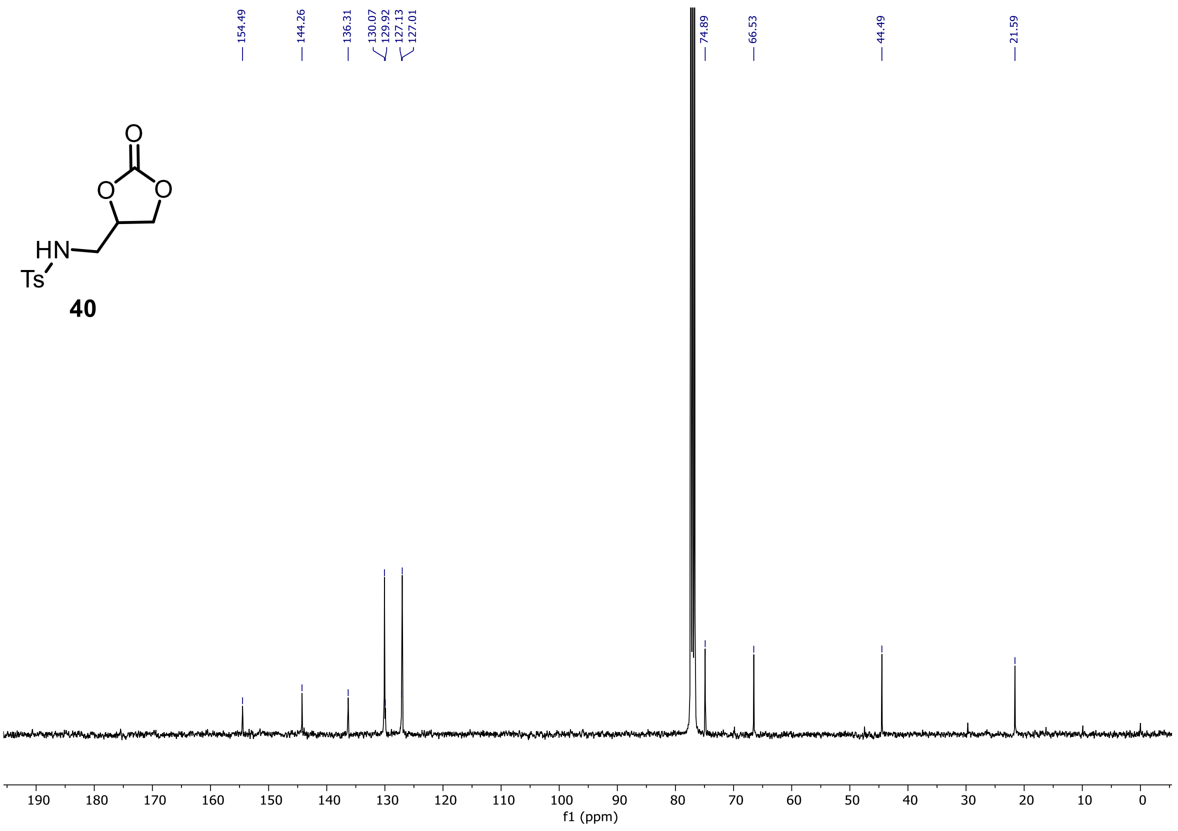

$^{13}C\{^{1}H\}$ NMR spectrum of compound **40** (101 MHz, $CDCl_3$)

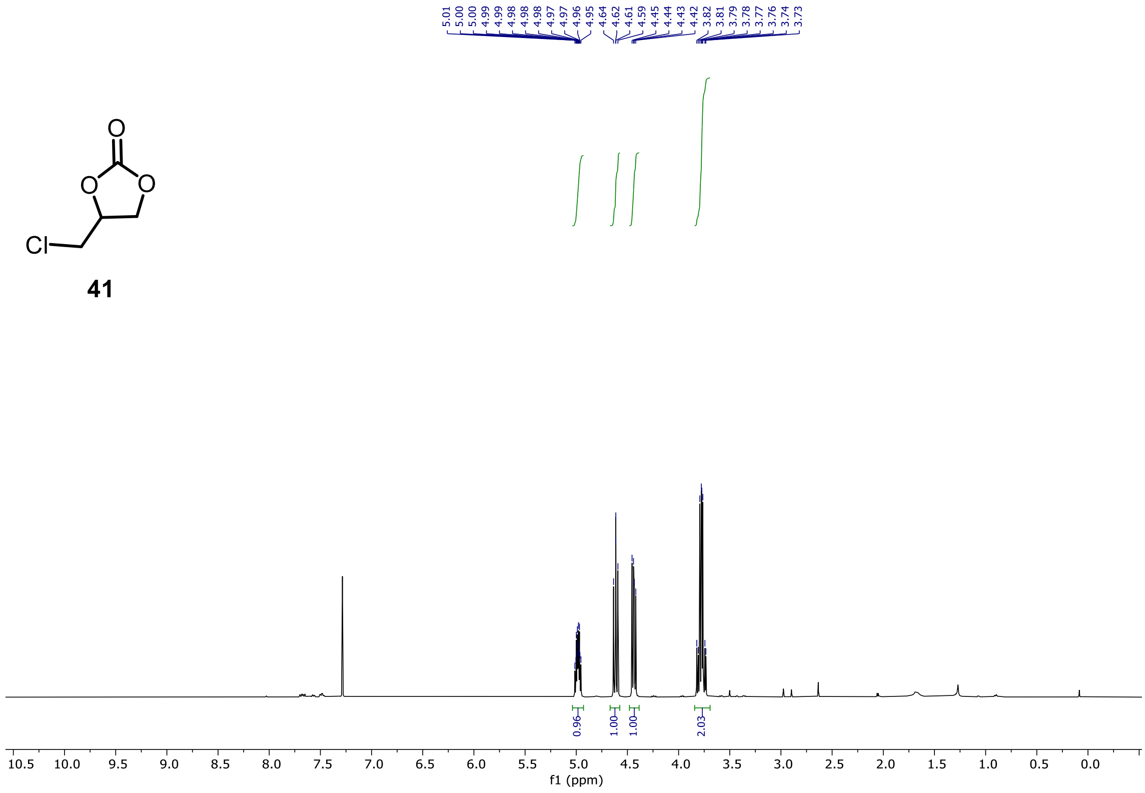

^{1}H NMR spectrum of compound **41** (400 MHz, $CDCl_3$)

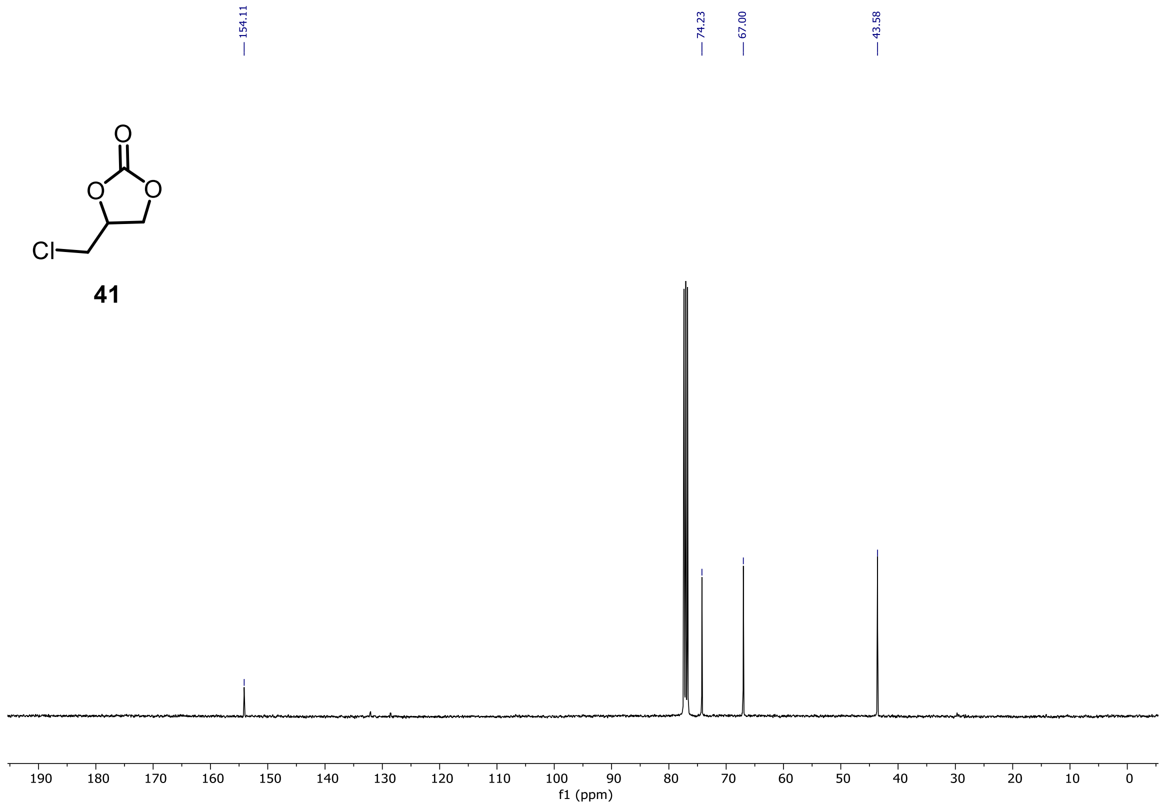

$^{13}C\{^{1}H\}$ NMR spectrum of compound **41** (101 MHz, $CDCl_3$)

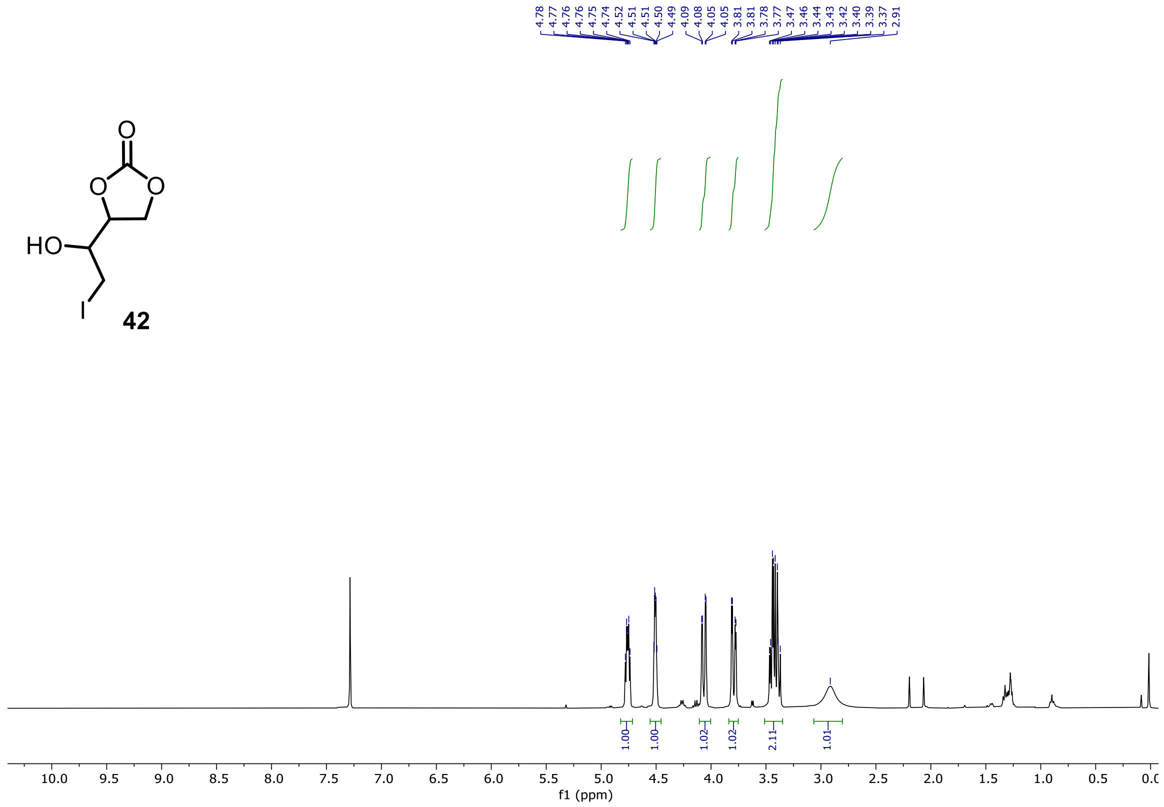

^{1}H NMR spectrum of compound **42** (400 MHz, $CDCl_3$)

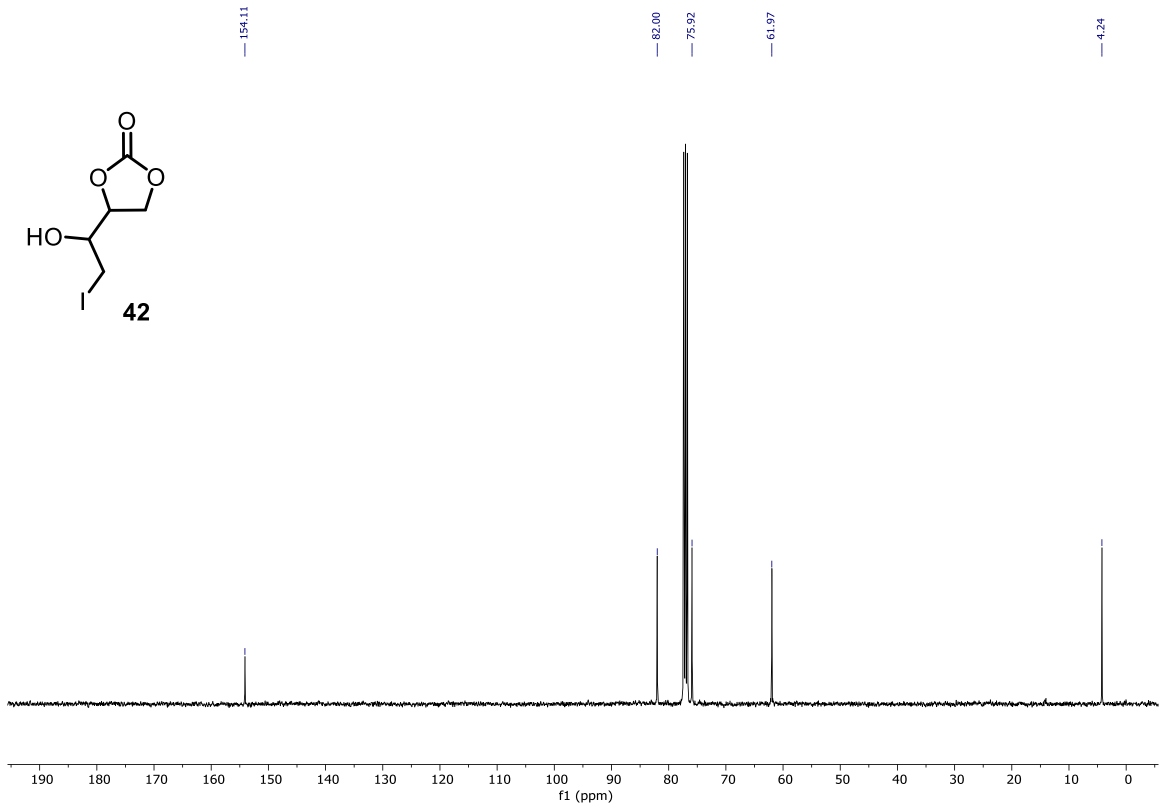

$^{13}C\{^{1}H\}$ NMR spectrum of compound **42** (101 MHz, $CDCl_3$)

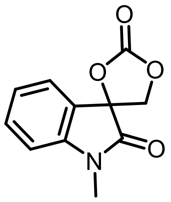

43

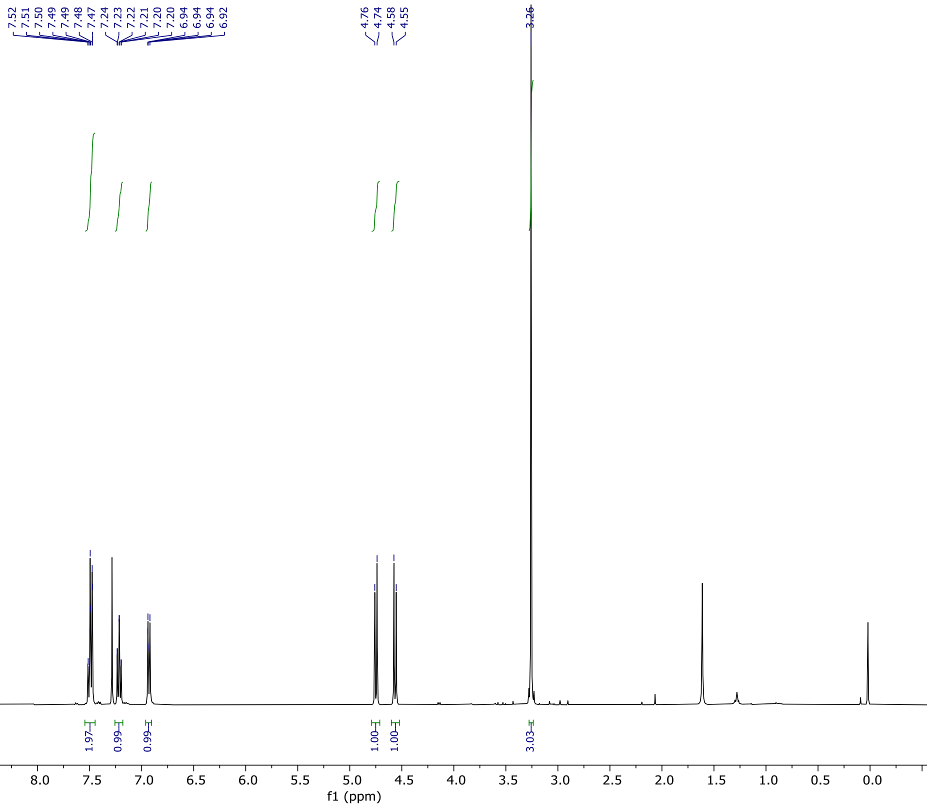

^{1}H NMR spectrum of compound **43** (400 MHz, $CDCl_3$)

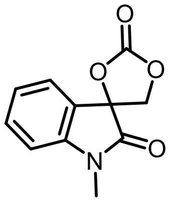

43

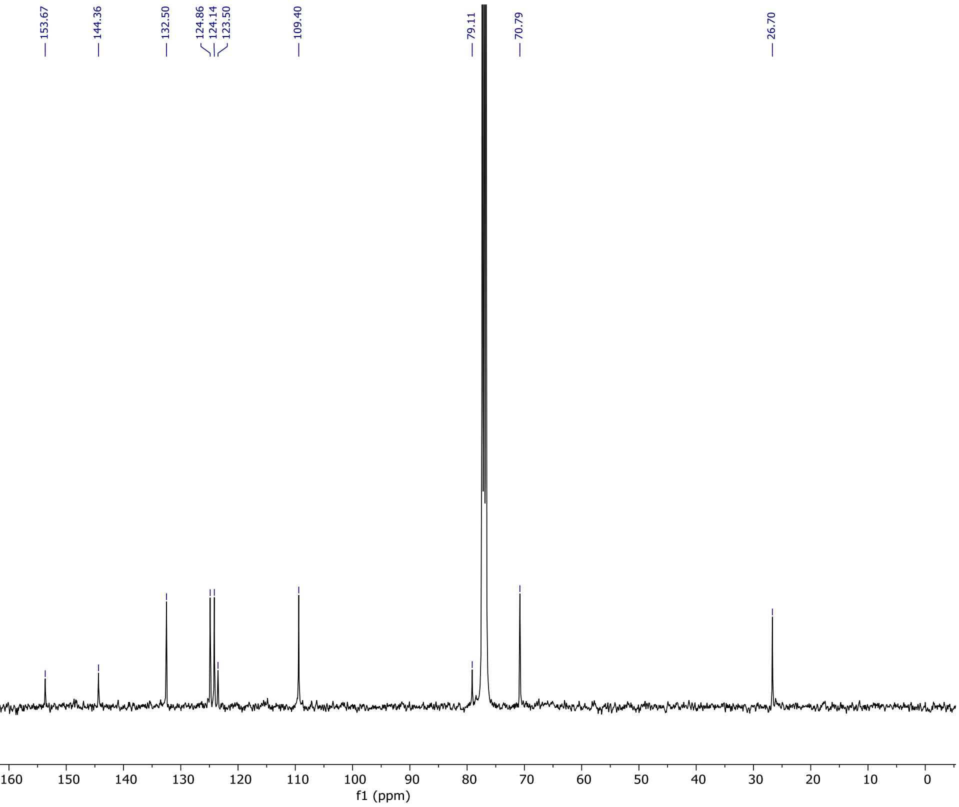

$^{13}C\{^1H\}$ NMR spectrum of compound **43** (101 MHz, $CDCl_3$)

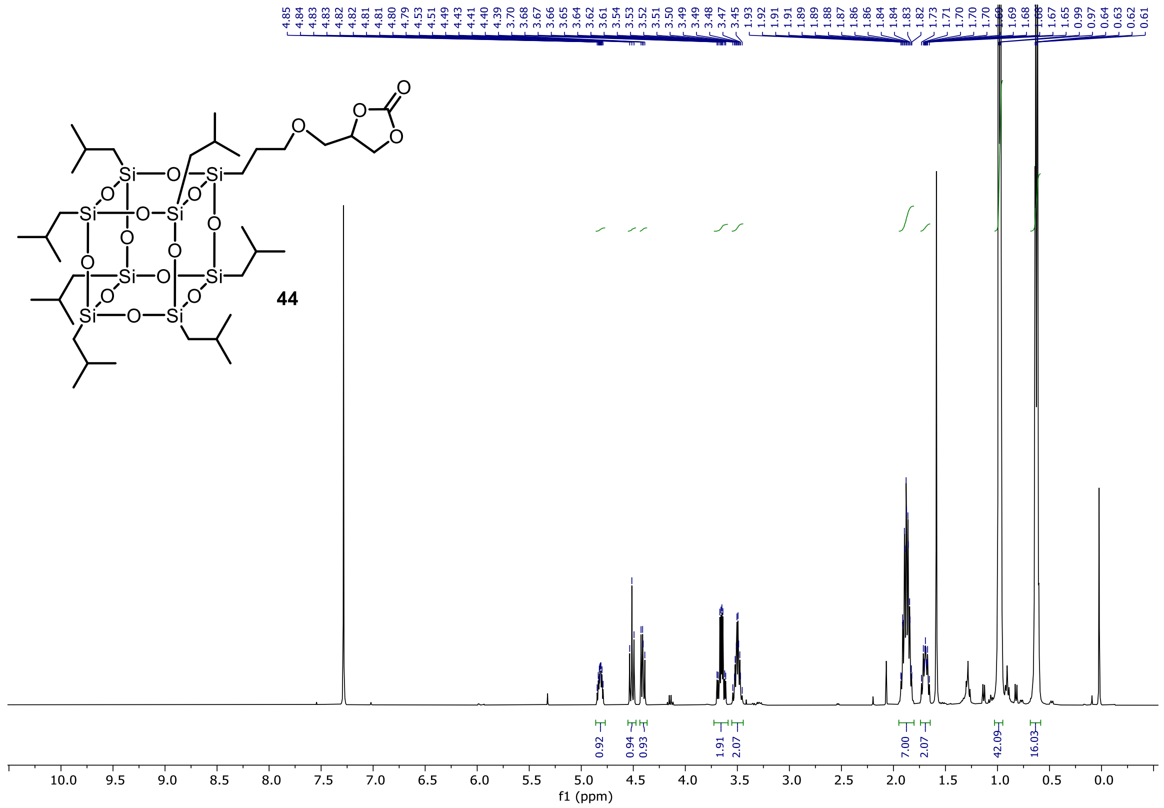

^{1}H NMR spectrum of compound **44** (400 MHz, $CDCl_3$)

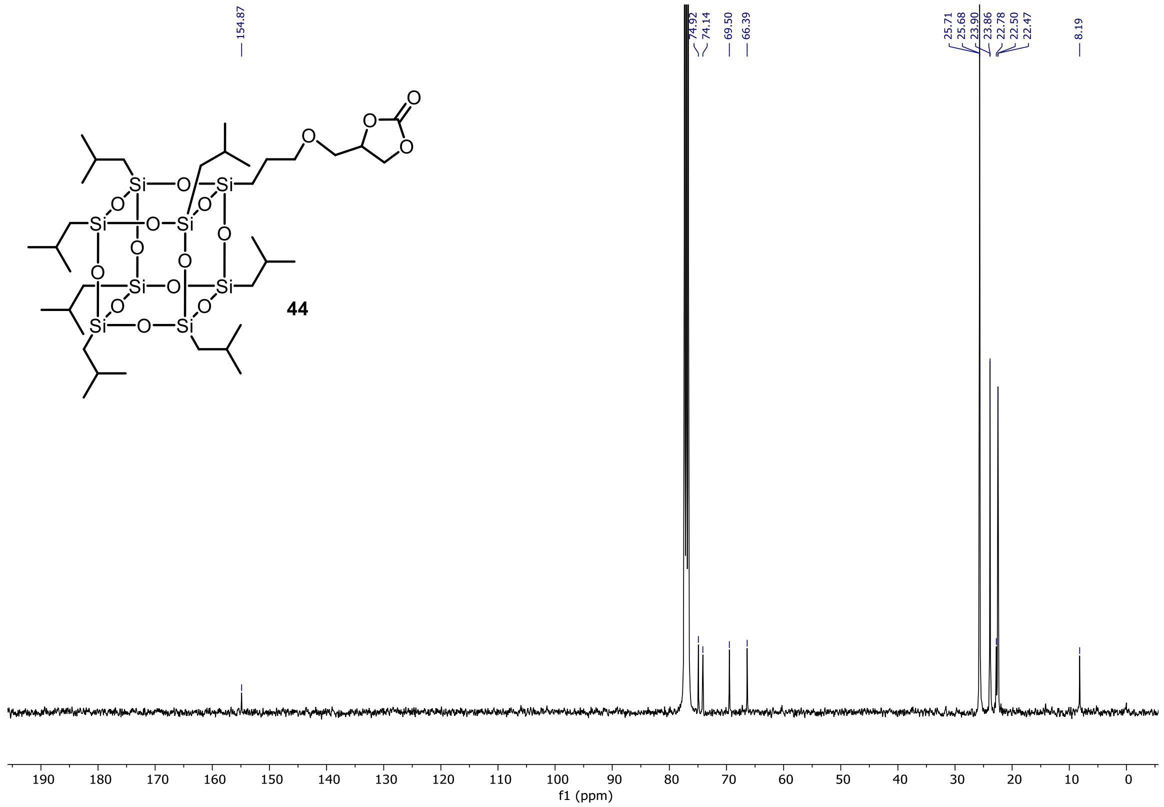

$^{13}C\{^1H\}$ NMR spectrum of compound **44** (101 MHz, $CDCl_3$)

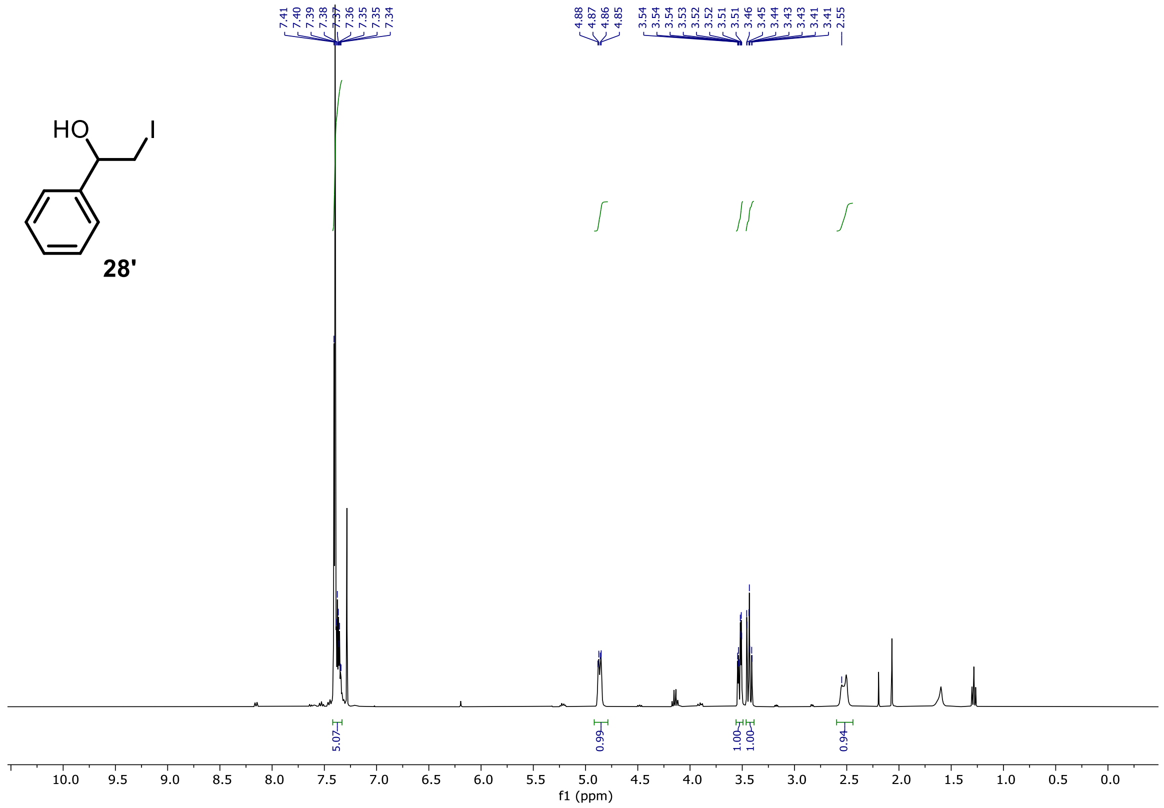

^{1}H NMR spectrum of compound **28’** (400 MHz, $CDCl_3$)

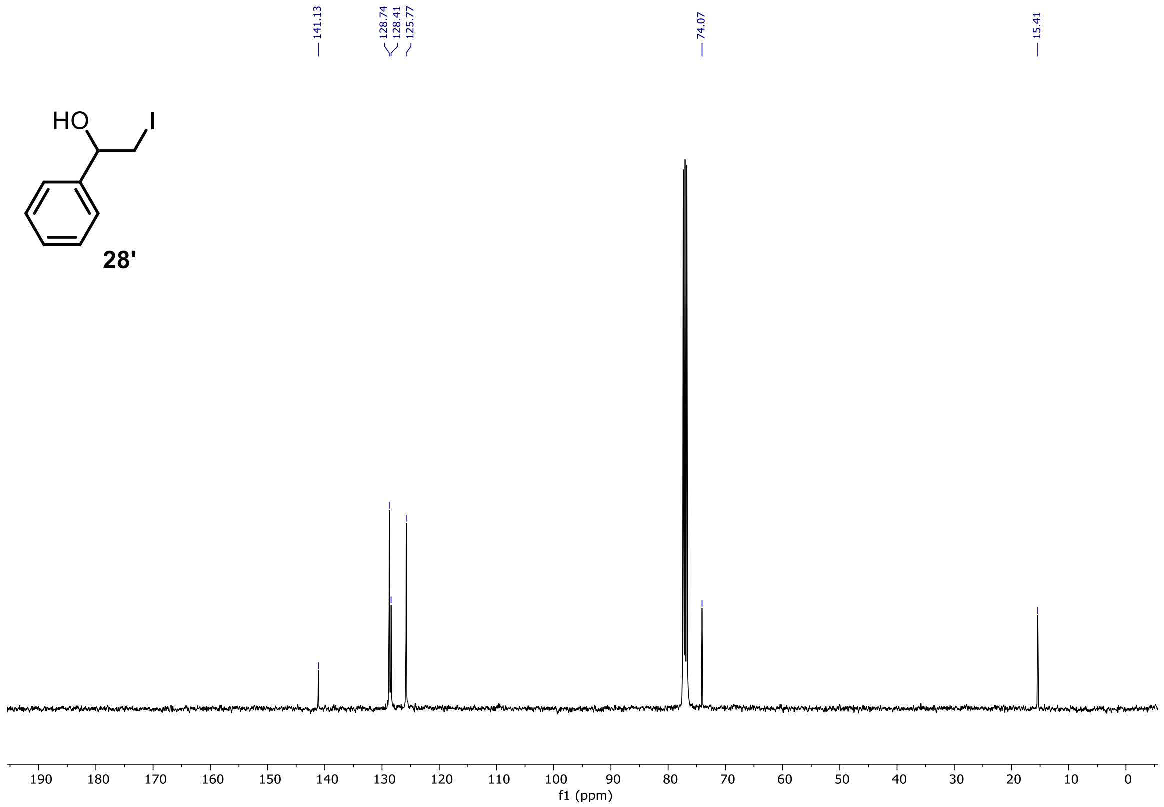

$^{13}C\{^{1}H\}$ NMR spectrum of compound **28’** (101 MHz, $CDCl_3$)

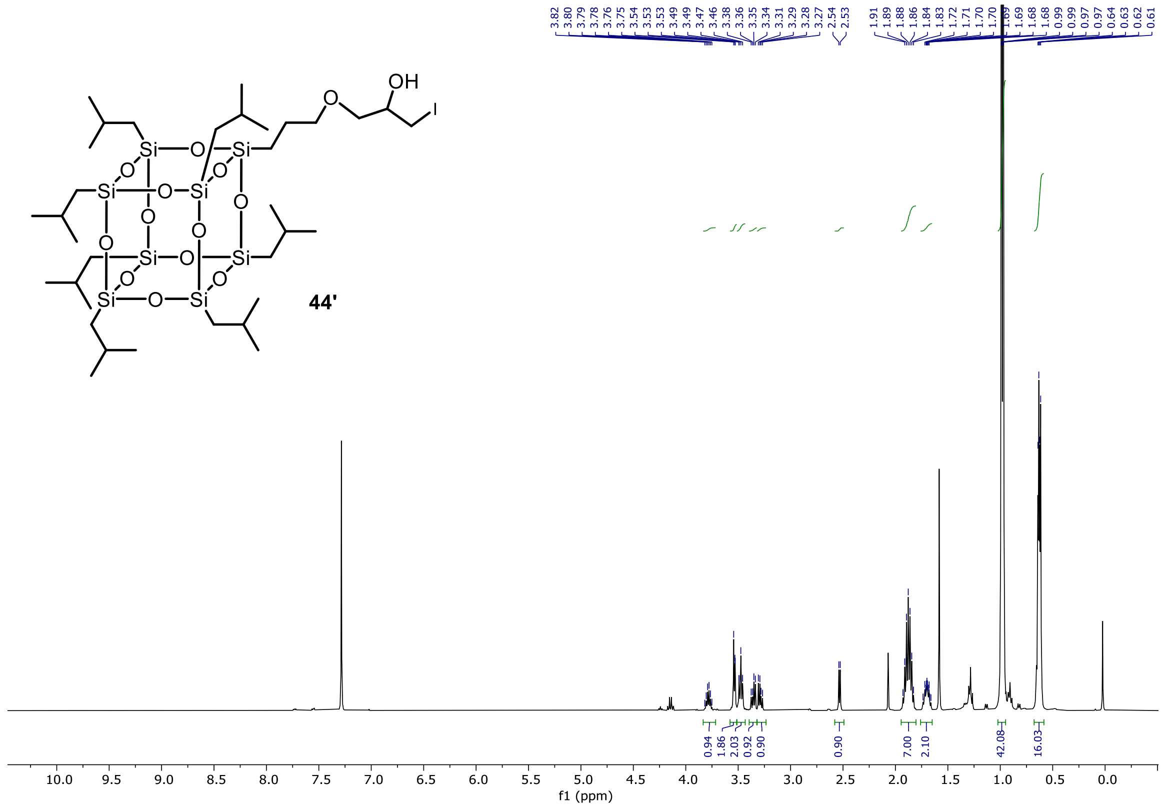

^{1}H NMR spectrum of compound **44’** (400 MHz, $CDCl_3$)

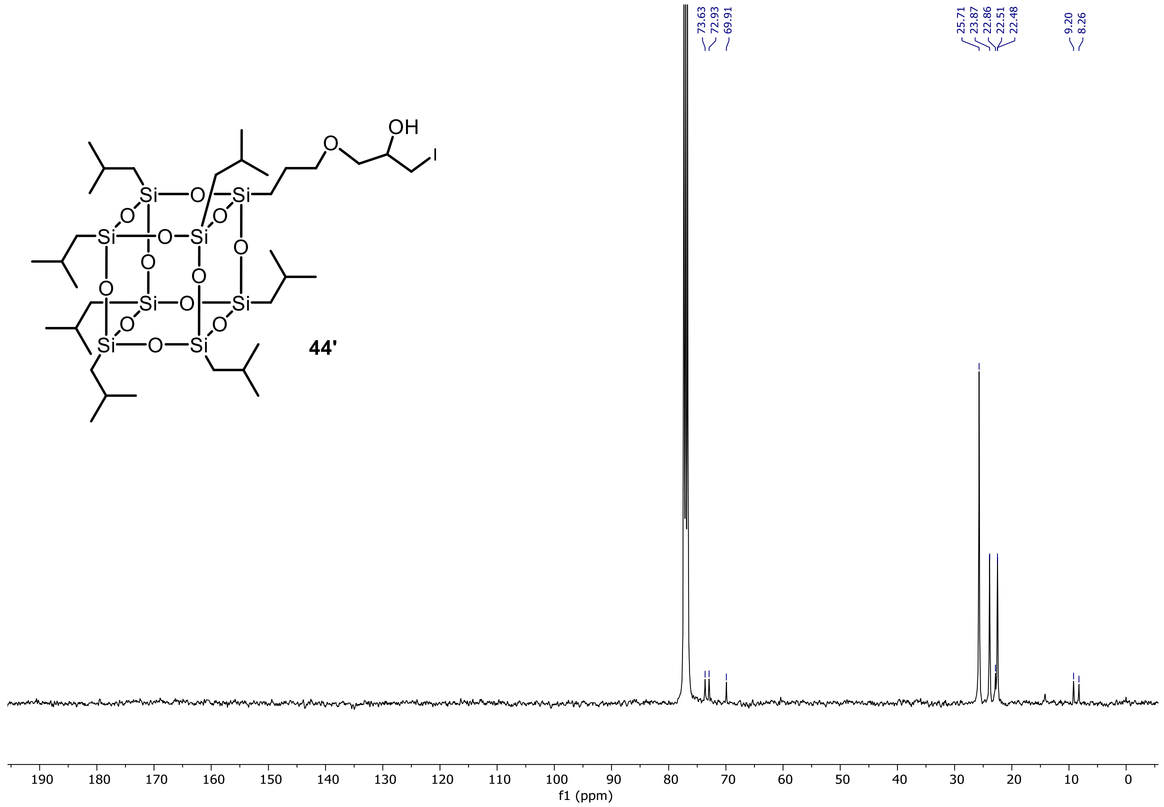

^{13}C{^{1}H} NMR spectrum of compound **44’** (101 MHz, $CDCl_3$)

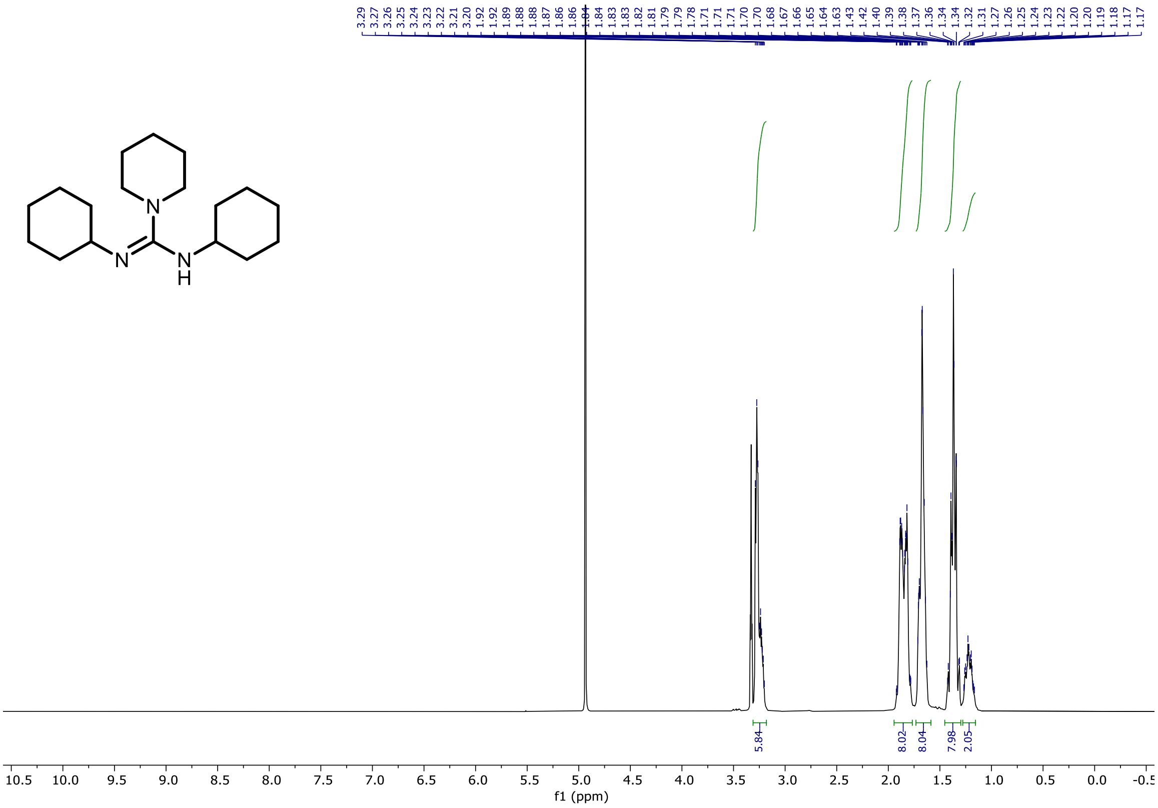

^{1}H NMR spectrum of compound **G** (400 MHz, CD_3OD_SPE)

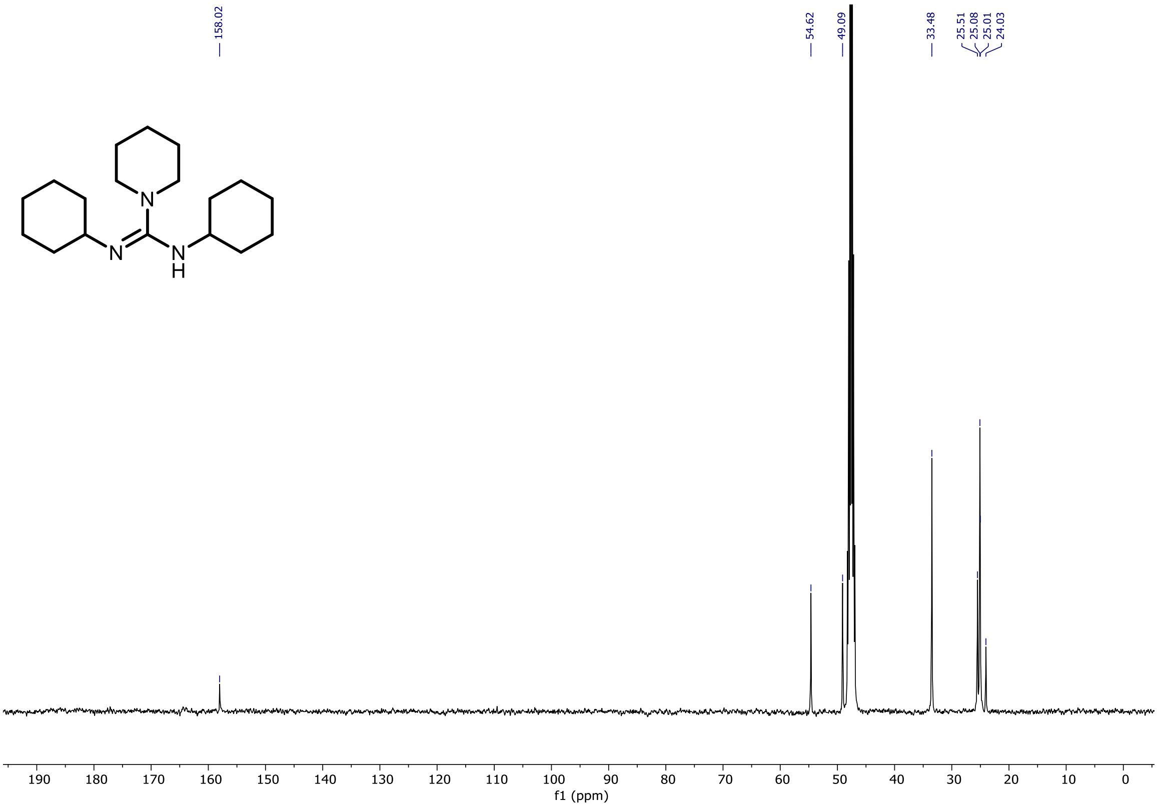

$^{13}C\{^{1}H\}$ NMR spectrum of compound **G** (101 MHz, CD_3OD_SPE)

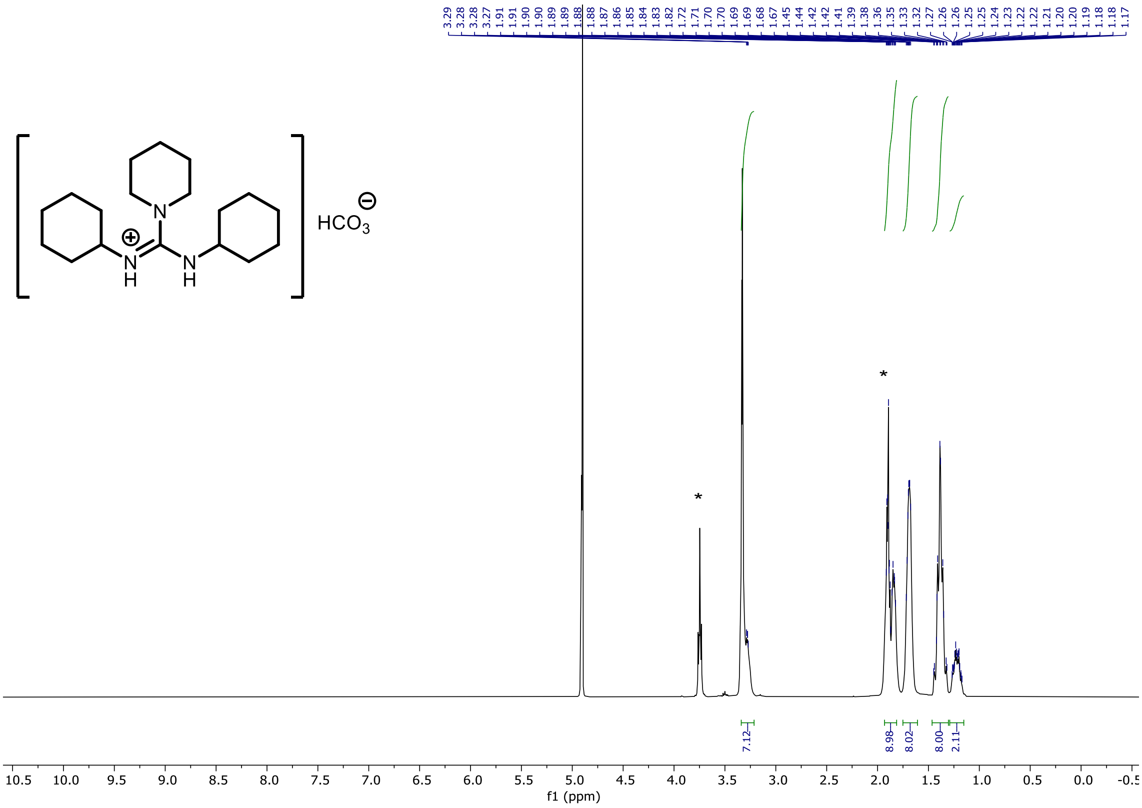

^{1}H NMR spectrum of compound **[GH][HCO₃]** (400 MHz, CD_3OD_SPE). * represents the residual of THF

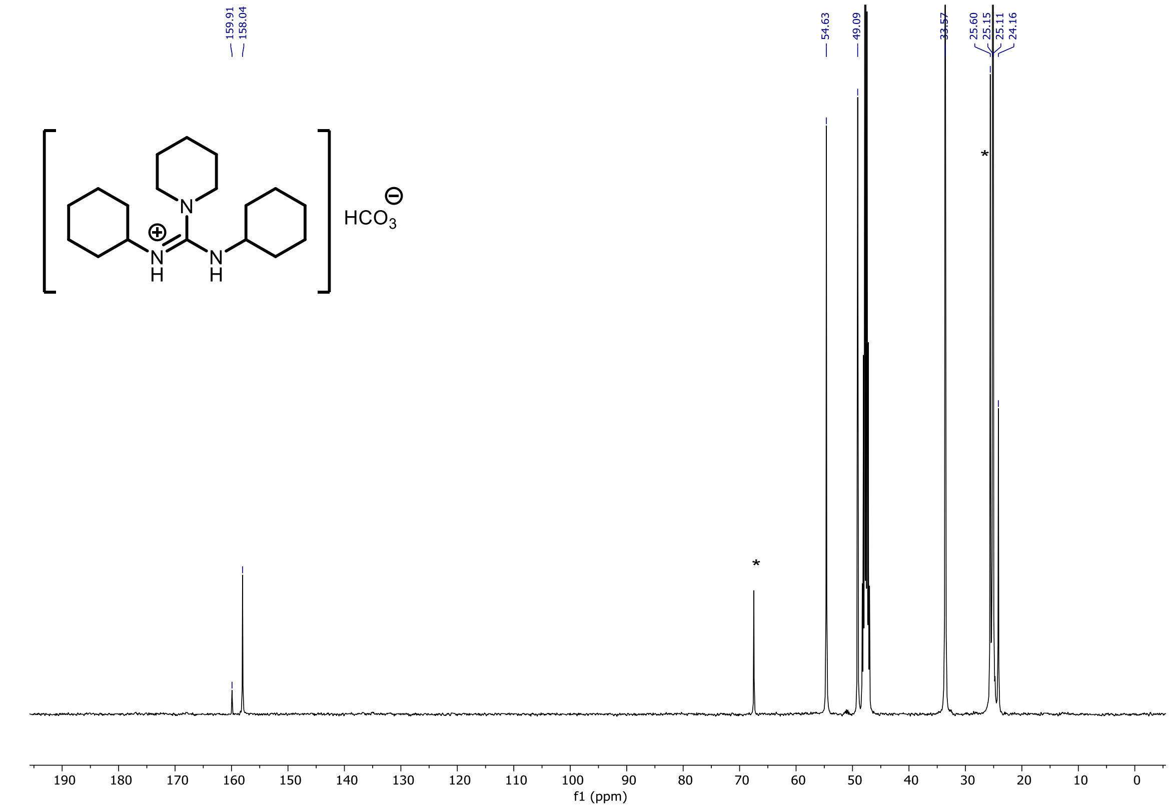

^{13}C{^{1}H} NMR spectrum of compound **[GH][HCO₃]** (101 MHz, CD_3OD_SPE). * represents the residual of THF
